# Assessing a multicomponent quality improvement strategy for chronic care of cardiovascular diseases in India: The C-QIP randomized feasibility trial

**DOI:** 10.1371/journal.pmed.1004932

**Published:** 2026-09-29

**Authors:** Kavita Singh, Ambuj Roy, Dimple Kondal, Kalyani Nikhare, Mareesha Gandral, Satish G. Patil, Kiran Aithal, Meenahalli Palleda Girish, Mohit Gupta, Kushal Madan, Jitendra P. S. Sawhney, Kamar Ali, Meetushi Jain, Savitesh Kushwaha, Devraj Jindal, Emily Mendenhall, Shivani A. Patel, Kabayam M. Venkat Narayan, Nikhil Tandon, Mark D. Huffman, Dorairaj Prabhakaran

**Affiliations:** 1 Heidelberg Institute of Global Health, Heidelberg University, Heidelberg, Germany; 2 Public Health Foundation of India, New Delhi, India; 3 Centre for Chronic Disease Control, New Delhi, India; 4 All India Institute of Medical Sciences, New Delhi, India; 5 SDM College of Medical Sciences and Hospital, Shri Dharmasthala Manjunatheshwara University, Dharwad, Karnataka, India; 6 GB Pant Hospital, New Delhi, India; 7 Sir Ganga Ram Hospital, New Delhi, India; 8 Georgetown University, Washington, District of Columbia, United States of America; 9 Emory Global Diabetes Research Center of Woodruff Health Sciences Center and Emory University, Atlanta, Georgia, United States of America; 10 Washington University School of Medicine, St. Louis, Missouri, United States of America; 11 The George Institute for Global Health, University of New South Wales, Sydney, Australia; 12 University of Abuja, Abuja, Nigeria; South African Medical Research Council, SOUTH AFRICA

## Abstract

**Background:**

Chronic cardiovascular diseases (CVD) care quality remains suboptimal, globally. We assessed the feasibility, acceptability, fidelity, and preliminary effect of a multicomponent, collaborative quality improvement (C-QIP) strategy among patients with CVD attending outpatient clinics in India.

**Methods and findings:**

In this pragmatic feasibility randomized controlled trial, 410 adults with ischemic heart disease, ischemic stroke, or heart failure attending four public and private hospitals were randomized to C-QIP (electronic decision support for providers, non-physician care coordination, patient education, text-message reminders, and audit-feedback) or usual care. Primary outcomes were feasibility, fidelity, adoption, and acceptability; secondary outcomes included guideline-directed medical therapy (GDMT), self-reported adherence, care processes, and risk factors. At end of study (median follow-up 15 months), retention was 192/206 (93.2%) in C-QIP and 187/204 (91.7%) in usual care. Fidelity was high: 187/198 (94.4%) C-QIP participants received lifestyle counseling at end of study. C-QIP increased GDMT use for ischemic heart disease (58.3% versus 32.4%; relative risk [RR] 1.45, 95% CI [1.18, 1.78]) and ischemic stroke (76.7% versus 31.8%; RR 2.41, 95% CI [1.52, 3.81]), but not heart failure. Self-reported adherence was higher for medications (90.9% versus 82.3%; RR 1.08, 95% CI [1.04, 1.12]), diet (91.9% versus 82.3%; RR 1.07, 95% CI [1.02, 1.13]), and physical activity (91.4% versus 70.4%; RR 1.23, 95% CI [1.16, 1.30]). Limitations include feasibility design, individual randomization within shared clinics, and limited power for clinical outcomes and subgroup analysis.

**Conclusions:**

The C-QIP trial demonstrated that a multicomponent strategy is feasible, acceptable, and improved chronic CVD care processes in India. Future large, confirmatory hybrid trials are needed to establish whether such quality improvement strategies can reduce cardiovascular morbidity and mortality.

**Trial Registrations:**

NCT05196659; CTRI/2022/04/041847

## Author summary

**Why was this study done?:**

- Many people living with heart disease, stroke, or heart failure do not consistently receive the long-term medicines and follow-up care known to prevent future cardiovascular events.
- In India, outpatient care for chronic cardiovascular disease is often fragmented, and practical strategies are needed to help clinics deliver optimal guideline-based care.
- We tested whether a multicomponent strategy combining care coordination, clinical decision support, patient reminders, education, and audit-feedback could be delivered in routine outpatient clinics to improve cardiovascular disease care in India.

**What did the researchers do and find?:**

- We randomized 410 adults with ischemic heart disease, ischemic stroke, or heart failure at four hospitals in India to receive the C-QIP strategy or usual care.
- The strategy was feasible and acceptable: more than 90% of participants completed the final study visit, and most patients and providers reported willingness to continue using the C-QIP strategy.
- Compared with usual care, C-QIP improved use of recommended combination of cardiovascular preventive medicines for ischemic heart disease and ischemic stroke, and improved self-reported adherence to medicines, diet, and physical activity.

**What do these findings mean?:**

- A multicomponent quality-improvement strategy can be implemented into routine outpatient clinics in India and can improve cardiovascular care processes.
- The study was not designed to prove effects on heart attacks, strokes, deaths, or long-term risk-factor control, so these results should be interpreted as feasibility and preliminary effectiveness findings.
- Future larger studies should use cluster-randomized designs to reduce contamination between treatment groups and assess whether this approach improves clinical outcomes at scale.

## Abstract 3

Kavita Singh and colleagues evaluated a multicomponent quality improvement strategy for chronic cardiovascular disease care in India and found it was feasible to implement, well accepted by patients and providers, and delivered with high fidelity across diverse outpatient settings.

## Background

Cardiovascular diseases (CVD) caused an estimated 20.5 million deaths and 300 million disability-adjusted life years in 2024 [1,2]. The societal and economic consequences are profound, particularly in low- and middle-income countries (LMICs), where CVD affects working-age adults [3,4]. Secondary prevention is a critical strategy to reduce the rapidly growing CVD burden [5]. Despite advances in evidence-based guidelines, wide gaps persist in their implementation and quality of chronic CVD care across diverse healthcare settings [6,7]. For instance, the Prospective Urban Rural Epidemiology (PURE) study underscores the magnitude and persistence of this gap among adults with prior CVD across 17 countries, use of proven secondary prevention drugs was strikingly low overall: antiplatelets 25%, β-blockers 17%, ACEi/ARB 20%, statins 15%, with the lowest use in low-income settings (e.g., statins 3%) [8]. Updated 2025 PURE analyses reported global disparities in CVD drug use over a median follow-up of 12 years and found persistently low and declining use of secondary prevention drugs among people with CVD [9]. Globally, use of ≥1 recommended class (antiplatelet, statin, ACEi/ARB, β-blocker) was ~41% at baseline in 2007, peaked near 43%, then fell to ~31% by the last visit (2019); in high-income countries it decreased from ~89% to ~77%. In India, use of ≥1 secondary-prevention medication class was 21.4% at baseline, rose to a peak of 43.0% by visit 4 (mid-period), and then declined to 21.6% by the last visit; the highest use of ≥3 classes reached 10.7% during follow-up. These data on low use of CVD secondary prevention drugs underscore a stalled (or worsening) implementation gap, especially in lower-resource settings [9].

These gaps further reflect multilevel barriers, including fragmented healthcare delivery, clinical inertia, inadequate universal healthcare coverage, limited patient awareness, and poor adherence to long-term treatment [10,11]. Closing these persistent “know-do” gaps requires pragmatic, context-specific strategies that go beyond single interventions [12,13]. To address these gaps, the Indian government has updated National Programme for Prevention and Control of Noncommunicable Diseases (NP-NCD, 2023 operational guidelines) [14], with emphasis on integrated team-based care, use of digital health tools, task-sharing with non-physician health workers, and strengthening secondary prevention services across public and private systems [15]. In light of this evolving policy context, the C-QIP trial builds on prior implementation trials in India, including, the ACS-QUIK trial [16,17], focused on hospital-based quality improvement intervention in patients with acute coronary syndrome, the SPREAD [18] trial involving community health worker-led intervention to support patients after acute coronary syndrome, and the CARRS Translation Trial [19,20] utilizing multicomponent, technology enabled-intervention for cardiovascular risk reduction in people with poorly controlled type 2 diabetes.

However, strategies that combine system-, provider-, and patient-level interventions that integrates clinical decision support, task-sharing, patient engagement, and audit-feedback to improve the quality of chronic CVD care have not been systematically tested in Indian context. To address this critical gap, we evaluated the feasibility and preliminary effect of a collaborative quality improvement (C-QIP) strategy compared with usual care among patients with CVD attending outpatient clinics in India.

## Methods

### Study design

The C-QIP trial was a multi-site, individual-level randomized (1:1), controlled, open-label, feasibility trial with collection of implementation and effectiveness outcomes to inform a larger outcomes-driven hybrid effectiveness-implementation trial. Full details of the study design and methods have been described previously [21] (This study is reported as per the Consolidated Standards of Reporting Trials (CONSORT) guidelines, S1 Checklist and trial protocol provided in S1 Protocol). The trial protocol was registered prospectively on Clinicaltrials.gov: NCT05196659 and Clinical Trials Registry India: CTRI/2022/04/041847. The C-QIP trial was conducted at four hospitals (2 public and 2 private) located in Delhi and Karnataka in India. The C-QIP trial began recruitment on 12 September 2022 and completed target recruitment on 30 September 2023. Follow-up visits were completed in November 2024.

### Participants

Patients with a confirmed diagnosis of CVD including ischemic heart disease, ischemic stroke, or heart failure irrespective of ejection fraction were eligible for the trial. Patients aged ≥18 years, both sexes, living within same city/town as one of the sites and those willing to provide consent were recruited in the trial. Pregnant women and patients having disease associated with frequent hospitalization (e.g., advanced cancer, end-stage renal disease); bedridden or debilitating conditions, and life expectancy less than 12 months were excluded from the trial. The recruitment goal was 100 participants at each of the four clinic sites for a total sample size of 400 participants to ensure feasibility, and balanced representation across diverse outpatient settings in India. The site-level target of 100 participants facilitated operational efficiency, consistent delivery of the intervention, and evaluation of between-site heterogeneity in implementation and clinical outcomes. Eligible participants attending the outpatient clinic at the four sites were recruited prospectively. Participants provided written informed consent and were then randomly allocated to one of the study arms.

### Randomization

Randomization occurred via a computerized randomization program that was accessed through a secure web interface. The random allocation sequence was in a uniform 1:1 allocation ratio with a varied block size of 4, 6 and 8 and concealed from study personnel. Study staff enrolled/randomized eligible patients by entering data into the secure web interface.

### Intervention

A multicomponent C-QIP strategy was developed using a co-design approach engaging with multiple stakeholders (patients living with heart disease, and their caregivers, non-physician health workers [e.g., nurses, pharmacists], physicians, cardiologists, healthcare administrators, and policy makers). The co-design process involved (i) a systematic scoping review of published literature [22], (ii) qualitative interviews with patients, caregivers, providers, healthcare administrators, and policy makers [23], and (iii) a modified Delphi survey among experts practicing cardiology or related fields to generate consensus around the C-QIP strategy components [24]. The C-QIP strategy consisted of *five components*—(1) Trained and supervised non-physician health worker (named as cardiovascular care coordinator) facilitated care for patients with CVD. The cardiovascular care coordinator managed around 50–60 patients randomized to the intervention arm and assisted the physician to devise a patient-tailored CVD care plan based on patient’s CVD risk factor levels, adherence, and motivation. (2) Electronic health record—decision support system (EHR-DSS) [21] to improve physician’s responsiveness to timely treatment modification for CVD management. The DSS provided automated decision-support prompts of guideline-recommended care tailored to each patient’s CVD condition, risk level, and adherence to current medications. The EHR-DSS was co-managed by the site physician and cardiovascular care coordinator. The cardiovascular care coordinator collected and entered all patient-related data into the EHR. The site physician reviewed the computer-generated DSS prompts (CVD management plan) and made appropriate modifications to the treatment plan. (3) Text-message-based reminders tailored to patient’s lifestyle habits and to promote healthy behaviors and regular clinic visit attendance and laboratory testing, and for improving adherence to prescribed therapy. (4) Patient diary containing reinforcement tool for lifestyle modifications and visual tool for medication adherence (VITA), (5) Quarterly audit and feedback reports for providers. The audit and feedback reports were delivered by email to the participating site clinical team. Audit/feedback report included patient-level profiles and aggregate summaries of key quality indicators (use of aspirin, ≥2 blood pressure-lowering medicines, statins, and glucose-lowering medicines for patients with diabetes), as well as clinic-level averages of systolic/diastolic blood pressure, LDL cholesterol, and glycated hemoglobin (HbA1c). Site teams were instructed to use this information within a Plan–Do–Study–Act (PDSA) cycle, reviewing performance against quarterly goals, identifying gaps, and implementing corrective actions (see online S1 File for details on C-QIP strategy components and S2 Checklist).

### Control arm: Usual care

Because the trial used individual randomization within shared outpatient clinics, several procedures were used to reduce potential contamination between treatment groups. Participants randomized to the control arm received a leaflet (i.e., printed information) on healthy behaviors to prevent recurrent CVD events, delivered by a different site team member to minimize the risk of contamination and were not exposed to the electronic decision-support system, cardiovascular care coordinator follow-up, tailored SMS reminders, patient diary/VITA tools, or audit-feedback procedures. Patients in the usual care group received routine care and were only contacted once per year before their scheduled annual study visit.

### Trial procedures

The first study visit included screening, randomization, and baseline assessment including clinical measures (i.e., blood pressure, heart rate, weight, body mass index). Further, fasting blood glucose (FBG), HbA1c, and lipids were tested for all randomized participants at baseline. In addition, validated questionnaires were used for the assessment of processes of care measures including self-care, adherence to medications, diet plan and physical activity, global health assessment scale for physical and mental health assessment, patient’s treatment satisfaction, health service use, and reminders for clinic visit or lab tests [21].

Participants randomized to the intervention arm had more frequent follow-up visits per the DSS recommendations, e.g., in case of uncontrolled blood pressure (BP: ≥140/90 mmHg), or high-risk heart failure patients. Intermediate visit assessment for intervention arm participants included interval history, presenting complaints, self-care measures, and vital signs. All intermediate visits data were documented in the EHR. At annual visit and end-of-study (EOS) visit, all participants were assessed for study outcomes, including implementation outcomes, processes of care measures and clinical measures including BP, lab tests, self-care behaviors, health-related quality of life, treatment satisfaction, consultation time, reminders system for next clinic visit/lab tests, and health service utilization including outpatient and inpatient care. Because participants were recruited and randomized over a staggered period, their total post-randomization follow-up time varied. To maximize follow-up duration within the trial period, EOS visits were conducted during the trial close-out period between July and October 2024 and replaced the next scheduled annual visit, whether this would otherwise have occurred at approximately 12 or 24 months after randomization. Participants randomized to the intervention arm also had intermediate follow-up visits according to eDSS recommendations, for example in the case of uncontrolled blood pressure or high-risk heart failure; these intermediate visits were used to support intervention delivery and monitor patient status.

### Outcomes

The co-primary outcomes were feasibility and implementation domains related to delivery of the C-QIP strategy, including intervention feasibility, fidelity, adoption and acceptability assessed from patient and provider perspectives. These outcomes were selected to determine whether the intervention could be implemented and measured in routine outpatient settings, rather than to test a definitive clinical effectiveness hypothesis. Details of data sources, timing and success thresholds for implementation domains were prespecified in the published trial protocol [21]. *Feasibility* was defined as the extent to which C-QIP strategy was successfully implemented in routine outpatient workflows with available staff, time and infrastructure (e.g., enrollment of eligible patients and retention ≥80%). *Fidelity* was defined as the degree to which C-QIP components were delivered per protocol (e.g., cardiovascular care coordinator delivered counseling for adherence to GDMT, lifestyle advice, patient diary and VITA tools: ≥80%; SMS sent/delivered: ≥80%; A&F report delivered/reviewed ≤7 days with ≥1 change tested per cycle). *Adoption* was defined as the initial uptake of C-QIP DSS prompts by clinicians (DSS used in eligible visits: ≥70%; clinicians using DSS: ≥80% of enrolled patients; clinics assigning NPHW roles (100%); patients consenting to SMS/VITA tools (≥80%)). *Acceptability* was defined as perceived satisfaction, fit, and value among providers and patients (≥80% of providers and patients willing to continue using C-QIP strategy). Secondary outcomes included processes of care measures (e.g., prescription of GDMT, and patient’s adherence to prescribed medications, diet plan, physical activity, clinic visits attendance, routine monitoring of CVD risk factors, mean changes in BP, LDLc, and HbA1c, reminders for clinic visit/lab test, consultation time, and family support for self-care) from baseline to end-of-study visit. GDMT for ischemic heart disease was defined as antiplatelet + statin + Beta blocker + Angiotensin-Converting Enzyme inhibitor/Angiotensin II Receptor Blocker (ACEi/ARB). GDMT for ischemic stroke was defined as antiplatelet + statin + ACEi or ARB/Diuretic. GDMT for heart failure was defined as ACEi or ARB/ARNI + Beta blocker + Mineralocorticoid Receptor Antagonist (MRA) + Sodium–Glucose Co-Transporter 2 inhibitor (SGLT2i). All primary and secondary outcomes were assessed using baseline and EOS data to estimate preliminary effects and inform the design of a future confirmatory effectiveness-implementation trial. The present manuscript reports quantitative feasibility trial outcomes only. The costs of delivering C-QIP strategy, economic evaluation and the qualitative CFIR/RE-AIM framework-informed process evaluation addresses distinct implementation questions and will be reported separately.

### Statistical analysis

The sample size proposed for this feasibility trial was 400 patients randomized from four sites (100 patients per site). Given the primary goal of the trial was to establish that data collection protocols were feasible, fidelity of implementation, and participant adherence and retention, and to estimate the confidence intervals (CIs) for processes of care measures and clinical outcomes, a sample size of at least 70 per arm was considered adequate [25,26]. We proposed to recruit 100 participants per site (taking into account 10% drop-out rate) with a total of 400 participants to demonstrate the feasibility of C-QIP strategy implementation.

The baseline characteristics of the participants were reported by treatment group. The results for the primary implementation outcomes, i.e., feasibility, fidelity, adoption, and acceptability from provider’s and patient’s perspectives were reported as number (percentages) over the study period in the intervention arm only. For instance, the number (percentage) for the acceptance of clinical DSS prompts by the treating doctors was reported overall and stratified by sites.

The analyses for secondary outcomes such as prescription of medications (provider-level), adherence to prescribed treatment, mean changes in BP, LDLc, and HbA1c (patient-level outcomes) were conducted with the intention-to-treat (ITT) principle based on the pre-specified statistical analysis plan (SAP) (S2 File).

Because C-QIP was a feasibility randomized trial, analyses were intended primarily to estimate implementation feasibility, fidelity, adoption, acceptability, and preliminary effect sizes for process and clinical outcomes, rather than to provide confirmatory hypothesis testing.

The analysis for the prescription of medications, and adherence to self-care practices, was conducted using generalized estimating equations (GEE) using log binomial model taking into account correlation of observations within participants over time, using an exchangeable working correlation structure with robust standard errors. Site was included as a fixed effect in the model. The covariates included in the model were time (baseline and EOS), and treatment group (C-QIP strategy and usual care). The model was adjusted for the age, sex, and site. We used the Poisson link if the model did not converge using binomial link. Effect estimates (relative risk, RR or risk difference) with 95% confidence intervals (CIs) along with *p*-values were reported. To further assess the possibility of contamination, GDMT patterns were reported by treatment group and stratified by site to examine whether usual-care prescribing improved in parallel with intervention-arm prescribing at the same site, which could suggest spillover of C-QIP-related prescribing practices into usual care.

The analysis for the continuous outcomes: processes of care, health status, treatment satisfaction, clinical measure (SBP, DBP, LDLc, HbA1c, serum creatinine, weight and body mass index, BMI) was conducted using linear regression with GEE model. The covariates included in the model were time (baseline and EOS), treatment arm (C-QIP strategy and usual care), and adjusted for age, sex and site. The mean difference with 95% CI was reported for continuous outcomes. We performed the ITT analysis and did not impute the missing continuous outcomes. Model diagnostics were assessed for these outcomes by examining residual-versus-fitted plots, histograms of residuals, normal quantile–quantile (Q–Q) plots, boxplots of residuals by treatment group, and LOWESS-smoothed residual plots across study visits. These diagnostics were used to evaluate residual distributions, approximate linearity, homoscedasticity, potential group-specific patterns, and overall model fit. We also assessed the presence of influential observations and marked deviations from model assumptions. Overall, the diagnostic plots indicated adequate model fit, with residuals generally centered around zero and no evidence of model misspecification. Although mild skewness and several outlying observations were observed for certain healthcare utilization and laboratory variables (e.g., clinic visits, blood pressure checks, blood glucose testing, triglycerides, and FBG), no important violations were identified that were likely to affect the validity of the GEE analyses or the overall study results.

We compared the baseline characteristics between the final study sample and those who were lost to follow-up. We assessed the plausibility of missing-at-random assumptions by comparing baseline characteristics of participants with and without EOS data. As sensitivity analyses, we repeated key models using inverse probability weights for EOS completion. For IPTW sensitivity analyses, the probability of EOS completion was estimated using baseline demographic (age, gender, facility type, education, income) and clinical characteristics (treatment arm, systolic blood pressure, LDL-c, medication adherence) and inverse probability weights were incorporated into the key outcome models to assess the robustness of findings to missing outcome data under a missing-at-random assumption. Results from this sensitivity analysis were compared with the primary ITT analyses to evaluate the potential impact of missing outcome data on study conclusions.

Subgroup analyses were pre-specified for exploratory outcomes and conducted for self-reported medication adherence and systolic blood pressure as these measures were applicable to all patients enrolled in the study (irrespective of type of CVD condition). The prespecified subgroups were age; sex; facility type; presenting condition (ischemia heart disease, ischemic stroke, heart failure); prior history of hypertension, diabetes; baseline BMI, baseline BP; and LDLc. For each of the aforementioned subgroups, we repeated the GEE analysis with the addition of the subgroup variable along with its interaction with treatment group. Heterogeneity was assessed based on the significance of the interaction term. Serious adverse events including deaths, and hospitalizations were reported overall and by treatment group. Analyses were conducted in Stata version 18 using prespecified analysis scripts, retained with version logs, and independently checked by the study statistician; archived analysis code will be made available subject to data-sharing and ethics approvals. The SAP was finalized before database lock. Table K in S2 Appendix presents a summary of SAP-specified analyses, and reasons for any deviations. The primary analysis considered comparisons between group at EOS visit. No mixed-methods integration or triangulation of quantitative and qualitative data analysis was conducted for this manuscript.

### Ethics

The C-QIP Trial protocol was reviewed and approved by the institutional ethics committee of the Public Health Foundation of India (TRC-IEC-382.2/18) and Health Ministry Screening Committee in India (2018-0491). All participating sites also obtained their respective local site ethics committee approvals. Participation in this trial was voluntary, and a written signed informed consent was obtained from each trial participant.

## Results

### Feasibility and participant flow

A total of 492 participants were screened, of whom 410 were randomized to either the C-QIP strategy (*n* = 206) or usual care (*n* = 204). Baseline assessments were completed for 198 = C-QIP and 203 = usual care participants, with BP, LDL-C, and fasting glucose/HbA1c measurements available in over 90% of participants. At 12 months, 91/114 expected patient follow-up visits were completed in the C-QIP arm and 97/129 in usual care, corresponding to 85% visit completion. By the EOS, 192 participants in the C-QIP and 187 in usual care arm completed final study visit, yielding retention rates of 93.2% and 91.7%, respectively. The median follow-up duration was similar between arms, i.e., 15.3 months (interquartile range, [IQR] [12.8, 19.8]) in C-QIP and 15.1 months (IQR [12.6, 20.1]) in usual care arm. Dropouts and deaths were few (11 versus 1 dropouts; 9 versus 10 deaths in the C-QIP and usual care arm, respectively), confirming high trial feasibility and participant retention across both public and private sites (Fig 1).

**Fig 1:**
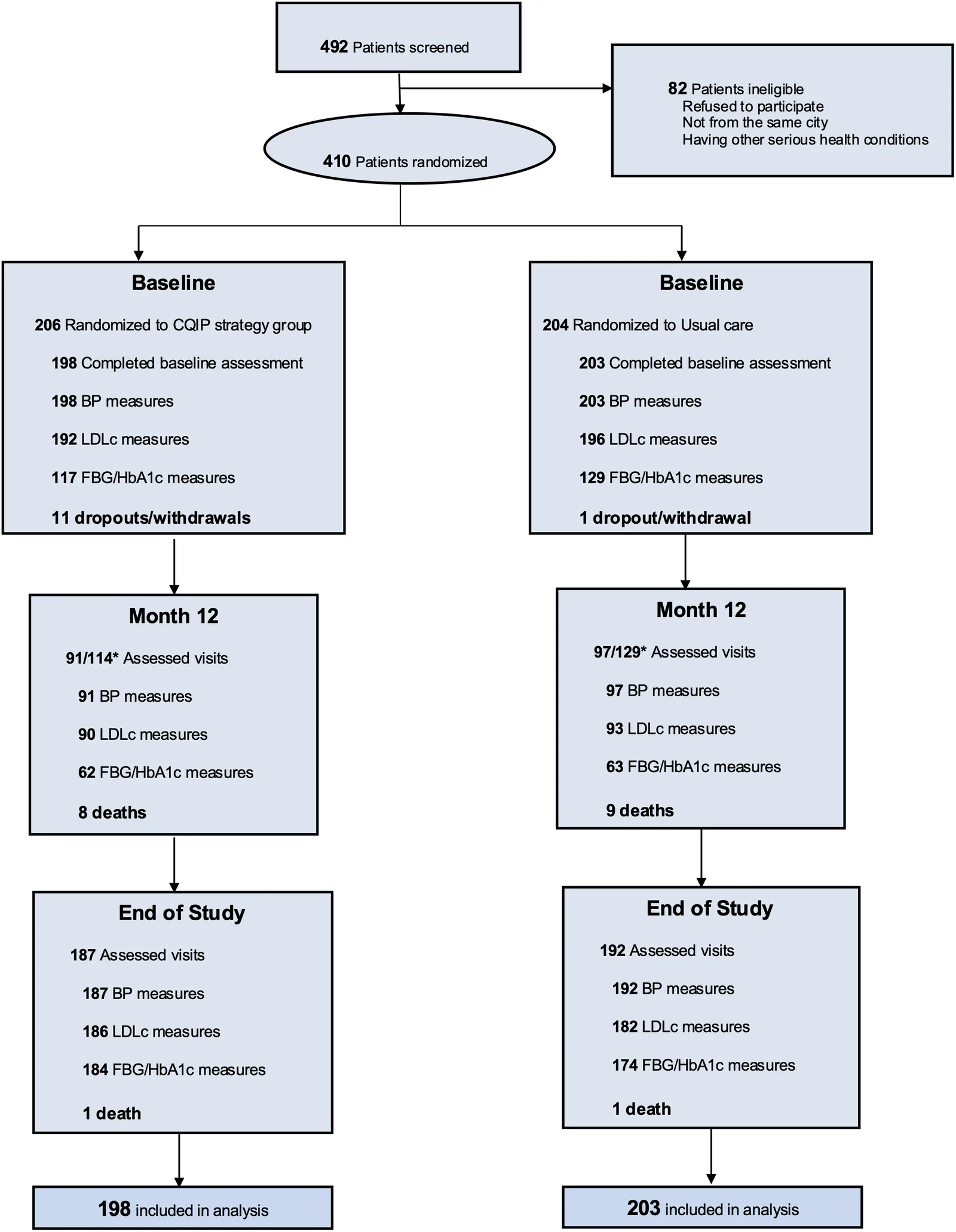
Trial Profile: Feasibility of patient recruitment, retention, and follow-ups. **Abbreviations:** CQIP, Collaborative Quality Improvement; BP, blood pressure; LDLc, low-density lipoprotein; FBG, fasting blood glucose; HbA1c, glycated hemoglobin. *Assessed visits at Month 12 are based on expected number of patients (114 vs. 129 in C-QIP and Usual care groups) due for first annual follow-up visit until June 2024. All active randomized patients were thereafter invited for EOS (end of study) visits between July and October 2024 for a final visit assessment. Median time (months) from baseline to EOS visit: C-QIP strategy—15.3 (12.8, 19.8); Usual care—15.1 (12.6, 20.1). Median time (days) from baseline to EOS visit: C-QIP strategy—459 (383, 594); Usual care—451.5 (377.5, 602). Total dropouts: 11 vs. 1; and deaths: 9 vs. 10 in the C-QIP and Usual care groups, respectively.

### Baseline characteristics by treatment group

The C-QIP strategy (*n* = 198) and usual care arm (n = 203) were well-matched at baseline. The mean age was 57.4 years (standard deviation [SD] 11.7), with similar proportions of male participants (72.7% in C-QIP versus 73.9% in usual care) and mean (SD) years of education: 10.5 (5.7) years in C-QIP versus 11.0 (5.3) in usual care arm. Prior history of CVD including coronary heart disease (76.3% versus 71.4%), heart failure (22.7% versus 18.2%), and ischemic stroke (15.2% versus 21.7%), were not significantly different between C-QIP and usual care arms, respectively. Baseline CVD risk factors were also not different between the two groups: mean SBP 126.4 ± 19.8 mmHg versus 129.2 ± 16.5 mmHg; DBP 75.4 ± 12.1 versus 77.2 ± 10.9 mmHg; LDL-C 82.1 ± 40.1 mg/dL versus 78.8 ± 34.3 mg/dL in the C-QIP versus usual care arm, respectively (Table 1).

**Table 1:** Baseline characteristics of study participants.

	C-QIP strategy	Usual care
	*N* = 198	*N* = 203
Age (years; mean, SD)	57.2 (11.2)	57.6 (12.2)
Male, *n* (%)	144 (72.7%)	150 (73.9%)
Education, (years; mean, SD)	10.5 (5.7)	11.0 (5.3)
Household Income (monthly) Indian rupees, median (IQR)	20,000 (10,000, 30,000)	20,000 (12,000, 30,000)
Medical history		
Coronary heart disease, *n* (%)	151 (76.3%)	145 (71.4%)
Heart failure, *n* (%)	45 (22.7%)	37 (18.2%)
Ischemic stroke, *n* (%)	30 (15.2%)	44 (21.7%)
Hypertension, *n* (%)	86 (43.4%)	107 (52.7%)
Diabetes, *n* (%)	82 (41.4%)	86 (42.4%)
Biomarkers		
Systolic BP (mmHg; mean, SD)	126.4 (19.8)	129.2 (16.5)
Diastolic BP (mmHg; mean, SD)	75.4 (12.1)	77.2 (10.9)
Total cholesterol (mg/dL; mean, SD)	144.0 (46.5)	145.2 (41.2)
LDLc (mg/dL; mean, SD)	82.1 (40.1)	78.8 (34.3)
FBG (mg/dL; mean, SD)	123.2 (54.7)	121.3 (55.3)
HbA1c (%; mean, SD)	6.8 (1.5)	6.7 (1.7)
Medications use		
Antiplatelet	178 (89.9%)	173 (85.2%)
Lipid lowering (Statin)	181 (91.4%)	163 (80.3%)
ACEi/ARB	123 (62.1%)	116 (57.1%)
ARNI (HF = 82)*	14 (31%)	11 (30%)
Beta-blockers	148 (74.7%)	136 (67.0%)
Calcium Channel Blockers	36 (18.2%)	35 (17.2%)
Diuretics	16 (8.1%)	14 (6.9%)

Values reported are mean (SD) or median (p25, p75) or *n* (%); p25 and p75 is the 25th and 75th percentile.

*Only reported for heart failure patients.

**Abbreviations:** LDLc, *Low-density lipoprotein cholesterol; FBG, Fasting Blood Glucose; HbA1c, glycated hemoglobin; ACEi, Angiotensin-Converting Enzyme Inhibitors; ARB, Angiotensin Receptor Blocker; ARNI, Angiotensin Receptor-Neprilysin Inhibitor; CCBs, Calcium Channel Blocker.*

### Implementation outcomes: Fidelity, adoption, and acceptability

Implementation fidelity was high. Cardiovascular care coordinators (CCCs) delivered lifestyle counseling advice to 99.5% of participants at baseline, 79.8% at 12 months, and 94.4% at EOS (Fig 2a). At the final visit, counseling was provided to 99.5% participants for healthy diet, 98.4% for exercise, 90.9% for stress avoidance, and 90.4% for home BP monitoring. Among those eligible, counseling for smoking cessation reached 60% and alcohol moderation 33% at EOS (Table A in S2 Appendix). Adoption of the eDSS was universal, i.e., 100% of follow-up visits included review of DSS prompts with variable acceptance across prompt types (antiplatelet, BP, lipid, glycemia) as illustrated in Fig 2b and Fig A in S1 Appendix. Intermediate-visit completion as per DSS recommendations between baseline and annual follow-up exceeded 90% for the first three quarterly visits and gradually declined thereafter (Table B in S2 Appendix). Both patients and providers reported high acceptability at EOS as most participants expressed satisfaction with the C-QIP strategy and willingness to continue beyond the trial duration, while providers endorsed the DSS as helpful for guideline-based prescribing (Fig 2c). Among patients, 98.4% reported C-QIP made medication adherence easier, and 97.9% wanted to continue using C-QIP beyond trial duration. Among providers, 98.4% found DSS-supported care satisfactory, and 100% found it easy to follow guidelines (Fig 2c: Acceptability of C-QIP strategy).

**Fig 2:**
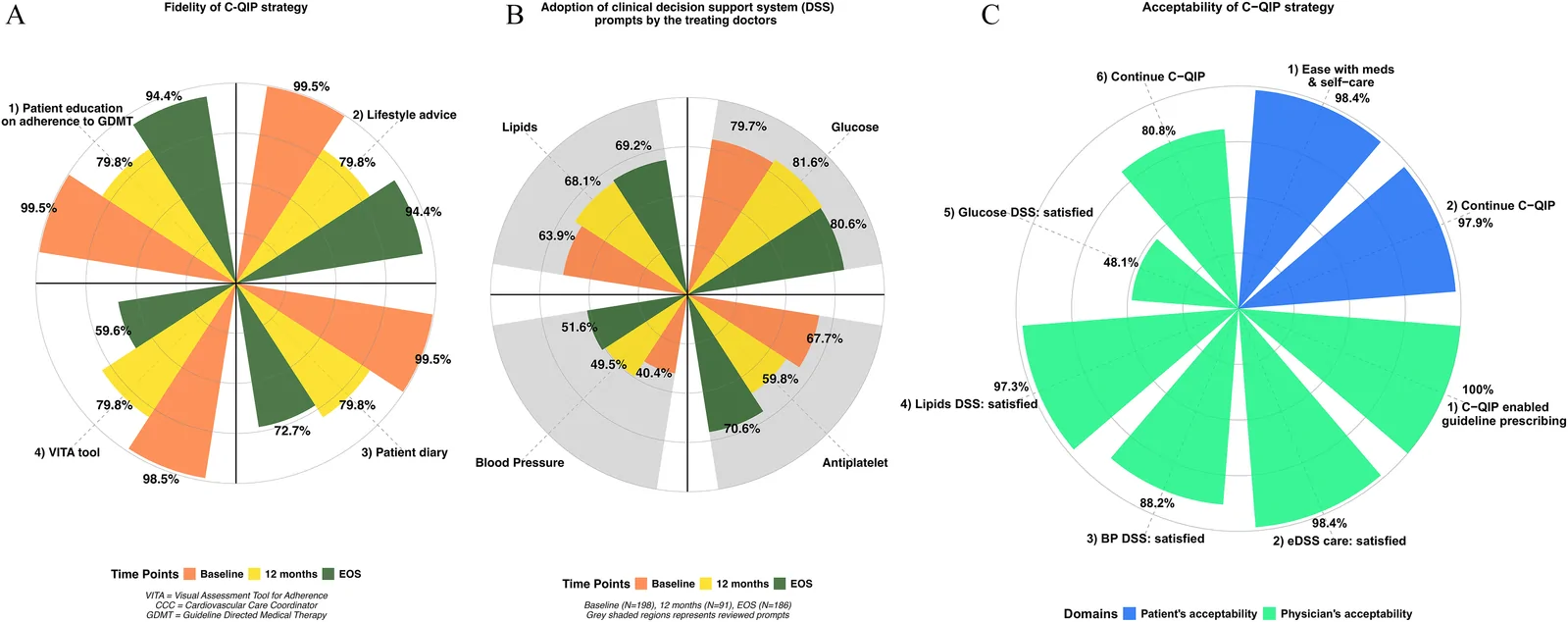
Implementation outcomes: Fidelity, Adoption, and Acceptability of C-QIP strategy. **(a)** Fidelity shows whether C-QIP strategy was **delivered** as planned: Cardiovascular Care Coordinator (CCC) delivered: (1) patient education on adherence to GDMT; (2) lifestyle advice; (3) Patient diary; (4) VITA tool. **(b)** Adoption reflects the actual use of DSS prompts, which were reviewed at each clinic visit for all C-QIP strategy arm patients (100%)—represented by gray shaded regions. Among all reviewed prompts, **acceptance** varied by DSS prompt type. DSS, Decision Support System. **(c)** Acceptability of C-QIP strategy at EOS: **Patient-level** (1) Ease with meds and self-care, (2) Continue C-QIP; **Provider-level** (1) C-QIP enabled guideline prescribing, (2) eDSS care: satisfied, (3) BP DSS: satisfied, (4) Lipids DSS: satisfied, (5) Glucose DSS: satisfied, (6) Continue C-QIP.

### Processes of care outcomes: Prescription of GDMT, and adherence to therapy

At EOS, C-QIP increased prescription of antiplatelets, statins, ACEi/ARB, β-blockers, and diuretics, without differences for CCB or ARNI; evidence-based drug bundles were higher in the C-QIP arm than in the usual care, for patients with coronary heart disease (58.3% versus 32.4%; relative risk [RR] 1.45; 95% confidence intervals [CI] [1.18, 1.78]; *p* = < 0.001) and ischemic stroke (76.7% versus 31.8%; RR 2.41; 95% CI [1.52, 3.81]; *p* = < 0.001) (Table 2). Among patients with heart failure, use of GDMT did not differ significantly between treatment groups (26.7% versus 29.7%; RR 1.27; 95% CI [0.72, 2.25]; *p* = 0.411). Patient behaviors favored C-QIP: adherence to medications (90.9% versus 82.3%; RR 1.08, 95% CI [1.04, 1.12]; *p* = < 0.001), diet (91.9% versus 82.3%; RR 1.07; 95% CI [1.02, 1.13]; *p* = 0.007), and physical activity (91.4% versus 70.4%; RR 1.23; 95% CI [1.16, 1.30]; *p* = < 0.001) was higher in the C-QIP versus usual care arm.

**Table 2:** Prescription of medications and adherence to self-care measures at end-of-study visit.

Medications use	N	C-QIP strategy	N	Usual care	Relative risk [95% CI]*	*p* value	Risk difference % [95% CI]*
**Among all patients**							
Antiplatelet	**198**	176 (88.9%)	**203**	154 (75.9%)	1.11 [1.04, 1.19]	**0.003**	9.0 [3.2, 14.8]
Lipid lowering	**198**	178 (89.9%)	**203**	148 (72.9%)	1.18 [1.10, 1.27]	**<0.001**	14.2 [8.2, 20.2]
ACEi/ARB	**198**	127 (64.1%)	**203**	100 (49.3%)	1.17 [1.01, 1.34]	**0.032**	9.2 [0.9, 17.5]
Beta-blockers	**198**	148 (74.7%)	**203**	124 (61.1%)	1.17 [1.05, 1.29]	**0.004**	11.0 [3.7, 18.3]
Calcium channel blockers	**198**	42 (21.2%)	**203**	37 (18.2%)	1.12 [0.78, 1.60]	0.545	2.2 [−4.8, 9.1]
Diuretics	**198**	26 (13.1%)	**203**	11 (5.4%)	1.82 [1.02, 3.28]	**0.045**	5.0 [0.2, 9.8]
ARNI^#^	**45**	14 (31.1%)	**37**	11 (29.7%)	1.03 [0.60, 1.77]	0.919	0.9 [−16.3, 18.1]
**Patients with coronary heart disease (*N* = 296)**							
Antiplatelet	151	141 (93.4%)	145	119 (82.1%)	1.08 [1.02, 1.14]	**0.007**	7.0 [2.0, 12.0]
Lipid lowering	151	143 (94.7%)	145	117 (80.7%)	1.14 [1.07, 1.21]	**<0.001**	11.8 [6.4, 17.1]
ACEi/ARB	151	98 (64.9%)	145	74 (51%)	1.10 [0.94, 1.29]	0.220	5.9 [−3.4, 15.3]
Beta-blockers	151	128 (84.8%)	145	99 (68.3%)	1.17 [1.06, 1.30]	**0.003**	12.5 [4.6, 20.4]
Antiplatelet + Lipid lowering drug (statin) + ACEi/ARB + Beta Blocker	151	88 (58.3%)	145	47 (32.4%)	1.45 [1.18, 1.78]	**<0.001**	17.0 [8.1, 25.9]
**Patients with ischemic stroke (*N* = 74)**							
Antiplatelet	30	27 (90%)	44	32 (72.7%)	1.14 [0.96, 1.36]	0.146	10.8 [−3.3, 25.0]
Lipid lowering	30	27 (90%)	44	28 (63.6%)	1.34 [1.08, 1.67]	**0.009**	23.4 [7.4, 39.4]
ACEi/ARB/Diuretics	30	23 (76.7%)	44	21 (47.7%)	1.52 [1.11, 2.08]	**0.009**	28.0 [7.8, 48.3]
Antiplatelet + Lipid lowering drug (statin) + ACEi/ARB/Diuretic	30	23 (76.7%)	44	14 (31.8%)	2.41 [1.52, 3.81]	**<0.001**	44.3 [24.9, 63.7]
**Patients with Heart failure (*N* = 82)**							
ACEi/ARB/ARNI	45	33 (73.3%)	37	25 (67.6%)	1.16 [0.92, 1.47]	0.209	11.0 [−6.0, 28.0]
Beta-blockers	45	35 (77.8%)	37	29 (78.4%)	1.06 [0.87, 1.28]	0.574	4.5 [−11.2, 20.2]
MRA	45	26 (57.8%)	37	25 (67.6%)	1.16 [0.92, 1.46]	0.198	9.9 [−4.9, 24.6]
SGLT2i	45	19 (42.2%)	37	16 (43.2%)	1.28 [0.85, 1.91]	0.237	11.6 [−7.2, 30.4]
ACEi/ARB/ ARNI + Beta blocker + MRA	45	22 (48.9%)	37	18 (48.6%)	1.26 [0.82, 1.94]	0.299	9.7 [−8.3, 27.7]
ACEi/ARB/ARNI + Beta blocker + MRA + SGLT2i	45	12 (26.7%)	37	11 (29.7%)	1.27 [0.72, 2.25]	0.411	6.9 [−9.5, 23.3]
**PATIENT-LEVEL MEASURES (self-reported)**							
Adherence to prescribed treatment	198	180 (90.9%)	203	167 (82.3%)	1.08 [1.04, 1.12]	**<0.001**	6.8 [3.4, 10.2]
Adherence to diet plan	198	182 (91.9%)	203	167 (82.3%)	1.07 [1.02, 1.13]	**0.007**	6.1 [1.8, 10.4]
Adherence to physical activity	198	181 (91.4%)	203	143 (70.4%)	1.23 [1.16, 1.30]	**<0.001**	17.2 [12.4, 22.0]

Adherence to prescribed treatment, diet plan and physical activity was self-reported and measured on number of days adhered in the last week and defined as ≥4 days in the past week.

*Relative Risk was estimated via generalized linear model using Poisson regression, adjusting for age, sex, site, and time. Estimates combined all non-missing values collected at baseline and end of the study. p-values for group comparisons were obtained from Wald chi-squared tests of contrasts derived from the fitted GEE model.

# Only reported for heart failure patients.

**Abbreviations:** LDLc, Low-density lipoprotein cholesterol; FBG, Fasting Blood Glucose; HbA1c, glycated hemoglobin; ACEi, Angiotensin-Converting Enzyme Inhibitors; ARB, Angiotensin Receptor Blocker; ARNI, Angiotensin Receptor-Neprilysin Inhibitor; CCBs, Calcium Channel Blocker, MRA, Mineralocorticoid Receptor Antagonists; SGLT2i, Sodium-Glucose Cotransporter-2 inhibitors.

In exploratory site-stratified analyses, usual-care GDMT use did not show consistent improvement from baseline to EOS across sites. (Table J in S2 Appendix).

Fig 3 reports data on routine monitoring of risk factors, health status, and treatment satisfaction at EOS. Participants in the C-QIP arm had more frequent clinical interactions with the care providers in the past one year: mean (SD) number of outpatient clinic visits was 7.1 (4.0) versus 2.0 (2.8) (Mean difference 2.17; 95% CI [1.56, 2.78]; *p* = < 0.001), and BP checks at clinic was 7.6 (4.3) versus 1.6 (2.9) (Mean difference 2.87; 95% CI [2.14, 3.60]; *p* = < 0.001). There was small but significant improvement in the physical health T-score in the C-QIP strategy group compared to usual care group at end of study (46.9 versus 48.6; Mean difference 0.95; 95% CI [0.04, 1.85]; *p* = 0.041). Treatment satisfaction scores were also significantly higher across all domains, e.g., mean (SD) satisfaction with current treatment: 5.0 (1.3) versus 4.5 (1.6) (Mean difference 0.51; 95% CI [0.39, 0.63]; *p* = < 0.001).

**Fig 3:**
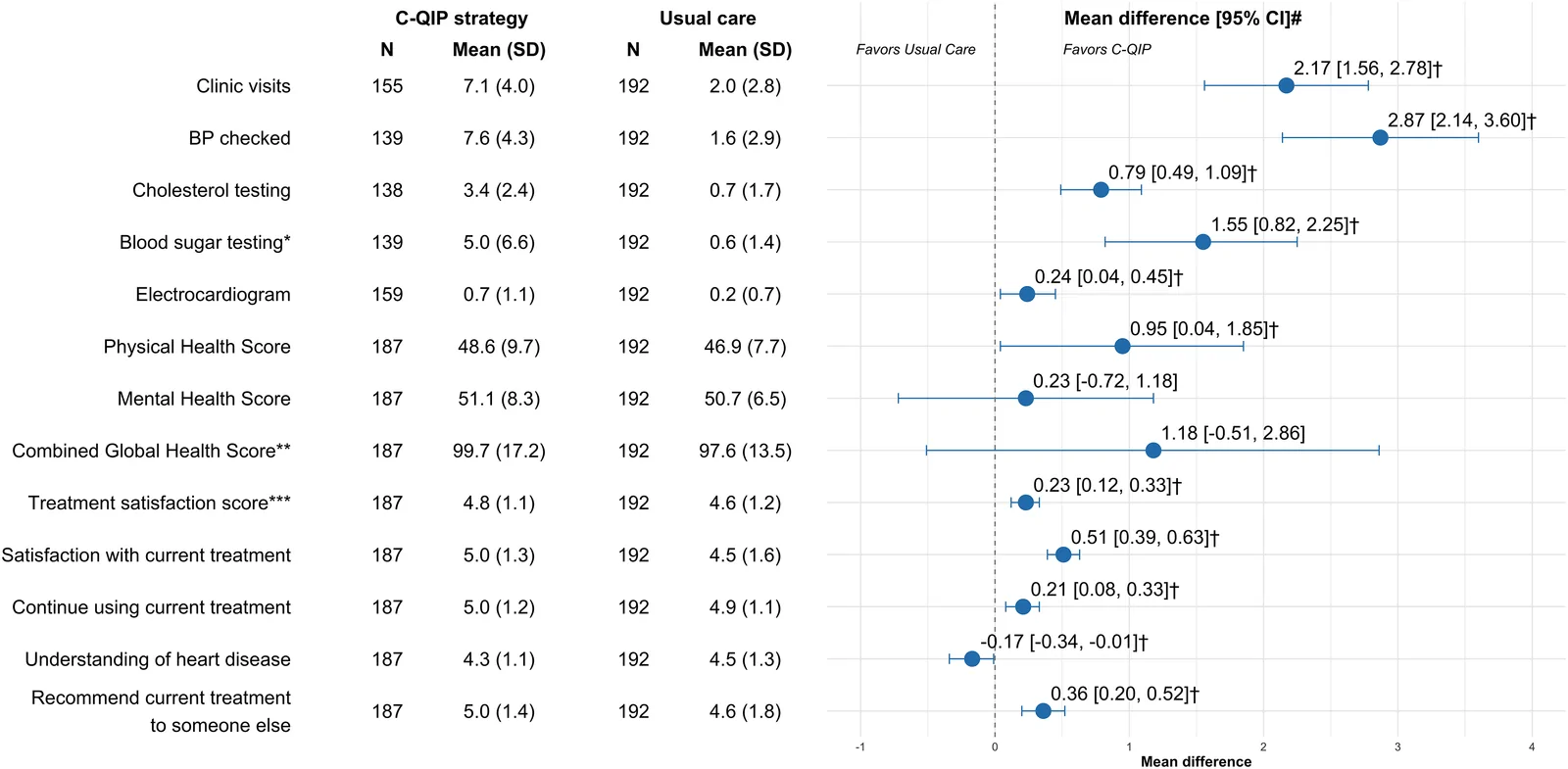
Processes of care, health status, and treatment satisfaction at EOS. ^†^*p*-value <0.05 (statistically significant values; ^#^Mean difference (95% Confidence Interval) was estimated via GEE (generalized estimating equations) model, adjusting for age, sex, site, and time. Estimates include all non-missing values collected at baseline and end of the study. ^*^Blood sugar testing included physician-recommended fasting blood sugar test. **Raw averages under each section (Physical and mental (4 items in each section with Likert scale rating of 1–5)) ranged from 4 to 20 which were converted to standardized T-Scores (range: 0–100) based on the scoring guidelines of the PROMIS Global Health instruments. ***Treatment satisfaction score was calculated by taking average of the 4 questions related to CVD treatment satisfaction including satisfaction with current treatment, continue using current treatment, understanding of health disease, recommend current treatment to someone else. EOS, end of study; BP, blood pressure.

Reminder systems were markedly enhanced in the C-QIP strategy arm versus usual care, phone calls after missed appointments 70.7% versus 4.4%, and calls prompting lab investigations 41.4% versus 0.5% (both *p* = < 0.001) (Table C in S2 Appendix). Consultation time was also longer at in the C-QIP compared to usual care group (median 10 versus 7 min, *p* = < 0.001). At trial end, family involvement in the C-QIP group was significantly higher across several self-care domains such eating healthy, exercise and quit smoking. Family support for smoking cessation was reported to be stronger in the C-QIP versus usual care arm (family joined: 7.7% versus 0%, *p* = 0.05; family reminded: 30.8% versus 2.6%, *p* = < 0.001, Table D in S2 Appendix: Family support for self-care behaviors).

### Exploratory clinical measures: Cardiovascular risk factors

At EOS, diastolic BP was significantly lower in the C-QIP group: 72.4 mmHg (SD 9.6) versus 74.3 mmHg (SD 9.6); Mean difference −1.90; 95% CI [−3.56, −0.23]; *p* = 0.03. Systolic BP showed a small but non-significant reduction in the C-QIP versus usual care group: 118.8 mmHg (SD 15.5) versus 121.0 mmHg (SD 15.3); Mean difference −2.44; 95% CI [−5.07, 0.20]; *p* = 0.07. No significant differences were observed in LDL cholesterol, HbA1c, or other risk factors (Fig 4).

**Fig 4:**
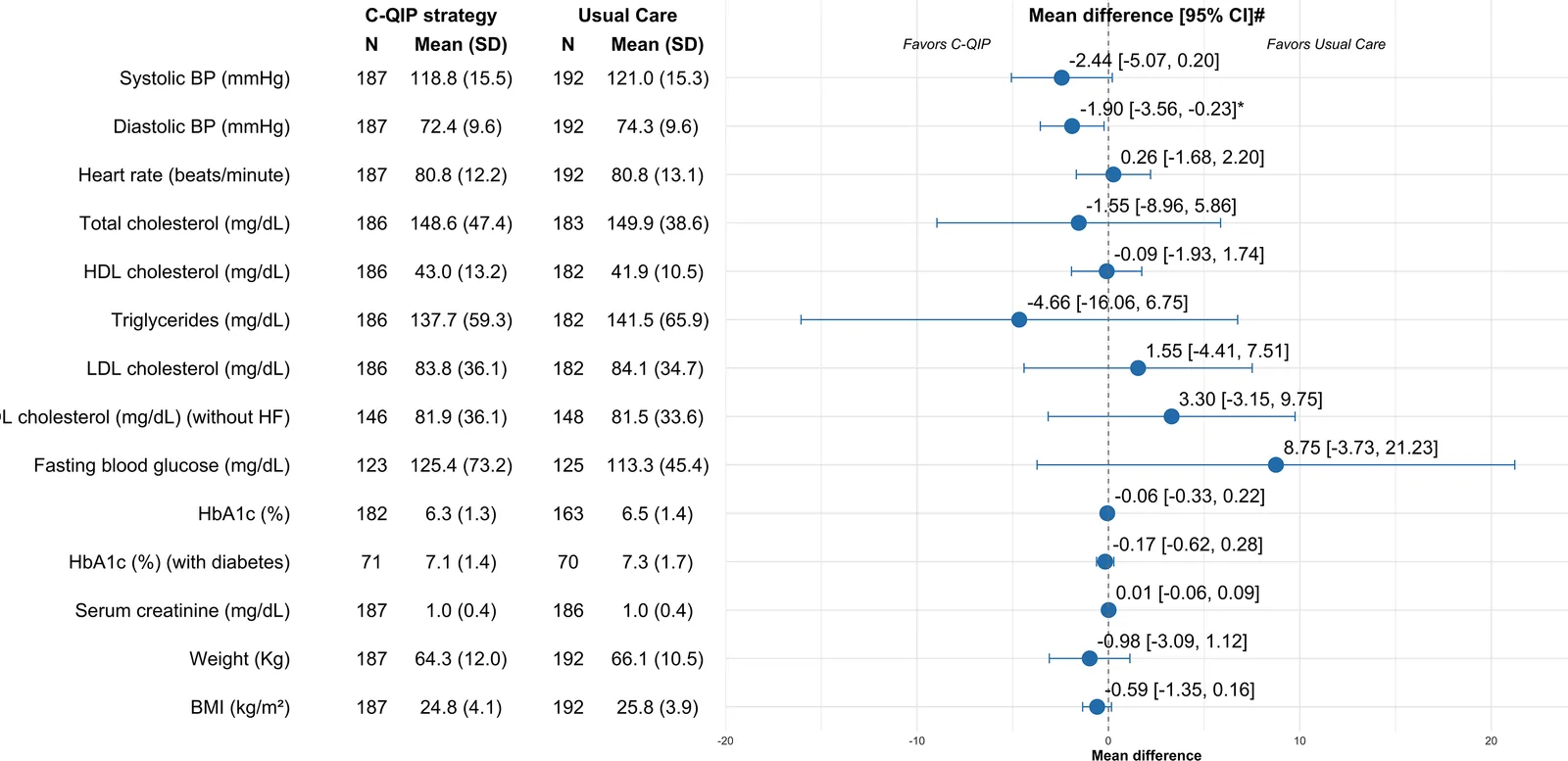
Clinical measures by treatment group at EOS. *p-value <0.05 (statistically significant values); Mean difference (95%CI) and *p*-value was estimated via GEE model, adjusting for age, sex, site, and time. Estimates include all non-missing values collected at baseline and end of the study. EOS, end of study; BP, blood pressure; BMI, body mass index; HbA1c, glycated hemoglobin; LDLc, low-density lipoprotein cholesterol; mg/dl, milligram per deciliter; CI, confidence interval.

#### Sub-group analysis.

Overall, there was limited evidence of treatment-effect heterogeneity across prespecified subgroups. However, two findings suggested possible heterogeneity and should be interpreted as hypothesis-generating. The improvement in medication adherence appeared more pronounced in private facilities than public facilities, and the effect on medication adherence was higher among patients with ischemic stroke (Fig B in S1 Appendix). For systolic blood pressure, subgroup estimates were generally directionally consistent with the overall effect, with no clear evidence of clinically meaningful heterogeneity across most strata. (Fig C in S1 Appendix)

#### Serious adverse events.

A total of 19 deaths were reported: 9 in C-QIP and 10 in usual care arm, including 11 cardiovascular deaths and 8 non-cardiovascular deaths from diverse causes. Five hospitalizations were reported during the trial. (Table E in S2 Appendix). There were no major differences in sociodemographic or clinical characteristics between participants that completed the end-of-study visit assessment and those lost to follow-up (Table F in S2 Appendix)

The IPTW sensitivity analyses for key secondary outcomes yielded estimates that were directionally consistent with the primary complete-case analyses and did not substantially change the interpretation of the main findings. In particular, the observed improvements in GDMT prescription and self-reported adherence outcomes remained consistent after weighing for the probability of EOS completion, supporting the robustness of the findings to missing outcome data related to observed baseline characteristics.

## Discussion

The C-QIP trial demonstrated that a multicomponent, quality-improvement strategy for chronic CVD care was both *feasible* and *acceptable* to patients and providers. Patient recruitment, retention, and follow-up rates were high, and fidelity to the planned intervention, including delivering patient education on adherence to therapy, lifestyle advice, patient diary and VITA tool, DSS prompts review, and structured follow-up was maintained across sites. Adoption of DSS prompts by physicians varied by prompt type, yet overall uptake and user satisfaction were encouraging, highlighting the feasibility of embedding decision-support tools into routine outpatient CVD care. Patient and provider acceptability of the C-QIP strategy was high, with most respondents expressed willingness to continue using C-QIP strategy beyond trial duration. As a result, the intervention produced measurable improvements in GDMT prescription rates and self-reported adherence to medication, diet, and physical activity. Processes of care such as timeliness of follow-up, counseling for lifestyle modification, and family engagement also improved. Exploratory analyses of CVD risk factors showed small, non-significant trends in favor of the C-QIP arm attributed to the limited power to detect between-group differences in BP, lipids, or HbA1c.

Compared with large-scale CVD implementation trials, C-QIP achieved similar or greater gains in implementation *processes and uptake measures* despite its resource-constrained setting. Few CVD secondary prevention trials reported implementation outcomes in a structured way. C-QIP prospectively measured feasibility, fidelity, adoption, and acceptability and demonstrated high delivery of coordinator-led counseling across visits; routine use and clinician acceptance of eDSS prompts (with variation by domain); and strong retention; and high patient/provider acceptability. Among Indian trials, ACS-QUIK (stepped-wedge cluster RCT) also paired processes of care gains with a mixed-methods process evaluation [16,17], documenting clinician acceptability, barriers, and site-level variation, methodologically closest to C-QIP, though focused on *acute* rather than chronic care. SPREAD [18] (post-ACS, CHW-led adherence/lifestyle support) reported high visit completion and patient engagement showing evidence of *feasibility and acceptability* for task-sharing in India, but provided fewer granular fidelity or adoption metrics. CARRS Translation Trial [27] among patients with poorly controlled type 2 diabetes, demonstrated sustained program delivery in routine diabetes clinics and strong patient engagement, but again with less domain-specific reporting of adoption than C-QIP. Compared with international trials, COORDINATE-Diabetes [28] (cluster RCT across 42 US cardiology clinics implemented a clinic-level multifaceted program: care pathways, clinician education, audit/feedback, patient tools, increased concurrent use of evidence-based drugs in AsCVD and type 2 diabetes patients) and BRIDGE [29] (cluster RCT of case-management, decision support, audit/feedback and patient/clinician education in primary care in Brazil) describe clinic-level education/audit-feedback performance and site engagement but provide limited, granular implementation metrics in the main study reports. NAILED-Stroke [30,31] (nurse-led telephone follow-up with medication titration in Sweden) and STRONG-HF [32] (of protocol-based high-intensity up-titration with close follow-up) implicitly demonstrate fidelity through protocol-based follow-up and titration, while CONNECT-HF [33] (cluster RCT of hospital-based + post-discharge quality improvement program) underscores the challenge of scaling education/audit-feedback with heterogeneous adoption across 161 hospitals. Overall, C-QIP adds unique, LMIC-based implementation granularity (including eDSS adoption) that complements efficacy signals and bridges India’s prior acute and diabetes implementation experience into chronic CVD care.

C-QIP achieved meaningful gains in the use of GDMT: higher use of antiplatelets, statins, ACEi/ARB, β-blockers, and multi-drug GDMT. It also improved self-reported adherence to medication, diet, and physical activity. These improvements in the GDMT use and medication adherence are directionally consistent with COORDINATE-Diabetes [28] and BRIDGE trials [29]. In India, ACS-QUIK improved *acute* process-of-care measures hospital-wide [16], whereas C-QIP translates that system-thinking into the *chronic* phase with sustained gains in prescribing and adherence. SPREAD Trial [18] similarly improved post-ACS adherence and lifestyle practices with community health worker support—convergent with C-QIP’s cardiovascular care coordinator-led counseling—but without the eDSS layer that likely aided prescriber behavior in C-QIP. CARRS Translation Trial [19] showed that multicomponent strategy can improve longitudinal adherence and care processes in diabetes; C-QIP extends this experience to multiple-condition CVD (ischemic heart disease, ischemic stroke, heart failure) and demonstrates parallel improvements in medication bundles and patient behaviors. In heart failure-focused implementation trials, EPIC-HF [34] (comprising of patient activation tool: 3-minute video and 1-page checklist delivered electronically before clinic consultation) and PROMPT-HF [35] (EHR-based best-practice alerts) similarly moved near-term GDMT initiation or intensification. Conversely, CONNECT-HF despite scale was neutral on its composite clinical and quality endpoints, highlighting that contextual fit and implementation strength are critical for translating toolkits into clinical practice change. In the C-QIP trial, GDMT for heart failure patients remained low at EOS. This suggests that decision support and care coordination alone may be insufficient to improve uptake of newer, higher-cost treatment for heart failure patients unless accompanied by strategies addressing medicine access, affordability, and prescribing pathways.

Compared with other data-driven cardiovascular quality-improvement trials, C-QIP provides early feasibility evidence from low-resource settings in India, whereas QUEL [36] evaluated a clinic-level quality-improvement strategy for coronary heart disease in Australian primary care using a cluster-randomized design, with an accompanying process evaluation highlighting the importance of practice engagement, audit-feedback, and local implementation capacity. QUICR similarly uses a cluster-randomized hybrid design to evaluate data-driven quality improvement in cardiac rehabilitation [37], although peer-reviewed effectiveness results are not yet available. Together, these studies support the relevance of data-driven, multicomponent quality-improvement approaches, while reinforcing the need for future cluster-randomized hybrid evaluations of C-QIP to minimize contamination and assess scalability and clinical outcomes.

C-QIP was not powered for between-group differences in BP, LDL-C, or HbA1c and showed small, non-significant trends (e.g., modest diastolic BP reduction). In contrast, trials designed around protocol-based titration to targets notably NAILED-Stroke [31] improved BP and LDL-C over 12–36 months and STRONG-HF [32] involving frequent follow-ups, GDMT up-titration with better 180-day clinical outcomes, demonstrated that dose and pace of follow-up are key to achieving CVD risk factor control. On the other hand, TEXTMEDS [38] (SMS-only) did not improve LDL-C or adherence, underscoring that single-component text-message-based reminders system is insufficient. Taken together, these comparisons suggest that C-QIP’s process of care improvements are consistent with other landmark CVD trials, and that future, large hybrid trial with longer-term follow-up is the logical next step to detect meaningful differences in CVD risk factors and events.

The trial’s findings are directly relevant to India’s NP-NCD program [14], which emphasize longitudinal, team-based chronic-care management for CVD, diabetes, and hypertension. C-QIP builds on prior Indian experiences (ACS-QUIK, CARRS Translation, and SPREAD), and extends them to sustained, people-centered chronic CVD care. Demonstrating feasibility of a coordinator-supported, decision-aided model within mixed public and private settings in India, C-QIP trial supports integration of digital clinical decision support and counseling workflows into NP-NCD clinics. Importantly, C-QIP highlights the role of patient-family engagement and routine follow-up reminders, aligning with current global calls for *people-centered, integrated chronic-care models* in LMICs. Broader adoption could strengthen guideline adherence, improve continuity, and potentially reduce premature CVD mortality if implemented effectively and sustained.

The C-QIP trial has several notable strengths. First, it is among the few randomized implementation trials in LMICs that prospectively measured and reported core implementation outcomes: feasibility, fidelity, adoption, and acceptability alongside clinical process measures. The intervention integrated a decision-support system, structured counseling by cardiovascular care coordinators, and automated reminders, thereby demonstrating that such multicomponent strategies are feasible in routine outpatient settings across diverse public and private hospitals in India. Second, the study benefited from high recruitment, retention, and follow-up rates, reflecting the feasibility and cultural acceptability of the C-QIP strategy. Third, the multicenter implementation across varying levels of health infrastructure allowed assessment of contextual adaptability, showing that cardiovascular care coordinator–led quality improvement strategies can be integrated into existing workflows without disrupting service delivery. Fourth, the use of electronic data capture and centralized monitoring minimized data loss and enabled real-time feedback. Finally, the intervention’s grounding in prior successful models such as ACS-QUIK, SPREAD, and CARRS Translation Trial strengthens and creates a bridge from acute and CVD risk reduction to the chronic-care phase of CVD management. Collectively, these features make C-QIP a pragmatic, scalable unit for improving the quality of long-term CVD care in resource-constrained environments.

Several limitations warrant consideration. First, the individual-level randomization used within clinics, while pragmatic, may have led to contamination between intervention and control arms, as providers exposed to decision-support prompts could have altered care for control patients. Providers exposed to C-QIP decision-support prompts and audit-feedback may have incorporated some learning into routine care for usual-care participants. Future confirmatory trials could consider cluster randomization at the clinic, or provider-team level to minimize contamination. Second, the trial was not blinded, and several implementation measures, including provider fidelity and patient adherence, relied partly on self-report, raising the possibility of social desirability and ascertainment bias. Third, baseline prescription rates of evidence-based medications were already high, potentially limiting the measurable margin of improvement and attenuating effect sizes. Moreover, the trial was not powered for clinical outcomes; small differences in CVD risk factors such as diastolic BP or LDL-C must therefore be interpreted cautiously, as chance findings cannot be excluded. Additionally, because several secondary outcomes, sensitivity analyses, and prespecified exploratory subgroup analyses were examined without adjustment for multiple comparisons, the possibility of chance findings cannot be excluded. The sensitivity analyses were intended to assess the robustness of the main findings rather than to generate additional confirmatory evidence; nevertheless, nominally significant secondary and subgroup findings should be interpreted cautiously. Finally, implementation was conducted under research-supported conditions with dedicated coordinators, and real-world rollout under programmatic conditions may face resource and workload challenges. Despite these limitations, the trial’s consistent direction of effect across implementation measures, processes of care, and behavioral outcomes underscores the robustness of its design and provides a strong foundation for larger confirmatory hybrid effectiveness–implementation studies.

The C-QIP trial demonstrated feasibility, fidelity, and acceptability of a multicomponent CVD care strategy across Indian outpatient settings. The intervention achieved significant improvements in guideline-directed prescribing, patient adherence, and care processes, with strong participant retention and no excess in adverse events. While CVD risk factor changes were modest, the process and implementation gains provide the empirical foundation for future large-scale, outcomes-driven hybrid effectiveness–implementation trial to reduce CVD morbidity and mortality.

## Supporting information

S1 Checklist CONSORT Checklist: 2025 Hopewell et al. This is an Open Access article distributed under the terms of the Creative Commons Attribution License (https://creativecommons.org/licenses/by/4.0/), which permits unrestricted use, distribution, and reproduction in any medium, provided the original work is properly cited. (DOCX)

S2 Checklist TiDieR checklist. (DOCX)

S1 File C-QIP Strategy Components. (DOCX)

S2 File Statistical analysis plan. (PDF)

S1 Appendix Figs A–C. (DOCX)

S2 Appendix Tables A–K. (DOCX)

S1 Protocol C-QIP Trial Protocol. (PDF)

## Abbreviations

CI: confidence intervals
CONSORT: Consolidated Standards of Reporting Trials
CVD: Cardiovascular diseases
EOS: end of study
FBG: fasting blood glucose
GDMT: guideline-directed medical therapy
GEE: generalized estimating equations
IQR: interquartile range
ITT: intention to treat
LMICs: low- and middle-income countries
MRA: Mineralocorticoid Receptor Antagonist
PDSA: Plan–Do–Study–Act
PURE: Prospective Urban Rural Epidemiology
RR: relative risk
SAP: statistical analysis plan
SD: standard deviation

## References

[1] Chong B, Jayabaskaran J, Jauhari SM, Chan SP, Goh R, Kueh MTW, et al. Global burden of cardiovascular diseases: projections from 2025 to 2050. Eur J Prev Cardiol. 2024. doi: 10.1093/eurjpc/zwae281 39270739

[2] Collaborators GBDCD. Global, regional, and national burden of cardiovascular diseases and risk factors in 204 countries and territories, 1990-2023. J Am Coll Cardiol. 2025. doi: 10.1016/j.jacc.2025.08.015 40990886

[3] Leong DP, Yusuf R, Iqbal R, Avezum Á, Yusufali A, Rosengren A, et al. The burden of cardiovascular events according to cardiovascular risk profile in adults from high-income, middle-income, and low-income countries (PURE): A cohort study. Lancet Glob Health. 2025;13(8):e1406–14. doi: 10.1016/S2214-109X(25)00155-X 40712612

[4] Walker IF, Garbe F, Wright J, Newell I, Athiraman N, Khan N, et al. The economic costs of cardiovascular disease, diabetes mellitus, and associated complications in South Asia: A systematic review. Value Health Reg Issues. 2018;15:12–26. doi: 10.1016/j.vhri.2017.05.003 29474174

[5] Wood DA. WHF roadmap on secondary prevention of CVD. Glob Heart. 2024;19(1):9. doi: 10.5334/gh.1294 38273997 PMC10809851

[6] Tabak RG, Kandula NR, Angell SY, Brewer LC, Grandner M, Hayman LL, et al. Implementation of evidence-based behavioral interventions for cardiovascular disease prevention in community settings: A scientific statement from the American Heart Association. Circulation. 2025. doi: 10.1161/CIR.0000000000001349 40755303

[7] Yoo SGK, Chung GS, Bahendeka SK, Sibai AM, Damasceno A, Farzadfar F, et al. Aspirin for secondary prevention of cardiovascular disease in 51 low-, middle-, and high-income countries. JAMA. 2023;330(8):715–24. doi: 10.1001/jama.2023.12905 37606674 PMC10445202

[8] Yusuf S, Islam S, Chow CK, Rangarajan S, Dagenais G, Diaz R, et al. Use of secondary prevention drugs for cardiovascular disease in the community in high-income, middle-income, and low-income countries (the PURE Study): A prospective epidemiological survey. Lancet. 2011;378(9798):1231–43. doi: 10.1016/S0140-6736(11)61215-4 21872920

[9] Joseph P, Avezum A, Ramasundarahettige C, Mony PK, Yusuf R, Kazmi K, et al. Secondary prevention medications in 17 countries grouped by income level (PURE): A prospective cohort study. J Am Coll Cardiol. 2025;85(5):436–7. doi: 10.1016/j.jacc.2024.10.121 39909677

[10] Mulure N, Hewadmal H, Khan Z. Assessing barriers to primary prevention of cardiovascular diseases in low and middle-income countries: A systematic review. Cureus. 2024;16(7):e65516. doi: 10.7759/cureus.65516 39188440 PMC11346380

[11] Agyemang C, van den Born B-J. Limited access to CVD medicines in low-income and middle-income countries: poverty is at the heart of the matter. Lancet Glob Health. 2018;6(3):e234–5. doi: 10.1016/S2214-109X(18)30048-2 29433655

[12] Mendis S, Graham I. Prevention and control of cardiovascular disease in “real-world” settings: sustainable implementation of effective policies. Front Cardiovasc Med. 2024;11:1380809. doi: 10.3389/fcvm.2024.1380809 39628553 PMC11611850

[13] Schwalm JD, McKee M, Huffman MD, Yusuf S. Resource effective strategies to prevent and treat cardiovascular disease. Circulation. 2016;133(8):742–55. doi: 10.1161/CIRCULATIONAHA.115.008721 26903017 PMC4766731

[14] National programme for prevention and control of NCDs (NP-NCD). Ministry of Health and Family Welfare, Government of India 2023 [cited 2025 Nov 10]. Available from: https://ncd.nhp.gov.in/ncdlandingassets/aboutus.html

[15] Meghana RVNSP, Singh J, Huda RK. Challenges, barriers, and facilitators in implementing digital health initiatives for NP-NCD management in India’s public health system: A scoping review. Clin Epidemiol Global Health. 2025;35:102137. doi: 10.1016/j.cegh.2025.102137

[16] Huffman MD, Mohanan PP, Devarajan R, Baldridge AS, Kondal D, Zhao L, et al. Effect of a quality improvement intervention on clinical outcomes in patients in india with acute myocardial infarction: The ACS QUIK randomized clinical trial. JAMA. 2018;319(6):567–78. doi: 10.1001/jama.2017.21906 29450524 PMC5838631

[17] Singh K, Devarajan R, Mohanan PP, Baldridge AS, Kondal D, Victorson DE, et al. Implementation and acceptability of a heart attack quality improvement intervention in India: A mixed methods analysis of the ACS QUIK trial. Implement Sci. 2019;14(1):12. doi: 10.1186/s13012-019-0857-7 30728053 PMC6364470

[18] Xavier D, Gupta R, Kamath D, Sigamani A, Devereaux PJ, George N, et al. Community health worker-based intervention for adherence to drugs and lifestyle change after acute coronary syndrome: a multicentre, open, randomised controlled trial. Lancet Diabetes Endocrinol. 2016;4(3):244–53. doi: 10.1016/S2213-8587(15)00480-5 26857999

[19] Ali MK, Singh K, Kondal D, Devarajan R, Patel SA, Menon VU, et al. Effect of a multicomponent quality improvement strategy on sustained achievement of diabetes care goals and macrovascular and microvascular complications in South Asia at 6.5 years follow-up: Post hoc analyses of the CARRS randomized clinical trial. PLoS Med. 2024;21(6):e1004335. doi: 10.1371/journal.pmed.1004335 38829880 PMC11198027

[20] Singh K, Johnson L, Devarajan R, Shivashankar R, Sharma P, Kondal D, et al. Acceptability of a decision-support electronic health record system and its impact on diabetes care goals in South Asia: A mixed-methods evaluation of the CARRS trial. Diabet Med. 2018;35(12):1644–54. doi: 10.1111/dme.13804 30142228

[21] Singh K, Nikhare K, Gandral M, Aithal K, Patil SG, Mp G, et al. Rationale, design and baseline characteristics of a randomized controlled trial of a cardiovascular quality improvement strategy in India: The C-QIP trial. Am Heart J. 2024;276:83–98. doi: 10.1016/j.ahj.2024.07.008 39033994 PMC12423933

[22] Singh K, Bawa VS, Venkateshmurthy NS, Gandral M, Sharma S, Lodhi S, et al. Assessment of studies of quality improvement strategies to enhance outcomes in patients with cardiovascular disease. JAMA Netw Open. 2021;4(6):e2113375. doi: 10.1001/jamanetworkopen.2021.13375 34125220 PMC8204210

[23] Singh K, Huffman MD, Johnson LCM, Tandon N, Prabhakaran D, Mendenhall E. Collaborative quality improvement strategy in secondary prevention of cardiovascular disease in india: findings from a multi-stakeholder, qualitative study using consolidated framework for implementation research (CFIR). Glob Heart. 2022;17(1):72. doi: 10.5334/gh.1161 36382156 PMC9562780

[24] Singh K, Joshi A, Venkateshmurthy NS, Rahul R, Huffman MD, Tandon N, et al. A Delphi study to prioritize evidence-based strategies for cardiovascular disease care in India. Glob Implement Res Appl. 2023;:1–12. doi: 10.1007/s43477-023-00087-2 37363377 PMC10240122

[25] Billingham SAM, Whitehead AL, Julious SA. An audit of sample sizes for pilot and feasibility trials being undertaken in the United Kingdom registered in the United Kingdom Clinical Research Network database. BMC Med Res Methodol. 2013;13:104. doi: 10.1186/1471-2288-13-104 23961782 PMC3765378

[26] Totton N, Lin J, Julious S, Chowdhury M, Brand A. A review of sample sizes for UK pilot and feasibility studies on the ISRCTN registry from 2013 to 2020. Pilot Feasibility Stud. 2023;9(1):188. doi: 10.1186/s40814-023-01416-w 37990337 PMC10662929

[27] Johnson LCM, Nikhare K, Jaganathan S, Ali MK, Narayan KMV, Prabhakaran D, et al. Stakeholder perspectives regarding the acceptability and sustainability of a multi-component diabetes care strategy in South Asia: A longitudinal qualitative analysis. Glob Implement Res Appl. 2022;2(4):350–60. doi: 10.1007/s43477-022-00060-5 37745272 PMC10516368

[28] Pagidipati NJ, Nelson AJ, Kaltenbach LA, Leyva M, McGuire DK, Pop-Busui R, et al. Coordinated care to optimize cardiovascular preventive therapies in type 2 diabetes: A randomized clinical trial. JAMA. 2023;329(15):1261–70. doi: 10.1001/jama.2023.2854 36877177 PMC9989955

[29] Machline-Carrion MJ, Soares RM, Damiani LP, Campos VB, Sampaio B, Fonseca FH, et al. Effect of a multifaceted quality improvement intervention on the prescription of evidence-based treatment in patients at high cardiovascular risk in Brazil: The BRIDGE cardiovascular prevention cluster randomized clinical trial. JAMA Cardiol. 2019;4(5):408–17. doi: 10.1001/jamacardio.2019.0649 30942842 PMC6537802

[30] Irewall A-L, Ögren J, Bergström L, Laurell K, Söderström L, Mooe T. Nurse-led, telephone-based, secondary preventive follow-up after stroke or transient ischemic attack improves blood pressure and LDL cholesterol: Results from the first 12 months of the randomized, controlled NAILED stroke risk factor trial. PLoS One. 2015;10(10):e0139997. doi: 10.1371/journal.pone.0139997 26474055 PMC4608694

[31] Ögren J, Irewall A-L, Söderström L, Mooe T. Long-term, telephone-based follow-up after stroke and TIA improves risk factors: 36-month results from the randomized controlled NAILED stroke risk factor trial. BMC Neurol. 2018;18(1):153. doi: 10.1186/s12883-018-1158-5 30241499 PMC6148791

[32] Mebazaa A, Davison B, Chioncel O, Cohen-Solal A, Diaz R, Filippatos G, et al. Safety, tolerability and efficacy of up-titration of guideline-directed medical therapies for acute heart failure (STRONG-HF): A multinational, open-label, randomised, trial. Lancet. 2022;400(10367):1938–52. doi: 10.1016/S0140-6736(22)02076-1 36356631

[33] DeVore AD, Granger BB, Fonarow GC, Al-Khalidi HR, Albert NM, Lewis EF, et al. Effect of a hospital and postdischarge quality improvement intervention on clinical outcomes and quality of care for patients with heart failure with reduced ejection fraction: The CONNECT-HF randomized clinical trial. JAMA. 2021;326(4):314–23. doi: 10.1001/jama.2021.8844 34313687 PMC8317015

[34] Allen LA, Venechuk G, McIlvennan CK, Page RL 2nd, Knoepke CE, Helmkamp LJ, et al. An electronically delivered patient-activation tool for intensification of medications for chronic heart failure with reduced ejection fraction: The EPIC-HF trial. Circulation. 2021;143(5):427–37. doi: 10.1161/CIRCULATIONAHA.120.051863 33201741 PMC7855616

[35] Ghazi L, Yamamoto Y, Riello RJ, Coronel-Moreno C, Martin M, O’Connor KD, et al. Electronic alerts to improve heart failure therapy in outpatient practice: A cluster randomized trial. J Am Coll Cardiol. 2022;79(22):2203–13. doi: 10.1016/j.jacc.2022.03.338 35385798

[36] Hafiz N, Hyun K, Tu Q, Knight A, Hespe C, Chow CK, et al. Process evaluation of a data-driven quality improvement program within a cluster randomised controlled trial to improve coronary heart disease management in Australian primary care. PLoS One. 2024;19(6):e0298777. doi: 10.1371/journal.pone.0298777 38833486 PMC11149853

[37] Candelaria D, Redfern J, O’Neil A, Brieger D, Clark RA, Briffa T, et al. Data-driven collaborative QUality improvement in Cardiac Rehabilitation (QUICR) to increase program completion: Protocol for a cluster randomized controlled trial. BMC Cardiovasc Disord. 2024;24(1):302. doi: 10.1186/s12872-024-03971-3 38877422 PMC11177531

[38] Chow CK, Klimis H, Thiagalingam A, Redfern J, Hillis GS, Brieger D, et al. Text messages to improve medication adherence and secondary prevention after acute coronary syndrome: The TEXTMEDS randomized clinical trial. Circulation. 2022;145(19):1443–55. doi: 10.1161/CIRCULATIONAHA.121.056161 35533220

