# Supplementary material for: Assessing a multicomponent quality improvement strategy for chronic care of cardiovascular diseases in India: The C-QIP randomized feasibility trial
Source: PLoS Med. 2026 Sep 29;23(9):e1004932. doi: 10.1371/journal.pmed.1004932 (PMC13641716; doi:10.1371/journal.pmed.1004932)
Supplement: Supplementary file 1 [file pmed.1004932.s004.pdf]

STATISTICAL ANALYSIS PLAN

Final Version 1.0 date 30 October 2024

Developing and testing a Collaborative Quality ImProvement initiative for prevention of cardiovascular disease in India (C-QIP)

Protocol: Version 1.2: 07 February 2022

Prepared for:

C-QIP Trial

Version: 1.0

**Prepared by:**	**Signature**	**Date**
Dr Dimple Kondal<br><br>Dr Kavita Singh		**22.10.2024**
**Reviewed and approved by:**		
Dr Mark Huffman		**30.10.2024**
Dr Dorairaj Prabhakaran		**30.10.2024**

Contents

Introduction ... 4
Study Objectives ... 4
Implementation Feasibility and Fidelity ... 4
Primary Objective ... 4
Secondary Objective ... 5
Safety ... 5
Study Design ... 5
Intermediate ... 6
12-month Follow-up ... 6
End of study ... 6
Sample Size ... 6
Study Outcomes/Endpoints ... 7
Statistical Analysis ... 8
General Principles ... 8
Baseline analysis ... 9
CONSORT Statement ... 9
Primary endpoint analyses ... 9
Primary analyses: Implementation outcomes ... 9
Secondary and Exploratory endpoint analyses ... 11
Secondary analyses: Prescription of GDMT and Adherence to CVD medications ... 11
Exploratory analyses: Mean change in systolic blood pressure and LDL-cholesterol 11
Definition of end of follow-up ... 11
Adjusted Analyses- For Secondary and Exploratory outcomes. ... 12
Sub-group analysis ... 12
Missing data handling ... 13
Safety Analysis ... 13
Interim Analysis ... 13

Independent Data Safety Monitoring Board (DSMB) 13
Appendix: Templates for Tables, Data Listings and Figures 14

Introduction

This Statistical analysis plan (SAP) describes the planned analysis and reporting for the NIH funded study title "Developing and testing a Collaborative Quality ImProvement (C-QIP) initiative for prevention of cardiovascular disease in India.

The structure and content of this SAP provides sufficient detail to meet the requirements identified by the FDA and International Conference on Harmonization of Technical Requirements for Registration of Pharmaceuticals for Human Use (ICH): Guidance on Statistical Principles in Clinical Trials. All work planned and reported for this SAP will follow internationally accepted guidelines, published by the American Statistical Association and the Royal Statistical Society, for statistical practice.

The following documents were reviewed in preparation of this SAP:

- Clinical Research Protocol version 1.2 7th February 2022
- Case report forms (CRFs) version 1.2 7th February 2022.
- ICH Guidance on Statistical Principles for Clinical Trials.

The reader of this SAP is encouraged to also read the clinical protocols for details on the conduct of this study, and the operational aspects of clinical assessments and timing for completing a patient in this study.

The analysis plan also outlines the proposed layout of tables and figures that will be presented.

Study Objectives

Implementation Feasibility and Fidelity

Primary Objective

To test the **feasibility of collaborative quality improvement strategy**, compared to the usual care, among patients with cardiovascular disease (CVD) in India.

The main primary outcome of interest is the **implementation outcomes** defined by **acceptability** of intervention strategy (extent to which providers and patients perceive C-QIP strategy to be agreeable or satisfactory), **adoption** (i.e., uptake or utilization of C-QIP strategy by providers, non-physician health workers, and patients at the participating sites), **feasibility** (extent to which C-QIP can be successfully implemented and used by the participating sites), and **fidelity** (degree to which the C-QIP strategy was delivered as per the protocol i.e., dose delivered vs. dose received), **appropriateness** (i.e., perceived fit or relevance of the C-QIP strategy from the perspectives of providers and patients), and **cost** of implementing the C-QIP strategy.

Secondary Objective

The secondary outcomes are:

<u>Processes of care outcomes: Between group differences in:</u>

1. Prescription rate of guideline directed medical therapy (GDMT) by providers.
2. Patient's adherence rate to GDMT.

Exploratory clinical outcomes: Between group differences in:

1. Mean changes in systolic blood pressure at trial end.
2. Mean changes in LDL cholesterol at trial end.
3. Mean change in quality of life or health status at trial end.
4. Incremental cost of care at trial end.
5. Adherence to recommended lifestyle habits: smoking cessation, diet, physical and activity at trial end.

Safety

The CVD risk reduction delivery strategies of the intervention are not implementing any new drug or invasive procedure that requires specific monitoring of safety parameters. Rather, the intervention is a preventative health study that is enforcing existing, evidence-based guidelines for CVD risk reduction.

Serious adverse events (SAE) will be assessed between both groups.

Study Design

The study is a multi-site, individually randomized, controlled parallel group hybrid type1 implementation study testing a comprehensive, multicomponent collaborative quality improvement strategy (C-QIP) in 400 patients with CVD attending four out-patient clinics in India, for a mean follow-up of 12 months.

A total of 400 patients with established CVD (post myocardial infarction, stroke, or heart failure regardless of ejection fraction) will be enrolled in the study; 100 at each of the 4 out-patient clinics in India. The 100 eligible, consenting participants at each site will be individually randomized to usual care (n=50) OR to the intervention (n=50) which targets processes of care measures (use of evidence-based medications; smoking cessation advice; lifestyle modification advice), adherence to prescribed therapy, and blood pressure and lipids control. Table 1. Below shows the study measures and schedule of evaluations in the C-QIP study.

Table 1. Schedule of evaluations in the C-QIP study.

Evaluations/Visits	Screening	Baseline	Intermediate	12-month Follow-up	End of study
**Signed informed consent**	x				
**Assess Eligibility**	x				
**Enrolment/Randomization**	x				
**Demographics**		x			
**Medical history**		x			
**Family history**		x			
**Social History (lifestyle/habits)**		x	x	x	x
**Self-care questions**		x		x	x
**Quality of Life, short (Global Health Scale))**		x		x	x
**Treatment Satisfaction questions**		x		x	x
**Processes of care questions**		x		x	x
**Costs of care questions**		x		x	x
**Acceptability: providers and patient interviews**		Sub-sample		Sub-sample	Sub-sample
**Blood pressure and heart rate**		x	x	x	x
**Height**		x		x	x
**Weight, BMI (calculated), Waist Circumference**		x		x	x
**General Physical Examination**		x		x	x
**Serum Creatinine**		x		x	x
**Serum Sodium, Potassium and Liver enzymes**		x		x	x
**Fasting blood glucose**		x	x	x	x
**Glycated hemoglobin A1c (HbA1c)**		x	x	x	x
**Lipids (TC, HDL, LDL, TG)**		x	x	x	x
**Medications: antiplatelets, blood pressure lowering, and cholesterol lowering medications**		x	x	x	x
**Serious adverse event reporting**		x	x	x	x

BMI=body mass index; TC=total cholesterol, HDL=high density lipoprotein cholesterol, LDL=low density lipoprotein cholesterol, TG=triglycerides

Sample Size

The proposed study is a feasibility, individual randomized controlled trial, with a primary focus being on the development of locally adaptable and culturally sensitivity C-QIP intervention and to understand the feasibility, acceptability, and preliminary effects of the intervention on process measures and clinical outcomes. Therefore, we will select 4 CVD clinics (2 government and 2 private, 100 patients each), and a total of 400 patients (stratified by age, and sex) to conduct a feasibility RCT to assess the impact of the C-QIP strategy by examining implementation outcomes, processes of care measures (prescription rates and adherence rates), health-related quality of life, cost, and clinical outcomes. The results of this feasibility RCT, will inform sample size estimates and power calculation for a future outcomes-driven, larger randomized trial to study the effectiveness, cost-effectiveness, and scalability of C-QIP intervention for secondary prevention of CVD.

Study Outcomes/Endpoints

Primary outcomes: Implementation outcomes

1. *Feasibility*: Extent to which C-QIP strategy can be successfully used or carried out at the four participating sites (i.e., dose delivered and dose received). Number of clinical decision support prompts reviewed, accepted, or rejected to make changes in the treatment plan, no. of clinic visits, no, of lab tests performed in the intervention group patients, number of interactions between the patient and non-physician health workers, number of text-messages sent to the patients.
2. *Fidelity*: Degree to which C-QIP strategy was delivered as prescribed in the study protocol or as intended by the study investigators. Fidelity will be determined by objectively measuring adherence to treatment protocol using questionnaire and qualitative interviews with providers, and patients to explore the dose received and dose delivered, i.e., use of clinical decision support prompts by the providers, text-messages sent/received/actioned by the patients.
3. *Adoption*: Uptake, utilization, initial implementation, or intention to try using multicomponent C-QIP strategy by providers, non-physician health worker (NPHW) or cardiovascular care coordinator (CCC), and patients at participating sites.
4. *Acceptability*: Extent to which providers and patients perceive C-QIP strategy to be agreeable, or satisfactory.
5. *Cost*: Financial impact of implementing C-QIP strategy including marginal cost of treatment delivery in the intervention versus usual care group, cost of the implementation strategy including the non-physician health workers, text-messages, patient-diary, electronic health records and decision-support system and audit and feedback reports.
6. *Appropriateness*: Perceived fit, relevance, or compatibility of the C-QIP at the four participating sites (2 government and 2 private hospitals), as well as the usefulness and practicability of C-QIP strategy from the perspectives of the care providers, and patients for chronic care of CVD.

Secondary outcomes: Processes of care outcomes: Between group differences in:

1. Prescription rate of guideline directed medical therapy (GDMT) by providers in intervention vs usual care group.
2. Patient’s adherence to prescribed therapy in intervention vs usual care group.

Exploratory clinical outcomes: Between group differences in:

1. Mean changes in systolic blood pressure at trial end.
2. Mean changes in LDL cholesterol at trial end.
3. Mean change in health-related quality of life.
4. Incremental cost of care

5. Adherence to recommended lifestyle habits: smoking cessation, diet, physical activity, and mental health.

Adverse events

Definition of an Adverse Event: "An adverse event (AE) is any untoward medical occurrence in a subject (study participant) temporally associated with participation in the clinical study or with use of the experimental agent being studied. An adverse finding can include a sign, symptom, abnormal assessment (laboratory test value, vital signs, electrocardiogram finding, etc.), or any combination of these."

Definition of a Serious Adverse Event: "A serious adverse event (SAE) is any adverse event (any untoward medical occurrence in a subject/study participant temporally associated with participation in the clinical study or with use of the experimental agent being studied) that results in one or more of the following outcomes: death; a life threatening event; inpatient hospitalization or prolongation of existing hospitalization; a persistent or significant disability/incapacity; a congenital anomaly or birth defect; or an important medical event based upon appropriate medical judgment."

List of Serious Adverse Events which will be reported in the study are:

1. CVD events: Angina, non-fatal myocardial infarction (MI) / Unstable Angina, revascularization procedure [angioplasty or coronary artery bypass surgery (CABG)], Transient Ischemic Attack (TIA), Stroke (non-fatal), Arrhythmia
2. Renal: end-stage renal disease requiring renal replacement therapy (dialysis or transplantation)
3. Death
4. Any other major health conditions or hospitalization for more than 24 hours (important medical event based upon appropriate medical judgment)

Statistical Analysis

General Principles

Data analyses will be performed by the research team at Public Health Foundation of India (PHFI) with senior statistical oversight provided by Dr Dimple Kondal based at the Centre for Chronic Disease Control, New Delhi. This Statistical analysis plan will be finalized prior to the database lock.

All statistical tests will be two-sided with a significance level of α=0.05, unless specified otherwise, and will be performed using STATA Version 18.0. All treatment evaluations will be performed on the principle of 'intention to treat' unless otherwise specified.

Data will be summarized using descriptive statistics (number of subjects [n], mean, standard deviation [SD], median, minimum, and maximum) for continuous variables, and using frequency and percentage (i.e., number and proportion of subjects [n, %]) for discrete/categorical variables, unless specified otherwise.

Baseline analysis

We will report the descriptive statistics by intervention and usual care groups and by visit. The baseline difference between the groups for the continuous variables will be assessed using t-test or Wilcoxon-rank sum test and for the categorical variables chi-square test will be used. The p-value<0.05 will be considered statistically significant.

CONSORT Statement

All participants who were invited to participate in this trial should be accounted for and a CONSORT statement prepared. Reasons for early withdrawal will be listed for all participants that prematurely discontinued the study. The number of participants that were registered but not randomized will be presented and the reasons for non-participation (where available).

Primary endpoint analyses

Primary analyses: Implementation outcomes

- Feasibility: number of patients screened, number of patients found eligible, time needed to recruit eligible patients, number of patients randomized to intervention and control group, number of patients who completed scheduled study visits and follow-up rates, number of consent withdrawals, lost to follow-up/drop-out rates, time needed to collect and analyze data will be reported overall and by treatment group.
- Fidelity (adherence to study protocol),
- Adoption (use of EHR-DSS management plan for the intervention arm patients, number of DSS prompts accepted by the physicians, use of text-messages as reminders for next clinic visit, lab appointment and health lifestyle, number of text-messages sent to the patients), and
- Acceptability of C-QIP strategy will be assessed using treatment satisfaction questionnaire and qualitative interviews with providers, non-physician health workers, health administrators, patients, and caregivers.
- Costs: intervention set up and delivery costs
- Appropriateness: perceived fit or relevance of C-QIP strategy from the providers and patients' perspectives

All analyses relevant for primary endpoint, i.e., implementation outcomes will focus on intervention group participants. Data triangulation using both quantitative trial data collected at baseline and follow-up visits along with

qualitative interview data will be carried out to have robust and comprehensive results to guide future implementation or scale-up of C-QIP strategy.

Table 2. Summary of implementation outcomes, indicators, and timepoint for analysis.

Outcome (Definition)	Indicator(s)	Timepoint
**Feasibility** (extent to which C-QIP strategy was successfully implemented)	▪ No. of patients screened/enrolled.<br>▪ No. of patients randomized.<br>▪ No. of patient attending follow-up visits.<br>▪ No. of interactions between NPHW and patients<br>▪ Time to taken to analyze and send quarterly audit reports.<br>▪ Time taken to review audit reports.	• Baseline<br>• Baseline<br>• Follow-up visits<br>• Follow-up visits<br>• Follow-up visits<br>• Follow-up visits
**Fidelity** (degree to which intervention was delivered as planned)	▪ Intervention dose delivered vs dose received for all 5 components: i) EHRDSS, ii) NPHW facilitated care, iii) text-messages, iv) patient diary + VITA tool, v) audit and feedback report.<br>▪ No. of DSS prompts accepted/rejected.<br>▪ No. of text-messages sent to patients.	Baseline and follow-up visits
**Adoption** (uptake, utilization of C-QIP strategy)	▪ Use of EHR for data collection<br>▪ Use of DSS prompts.<br>▪ Use of patient diary<br>▪ Use of VITA tool<br>▪ Use of text-messages<br>▪ Use of audit reports<br>▪ Interaction between NPHW and patients	Follow-up visits
**Acceptability** (extent to which providers and patients perceive C-QIP strategy to be agreeable or satisfactory)	▪ Acceptance of DSS prompts.<br>▪ Acceptance of NPHW facilitated care.<br>▪ Acceptance of patient diary and VITA tool<br>▪ Acceptance of audit reports<br>▪ Acceptance of text-messages	Follow-up visits
**Costs**	▪ Cost of C-QIP intervention development and delivery	During trial duration
**Appropriateness**	▪ Perceived fit, relevance, or compatibility of C-QIP strategy at the participating sites (2 public and 2 private hospitals)	Using qualitative interviews before and after the C-QIP implementation

Secondary and Exploratory endpoint analyses

Analysis of secondary outcomes (prescription of guideline-directed medical therapy and adherence to prescribed therapy), plus exploratory outcomes (mean change in systolic BP and LDLc) will be conducted using standard statistical procedures applicable to categorical or continuous data. All secondary/exploratory endpoints data will be summarized in the form of frequency distributions and descriptive statistics at each visit and by treatment group using tabular form.

Secondary analyses: Prescription of GDMT and Adherence to CVD medications

Generalized estimating equation (GEE) analysis using data available at baseline and end of study visit (12 or 24 months), to account for correlation of observations within participants over time will be used. The log binomial GEE model will be used for the outcome's prescription of GDMT and adherence to CVD medications. In case of convergence issues with the log-binomial model, poison regression will be used to conduct the analysis.(Guangyong Zou).

Exploratory analyses: Mean change in systolic blood pressure and LDL-cholesterol

Similarly, the longitudinal analysis for exploratory outcomes (mean change in systolic BP and LDLc, health status and adherence to recommended lifestyle habits: smoking cessation, diet, physical and activity) will be performed using linear regression with a generalized linear model (Zerger and Liang, 1988).

For both secondary and exploratory outcomes, the model will include the following terms: treatment group, time as categorical variable, a treatment by time interaction and the baseline values.

To account for within-subject correlations, we will assume that errors are correlated according to compound-symmetry covariance structure and will use Generalized Estimating Equations (GEE) (Zeger and Liang, 1988).

Since the 'end of study' visit can happen at any time during follow-up, data collected at the 'end of study' visit will be reallocated to one of scheduled visits (Month 12, or 24) according to the process described below. Missing outcome data will not be imputed.

We will also test pairwise differences between treatment groups for each visit separately.

Definition of end of follow-up

The study secondary outcomes such as prescription of GDMT, adherence to prescribed therapy, mean change in systolic BP and LDLc, and adherence to recommended lifestyle habits: smoking cessation, diet, physical and activity at trial end. will be assessed between treatment group during the trial and at the end of

study (EOS) visit. Because patients will not all be followed for the same duration, depending on when they were randomized, end of follow-up data will be defined as data collected at the last visit performed (based on the assessment date recorded on the last follow-up assessment form). The End of Study (EOS) visits are planned to occur between June - August 2024 with average follow up duration of ~18months, and the window for the EOS visit is +/- 15 days. Average follow up duration takes into consideration first randomized patient last visit (24 months) and Last randomized patient last visit (12 months).

Adjusted Analyses- For Secondary and Exploratory outcomes.

The adjusted analysis for proportion of intervention vs usual care participants receiving GDMT, adherence to prescribed therapy at end of follow-up will be conducted using log-binomial regression containing the site, age, sex, and other covariates which will be identified as significant at baseline comparisons.

In case of convergence issues with the log-binomial model, poison regression will be used to conduct the analysis.

The adjusted analysis for mean change in systolic BP and LDLc between the intervention and usual care participants will be done using the linear regression with Generalised estimating equation (GEE) model adjusted for other covariates.

The adjusted model will include the treatment group, time as a categorical variable, a treatment-by-time interaction and the baseline values, age, sex and other covariates which will be identified as significant at baseline comparisons.

Sub-group analysis

The sub-group analyses are planned for secondary outcomes: i) adherence to prescribed medications, and ii) mean reduction in systolic blood pressure, using the following variables:

- Age: <49 years; 50-64 years, >64 years
- Sex: male vs female
- Education (years): <5, 5-10, 10-12, >12
- Household income (INR): <15,000, 15,000-30,000, >30,000
- Facility type: public vs private
- BMI (Kg/m^2): <25, >=25
- History of hypertension: yes or no
- History of diabetes: yes or no
- History of coronary heart disease: yes or no
- History of stroke: yes or no
- History of heart failure: yes or no
- Baseline SBP: <140 and 140+

- Baseline LDLc: <100 and 100+

In each subgroup analysis a model will include the subgroup variable along with its interaction with treatment. A test of whether the treatment effect differs across the levels of the subgroup will be constructed by assessing the significance of the interaction term. The results of these subgroup analyses will be treated with caution as this study was not powered for these analyses. Forest plots containing the same information will be prepared.

Missing data handling

The analysis will be according to the intention to treat principle. The baseline characteristics of the participants who completed and did not complete the final visit will be compared. We do not plan to impute the missing data for the secondary outcomes (prescription of GDMT, adherence to prescribed therapy, and mean change in BP or LDLc).

Safety Analysis

All serious adverse event data collected will be reported for the overall study population and by the treatment group.

Interim Analysis

No interim analysis is planned for this study. A DSMB will be constituted and will meet at enrolment of 50% to assess safety and efficacy data of each group of the study, with more frequent meetings as appropriate for safety and efficacy reason.

Independent Data Safety Monitoring Board (DSMB)

The study will use an independent DSMB to evaluate the outcome and safety data in the context of the overall study. The DSMB will determine the appropriate time frame for reviewing these data. A subset of the shell tables shown in this analysis plan can be used for the DSMB reports.

References

Guangyong Zou, A Modified Poisson Regression Approach to Prospective Studies with Binary Data, American Journal of Epidemiology, Volume 159, Issue 7, 1 April 2004, Pages 702–706, https://doi.org/10.1093/aje/kwh090).

Zeger, S. L., K. Y. Liang, et al. (1988). "Models for Longitudinal Data - a Generalized Estimating Equation Approach." Biometrics 44(4): 1049-1060.

Appendix: Templates for Tables, Data Listings and Figures

Figure 1: Registration, randomization and follow-up of study participants (CONSORT diagram)

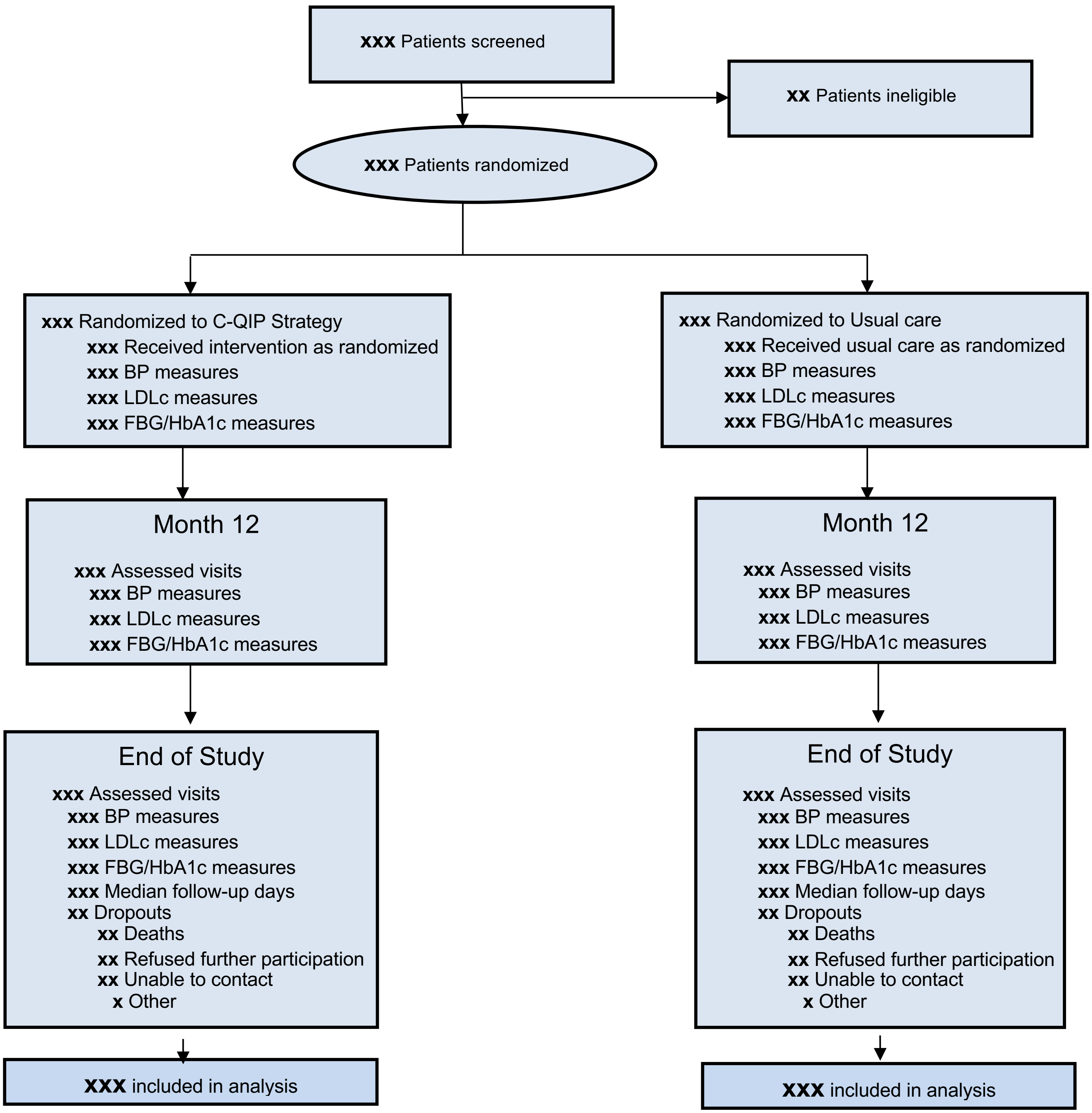

Table 1. Baseline characteristics by treatment group

	**Overall**	**C-QIP Strategy**	**Usual care**
Age (years; mean, SD)			
Male, n (%)			
Education (years; mean, SD)			
Household Income (monthly) Indian rupees, mean (SD)			
Systolic BP (mm Hg; mean, SD)			
Diastolic BP (mm Hg; mean, SD)			
Total cholesterol (mg/dL; mean, SD)			
LDLc (mg/dL; mean, SD)			
FBG (mg/dL; mean, SD)			
HbA1c (%; mean, SD)			
Coronary Heart Disease, n (%)			
Myocardial Infarction, n (%)			
Heart failure, n (%)			
Ischemic stroke, n (%)			
Hypertension, n (%)			
Diabetes, n (%)			
Hypertension and Diabetes, n (%)			
Antiplatelet			
Lipid lowering (Statin)			
ACEi/ARB			
ARNI			
Beta-blockers			
CCBs			
Diuretics			
Participants with Coronary Heart Disease			
Antiplatelet + Lipid lowering drug (statin) + ≥ 2 anti-hypertensive drugs			
Antiplatelet + Lipid lowering drug (statin) + ACEi or ARB + Beta Blocker			
Participants with Ischemic stroke			
Antiplatelet + Lipid lowering drug (statin)			
Antiplatelet + Lipid lowering drug (statin) + ACEi or ARB / Diuretic			
Antiplatelet + Lipid lowering drug (statin) + ≥ 2 anti-hypertensive drugs			
Participants with Heart failure			
ACEi / ARNI + Beta blocker + MRA			
ACEi/ARNI + Beta blocker + MRA + SGLT2i			
Participants forgot to take medicines in last one month, n (%)			

Participants stopped medication on his/her own, n (%)			

Table 2. Implementation Outcomes for Intervention arm participants

	Baseline visit	Final study visit
**Feasibility measures**		
**Recruitment rate**		
**Retention rate**		
**Drop outs**		
**Proportion of patients completing final study visit**		
**Retention of NPHW**		
**Fidelity measures**		
**No. of DSS prompts generated**		
**No. of DSS prompts reviewed**		
**No. of DSS prompts accepted**		
**No. of DSS prompts rejected**		
**No. of audit and feedback reports generated**		
**No. of audit and feedback reports reviewed by sites**		
**No. of text messages sent to the patients**		
**Adoption measures**		
**No. of patients who discontinued**		
**No. of patients who reported using patient diary**		
**No. of patients who reported using VITA tool**		
**No. of patients who reported receiving text messages**		
**Acceptability measures**		
**Physician's acceptability of C-QIP strategy**		
**Patient's treatment satisfaction**		
**Nomad tools results**		
**CSAT tools results**		

Table 3: Secondary outcomes: Processes of care and clinical measures at the **end of the study** by treatment group

	C-QIP Strategy %, [95% CI]	Usual care %, [95% CI]	Absolute Difference* [95% CI]	Relative Risk** [95% CI]
**Processes of care measures (%)**				
**Medications use**				
**Antiplatelet**				
**Lipid-lowering (Statin)**				
**ACEi/ARB**				
**ARNI**				
**Beta-blockers**				
**CCBs**				
**Diuretics**				
**GDMT for patients with CHD**				
**Antiplatelet + Lipid lowering drug (statin) + ≥ 2 anti-hypertensive drugs**				
**Antiplatelet + Lipid lowering drug (statin) + ACEi or ARB + Beta Blocker**				
**GDMT for patients with stroke**				
**Antiplatelet + Lipid lowering drug (statin)**				
**Antiplatelet + Lipid lowering drug (statin) + ACEi or ARB / Diuretic**				
**Antiplatelet + Lipid lowering drug (statin) + ≥ 2 anti-hypertensive drugs**				
**GDMT for patients with Heart failure**				
**ACEi / ARNI + Beta blocker + MRA**				
**ACEi/ARNI + Beta blocker + MRA + SGLT2i**				
**Participants forgot to take medicines in last one month, n (%)**				
**Participants stopped medication on his/her own, n (%)**				
**Time spent in-person with doctor, median (IQR)**				
**Time spent with dietician, nurse or clinical staff receiving self-care education**				
**Lifestyle habits and self-care practices**				
**Adherence to diet plan**				
**Adherence to physical activity**				
**Adherence to prescribed treatment**				
**Health service use, mean (SD)**				
**Clinic visits**				
**BP checked**				
**Cholesterol testing**				

**Blood sugar testing**				
**ECG test**				
**Dietician visit**				
**Treatment satisfaction score**				
**Clinical measures, mean (SD)**				
**Systolic BP (mmHg)**				
**Diastolic BP (mmHg)**				
**Heart rate**				
**Total cholesterol (mg/dL)**				
**HDL cholesterol (mg/dL)**				
**Triglycerides (mg/dL)**				
**LDLc (mg/dL)**				
**Fasting blood glucose (mg/dl)**				
**HbA1c (%)**				
**Serum creatinine**				
**Weight (Kg)**				
**BMI (Kg/m2)**				

*
Model will include treatment group, baseline values, time, interaction between time x treatment group

Figure 2. Sub-group analysis: Adherence to prescribed treatment and for mean reduction in systolic blood pressure by major sub-groups

- Age: <49 years; 50-64 years, >64 years
- Sex: male vs female
- Education (years): <5, 5-10, 10-12, >12
- Household income (INR): <15,000, 15,000-30,000, >30,000
- Facility type: public vs private
- BMI (Kg/m^2): <25, >=25
- History of hypertension: yes or no
- History of diabetes: yes or no
- History of coronary heart disease: yes or no
- History of stroke: yes or no
- History of heart failure: yes or no
- Baseline SBP: <140 and 140+
- Baseline LDLc: <100 and 100+

Table 4. Serious adverse events by treatment group

		Total events reported in trial		
**No.**	**Event description**	**Total**	**C-QIP Strategy**	**Usual care**
1	Death			
2				
3				
4				
5				
6				
7				
