# Supplementary material for: Assessing a multicomponent quality improvement strategy for chronic care of cardiovascular diseases in India: The C-QIP randomized feasibility trial
Source: PLoS Med. 2026 Sep 29;23(9):e1004932. doi: 10.1371/journal.pmed.1004932 (PMC13641716; doi:10.1371/journal.pmed.1004932)
Supplement: Supplementary file 2 [file pmed.1004932.s007.pdf]

Developing and testing a Collaborative Quality ImProvement (C-QIP) initiative for prevention of cardiovascular disease in India

Final Version 1.2 Date 7 February 2022

Short title: C-QIP Study

Coordinating Centre: Public Health Foundation of India

Project number: 1K43TW011164

Funding Source: Fogarty International Centre, National Institutes of Health, USA

STUDY PERSONNEL AND CONTACT DETAILS

**Sponsor:**	Public Health Foundation of India
**Principal investigator:**	**Kavita Singh, PhD**<br>Senior Research Scientist<br>Centre for Chronic Conditions and Injuries<br>Public Health Foundation of India.<br>Adjunct Assistant Professor, Emory University, USA<br>Plot number 47, Sector 44<br>Gurgaon, Haryana, India<br>Email: Kavita.singh@phfi.org<br>Telephone: 011-4608 2601
**Primary Mentor (India)**:	**Dorairaj Prabhakaran**, MD, DM (Cardiology), Vice President (Research and Policy), Public Health Foundation of India, Gurugram, Haryana, India
Secondary Mentor (India):	**Nikhil Tandon**, MD, PhD, Professor and Head, Department of Endocrinology, All India Institute of Medical Sciences, New Delhi, India
**Primary Mentor (USA)**:	**Mark D. Huffman**, MD, MPH, Professor of Medicine, Co-Director Global Health Center, Washington University School of Medicine, St. Louis, USA, and Adjunct Professor of Preventive Medicine (Epidemiology), Northwestern University, Chicago, USA
Secondary Mentor (USA):	**K.M. Venkat Narayan**, MD, Professor of Medicine, and Global Health, Emory University, Atlanta, USA
Secondary Mentor (USA):	**Shivani Patel**, PhD, Assistant Professor of Global Health, Emory University, Atlanta, USA
Secondary Mentor (USA):	**Emily Mendenhall**, PhD, Professor of Global Health, Georgetown University, Washington, D.C., USA

PARTICIPATING CLINIC SITES

**SITE Principal Investigator(s)** involved in the study:	**Full mailing address:**
**(1) NAME : Ambuj Roy**<br>Qualifications : MD, DM<br>Designation: Professor, Department of Cardiology<br>Mobile: +91- 9810992822<br>E-mail: drambujroy@gmail.com	Dr. Ambuj Roy<br>Department of Cardiology<br>All India Institute of Medical Sciences<br>Ansari Nagar – 110 029,<br>New Delhi, India
**(2) NAME: Mohit Gupta and Girish MP**<br>Qualifications: MD, DM<br>Designation: Professor, Department of Cardiology<br>Mobile: +91-9810121311<br>E-mail: drmohitgupta@yahoo.com ; mpgirish_l999@yahoo.com	Dr. Mohit Gupta / Dr. Girish MP<br>Department of Cardiology,<br>GB Pant Hospital,<br>1, Jawaharlal Nehru Marg, 64<br>Khamba, Raj Ghat, New Delhi,<br>Delhi 110002
**(3) NAME: Kushal Madan and JPS Sawhney**<br>Qualifications: MD, DNB, DM<br>Designation: Professor of Cardiology<br>Mobile: +91-9810688410<br>E-mail: kushalmadan@gmail.com; jpssawhney@yahoo.com	Dr. Kushal Madan<br>Department of Cardiology,<br>Sir Ganga ram Hospital,<br>Sarhadi Gandhi Marg, Old Rajinder<br>Nagar, Rajinder Nagar,<br>New Delhi, 110060
**(4) NAME: Kiran Aithal and Sathish Patil**<br>Qualifications: MD, DM<br>Designation: Professor and Head, Department of Medicine, and Department of Physiology<br>Mobile: +91- 9986789583<br>E-mail: drkiranaithal@yahoo.com; sathupatil@yahoo.co.in	Dr. Kiran Aithal, Dr. Sathish Patil<br>Department of Medicine,<br>SDM College of Medical Sciences and<br>Hospital, Manjushree Nagar,<br>Sattur Colony, Karnataka 580009

Contents

STUDY PERSONNEL AND CONTACT DETAILS 2

PARTICIPATING CLINIC SITES 3

ABBREVIATIONS AND ACRONYMS 6

PROTOCOL SYNOPSIS 7

SPECIFIC AIMS 10

RESEARCH STRATEGY 11

A. SIGNIFICANCE 11
A.1. BURDEN OF CARDIOVASCULAR DISEASES 11
A.2. THE EVIDENCE-PRACTICE GAPS FOR CARDIOVASCULAR DISEASE 11
A.3. INDIAN CONTEXT: BARRIERS TO CARDIOVASCULAR DISEASE CARE 12
A.4. RATIONALE FOR QUALITY IMPROVEMENT INITIATIVES 12
B. INNOVATION 13
C. APPROACH 14
C.1. OVERVIEW 14
C.2. SPECIFIC AIMS 1 AND 2 [PHASE 1: DEVELOPMENT PHASE] 14
C.2.1. REVIEW OF EVIDENCE 14
C.2.2. QUALITATIVE RESEARCH 15
C.2.3. EXPERT CONSULTATION MEETING 16
C.3. SPECIFIC AIMS 3 AND 4: FEASIBILITY TRIAL [PHASE 2: EVALUATION PHASE] 16
C.3.1. OVERVIEW 16
C.3.2. STUDY DESIGN 16
C.3.3. SELECTION AND ENROLMENT OF PARTICIPANTS 17
SETTING 17
PATIENT ELIGIBILITY CRITERIA 17
C.3.4. METHODS OF RECRUITMENT 17
C.3.5. INFORMED CONSENT 18
C.3.6. SCREENING AND BASELINE ASSESSMENT 18
RANDOMIZATION 18
C.3.7. STUDY INTERVENTION 19
DECISION-SUPPORT SOFTWARE 20
NON-PHYSICIAN HEALTH WORKER 21
TEXT-MESSAGES FOR HEALTHY LIFESTYLE 21
PATIENT DIARY 22
QUARTERLY AUDIT AND FEEDBACK REPORT 22
C.3.8. CONTROL ARM (USUAL CARE) 22
C.3.9. STUDY PROCEDURES 23
SCHEDULE OF EVALUATIONS 23
BASELINE ASSESSMENT VISIT 24
FOLLOW-UP VISIT 25
INTERMEDIATE VISITS 25
END OF STUDY VISIT 26
C.3.10. SAFETY ASSESSMENTS 26
ADVERSE EVENTS 26
EXPECTED RISKS 27
MEASURES TO MINIMIZE RISK 27
C.3.11. STUDY OUTCOMES 28
PRIMARY OUTCOMES 28
SECONDARY OUTCOMES 28
C.3.12. STATISTICAL CONSIDERATIONS 28
SAMPLE SIZE AND POWER CALCULATION: 28
DATA ANALYSIS 28
3.13. PROCESS EVALUATION 28
C.3.14. ECONOMIC EVALUATION 29

C.3.15. DATA MANAGEMENT 30
C.3.16. QUALITY ASSURANCE AND MONITORING 31
C.4. ETHICAL CONSIDERATIONS 31
C.4.1. RESEARCH ETHICS APPROVAL 31
C.4.2. INFORMED CONSENT 31
C.4.3. CONFIDENTIALITY 31
C.5. STUDY ORGANIZATION 32
C.6. PUBLICATION OF RESEARCH FINDINGS 32
C.7. STUDY DURATION AND TIMELINES 32
C.8. STUDY DISCONTINUATION 33
C.9. STUDY IMPLICATIONS 33
D. REFERENCES 35

ABBREVIATIONS and ACRONYMS

ACEi	Angiotensin Converting Enzyme inhibitor
BMI	Body Mass Index
BP	Blood Pressure
CAD	Coronary Artery Disease
CCC	Cardiovascular Care Coordinator
CHD	Coronary Heart Disease
CRF	Case Report Form
CVD	Cardiovascular Disease
C-QIP	Collaborative Quality Improvement
DSS	Decision Support Software
EHR	Electronic Health Record
FIC	Fogarty International Centre
GCP	Good Clinical Practice
GDMT	Guideline Directed Medical Therapy
HbA1c	Glycated Haemoglobin
HrQoL	Health related Quality of Life
ICH	International Conference on Harmonisation
ICMR	Indian Council of Medical Research
ID	Identification number
IT	Information Technology
IWRS	Interactive Web Response System
LAR	Legally Acceptable Representative
LDLc	Low-density lipoprotein cholesterol
NPHW	Non-Physician Health Worker
MRC	Medical Research Council
NCD	Non-Communicable Disease
NIH	National Institutes of Health
OPD	Out-Patient Department
PHFI	Public Health Foundation of India
PI	Principal Investigator
QI	Quality Improvement
RCT	Randomized Controlled Trial
RCC	Research Coordinating Centre
SD	Standard Deviation
SMS	Short Message Service
StaRI	Standards for Reporting Implementation Studies
UK	United Kingdom
USA	United States of America
VITA	Visual Assessment Tool of Medication Adherence
WHO	World Health Organisation

PROTOCOL SYNOPSIS

**Title**	Developing and testing a Collaborative Quality ImProvement initiative for prevention of cardiovascular disease (CVD) in India
**Short title**	C-QIP Study
**Principal Investigator**	Dr. Kavita Singh
**Objectives**	1. To describe current practices, context, challenges, and opportunities regarding chronic management of CVD from patient, caregiver, provider, and health administrator perspectives in India.<br>2. To assess the transferability of components of internationally successful multifaceted quality improvement (QI) strategies and chronic care models to the Indian healthcare context.<br>3. To conduct a pilot study to assess the acceptability, feasibility, and implementation fidelity of C-QIP strategy among individuals with CVD attending out-patient clinics in India.<br>4. Among individuals with CVD, evaluate the effect of C-QIP strategy on processes of care and clinical outcomes, health-related quality of life and costs at 1 year compared with usual care.
**Study type**	Hybrid type 1 implementation study using mixed-methods study design and a feasibility randomized controlled trial
**Setting**	Hospital-based study in Delhi and Karnataka
**Sample size estimate**	The proposed study is a feasibility, randomized controlled trial design, with a primary focus being on the development of locally adaptable and culturally sensitive multifaceted collaborative quality improvement (C-QIP) strategy and to understand the feasibility, acceptability, fidelity, and preliminary effects of the C-QIP strategy on process measures (prescription of and adherence to guideline directed medical therapy) and clinical outcomes (changes in blood pressure and blood cholesterol). We will select 4 CVD clinics (2 government and 2 private, 100 patients each), and will recruit and randomize a total of 400 patients (1:1, stratified by site) to assess the impact of the C-QIP strategy by examining feasibility and acceptability of C-QIP strategy and its preliminary effect on processes of care (prescription rates and adherence rates), clinical outcomes (changes in blood pressure and blood lipids), health-related quality of life, and costs of care between the treatment groups at the end of 1 year of intervention delivery. Also, the results of this feasibility trial, will inform sample size estimates and power calculation for a future outcomes-driven, larger randomized trial to study the effectiveness, cost-effectiveness, and scalability of C-QIP strategy for secondary prevention of CVD.<br><br>A qualitative study will be conducted with participants in both groups to explore their experience of taking part in the study. Depending on the saturation of themes, semi-structured interviews will be conducted with around 40 participants and care providers at the four participating sites. Purposive sampling will be utilised to ensure representation of diversity within the feasibility RCT population. Those who decline to participate in the study will be invited to complete a brief questionnaire and to be interviewed to explore reasons behind their non-participation. A qualitative study will be conducted with the non-physician health workers (n=4; 1 per site) to mainly explore their experience of delivering the C-QIP strategy.
**Number of participants**	Phase 1 (Development of C-QIP strategy) – 40 patients and their caregivers, 20 providers, and 10 hospital administrators will be invited for the in-depth qualitative interviews.<br><br>Phase 2 (Feasibility trial) – 400 patients with CVD from 4 clinics in Delhi and Karnataka will be invited to participate in the study.
**Eligibility criteria**	Inclusion criteria: Participants should be:<br>• age ≥18 years, both sexes

	• confirmed diagnosis of CVD (ischemic heart disease, ischemic stroke, or heart failure irrespective of ejection fraction)<br>• able to provide written informed consent.<br>Exclusion criteria:<br>• pregnant women<br>• those with any serious or uncontrolled medical condition (e.g., cancer) that may restrict patient to come for follow-up.
**Description of interventions**	Intervention (C-QIP):<br>The specific components of the C-QIP strategy are informed by the results of formative research conducted during the overall study aims 1 and 2. The C-QIP strategy will consist of following components: 1) Electronic health records and clinical decision-support system to enhance physician's responsiveness of timely treatment modification. 2) Team-based care facilitated by a trained and supervised non-physician health worker (NPHW) also called as Cardiovascular Care Coordinator (CCC) to educate, empower and encourage patients for self-management. 3) Text-messages for healthy lifestyle. 4) Patient diary as a reinforcement tool for lifestyle modification and **Vi**sual assessment **t**ool for medication **a**dherence (VITA), 5) Quarterly audit and feedback report for the care providers.<br><br>The C-QIP strategy will involve training of physicians and non-physician health workers (CCC) to support patient self-management and follow up, navigation of clinical services and treatment coordination and on the use of electronic health records-decision-support system to aid shared decision making between care providers and patients.<br><br>Control (usual care): Currently, no C-QIP type strategy is available in India for people with CVD. The control group participants will receive a leaflet (printed information) for healthy lifestyle. This will be delivered by a different team member (i.e., not by the trained NPHW) to avoid contamination. This provision would ensure that the control group participants feel that there are benefits to participation (hence, lower attrition). All physicians will be provided current treatment guidelines for the management of CVD.
**Duration of study**	**Overall**: 60 months<br><br>Phase 1 formative research to inform the design and development of C-QIP strategy over 24 months.<br><br>Phase 2 comprising of pilot testing of intervention materials at 1 site for 3-6 months, followed by a formal evaluation of C-QIP strategy in an individual randomized trial over 24 months (Proposed start date of the feasibility study: April 2022). 6 months recruitment period, and average 12 months follow-up.<br>Last 6 months are earmarked for data cleaning, analysis, and report writing/dissemination of study findings.<br><br>Per participant:<br>Each participant will be involved for at least 1 year (intervention delivery/follow-up). Subsequently, one semi-structured interview will be conducted with some of them.
**Methods of analysis**	Feasibility trial: We will report the baseline demographics, clinical history, examination, processes of care measures and laboratory test results among the participants before and after the implementation of C-QIP strategy, adjusted for age, sex, and site. The primary analyses will be conducted under the intention to treat principle. All randomized patients will be analysed in the groups to which they were originally allocated to, regardless of whether they actually retained that specific group membership over the course of the trial or not. Patients who withdraw consent for use of their data will not be included in any analyses.<br><br>(a) RCT:<br>**Primary outcome**

	Implementation outcomes: feasibility (screening, recruitment, randomization, and follow-up rates, time needed to collect and analyse data); fidelity (adherence to study protocol), adoption and acceptability from the perspectives of patients and providers (use of EHR-DSS management plan for the intervention arm patients, number of DSS prompts accepted by the physicians, use of text-messages as reminders for next clinic visit, lab appointment and health lifestyle, number of text-messages sent to the patients).<br><br>**Secondary outcomes**: Processes of care measures (prescription of guideline directed medical therapy (GDMT), and patient's adherence to prescribed GDMT). In patients with established CVD, guidelines recommend prescription of two blood pressure lowering drugs (ACEi/ARB, beta-blocker), aspirin, and statins.<br><br>**Exploratory outcomes**: clinical changes in blood pressure, LDL-cholesterol, measures of variance in blood pressure and LDLc i.e., standard deviation (SD) of the outcome measure (i.e., mean change in systolic blood pressure and LDL-cholesterol level between the two treatment arms at trial end), other biochemical parameters (glycated haemoglobin (HbA1c) and lipid profile), physiological parameters (blood pressure and heart rate), anthropometric parameters (waist circumference, weight, and body mass index (BMI)), diet, physical activity, tobacco use, health related quality of life and mental health (assessed using Global health scale), and cost of care.<br><br>Numbers and percentages (for categorical data), and summary measures of mean and standard deviation (for continuous data) will be reported. Being a feasibility study, it is not adequately powered to detect a difference in clinical outcomes between the two study arms. However, appropriate regression methods will be used to indicate efficacy of the intervention. Effect sizes will be presented as risk ratios for binary outcomes, and as mean differences for continuous outcomes; 95% confidence intervals will be given for both. Data analysis will be performed using STATA 16.0 (Statacorp Texas).<br><br>(b) Process evaluation/Qualitative study: The Implementation Research Logic Model guided by Consolidated Framework for Implementation Research (CFIR) and the RE-AIM (reach, effectiveness, adoption, implementation, and maintenance) framework will be used to inform the development of the intervention and process evaluation, respectively. All the semi-structured interviews will be transcribed (verbatim), translated, anonymised, and checked for accuracy. An interpretive analysis will be conducted using thematic analysis, using MAXQDA software. Transcripts will be read and re-read. Initial codes will be developed and applied initially to a small number of transcripts, enabling further iteration of the thematic index. We will use illustrative non-attributable quotations.

SPECIFIC AIMS

Globally, cardiovascular disease (CVD) causes around 18 million deaths each year and around 35 million people experience non-fatal cardiovascular events[1]. Having CVD increases the risk of recurrent myocardial infarction, stroke, heart failure or cardiovascular death at 5 years by 20-30% as compared to those without CVD[2]. South Asians develop CVD at a younger age and have the highest age-standardized CVD mortality compared with other regions (except some eastern European countries), leading to productivity loss, poor economic growth, and social challenges. Among those with CVD, secondary prevention drugs (aspirin, beta-blockers, ACE-inhibitors, and statins), and lifestyle interventions (smoking cessation, physical activity, healthy diet, and stress management) are effective for reducing premature mortality and morbidity[1,3,4]. However, knowledge translation is deficient such that there is a 55%-point gap between CVD event reduction due to multi-drug CVD therapy in efficacy trials compared with real-world effectiveness. This gap is ostensibly due to several factors related to prescription rates (provider-level) and adherence rates (patient-level)[5-7].

Despite a large need, strategies to improve adherence to CVD drugs in low- and middle- income countries (LMIC) remain understudied and undeveloped. For example, a 2011 report of the Prospective Urban Rural Epidemiology (PURE) study that enrolled 28,747 participants from India (3% with prevalent CVD) demonstrated that less than 10% of patients with CVD were taking a combination of aspirin, statins, and BP lowering drugs[8]. A 2014 Cochrane systematic review found that although interventions to improve adherence have been tested in high-income countries, many were marginally effective, resource intensive, and not readily scalable[9]. Several factors at the patient, provider, and health system levels (e.g., literacy, increased time spent with patients, and integrated health care) may effectively reduce CVD burden in India. Multifaceted quality improvement interventions (e.g., non-physician health worker, SMS reminders, decision-support software, audit-and feedback mechanisms) have been successful in high-income countries for improving care among individuals with prevalent CVD but have not been extensively evaluated in India. These interventions could be sources of innovation in CVD prevention, treatment, and control through implementation science research.

The overarching goal of this proposal is to develop, implement, and evaluate the effect of a **Collaborative Quality ImProvement (C-QIP)** intervention (consisting of non-physician health workers, text messages for healthy lifestyle and a clinical decision-support system) on processes of care and clinical outcomes among individuals with prevalent CVD in India using the United Kingdom's Medical Research Council (MRC) framework for developing and evaluating complex interventions[10].

The **specific aims** are:

Aim 1: To describe current practices, context, challenges, and opportunities regarding chronic management of CVDs from patient, caregiver, provider, and health administrator perspectives in India.

Hypothesis: I will identify positive and negative patient, caregiver, provider and health administrator level characteristics, practices, and context that influence overall chronic management of CVDs.

Aim 2 - To assess the *transferability* of components of internationally successful multifaceted quality improvement (QI) strategies and chronic care models to the Indian healthcare context.

Hypothesis: Adaptation of existing successful quality improvement models, such as chronic care model, will be acceptable and feasible among CVD clinics in India.

Aim 3: To conduct a pilot study to assess the acceptability, feasibility, and implementation fidelity of C-QIP strategy among individuals with CVD attending out-patient CVD clinics in India.

Hypothesis: C-QIP strategy will be acceptable to providers, patients and feasible to implement within the existing resources and infrastructure at the public or private hospitals in India.

Aim 4: Among individuals with CVD, evaluate the effect of C-QIP strategy on processes of care and clinical outcomes, health-related quality of life and costs at 1 year compared with usual care.

Hypothesis: The intervention group participants will have improved process measures, improved adherence to therapy, higher quality of life, better clinical outcomes, and lower cost of care.

Implications: Given the epidemic proportion of CVD burden in India, the evidence-practice gaps in implementation of effective interventions, as well as heterogeneity in access and care delivery, the proposed study will implement locally developed/adapted, action-oriented, integrated, and multifaceted C-QIP strategy complementing the existing infrastructure and activities emanating from the national program for prevention and management of CVD in India.

RESEARCH STRATEGY

A. SIGNIFICANCE

A.1. Burden of cardiovascular diseases

Cardiovascular disease (CVD) now leads to more death and disability in low- and middle- income countries (LMIC), such as India, than in high-income countries, with age-adjusted prevalence rates that are increasing, while comparable rates in high-income countries are decreasing[11]. CVD also affects Indians at a younger age than their counterparts in developed countries, as well as many other developing countries[12].

Many evidence-based interventions for prevention, treatment, and control of CVD are available and are widely promulgated in international clinical practice guidelines. However, significant gaps between the evidence and implementation persist ('evidence–practice gap') for even basic pharmacotherapy including aspirin, statin, blood pressure lowering drugs, and tobacco cessation strategies[13,14]. To better tackle the global burden of CVD, more effective strategies for equitable dissemination and application are needed[7,15]. This is aligned with the World Health Organization's 25x25 goal[4] (*to reduce the risk of premature [30 to 69 years] mortality from non-communicable diseases, including cardiovascular diseases by 25%, by 2025*) and is aligned with the Fogarty International Center's Strategic Plan (Goals 1-4) to build research capacity, stimulate innovation, and support research in implementation science to reduce the global burden of non-communicable diseases[16]. A better assessment and understanding of the most important modifiable context-specific barriers to evidence-based care will allow optimal tailoring of interventions to overcome them[15].

A recent systematic review found that interventions to improve adherence to medications have been mostly complex in nature (transdisciplinary approach, a pharmacist consultation service, and a discharge educational session with materials), which makes adaptation, implementation, scalability, and sustainability difficult in practice[17]. However, low-cost, relatively simple, innovative, and—most importantly— effective health system level interventions can enhance the adherence to prescribed therapy and clinical outcomes. Examples of such interventions include mobile phone text messaging, non-physician health workers to facilitate care for patients with CVD, and interactive web-based or mHealth clinical decision support system to enhance providers responsiveness. I have substantial experience in evaluating these interventions, but most are pilots/fragmented and largely focused in management of diabetes and hypertension.

A.2. The evidence-practice gaps for cardiovascular disease

Despite the high-quality evidence-base for CVD prevention drugs (aspirin, statins, and blood pressure lowering drugs) and incorporation of this evidence in clinical practice guidelines,[18-21] a substantial gap between recommended treatment and clinical practice remains. Typically, the five-year risk of recurrent CVD event is 20-30%,[22] and the overall estimate for effect attributable to CVD drug treatment in secondary prevention of CVD is a 75% reduction with a low rate of observed side effects[23-28]. However, community-based studies indicate low use of drug therapies for secondary prevention of CVD, including in India[8]. For example, beyond the aforementioned PURE data, CVD was found to be the leading cause of death in 2003-04 in a rural region of Andhra Pradesh[29]. Less than one-sixth of those with a previous cardiovascular event were receiving antiplatelet therapy, and fewer than 20% of individuals with established CVD were prescribed a combination of two or more proven therapies for vascular risk modification[29,30].

Low medication adherence rates comprise a well-documented, highly prevalent obstacle to successful ongoing prevention and treatment of chronic diseases like CVD[31-33]. Barriers to adopting guideline recommendations by doctors may include lack of time, multiplicity, and lack of awareness of guidelines, and insufficient resources to implement recommendations[33,34]. From a patient's perspective, non-adherence is associated with taking multiple medicines with complex dosing regimens[35,36]. Recent placebo-controlled trials show that pharmacological side-effects are not the major contributor to non-adherence to common cardiovascular medications. In long-term trials of low-dose aspirin, blood pressure and cholesterol lowering drugs, only 1-3% of people stopped medication due to side effects attributable to the medicines, but a further 10-25% stopped for other reasons. For example, in the Perindopril pROtection aGainst REcurrent Stroke Study (PROGRESS), 13% of people in the perindopril group stopped all treatment over four years, as did 11% in the placebo group[23]. These findings indicate

that people may be non-adherent to CVD medication for social or economic reasons as opposed to medical reasons.

A.3. Indian context: Barriers to cardiovascular disease care

Factors influencing the CVD care gaps in LMIC such as India are exacerbated for the following reasons:

- *Economic burden on patient and family*: the costs of even a single component of established cardiovascular therapies are prohibitive – a month's treatment can be equal to a month's wages and both the urban and rural poor have competing priorities. For example, our data from Kerala show median out of pocket expenditures for CVD treatment over 15-month follow-up after CVD hospitalization was International dollar (INT$) 2,917 with >50% of respondents reporting catastrophic health expenditure[37].
- *Education*: understanding the importance of taking lifelong medication for a chronic disease is difficult to grasp particularly for conditions that may frequently be free of symptoms; this is especially true among individuals with co-morbidities or competing financial obligations.
- *Inappropriate models of healthcare for the setting.* There is a false anticipation that western models of health care delivery will readily translate to developing countries where there may be a lack of health system building block infrastructure.
- *Access and Supply.* Efficacy may be proven and recognized at a high level, but access and supply represent major challenges.

A.4. Rationale for quality improvement initiatives

Gaziano *et al.*[38] estimate that the combined use of aspirin, beta-blocker, statin, and ACE-I, compared to no therapy, has the potential to prevent 4,043 acute coronary heart disease (CHD) events and 122 strokes per 100,000 over 10 years in South Asia alone. Lim *et al*. estimated that 54% of CHD deaths and 46% of stroke deaths could be avoided in India with the use CVD prevention drugs over 10 years[39]. The health benefits that can be obtained by aggressive implementation of well-known, proven interventions for chronic care of CVD are therefore potentially large, but **translation of care interventions from controlled environments to delivery in real-life settings remains a major challenge**.

Of the evidence supporting quality of care improvement strategies, two distinct, but congruent, innovative strategies, in addition to standard physician consultations, that may reform quality of care for patients with chronic conditions within fragmented, chaotic health systems are: **1) task-sharing** CVD care through the introduction of non-physician health worker (NPHW) to empower, encourage, and facilitate care processes have been tested individually in high-income countries and recently in India with promising results, including hard-to-reach populations[40-52]. For example, the Secondary Prevention of coRonary Events After Discharge from hospital (SPREAD) trial[53,54] in India (n=806 participants) showed that a community health worker based intervention in patients with acute coronary syndrome (ACS) after hospital discharge improved adherence to therapy and healthy lifestyles, and improvement in systolic blood pressure at 1 year, and, **2) interactive web-based decision support systems** that aid calculation of risk, provide prompts and reminders[55], while storing serial data in accessible software programs, may stimulate better continuity and more comprehensive CVD care delivery[56]. For example, The Treatment of Cardiovascular Risk using Electronic Decision Support (TORPEDO) study, a cluster randomized trial in 60 Australian primary health care centres (n=38,725 participants) showed that a computer-guided quality improvement intervention improved CVD risk measurement but did not increase prescription rates in high-risk patients.

Together, skilled non-physician health worker and technology combine to offer an achievable, low cost care delivery model for improving uptake of proven interventions. This model has been successfully implemented by our research team in the CARRS Trial (n=1,146; co-PIs: Prabhakaran, Tandon, Narayan; NCT01212328), sponsored by the National Heart, Lung, and Blood Institute (NHLBI). This intervention demonstrated significant benefits in multiple risk factor control among patients with type 2 diabetes in South Asia[56-58], **but as yet has not been tested among patients with CVD**, among whom sub-optimal coordination and care are barriers to effective translation of trial-based evidence and clinical guidelines for CVD prevention.

Prior work: Limited quality improvement programs in India have demonstrated various degrees of success. For example, an ACS registry-based quality improvement program in Kerala demonstrated improvements in symptom-to-door time, door-to-needle time, and appropriate post-ACS discharge

medication rates through community- and provider-level education and process-of-care interventions[59]. More recently, a NHLBI funded cluster-randomized, stepped wedge ACS QUIK trial across 63 hospitals in Kerala (n=21,374; PI: Dr. Huffman, co-mentor: Prabhakaran; NCT02256657) showed improvements in in-hospital and discharge medications. The rate of 30-day major adverse cardiovascular events was lower in the intervention group compared with the control group (5.3% versus 6.4%) but the effect was attenuated after adjusting for clustering and temporal trends[59-62]. More recently, the American College of Cardiology's PINNACLE (Practice Innovation and Clinical Excellence) India Quality Improvement Program [PIQIP], operates as an outpatient registry of CVD patients and has found about half of the patients were taking aspirin (49%), statins (50%), and BP lowering drugs (58%)[63-67]. However, this study has not reported on proportions receiving combination of >2 or >3 CVD drugs and the data may have limitations due to extraction from clinic medical records, which have not been validated. Further, my colleagues and I have evaluated the effectiveness of a multicomponent intervention to improve diabetes care delivery in South Asia. In this CARRS (Centre for Cardiometabolic Risk Reduction in South Asia) Trial, we found that multicomponent intervention (care coordinators and decision-support electronic health records) improved multiple risk factor control (glycaemia, blood pressure and LDL cholesterol) and quality of life among 1,146 patients with poorly controlled type 2 diabetes in India and Pakistan. In the proposed K43 project, I aim to adapt this intervention to the needs of cardiology practices for chronic care of CVD starting with a comprehensive scoping review of existing international successful modes of CVD care, coupled with in-depth interviews with patients, carers, providers, and hospital administrators, first to define the current practices, context, challenges, and opportunities to improve CVD care in India. Further, through an expert consultation process my team and I will contextualize the most effective elements, which will be assessed for feasibility, acceptability, implementation fidelity and effect on process measures, health-related quality of life, cost, and clinical outcomes among patients with CVD. We will leverage the existing platform (**Table 1**) to build upon these preliminary findings by creating a locally-developed and -adapted **c**ollaborative cardiovascular quality improvement intervention as a follow-up to the ACS-QUIK trial[60], CARRS Trial[57], and INPUT (INTerpreting Processes of UMPIRE Trial)[68-70] study to demonstrate the feasibility of implementing a **C**ollaborative **Q**uality **I**m**P**rovement (C-QIP) intervention and study the effect of intervention on processes of care (medication prescription rates, adherence to therapy, risk factor control) and acceptability of intervention from patient and provider's perspectives.

Table 1: Description of studies (prior work)

Study Characteristics	ACS-QUIK Trial	CARRS Trial	UMPIRE Trial
**Participants**	Patients with ACS	Patients with Type 2 diabetes	Patients with CVD
**Intervention**	Admission and discharge checklist, Audit and feedback report, patient education material, and guidelines for rapid response + code blue team.	Non-physician care coordinator + electronic health records – decision support software	Fixed dose combination cardiovascular polypill (4 pills: aspirin, statin, atenolol, lisinopril)
**Comparator**	Usual care	Usual care	Usual care
**Outcomes**	30-day mortality and cardiovascular events, processes of care	Multiple risk factor control (HbA1c<7%, & BP<130/80mmHg or LDLc<100mg/dl)	Adherence, BP and LDLc reduction
**Study design**	Stepped-wedge cluster RCT	Individual RCT	Individual RCT

ACS: Acute Coronary Syndrome, CVD: Cardiovascular Disease, HbA1c: glycated haemoglobin BP: Blood Pressure, LDLc: Low-density lipoprotein cholesterol, RCT: randomized controlled trial

B. INNOVATION

This proposal offers significant contributions as it can generate new knowledge in the following areas. **First**, we will define contextualizing factors that contribute to successes or barriers to implementing a quality improvement intervention for *chronic* CVD management including barriers or enablers to clinic level management performance. This builds on prior research within my own and my team's experience in acute coronary syndrome and chronic diabetes care. **Second**, we will create new evidence on *feasibility and effect of C-QIP strategy on process measures* for patients with CVD, which will be critical for larger, outcomes- driven trials. **Third**, we will define innovative ways to *empower and engage patients* in their healthcare decision-making by introducing a non-physician health worker and interactive web-based or mHealth based decision-support system for chronic CVD care, which will be relevant to other chronic conditions. These interventions are the mainstay for chronic infectious diseases, such as HIV, yet have been understudied in CVD. **Fourth**, given the limited health economic data in LMICs, this

study will *estimate the cost of delivering the C-QIP* strategy versus usual care from health care provider, patient, and societal perspective.

C. APPROACH

C.1. Overview

A hybrid type 1 implementation study using mixed-methods study design comprising of qualitative research to inform the development of a quality improvement strategy (Phase 1 – Development of intervention) and quantitative evaluation of the feasibility, implementation, and effect on processes of care among individuals with CVD using an individual randomized controlled trial (RCT) of a locally-developed and -adapted **C**ollaborative-**Q**uality **I**m**P**rovement (C-QIP) strategy versus usual care (Phase 2 – Evaluation of intervention). The development and evaluation of C-QIP strategy will be guided by the Consolidated Framework for Implementation Research (CFIR)[71,72] and RE-AIM[73,74] (reach, effectiveness, adoption, implementation, and maintenance), respectively[75]. [**Figure 1**]

Figure 1: Development and Evaluation Framework for the Proposed Study

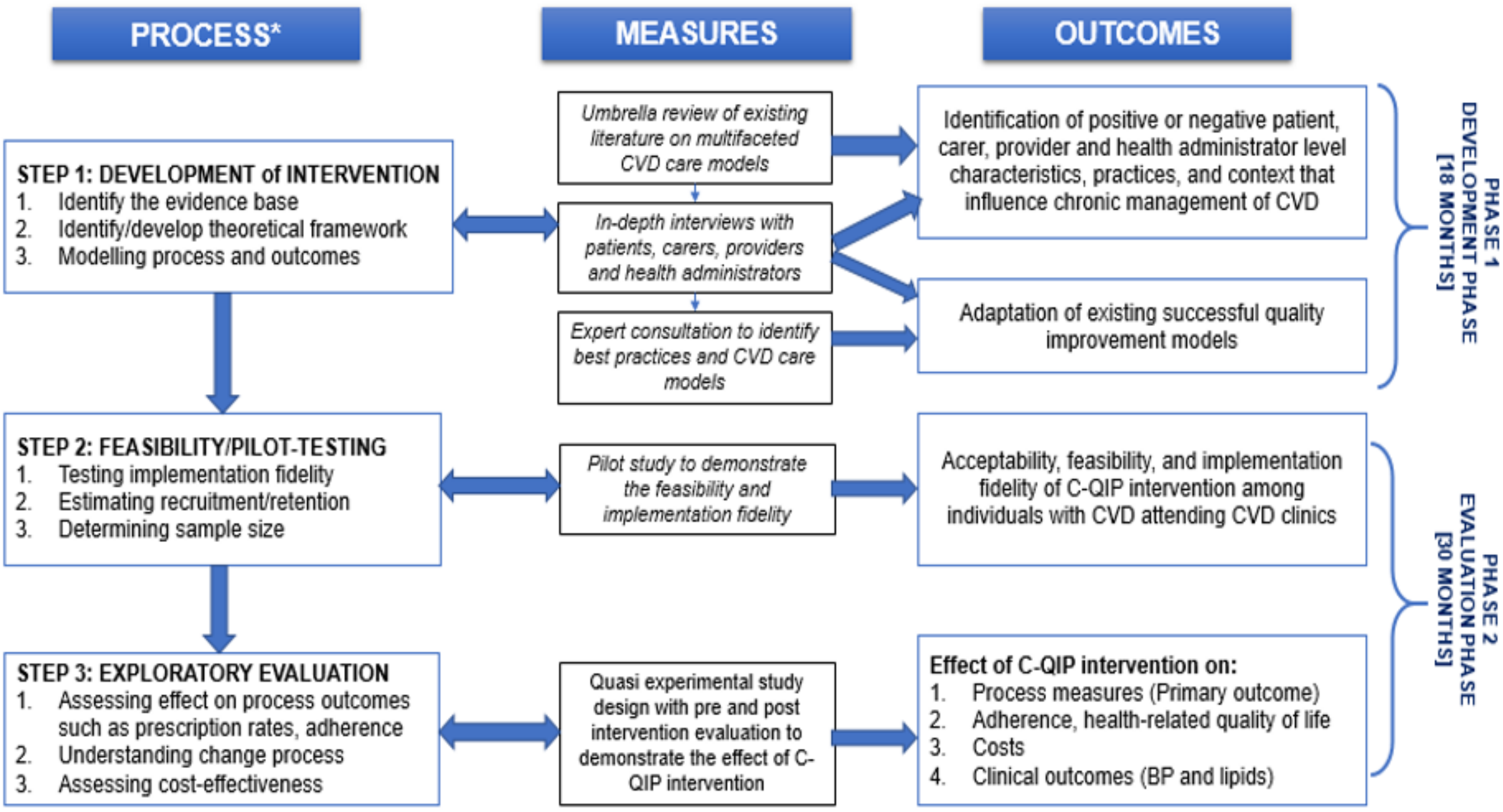

**Development and Evaluation of the C-QIP guided by the CFIR & RE-AIM framework.*

C.2. Specific Aims 1 and 2 [Phase 1: Development phase]

We will use three approaches to inform the development of multifaceted C-QIP strategy for chronic care of patients with CVD: 1) review of evidence, 2) qualitative research, and 3) expert consultation.

C.2.1. Review of evidence

We will perform a scoping review to compile the global evidence from multiple systematic reviews and individual studies of multifaceted collaborative quality improvement models (chronic care model, case management, complex interventions, multidisciplinary teams and self-management and information communication tools (ICT) based care models) to manage CVD into one accessible and usable document. The aim of the scoping review is to summarize the global evidence and identify potentially implementable components of quality improvement strategies for chronic care of CVD. The scoping review will help characterize the quantity and quality of literature by study design and other key features (such as intervention components, effect size, etc.). We will also assess quality of studies within individual studies and of systematic reviews. The scoping review will ultimately allow us to synthesize evidence on what is known, what remains unknown, recommendations for practice, and for future research and leverages the experience of my primary US mentor (Huffman) in evidence synthesis and training, including with the Cochrane Collaboration. The details of our review protocol, including inclusion/exclusion criteria, primary and secondary outcomes, search strategy, data collection/extraction and statistical analysis will be published on PROSPERO.

C.2.2. Qualitative research

The objective of the qualitative research component is to understand the perspectives of four stakeholder groups on the collaborative quality improvement models for secondary prevention of CVD: 1) patients with existing CVD, 2) patients' carers or caregivers, often family members, 3) health providers, including non-physician health workers, and 4) health administrators (coupled with a facility-based evaluation using an adapted World Management Survey based on our team's previous work)[76]. The qualitative research will be conducted concurrent with and informed by the scoping review of existing chronic care disease models because we will seek stakeholders' perspectives on potential interventions identified in the review process. The qualitative research design, data collection, and data analysis will be conducted under the mentorship of Dr. Emily Mendenhall, who is a medical anthropologist and global expert in this methodology.

In-depth interviews (IDIs) among patients, and caregiver: We will conduct 80 IDIs with patients and caregiver, including no fewer than 40 people from each group, with men [18-55 years=20, ≥55 years=20] and women [18- 55 years=20, ≥55 years=20] equally represented[77]. The participants for IDI will be selected from a public and private hospital in Delhi. We expect to glean an understanding of patients' and caregivers' priorities, competing social and economic demands, and cultural perceptions of the appropriateness of various components of collaborative cardiovascular quality improvement care models. No fewer than 20 individuals should be interviewed per group by gender to reach consensus. We will recruit patients with diagnosed conditions (ischemic heart disease, stroke, or heart failure) seeking treatment from the participating outpatient CVD clinics and carers associated with such patients. Patient IDIs will provide fundamental information around patient perceptions of illness and care experiences, including self-care support, challenges, priorities, and needs. We will also ask for a fuller description of how CVD care compares to care for other co-occurring social and health demands (as co-morbidity or multi-morbidity will be a common condition). The carer will be an adult family member of a patient with CVD which s/he identifies as main caregiver. IDIs with carers will further explore competing social and family demands that patients and carers face, challenges of caring and seeking health care for CVD, needs and priorities of the family, and carers' expectations from health care system. My mentoring team has analyzed the microeconomic impact of CVD hospitalization in LMIC using a World Bank developed instrument and found that family members increased their work time (or started new work) to compensate for their family members' disability[37]. Others have found that many patients become non- adherent because financial and social demands are more influential than medical influences[33,78,79].

IDIs with health providers and health administrators: We will conduct 20 IDIs each with health providers (cardiologists, nurses, NPHW), and health administrators (medical officer in-charge) (total=40). Purposive sampling will be used to get diverse sample (e.g., gender, duration of experience, etc.) with varied opinions. The IDIs with health providers and administrators aims to understand the current approaches of chronic management of CVD, challenges, and opinion on incorporating C-QIP intervention in the existing infrastructure and their perspectives on C-QIP intervention feasibility, acceptability, and sustainability. The physicians selected for the interview will be participating in the feasibility trial. Additionally, other cardiologists or practicing physicians will be invited to participate in the qualitative interview to collect diverse views on the treatment practices, barriers, and facilitators to improve care for patients with CVD.

Training and data collection: IDI will be conducted by a trained researcher, with experience in qualitative research and fluent in both English and Hindi. PI will undergo extensive qualitative methods training under Dr. Mendenhall, which will be beneficial to develop a more holistic understanding of competing social and medical demands for implementation science. The IDI guides will be translated to Hindi and back translated into English. All translated instruments will be pilot tested and refined before data collection. We will obtain informed consent and will use IDI guides that are tailored to patients, carers, providers, and administrators. All IDIs will be conducted in the local language, Hindi, or English (based on interviewee's preference), and audio recorded with consent. Each interviewer will write extensive field notes after the conclusion of the interview to ethnographically capture non-verbal and non-recorded communication between the interviewer and interviewee. These field notes will be one level of analysis that will provide a time- sensitive communication tool between interviewer and research team.

Data analysis: After completion of the interviews, team meetings with all interviewers, and a discussion of all perceived emergent themes, PI will lead the development of a code book that will highlight between 30 and 40 primary emergent themes. This codebook will be reviewed by all interviewers and advisors to reach consensus on what themes emerged and how the themes should be defined. These codes will then be systematically applied to all IDIs. We will complete IDIs for patients and carers first, so some themes from these interviews may be reflected in the administrators and providers interviews. After data are transcribed verbatim and translated to English, two coders (including a research assistant and me) will closely read each transcript and apply the codes from the code book. Then we will analyze these codes to understand what the most common responses were and what these responses tell us about CVD experiences and care. Inter-coder agreement will be used to resolve any differences in coding and data interpretation. Our analyses using MAXQDA software will focus on identifying promising, innovative components/interventions, context, barriers, and opportunities for C-QIP initiative in current health care system.

C.2.3. Expert consultation meeting

We will establish a national working group consisting of national experts in public health, health systems, academics (CVD and implementation research), government representative, and clinicians drawing on PHFI/CCDC professional networks. We will present the findings from our evidence synthesis and qualitative research to seek their expert opinion on components of intervention package for chronic care of CVD. Both Dr. Prabhakaran (primary Indian mentor) and Dr. Tandon (secondary-mentor) work closely with the health ministry (government representatives at centre and state-level health system) and policy makers. In addition, Dr. Ambuj Roy and Dr. Sandeep Bhalla (collaborators in this application) have strong networks of health professionals with expertise in CVD management, who will be invited to participate in this expert consultation. We will use the Delphi technique to achieve consensus on formulating an intervention package for chronic care of CVD. We will plan the meeting around scientific conferences and other meetings in which participation from multiple experts is expected. The resulting intervention components will be shared with international experts for their feedback. Based on the experts' feedback, we will refine and finalize the C-QIP strategy.

C.3. Specific Aims 3 and 4: Feasibility trial [Phase 2: Evaluation phase]

C.3.1. Overview

We will perform a pilot study for feasibility assessment and implementation fidelity to determine the reach, dose delivered in 1 CVD clinic, (3-6 month) and a feasibility RCT (24 months) to determine the effect of intervention on process measures (prescription of guideline directed medical therapy (GDMT), adherence to prescribed therapy, blood pressure and lipid control) and cost of delivering the intervention versus usual care in 4 outpatient CVD clinics in Delhi and Karnataka (400 patients [100 per clinic]) with an **average follow-up of 12 months** [**Figure 2**]. We will follow Standards for Reporting Implementation Studies (StaRI) for conduct and reporting[80].

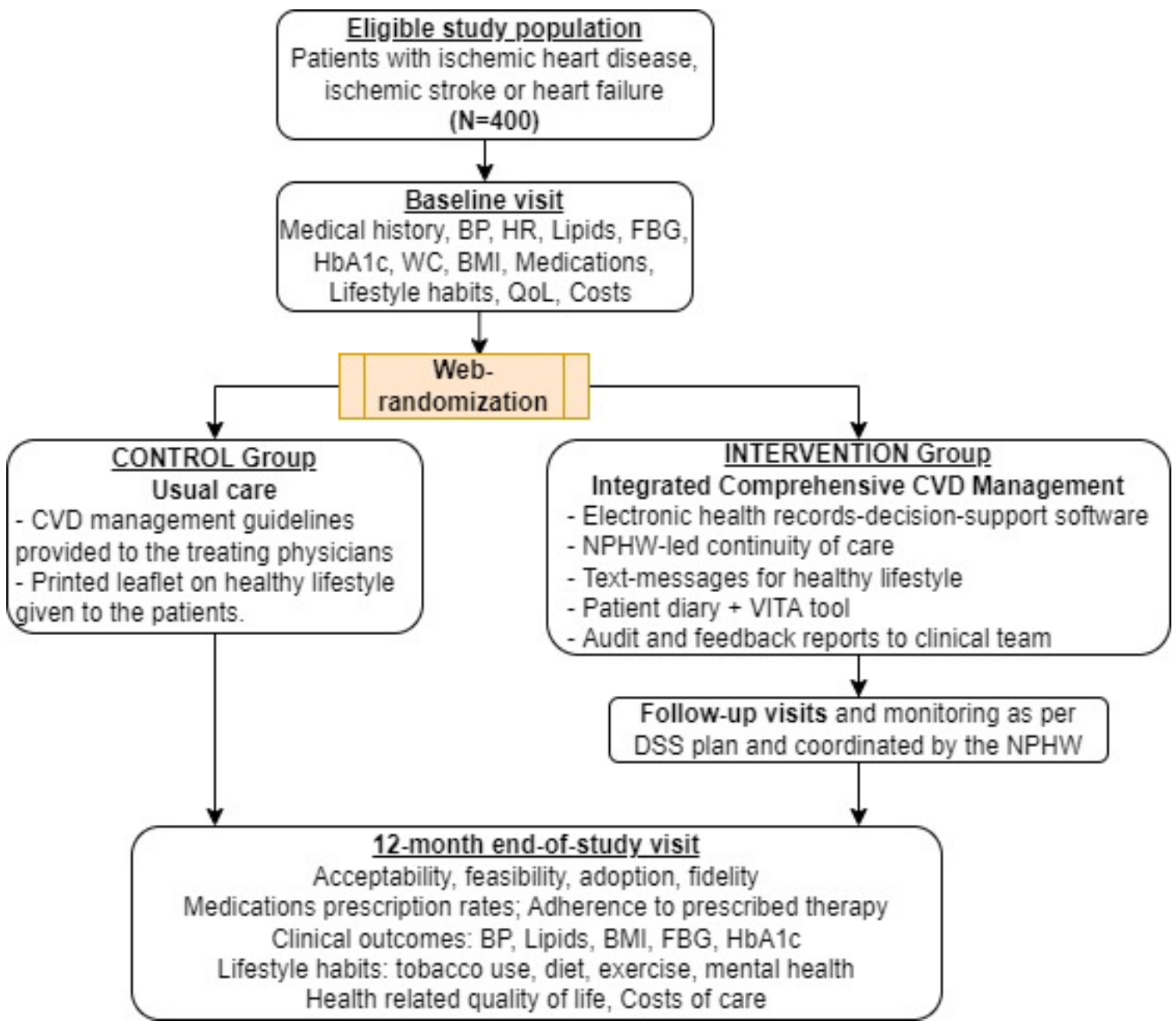

Figure 2. Study design

C.3.2. Study design

The study is a multi-site, individually randomized, controlled parallel group hybrid type 1 implementation study testing a comprehensive, multicomponent collaborative quality improvement strategy (C-QIP) in 400 patients with CVD attending four out-patient clinics in India, for a mean follow-up of 12 months.

A total of 400 patients with established CVD (post myocardial infarction, stroke, or heart failure regardless of ejection fraction) will be enrolled in the study; 100 at each of the 4 out-patient clinics in India. The 100 eligible, consenting participants at each site will be individually randomized to usual care (n=50) OR to the intervention (n=50) which targets processes of care measures (use of evidence-based medications; smoking cessation advice; lifestyle modification advice), adherence to prescribed therapy, and blood pressure and lipids control. The allocation scheme is shown in **Figure 3**.

Figure 3: Allocation Scheme

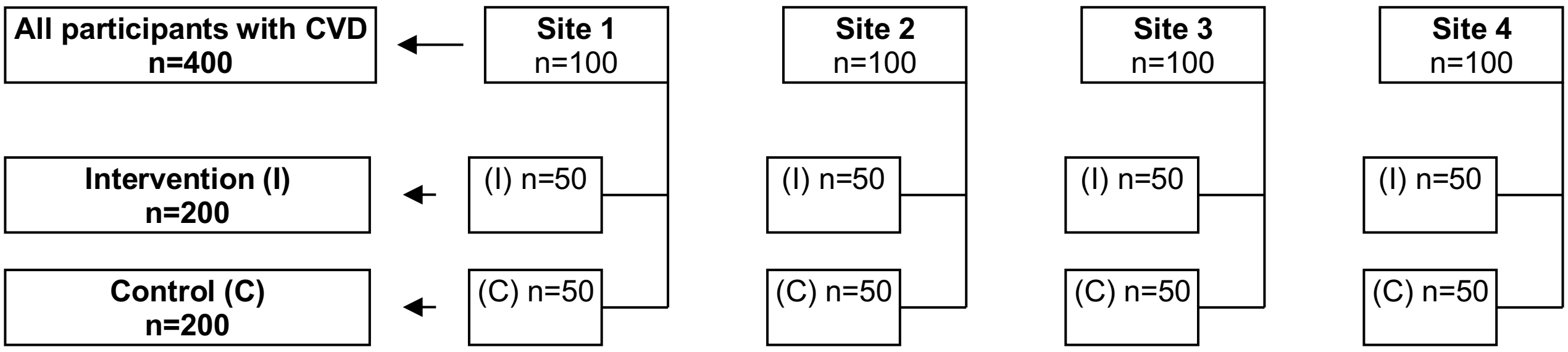

C.3.3. Selection and enrolment of participants

Setting

The proposed feasibility trial will be conducted in north and south India, viz, Delhi and Karnataka. Four hospitals (2 government and 2 private) have been selected (see Participating Sites, pg. 3) through the networks available to study principal investigators. The lead physicians of these sites are considered "Site Principal Investigators" and were participants in the C-QIP expert consultation meeting to engage their involvement/ownership in the trial, discuss trial procedures and coordination, and build team-spirit. Of these sites, one will serve as the site for piloting the decision-support software, and afterwards, piloting the full C-QIP strategy to assess feasibility (screening, recruitment, and retention; continuity of care) to establish procedures, and evaluate and refine multi-site trial coordination. Upon successful completion of this piloting in three months, an additional three sites will join the study.

Patient eligibility criteria

Inclusion criteria:

1. Age: ≥18 years, both sex
2. Having physician diagnosis of or on treatment for cardiovascular disease including ischemic heart disease, ischemic stroke, or heart failure regardless of ejection fraction
3. Living within same city/town
4. Consent to participate.

Exclusion criteria:

1. Pregnant women
2. Patients having diseases associated with frequent hospitalization (advanced cancer, end stage renal disease); bedridden or debilitating conditions and life expectancy <12 months that might limit completion of study follow-up

C.3.4. Methods of recruitment

The recruitment goal is 100 participants at each of the 4 clinic sites for a total sample size of 400 participants.

Researchers at each clinic site will identify participants qualified for the trial from clinical records based on the specified inclusion and exclusion criteria as described above. Medical record searches or reviews of existing hospital databases can be done initially by setting up the searches using the characteristics that match the inclusion/exclusion criteria. Additional "hand searches" may be necessary using the remaining inclusion/exclusion criteria not already part of the existing database, but that may be ascertained through examining the patient's existing clinical record. It is likely that all or most of the inclusion/ exclusion criteria will be available in most medical records. Further, to avoid any potential lost-to-follow-up, physicians will be encouraged to recruit local patients (within city) to improve compliance with the follow-up visits. Further, as we have proposed SMS text messages as one of the intervention components, so it would be preferred if patient has access to a mobile phone and can read the messages. However, we will also allow if a patient's caregiver (someone who is directly involved in care

of the patient at home) has access to mobile phone and can read the text messages sent by the non-physician health worker (NPHW) on lifestyle advise, medication adherence, and for the next clinic/lab appointments.

Potential participants will be contacted by telephone or approached in-person at the clinic to determine their interest in the study. They will be briefly told about the study, and if interested, they will be invited to the clinic site for informed consent and screening. If the consenting participant meets eligibility criteria after screening, he/she will be enrolled in the trial (scheduled for complete baseline testing, undergo randomization, and continue with follow-up).

C.3.5. Informed consent

At the first screening visit, the Site Investigator/Co-Investigator will obtain written, informed consent from all participants who are eligible to participate in the trial. A single participant information sheet and consent form will be used for all the procedures done as part of the screening, baseline assessment, and follow-up. We anticipate that most participants will be literate in their local language and/or English; translated consent forms will be made available in the local languages of all participating sites. See ***Appendix 1*** for the combined **participant information sheet (PIS) and informed consent** form.

The information sheet and consent form will be given to the potential participant and consent details will be explained, including purpose of the trial, screening and study procedures, benefits and risks, confidentiality terms, and rights of the participant, and trial contact information. The Site Investigator/Co-Investigator obtaining consent will ask questions to the participant about his/her understanding of what was explained to ensure that the participant correctly understands the PIS-consent information. The individual will be given the opportunity to review the document and any questions or concerns will be addressed. Participation is purely voluntary with no coercion and no material compensation. All participants will be assured that they have the right to voluntarily withdraw from the study at any time, without any repercussions by way of this action affecting their future medical care.

If the individual agrees to participate in the trial, then two copies of a signed consent form will be made, with the signatures of the participant and the site investigator. For those who cannot read, the details of the study will be explained to the participant and informed consent will be obtained by the participant's thumbprint and proxy signature by a legally acceptable representative (LAR). In the absence of a LAR, a literate third party (non-study staff) may act as witness. One copy of the consent form will be given to the participant to keep, and another copy will be kept in locked storage at each Clinical Site.

These consent procedures will be reviewed and approved by each clinical site's local ethics committee as well as the PHFI Institutional Ethics Committee. The study coordination center based at PHFI will ensure proper collection and storage of the consent forms.

C.3.6. Screening and Randomization

After informed consent has been obtained at the screening visit, the consented individual will undergo initial screening which will include basic medical history (inclusion/exclusion criteria). A screening log will be kept by each participating site to document any reasons for ineligibility and for non-participation of eligible candidates. Those participants who meet the eligibility criteria will be enrolled in the trial and continue with a further baseline assessment (including a thorough history and physical exam, anthropometric measurements, health-related quality of life, processes of care and cost of care questionnaires) and randomization.

Randomization

Restricted randomization of variable blocks will be used for the trial, stratified by the 4 clinic sites. The block allocation sequence for each site (n=100) will be computer-generated centrally (at the Research Coordinating Centre, RCC, at PHFI), allocating 50 participants to the intervention and 50 participants to usual care. The allocation sequences for each of the 4 sites will be kept in sealed envelopes, stored in a locked space at the RCC at PHFI accessible only to members of the RCC handling trial allocation.

The randomization will be provided centrally using an interactive web response system (IWRS). Once a consented, eligible participant has been confirmed at the site, the study physician or a designated site coordinator will visit the trial website on the internet and supply the inclusion/exclusion information and will receive a randomization allocation immediately. If the IWRS is not functioning for any reason (malfunction, electricity blackout, etc.), the site should call the RCC at PHFI for the allocation, which the RCC member overseeing the trial randomization will provide over the phone and by email/fax to the site

team. The site will be required to send an email/fax confirmation to the RCC of the participant's allocation. Participants will be told at the randomization visit which arm of the trial they have been allocated to.

Concealment of intervention versus standard care allocation to the participants is not possible, due to the transparency of the type of care the patients will receive.

C.3.7. Study intervention

During the face-to-face virtual expert consultation meeting held in November 2020, the experts narrowed down the QI interventions to five main strategies for evaluation in the C-QIP trial: 1) Electronic health records – decision support systems (EHR-DSS) for the providers, 2) non-physician health workers (NPHW) facilitated care for patients with CVD, 3) text-message based reminders for patients to promote healthy lifestyle, and regular clinic visit attendance and laboratory testing, 4) Patient diary with visual assessment tool for adherence to medications and reinforcement tool for lifestyle modifications, and, 5) Quarterly audit and feedback reports for care providers.

The intervention will involve training of physicians (both specialist doctors and internists, nurses, and counsellors), use of non-physician health workers (to support patient self -management and follow up, navigation of clinical services and treatment coordination) and patient record system (aiding shared decision making between physician and patients).

Each participating site will have eligible participants individually randomized to either the intervention or control group. The *intervention group* will receive multicomponent collaborative quality improvement (C-QIP) strategy through comprehensive CVD care supported by an EHR-DSS, NPHW, text-messages, patient diary and quarterly audit and feedback reports. The *control group* will receive usual care provided at the participating Site. Physicians treating both groups will be provided with evidence-based management guidelines to ensure fair access to current recommended clinical practices. Refer to **Figure 4** for a summary of the trial groups.

Figure 4: Schematic representation of care received by intervention and control groups.

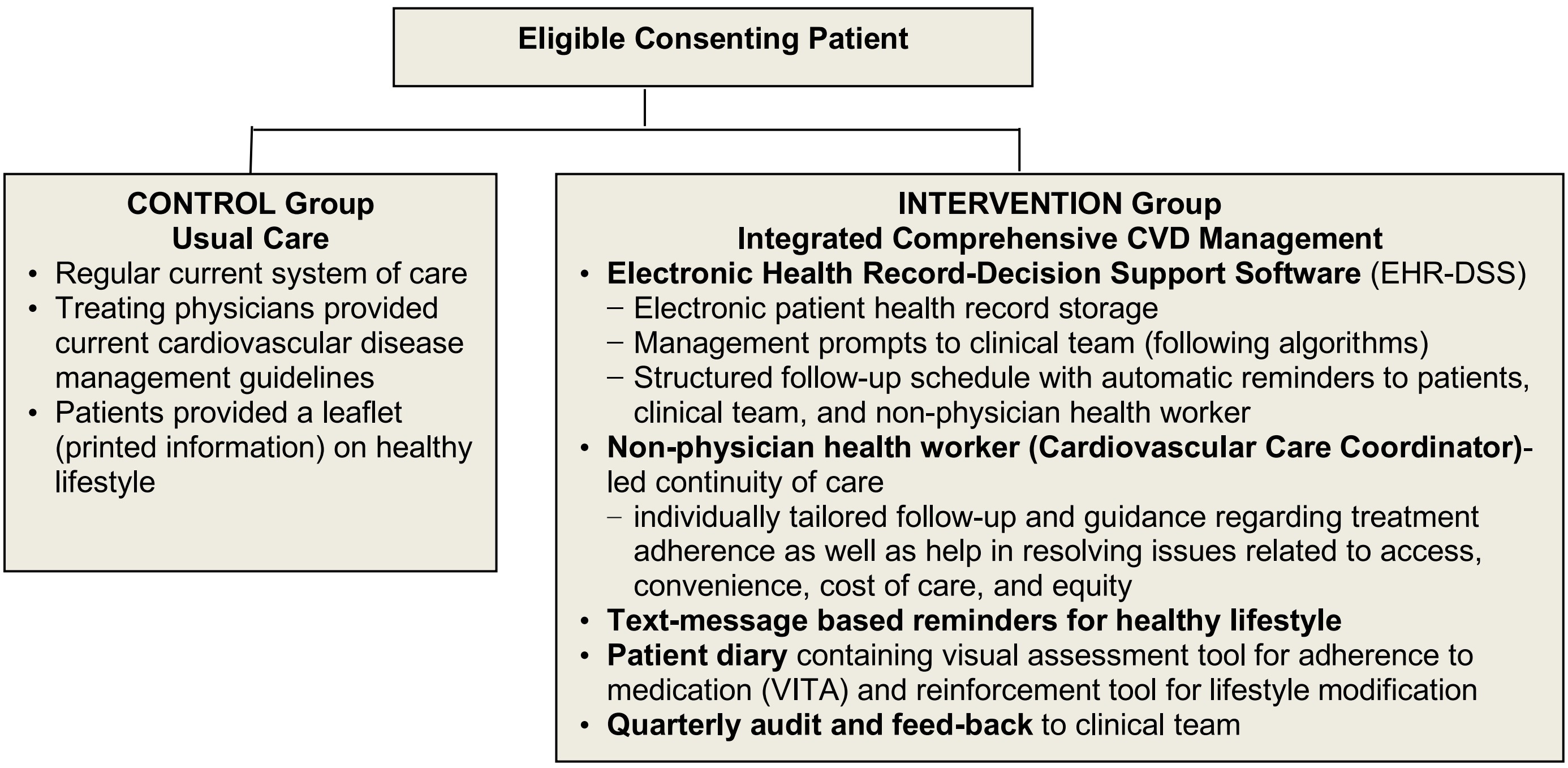

Participants randomized to the intervention group will receive targeted cardiovascular disease management through 5 main strategies: 1) an electronic health records- decision-support software (DSS) based on most recent evidence based CVD management guidelines (described below), 2) support from a non-physician health worker (NPHW) who manages the DSS and ensure patient follow-up according to the guidelines, 3) Text-messages for healthy lifestyle, 4) Patient diary containing visual

assessment tool for adherence to medication (VITA), and reinforcement tool for lifestyle modifications, 5) Audit and feedback reports to the care providers (**Figure 5**).

The CVD management algorithm-guidelines **(*Appendix 3*)** for individuals with CVD serve as the basis of the intervention's strategies. The evidence-based guidelines provide the clinical team with the up-to-date instruction for intensive CVD risk factor control, targeting regulation of blood pressure, and lipid control, smoking cessation, and processes of care (aspirin use; ACE-inhibitor use; Beta-blocker user, statin use). The guidelines follow an algorithm-format and provide progressive recommendations from simple lifestyle management to intensified treatment regimens and referral, according to the participant's status. The algorithm-guidelines have been developed by a multi-disciplinary team, led by study principal investigators. The guidelines follow current internationally accepted evidence, with adaptations to increase relevance to the Indian population. Feedback from the site investigators has been incorporated into the guidelines.

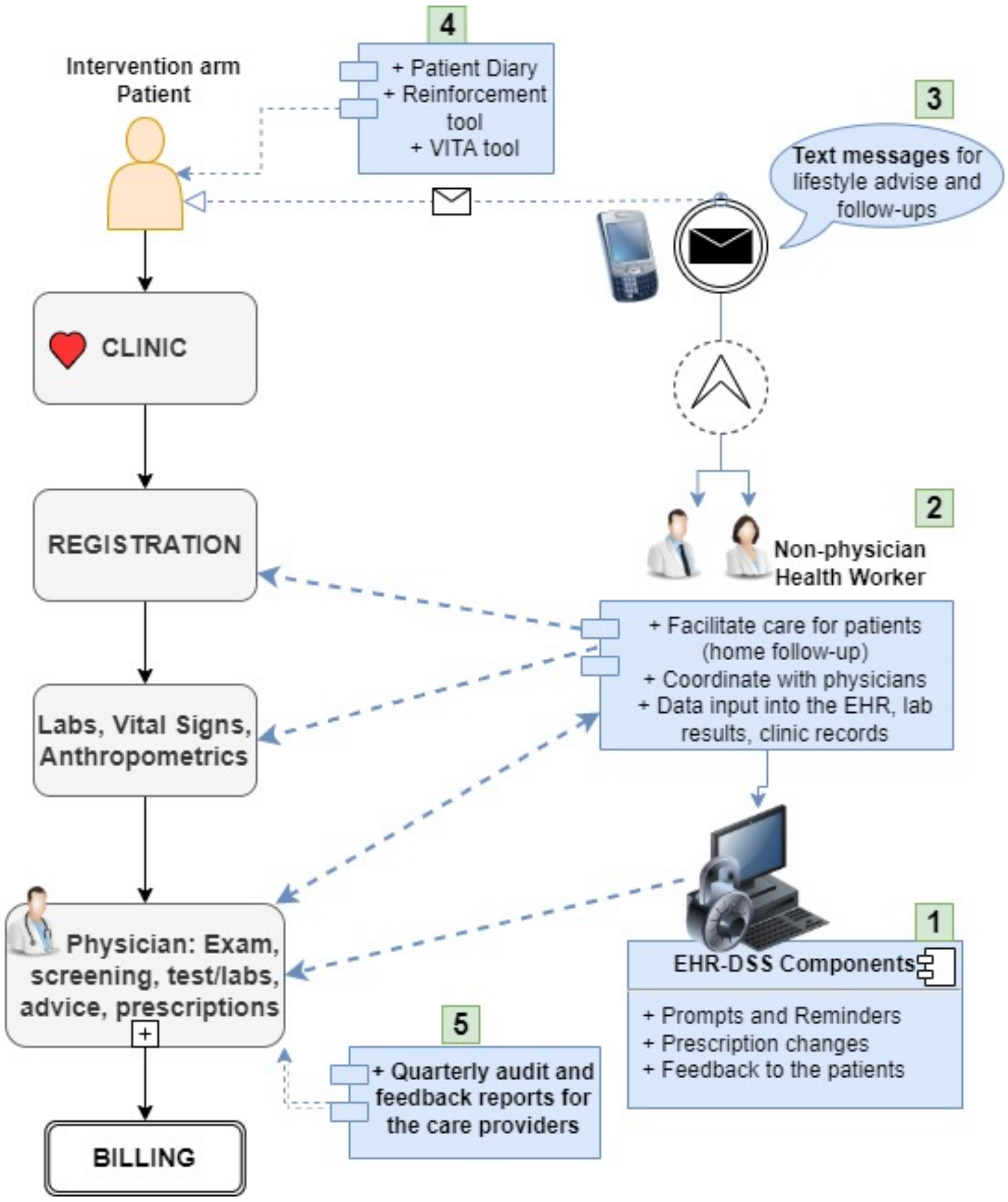

Figure 5. Intervention flow

Decision-Support Software
A simple decision-support software (DSS) will be setup at all clinic sites. The DSS is currently being designed by collaborating programmers based in Hyderabad who have a background in creating decision support electronic health records.

The web enhanced DSS will:
(a) store all electronic health records *(EHR component)* and patient habits, integrating all laboratory and consultation reports in one easily accessible location.
(b) provide automated decision-support prompts of guideline-recommended processes of care (e.g., treatment plan, laboratory tests, screenings) tailored to the participant's compliance and CVD risk level; and
(c) send out automatic reminders and alerts to patients, the clinical team, and NPHW to ensure adherence to the guidelines/ care plan.
(d) serve as a trial data collection and management portal of participant progress and their clinical activities in terms of prescriptions, intensification of therapy, advice given, etc.

The DSS will be managed by the **non-physician health worker (NPHW)** (see below), who will be responsible for enforcing its recommendations at each Clinic Site. Overall, the DSS will organize case management and will assist the NPHW in following-up with patient care.

Non-physician health worker
Recruitment: One non-physician health worker (NPHW) will be designated for each Clinic Site for the duration of the trial to facilitate care and follow-up of intervention group patients. Each NPHW will be recruited locally by the Site Investigator. The study will provide a competitive salary for the NPHW. The criteria for NPHW recruitment are as follows:

1. Registered nurse, social worker, or dietician OR community health worker with 2 years of health service experience
2. Strong inter-personal, motivational, and organizational skills
3. Willingness to stay for the full duration of the trial (2 years).
4. Basic computer application knowledge

Responsibilities: Each NPHW will be managing all 50 participants randomized to the intervention. The work of the NPHW will be a combination of interactions with the clinical team and personalized follow-up care with participants. The algorithm-based CVD management guidelines via the DSS recommendations will be enforced by the NPHW, complementing the role of physicians. The NPHW will NOT be allowed to make prescriptive changes for a participant.

Below are the expected functions of the NPHW:

1. Fully manage the DSS, from data-entry of participant information/progress to communication of DSS management prompts to the clinical team.
2. Assist the physician to devise a patient-tailored follow-up plan of participants based on their compliance and motivation and other CVD risk factors. Patients who are not compliant or at high-risk will require more intensive follow-up (e.g., home-visits and monitoring; motivation to improve lifestyle, treatment adherence etc.) while more compliant participants might require less intensive follow-up (group visits at the clinic with information sessions).
3. Facilitate follow-up, referral, treatment, and investigation appointments including reminders to enforce participant's care plan (achieving target BP, lipid, and glucose control; use of aspirin and RAS-modifier therapies; lifestyle modification and smoking cessation).
4. Encourage and motivate participants to better self-manage risk factors (by providing the appropriate guidance and tools), promote better lifestyle choices (physical activity, diet, smoking cessation) and treatment adherence.
5. Prompt earlier attention of patient's needs/progress to the treating physician (e.g., eliciting a prescription without waiting for the patient's next clinic appointment).
6. Arrange regular meetings with the clinical team to review patient progress, address patient needs, and other trial issues.
7. Resolve issues of access, convenience, and cost of care, and equity; and
8. Manage the intervention group's trial data collection, documentation, and communication with the Research Coordinating Centre (RCC).

Training of NPHW will occur in two stages prior to the start of the trial. A standardized instruction course for the full group of hired NPHW will be held at a selected training center in India, followed by individual training at each Clinic Site to contextualize duties in setting and with the clinical team.

The work of the NPHW will be routinely monitored by the RCC to document their progress and quality of care delivered by the physician- NPHW team. Routine feedback will be provided to NPHW to improve areas of weakness by the Site Investigator. Formal evaluation of NPHW will occur at selected testing time points during the trial (See ***Section C.3.16 Quality Assurance***).

Text-messages for healthy lifestyle
Intervention group participants will receive text-messages for lifestyle modifications, to motivate patients to adhere to prescribed therapy and reminders for the next clinic visit or lab tests. Frequency of text-

messages will be tailored according to the patient's compliance to the lifestyle habits, medication adherence, and scheduled clinic visits, or lab appointments.

Patient diary
Patient daily will contain visual assessment tool for medication adherence and reinforcement tool for lifestyle modification will provided to each intervention group participants to encourage them to set goals for risk factor targets (e.g., weight management, blood pressure, blood cholesterol and blood glucose) and to monitor clinical signs and symptoms of chest pain, neurological problems, swelling of feet. NPHW will provide necessary training to the intervention group participants to use the patient diary effectively, document the goals and track these on monthly basis.

Quarterly Audit and feedback report
Physicians will receive an audit and feedback report on quarterly basis to demonstrate the performance of their clinic site in terms of managing intervention group participants against the benchmark set by the American College of Cardiology to effectively manage patients with established CVD and for secondary prevention of CVD. The audit and feedback report would include a summary of patients recruited in the intervention group, summary estimates of the proportion of patients receiving aspirin, 2 blood pressure lowering medicines, and statins, and clinical measures such as mean blood pressure, total cholesterol, triglycerides, and fasting blood glucose. Lifestyle habits of the intervention group patients will also be summarized, e.g., proportions smoking, and non-adherent to recommended diet plan, or exercise.

C.3.8. Control arm (Usual care)
Currently, no such multicomponent C-QIP strategy is available in India for people with CVD. The control group participants will receive a leaflet (printed information) on health lifestyle to prevent recurrent CVD events. This will be delivered by a different team member (i.e., not by NPHW) to avoid contamination. This provision would ensure that the control group participants feel that there are benefits to participation (hence, lower attrition). As mentioned above, the physicians treating the control arm will also be provided with the CVD management guidelines to ensure fair access to current recommended clinical practices. Either a clinic staff or a hired **research assistant** will manage the control group's study-related visits and data collection – separate from the duties of the NPHW with the intervention group.

Responsibilities: The research assistant at each clinic site will be recording data collected on the 50 participants randomized to the control group into the electronic health record system.

The following are the expected functions of the research assistant:
- Assist with baseline assessment of participants.
- Collect study-visit and any non-study visit data of the control group participants using paper Case Report forms (CRFs). Data collection during non-study visits will be done AFTER the physician sees the participant (uni-directional flow of information – he/she will NOT feedback anything additional about the participant to the physician and vice-versa)
- Will have access to only the electronic health records aspect of the DSS (only the EHR, no decision-support) for his/her CONTROL arm patients only for data entry on a regular basis (once every 2 weeks)
- Must not advise participants or clinical staff member in any way
- Must NOT follow-up with participants between the baseline and annual study visits
- Must NOT arrange appointments or laboratory tests for participants other than the required annual study-visit
- Must NOT substitute for NPHW when he/she is on leave

Like the NPHW, the research assistant will be trained at the site initiation visit with the clinic team.

Like the NPHW, the work of the research assistant will be routinely monitored by the RCC to ensure proper data collection. The Site Investigator is also responsible for overseeing the Research Assistant's performance.

C.3.9. Study procedures

Schedule of evaluations

The first visit is for participant screening and randomization. Next visit is for baseline assessment. Guideline-recommended follow-up for all patients with CVD is on 3-monthly basis and more frequently if the participant has poorly controlled blood pressure, lipids, or other CVD risk factors. The intervention and control groups will have annual follow-up study visits to capture study related outcomes. All other intermediate visits by the participants will be documented.

For all randomized participants, the average post-randomization follow-up period will be 12 months. All follow-up 12 monthly visits must occur within ± 2 weeks of the scheduled follow-up visit date. If the visit happens outside this window, the Site Investigator should proceed with the visit but indicate a note in the file stating ‘protocol deviation.’

The study staff at each Clinical Site involved in executing the study procedures includes:

- **Site investigator/co-investigator**
  - Oversee all trial-related procedures (recruitment, screening, enrollment, randomization, and follow-up of participants)
- **Study physician (includes Site Investigator/co-investigator)**
  - Perform screening evaluations, baseline history and physical examination and conducts randomization of participant into trial.
  - If seeing an intervention participant, work as a team with the NPHW to manage the participant’s care
- **Non-physician health worker**
  - Data collection for intervention group patients on paper CRFs
  - Coordinate with site-investigator/co-investigator and study physician(s) to complete all follow-up visit requirements; ensure complete data collection for intervention group
  - Enter intervention group’s data into the eCRFs of the DSS
  - Supply CVD Management Plan (**CRF 4**) to study physician to finalize care plan at each visit
  - Other details below
- **Research assistant**
  - Data collection for control group on paper CRFs, minimal interaction during follow-up to prevent any change in the standard care
  - Enter control group’s data into the eCRFs of the DSS
  - Other details below

*All **case report forms (CRFs)** are provided in sequential order in the final appendix (**Appendix 5**) and included as separate documents in the electronic version.*

Schedule of evaluations (green-highlighted columns are study data-collection visits) (**Table 2**)

Table 2. Schedule of evaluations in the C-QIP study.

Evaluations/Visits	Screening	Baseline	Intermediate	12-month Follow-up	End of study
Signed informed consent	x				
Assess Eligibility	x				
Enrolment/Randomization	x				
Demographics		x			
Medical history		x			
Family history		x			
Social History (lifestyle/habits)		x	x	x	x
Self-care questions		x		x	x
Quality of Life, short (Global Health Scale))		x		x	x
Treatment Satisfaction questions		x		x	x
Processes of care questions		x		x	x
Costs of care questions		x		x	x
Acceptability: providers and patient interviews		Sub-sample		Sub-sample	Sub-sample
Blood pressure and heart rate		x	x	x	x
Height		x		x	x
Weight, BMI (calculated), Waist Circumference		x		x	x
General Physical Examination		x		x	x
Serum Creatinine		x		x	x
Serum Sodium, Potassium and Liver enzymes		x		x	x
Fasting blood glucose		x	x	x	x
Glycated haemoglobin A1c (HbA1c)		x	x	x	x
Lipids (TC, HDL, LDL, TG)		x	x	x	x
Medications: antiplatelets, blood pressure lowering, and cholesterol lowering medications		x	x	x	x
Serious adverse event reporting		x	x	x	x

BMI=body mass index; TC=total cholesterol, HDL=high density lipoprotein cholesterol, LDL=low density lipoprotein cholesterol, TG=triglycerides

Baseline assessment visit
After screening visit has been completed, the eligible patient will undergo the baseline assessment and randomization by study physician. This can occur the same day when screening is completed. After randomization the study physician will introduce the control arm participant to the research assistant and intervention arm participant to the non-physician health worker (NPHW).

For **control arm** (usual care) participants, the **research assistant** will:

- Complete **CRF 2 – Baseline assessment** which includes: demographics, social/lifestyle habits, and questionnaires (self-care, quality of life, treatment satisfaction, and frequency/costs of care).
- Advise the participant that his/her cardiovascular disease care will remain unchanged at present; he/she will be expected to see the usual physician at the frequency he/she advises. He/she must be available to return once a year for a study-related visit.
- Enroll the patient for the trial in the study electronic database and complete the eCRFs.

For **intervention arm** participants, the **NPHW** will:

- Complete **CRF 2 – Baseline assessment** which includes: demographics, social/lifestyle habits, and questionnaires (self-care, quality of life, treatment satisfaction, and frequency/costs of care).
- Advise the participant that their CVD care will be managed using the C-QIP strategy, and he/she will have regular follow-up from the NPHW and be asked to visit the clinic at least every 3 months. Enroll the patient for the trial in the DSS and complete the eCRFs.
- The NPHW will facilitate the participant's adherence to appointments and self-management using the DSS-generated reminders and phone calls.

Qualitative Assessment: A maximum of 3 study physicians (mainly Site Investigator/ Co-Investigators) at each clinic site will be consented and interviewed at the site initiation visit by RCC study team to have an initial understanding of their views on the intervention. These interviews of not more than 30 minutes will be conducted following interview topic guide.

Follow-up visit
All participants (intervention and control) will present for the 12-monthly follow-up visits. All participants will have to obtain all investigations: venous FBG, HbA1c, lipid profile, serum creatinine and other blood biochemistries (serum sodium, potassium, ALT) if necessary, prior to the visit. All participants will be required to present the lab results at the visit, if the Clinic Site does not have a system in place to obtain the laboratory results independently.

- The control arm will follow the usual process for setting up laboratory appointments.
- For the intervention arm, one week prior to the visit, the NPHW will ensure that the intervention participants have obtained the laboratory tests for the visit.

Prior to the physician assessment, the **NPHW/research assistant** will obtain the following data from the participants for **CRF 5**:

- Interval history, other complaints, and serious adverse events
- Measures: Blood pressure and heart rate, height, weight, BMI [calculated], waist circumference
- Investigations: values from lab reports.
- Social/lifestyle habits and questionnaires: self-care, quality of life [EQ5D], treatment satisfaction, and frequency/costs of care

For the **intervention arm:**

- The **NPHW** will input the specified data from **CRF 5** into the DSS and print out an **CVD Management Plan (CRF 4)** and provide this to the physician during consultation.
- The **study physician** will review **CVD Management Plan** (**CRF 4**) and accept/reject the management prompts, update medications and the patient management plan; fix next visit date and review the plan with the NPHW.
- The NPHW will update the patient; address any barriers to care and motivate him/her to achieve risk factor management goals and confirm the next appointment dates. The NPHW will input the updated **CRF 4** and rest of **CRF 5** in the DSS.
- The NPHW will facilitate the participant's adherence to appointments and self-management using the DSS-generated reminders and phone calls.

Qualitative Assessment: The same sample of interviewed physicians at each site will be interviewed during the following 2 annual monitoring visits by the RCC study team to assess changes in practice and feedback on the intervention. The interviews of maximum 30 minutes will be conducted by a trained research staff using written guides.

Intermediate visits
The research assistant and the NPHW will need to document all intermediate visits for the control and the intervention arm participants, respectively.

For the **control arm:**

- The control participant's file will be tagged, so that whenever the patient returns for a visit, a clinic staff should notify the research assistant that the patient has arrived.
- After the patient's visit is complete, the research assistant will need to fill out **CRF 3** only by looking at the patient's file.
- The research assistant will input **CRF 3** in the EHR.
- The research assistant does NOT contact the control patient to schedule appointments or ensure regular follow-up. The follow-up routine will continue as per existing care at the clinic.

For the **intervention arm:**

- For all other clinic visits outside the 12-monthly visits, the NPHW ensures patient appointments, documentation, and follow-up of all intermediate visits.
- One week prior to the visit, the NPHW will ensure that the participant has obtained the following laboratory tests for the visit: any lab tests that the physician had specified at the previous visit.
- Laboratory reports will be collected on the day of the visit by the NPHW.
- At the visit, the NPHW will complete **CRF 3** with the following:

    - Interval history, hypoglycemic events, adverse events, self-care
    - Measures: blood pressure, heart rate, weight, BMI (calculated with baseline/annual height) and waist circumference
    - Investigations: values from lab reports (venous fasting blood glucose and other tests ordered).
- The NPHW will input **CRF 3** into the DSS and print out an **CVD Management Plan (CRF 4)**. The NPHW will update the patient's medications in **CRF 4** and provide this to the physician during consultation.
- The study physician will review **CRF 4** and accept/reject the management prompts, update medications and the patient management plan; fix next visit date and review the plan with the NPHW.
- The NPHW will update the patient; address any barriers to care and motivate him/her to achieve risk factor management goals and confirm the next appointment dates. The NPHW will input the updated **CRF 4** in the DSS.
- The NPHW will facilitate the participant's adherence to appointments and self-management using the DSS-generated reminders and phone calls.

End of study visit
The end of study visit process is the same as the 12-monthly visit and will be completed using the **CRF 5 (same as annual visit form)**.
Scheduling:

(1) If an annual visit for a participant is scheduled for within 3 months from the last date of the trial (December 31, 2023), then this annual visit becomes the end of study visit.
(2) If the last annual visit for a participant is/was scheduled for more than 3 months from the last date of the trial (December 31, 2023), then conduct another visit designated as end of study visit as per the last trial date. For an intervention patient, a scheduled intermediate visit within 3 months from the last trial date will serve as the end of study visit.

Final Qualitative Assessment: The physicians interviewed previously will undergo a final interview with the RCC team at the trial closing visit for feedback on the intervention and changes in their clinical practice. In addition, NPHW and trial participants from both treatment groups will be invited to participate in an in-depth interview to understand their experience in the trial and of the C-QIP strategy. These interviews of maximum 30 minutes will be conducted by a trained research staff using written guides.

C.3.10. Safety Assessments
In this C-QIP study, we are not implementing any new drug or invasive procedure that requires specific monitoring of safety parameters. Rather, the intervention is a quality improvement strategy that is enforcing existing, evidence-based guidelines for CVD management. The control group will follow the existing usual care provided at the clinic site, while the intervention is designed to track quality of care delivery and achieve risk factor control (via regular examination of blood pressure, heart rate, blood cholesterol, fasting blood glucose, BMI, and prescription of guideline directed medical therapy). Adverse events and serious adverse events will be monitored.

Information about the occurrence of any serious adverse event will be sought at all scheduled visits, and outside of scheduled visits by participant self-report and tracking of non-study related visits. Patients will have access to the doctor at the participating hospital site for remedial measures in the event of side-effects due to anti-hypertensive/ aspirin/statin medications. The medications suggested are in general the drugs that are in common use and recommended for the secondary prevention of CVD by national and international guidelines for treatment and management of CVD.

Adverse events
Definition of an Adverse Event: "An adverse event (AE) is any untoward medical occurrence in a subject (study participant) temporally associated with participation in the clinical study or with use of the experimental agent being studied. An adverse finding can include a sign, symptom, abnormal assessment (laboratory test value, vital signs, electrocardiogram finding, etc.), or any combination of these."

Definition of a Serious Adverse Event: "A serious adverse event (SAE) is any adverse event (any untoward medical occurrence in a subject/study participant temporally associated with participation in

the clinical study or with use of the experimental agent being studied) that results in one or more of the following outcomes: death; a life threatening event; inpatient hospitalization or prolongation of existing hospitalization; a persistent or significant disability/incapacity; a congenital anomaly or birth defect; or an important medical event based upon appropriate medical judgment."

List of Serious Adverse Events which will be reported in the study are:

1. CVD events: Angina, non-fatal myocardial infarction (MI) / Unstable Angina, revascularization procedure [angioplasty or coronary artery bypass surgery (CABG)], Transient Ischemic Attack (TIA), Stroke (non-fatal), Arrhythmia
2. Renal: end-stage renal disease requiring renal replacement therapy (dialysis or transplantation)
3. Death
4. Any other major health conditions or hospitalization for more than 24 hours (important medical event based upon appropriate medical judgment)

Expected Risks

We judge the potential risks from data collection to be minimal. No invasive procedures are under consideration, except for collection of venous blood samples; these procedures are minimally invasive. However, there is a risk of bruising or discomfort with blood draws and, very rarely, the procedure can result in inflammation or infection of the arm veins. It is also unlikely, although possible that trial instrument questions may cause distress to the participant. Risks are considered minimal, and participants will be informed of all possible risks in the consent form. If the participant deems the risks too large, he or she may refuse to take part in the study or withdraw at any time.

Measures to minimize risk

Even though the potential for injury to study participants due to risks of the proposed procedures is judged to be minimal, all reasonable efforts will be made to minimize these risks through the exclusive use of properly trained and educated research personnel.

The following steps will be taken to minimize the risks:

1) To minimize risks associated with blood draws, the study team will include experienced and trained phlebotomists.
2) Participants will be informed of their right to refuse to answer any survey question that makes them uncomfortable.
3) Any undiagnosed disease or condition or abnormal test results that present during the study testing will be brought to the attention of the participant.
4) Standard drug regimens for management of cardiovascular diseases (diuretics; calcium channel blockers; ACE inhibitors, aspirin and statins) will be used as defined in the national program, for managing CVD.
5) Patients with concurrent depression/anxiety or alcohol use disorders will be managed according to WHO guidelines.
6) Optimal care for CVD has been defined in the Government of India's manual for Medical Officers and care received by patients will be assessed against these explicit criteria.
7) The topics discussed during the qualitative research process will not cover any sensitive issues. Qualitative data collection will be undertaken by trained and qualified research staff.
8) Participants in the intervention arm will receive SMS alerts as reminders for their scheduled follow up visits and healthy lifestyle practices (to improve self-care). These reminders will not disclose any details regarding their diagnoses or treatment.
9) Information about the occurrence of any adverse event or SAE will be sought at all scheduled visits, and outside of scheduled visits by participant self-report and tracking of non-study related visits. All SAEs reported in the study will be duly notified to the overseeing Institutional Ethics Committee of Public Health Foundation of India within 24 hours of notification of the event by the study PI.

Further, the data safety and monitoring board (DSMB) will convene annually to review the report and other issues pertaining to the trial's progress and safety. The annual report will be signed by the DSMB chairperson and forwarded to the PHFI IEC and the C-QIP study Investigators. Minutes of the DSMB recommendations will be provided to funding agency along with the annual study progress report.

C.3.11. Study outcomes

Primary outcomes: Implementation outcomes

1. *Acceptability*: Extent to which providers and patients perceive C-QIP strategy to be agreeable, or satisfactory.
2. *Adoption*: Uptake, utilization, initial implementation, or intention to try using multicomponent C-QIP strategy by providers, NPHW, and patients at participating sites.
3. *Appropriateness*: Perceived fit, relevance, or compatibility of the C-QIP at the four participating sites (2 government and 2 private hospitals), as well as the usefulness and practicability of C-QIP strategy from the perspectives of the care providers, and patients for chronic care of CVD.
4. *Cost*: Financial impact of implementing C-QIP strategy including marginal cost of treatment delivery in the intervention versus usual care group, cost of the implementation strategy including the non-physician health workers, text-messages, patient-diary, electronic health records and decision-support system and audit and feedback reports.
5. *Feasibility*: Extent to which C-QIP strategy can be successfully used or carried out at the four participating sites (i.e., dose delivered and dose received). Number of clinical decision support prompts reviewed, accepted, or rejected to make changes in the treatment plan, no. of clinic visits, no, of lab tests performed in the intervention group patients, number of interactions between the patient and non-physician health workers, no. of text-messages sent to the patients.
6. *Fidelity*: Degree to which C-QIP strategy was delivered as prescribed in the study protocol or as intended by the study investigators. Fidelity will be determined by objectively measuring adherence to treatment protocol using questionnaire and qualitative interviews with providers, and patients to explore the dose received and dose delivered, i.e., use of clinical decision support prompts by the providers, text-messages sent/received/actioned by the patients.

Secondary outcomes: Processes of care outcomes

1. Prescription rate of guideline directed medical therapy (GDMT) by providers
2. Patient's adherence rate to GDMT

Exploratory clinical outcomes: Between group differences in:

1. Mean changes in blood pressure at trial end
2. Mean changes in LDL cholesterol at trial end
3. Mean change in health-related quality of life
4. Incremental cost of care
5. Adherence to recommended lifestyle habits: smoking cessation, diet, physical activity, and mental health

C.3.12. Statistical considerations

Sample size and power calculation:

The proposed study is a feasibility, individual randomized controlled trial, with a primary focus being on the development of locally adaptable and culturally sensitivity C-QIP intervention and to understand the feasibility, acceptability, and preliminary effects of the intervention on process measures and clinical outcomes. Therefore, we will select 4 CVD clinics (2 government and 2 private, 100 patients each), and a total of 400 patients (stratified by age, and sex) to conduct a feasibility RCT to assess the impact of the C-QIP strategy by examining implementation outcomes, processes of care measures (prescription rates and adherence rates), health-related quality of life, cost, and clinical outcomes. The results of this feasibility RCT, will inform sample size estimates and power calculation for a future outcomes-driven, larger randomized trial to study the effectiveness, cost-effectiveness, and scalability of C-QIP intervention for secondary prevention of CVD.

Data analysis

Feasibility trial: We will report the baseline demographics, clinical history, examination, processes of care measures and laboratory results among the participants before and after the implementation of C-QIP strategy, adjusted for age, sex, and site. The primary analyses will be conducted under the intention to treat principle. All randomized patients will be analysed in the groups to which they were originally allocated to, regardless of whether they actually retained that specific group membership over the course of the trial or not. Patients who withdraw consent for use of their data will not be included in any analyses.

<u>(a) RCT</u>:
Primary outcomes: Implementation outcomes: *feasibility* (number of patients screened, number of patients found eligible, time needed to recruit eligible patients, number of patients randomized to intervention and control group, number of patients who completed scheduled study visits and follow-up rates, number of consent withdrawals, lost to follow-up/drop-out rates, time needed to collect and analyse data); *fidelity* (adherence to study protocol), *adoption* (use of EHR-DSS management plan for the intervention arm patients, number of DSS prompts accepted by the physicians, use of text-messages as reminders for next clinic visit, lab appointment and health lifestyle, number of text-messages sent to the patients), and acceptability of C-QIP strategy will be assessed using treatment satisfaction questionnaire and qualitative interviews with providers, non-physician health workers, health administrators, patients and care-givers.

Secondary outcomes: Processes of care measures (prescription of guideline directed medical therapy (GDMT), and patient's adherence to prescribed GDMT in the intervention and control group). In patients with established CVD, guidelines recommend prescription of two blood pressure lowering drugs (ACEi/ARB, beta-blocker), aspirin and statins.

Exploratory outcomes: clinical changes in blood pressure, LDL-cholesterol, measures of variance in blood pressure i.e., Standard deviation (SD) of the outcome measure (i.e., mean change in systolic blood pressure and LDL-cholesterol level between the two treatment arms at trial end), other biochemical parameters (glycated haemoglobin (HbA1c) and lipid profile), physiological parameters (blood pressure and heart rate), anthropometric parameters (waist circumference, weight, height and body mass index (BMI)), diet, physical activity, tobacco use, health related quality of life (using Global health scale), and cost of care.

Numbers and percentages (for categorical data), and summary measures of location and spread (for continuous data) will be reported. Being a feasibility study, it is not adequately powered to detect a difference in clinical outcomes between the two study arms. However, appropriate regression methods will be used to indicate efficacy of the intervention. Effect sizes will be presented as risk ratios for binary outcomes, and as mean differences for continuous outcomes; 95% confidence intervals will be given for both. Data analysis will be performed using STATA 16.0 (Statacorp Texas).

3.13. Process evaluation

The proposed mixed methods evaluation will leverage the strengths of both qualitative and quantitative methods to better understand the implementation fidelity and impact of C-QIP strategy. The aim of this formative evaluation using RE-AIM framework to assess the reach, adoption, acceptability, satisfaction, enabling factors, barriers and recommendation to inform modifications in the C-QIP strategy and a larger confirmatory randomized controlled trial[81]. Furthermore, we will use the Consolidated Framework for Implementation Research (CFIR) and Implementation Research Logic Model to conduct the process evaluation. To achieve this, we will conduct semi-structured interviews with providers directly involved in the intervention process [physicians (4), non- physician health worker (4), total providers' interviews=8) and intervention arm patients (n=20) and their carers (n=10)]. A qualitative study will be conducted with participants to explore their experience of taking part in the study (both groups) and in the intervention (intervention group only). Depending on the saturation of themes, semi-structured interviews will be conducted with around 20-30 participants. Purposive sampling will be utilised to ensure representation of diversity within the RCT population. (Those who decline to participate in the study will be requested to answer some questions in order to complete a questionnaire and some of them (those agreeing) will be interviewed to further explore the reasons behind. A qualitative study will be conducted with the NPHW (4) to mainly explore their experience of delivering the intervention.) These interviews will be done at the end of the intervention period or trial end. All the semi-structured interviews will be transcribed (verbatim), translated, anonymised, and checked for accuracy. An interpretive analysis will be conducted using thematic analysis, using MAXQDA software. Transcripts will be read and re-read. Initial codes will be developed and applied initially to a small number of transcripts, enabling further iteration of the thematic index. We will use illustrative non-attributable quotations.

C.3.14. Economic evaluation

We will follow international standard guidelines to conduct the economic evaluation alongside this feasibility randomized controlled trial[82-86]. We will document the capital and recurring cost of delivering

the C-QIP intervention [salaries of non-physician health worker, cost of training, cost of patient record system, for educational materials, and overhead costs (facility and administration) minus costs attributable only to research activities e.g., salaries of research assistant]. We will calculate health expenditures (direct and indirect) and estimate cost of delivering C-QIP strategy from healthcare and societal perspective versus usual care. In addition, we will incorporate qualitative data to provide competing beliefs and perceptions from patients, carers, providers, and health administrators about the economic factors that may impede/facilitate C-QIP strategy that need to be addressed to ensure success.

The study will collect data on the following domains:

- Direct costs (over **one year** and **over the duration of the trial**)
    - Costs of the C-QIP strategy, including research costs [DSS software development and maintenance, NPHW salary and benefits, laboratory tests], clinic costs [physician time, research assistant time, overheads, resources for patient management (telephone calls, letters, team meetings, audit and feedback reports, patient diary, and adherence activities)] patient costs [medication and therapeutic procedures, medical supplies, diagnostic tests other than lab and preventative tests covered by study, travel, routine clinic visits (outside the study), emergency room visits, and hospitalizations -- research staff at each clinic site will take detailed information regarding hospitalization including duration of stay, costs, procedures, diagnostics, and will seek consent from the participant to obtain a copy of the discharge summary for each hospital admission - a copy of the discharge summary will be sent to the research coordinating center at PHFI]
    - Costs of standard medical care will include patient costs (majority of expenses are out-of-pocket) and system costs (if government-funded)
- Indirect costs (over **one year** and over **the duration of the trial**)
    - Opportunity costs: salary of participant, salary of caregiver, time spent at clinic waiting, time spent on self-management
    - Lost productivity: symptom count and severity, loss of concentration, loss of function (EQ5D)
- Health outcomes
    - Risk factors: Difference in blood pressure and blood cholesterol from baseline to trial end.
    - any serious adverse events related to C-QIP strategy
- Willingness-to-pay for the C-QIP strategy
    - Interviewing patients and providers participating in the trial regarding their perceived value of the package of C-QIP strategies: EHR-DSS, NPHW, text-messages, patient diary, and audit and feedback reports

C.3.15. Data management

Personal, medical, health behaviours, medical investigations, medications prescription/adherence, costs on health care, processes of care and clinical measures are the information that will be captured in the 'C-QIP electronic health record application in the intervention arm and on a separate tablet application in the control/usual care arm'. Captured information will be kept strictly confidential and it will not be made available to anybody except for the participant, NPHW, doctor and the research team. Case records in electronic form will be stored under password protection. All possible source of identification of participants will be delinked before using the data for analysis. The blood samples collected will be solely used for the purpose of this study (and the participant's care). All the researchers involved in the study will also be trained to follow the protocol terms to maintain participant confidentiality.

1) The following identifiable information will be collected from each participant: Name, age, sex and contact details.
    - Age and sex are important determinants for cardiovascular diseases and for tailor-made case management for prevention of CVD.
    - Contact details are necessary to follow-up with the participants for the duration of the trial.
2) Each participant in the study will be assigned an automatically generated unique identification number (ID). This unique ID is generated once you enter personal details or information. Once the unique ID is created personal details fields will disappear from the electronic data capture of study database. The data files used for monitoring and evaluation will not contain personal information fields.
3) The database server will be housed in restricted-access buildings. All computers hosting the databases of the study will be password protected with restricted access. All electronic data files

will be always maintained under password protection. No paper-based data collection forms will be used.

Post study following completion of all analysis, all personal identifiers will be destroyed and only unique ID will be kept linked to participant data; the remaining information will be stored for 5 years and securely disposed thereafter.

C.3.16. Quality Assurance and Monitoring
During the study, representatives of the RCC will visit all the study sites on at least 3 occasions in the first year and on at least two occasions in the second year. The purpose of these visits will be to ensure that the study is conducted according to the protocol and good clinical practice guidelines are being followed. During the on-site monitoring visits, the trial monitor will review study records and source documents for specific verification of participant details, data quality, and completeness of intervention implementation.

Access to Case Report Forms, source documents, and other study files must be made available at all study sites for monitoring and audit purposes at these monitoring visits during the study and after the study. Any deviations will be documented by the assigned monitoring personnel.

All study data and laboratory samples will only be identified by a participant study ID number. Medical confidentiality will be preserved in accordance with International Conference on Harmonisation (ICH) Good Clinical Practice (GCP) guidelines. Any notes with personal identifiers will only be accessible to the research staffs that are in contact with participants, those responsible for coordinating visits and study personnel authorized by the principal investigator (PI).

In addition, this study will have only electronic version of the study forms and the dataset will be submitted to the data section at Public Health Foundation of India (PHFI), New Delhi, which also serves at the research coordinating centre. All data entry will be done by research team at the hospital sites. There will be built-in data checks and data cleaning will be done by trained data managers at PHFI, under the supervision of the PI. All electronic data will be encrypted, password protected and stored in secure computer networks. All electronic files will be backed up daily.

At the conclusion of the study, the electronic database will be archived in accordance with internal procedures and will be archived for a minimum of 10 years as per current guidelines for the PHFI research projects. Details of the participants of the qualitative research such as in-depth interviews will also be kept confidential and none of the personal identifiers will be made public.

C.4. Ethical considerations
C.4.1. Research ethics approval
This protocol and the patient information sheet-informed consent document and any subsequent modifications will be reviewed and approved by the institutional ethics committee of the Public Health Foundation of India (PHFI), who is responsible for oversight of the study.

C.4.2. Informed Consent
A signed consent form will be obtained from each participant. For participants who are illiterate, a (legally acceptable representative (LAR) or 3rd party witness must also sign the consent form. The consent form describes the purpose of the study, the procedures to be followed, and the risks and benefits of participation. A copy will be given to each participant or legal guardian and this fact will be documented in the participant’s record.

C.4.3. Confidentiality
Participation in this study is purely voluntary with no coercion and no material compensation will be provided for encouraging participation. All participants will be assured that they have the right to voluntarily withdraw from the study at any time, without any repercussions by way of this action affecting their future medical care.

All study data and laboratory samples will only be identified by a participant study ID number. Medical confidentiality will be preserved in accordance with ICH-GCP (international conference on harmonization and good clinical practices) guidelines. Any notes with personal identifiers will only be accessible to the

research staffs that see participants, those responsible for coordinating visits and study personnel authorized by the principal investigator.
In case a participant or a group of participants complain that confidentiality as reported in the consent form was violated that participant(s) can directly approach the principal investigator (PI) about the violation and seek necessary compensation.

C.5. Study organization
The C-QIP study is funded by the Fogarty International Centre (FIC), National Institutes of Health (NIH), USA and organized/coordinated by the Public Health Foundation of India based in Gurgaon, Haryana. The Research Coordinating Centre (RCC) based at PHFI will comprise of the study management team that serves as the operational arm, for day-to-day management and making any recommendations to the Steering Committee. The Research Coordinating Center (RCC), with input from the Steering Committee, is responsible for developing the protocol, certification of Clinical Sites; developing and distributing the Manuel of Procedures; training trial personnel in the standardized protocol implementation and data collection; collecting and managing all trial data; quality control; analyzing data; and preparing reports for the Data Safety Monitoring Board, Steering Committee, and funding agency (FIC, NIH, USA). The RCC will conduct at least annual visits to each Clinical Site to monitor and assure high performance during the trial.

During recruitment for the trial, the RCC will be responsible for monitoring patient recruitment and will provide monthly progress reports to the Steering Committee and other required entities.
The 400 trial participants will be recruited, randomized, treated, and followed at the 4 Clinical Sites. Each Clinical Site consists of an out-patient diabetes clinic which may be an individual practice or under a hospital facility. The RCC will work with the Clinical Sites on issues of trial setup and training, recruitment, compliance with protocol, data management, and quality control. The Clinical Sites will transmit their data directly to the RCC.

An independent Data Safety Monitoring Board (DSMB) will monitor data and oversee patient safety. DMSM members appointed by the Steering Committee, are senior experts in the areas of cardiovascular medicine, clinical trials, biostatistics, and bioethics. The study PI and senior staff of the RCC will participate in DSMB meetings as non-voting members. The DSMB will meet at least once a year to monitor safety, to advise FIC, NIH about the study progress, including to make recommendations to the Steering Committee regarding study continuation and protocol changes. Additionally, the RCC will provide the DSMB Chair any serious adverse event reports and trial data at his/her request at regular intervals to ensure identification of any other major adverse outcomes.

C.6. Publication of research findings
At the end of the study, formal analysis of quantitative and qualitative data will be reviewed for validity and subsequent publication. The investigators will form a Publication, Presentation and Ancillary Studies (PP&A) subcommittee, which will develop a suitable policy protecting the rights of involved organisations regarding ownership of study materials and data. Generally, publication of manuscripts from the study will be in the name of the research group, with each individual study investigator named personally at the end of the report; the support of Fogarty International Centre, National Institutes of Health, USA will be acknowledged. The full editorial control will reside with the Publication, Presentation and Ancillary Studies (PP&A) committee.

C.7. Study duration and timelines
Overall study duration is for 60 months comprising of 6 months for preparation of study documents, and tools, and obtaining institutional ethics committee approvals and health ministry screening committee clearance, 18 months for Phase 1- intervention development, 6 months for pilot testing of the intervention, 24 months for the feasibility trial and last 6 months are earmarked for data analysis, report writing and publication of study results (**Figure 6** and **Table 3**).

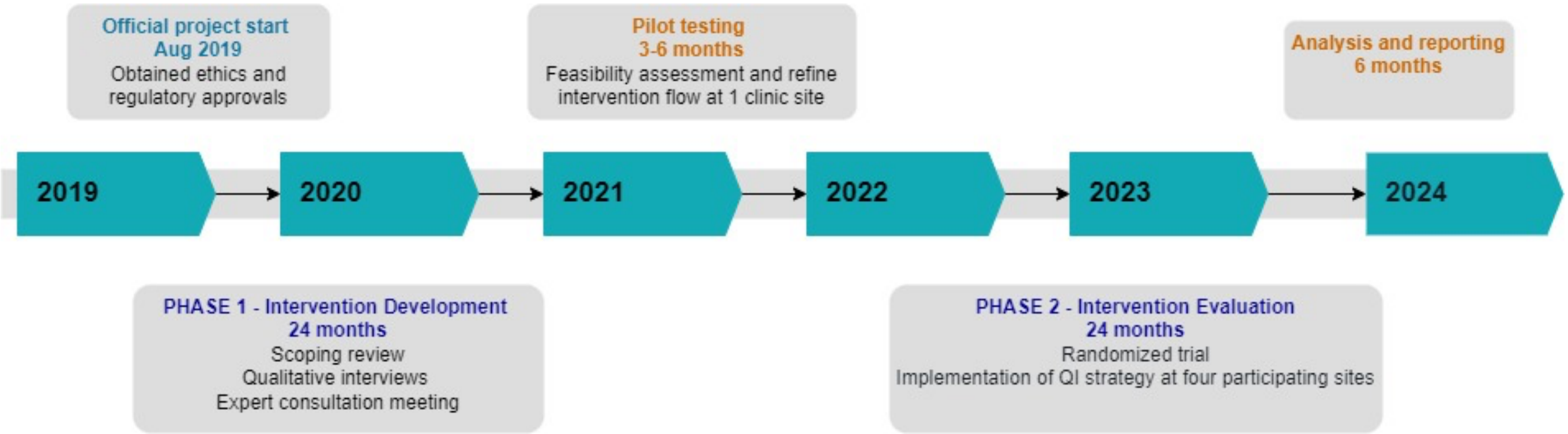

Figure 6. Study timelines

Table 3: Timeline for proposed research activities (X=1/4 year)					
	**Year 1**	**Year 2**	**Year 3**	**Year 4**	**Year 5**
**Design and planning**	X				
IRB clearances and permission	X				
Hiring of research staff	X				
**Study Aims 1 and 2 (Development Phase)**					
Umbrella review	X X X				
Protocols and tools for qualitative research	X				
Recruitment of staff and pilot testing	X X				
Qualitative research data collection and analysis	X X	X X			
Expert consultation and Intervention development		X X			
**Study Aims 3 and 4 (Evaluation Phase)**					
Developing study tools and softwarr's, recruiting research staff		X X X X			
Training of providers, non-physcian health worker		X			
Recruitment of study participants			X X X X		
Feasibility assessment (pilot-testing)		X	X		
Implementation of intervention and follow-up			X X X	X X X X	X X
Process evaluation			X	X	X X
Economic evaluation					X X
Data clearning and analysis					X X X
Abstract/Manuscript preparation	X		X		X X
Final report writing and dissemination of study results					X X
Apply for R01 and other grants for confirmatory trial					X

C.8. Study Discontinuation

The study may be discontinued at any time by the PHFI Ethics Committee, Indian Council of Medical Research (ICMR) or other applicable agencies as part of their duty to ensure that participants are protected. All study staff will be trained in procedures to prevent the potential for breaches of confidentiality, including but not limited to, ensuring that all files are closed and no conversations about individual study participants occur in public settings. All the enrolled and consented participants may discontinue at any point of time during the 24-month duration of the feasibility trial without stating any reason.

C.9. Study Implications

Developing, testing, and implementing a collaborative quality improvement strategy among CVD patients in *diverse mix of CVD clinics (government and private)* provides high quality usable evidence and offers opportunities to continuously refine the quality improvement strategy, while delivering policy- and patient-relevant findings. This project emphasizes paradigms (e.g., self-care, adherence, accountable care) of how to strengthen the Indian health system in contrast to fragmented care. This study will prompt indirect skills development with research personnel and non-physician health workers emerging with at least 2 years of practical experience and the ability to convey their skills to others.

The proposed work will lead to a future study in which we may consider expanding the feasibility study into a large randomized controlled effectiveness trial in South Asia to better understand the impact of C-QIP or related intervention on CVD prevention, which will be relevant to other LMIC settings as well.

D. REFERENCES

1. Prabhakaran D, Singh K, Roth GA, Banerjee A, Pagidipati NJ, Huffman MD. Cardiovascular Diseases in India Compared With the United States. *J Am Coll Cardiol.* 2018;72(1):79-95.
2. Kerr AJ, Broad J, Wells S, Riddell T, Jackson R. Should the first priority in cardiovascular risk management be those with prior cardiovascular disease? *Heart.* 2009;95(2):125-129.
3. Yusuf S. Two decades of progress in preventing vascular disease. *Lancet.* 2002;360(9326):2-3.
4. World Health Organization. Global action plan for the prevention and control of noncommunicable diseases 2013-2020. 2013.
5. Perel P, Avezum A, Huffman M, et al. Reducing Premature Cardiovascular Morbidity and Mortality in People With Atherosclerotic Vascular Disease: The World Heart Federation Roadmap for Secondary Prevention of Cardiovascular Disease. *Glob Heart.* 2015;10(2):99-110.
6. Perel P, Bianco E, Poulter N, et al. Reducing Premature Cardiovascular Mortality By 2025: The World Heart Federation Roadmap. *Glob Heart.* 2015;10(2):97-98.
7. Nieuwlaat R, Schwalm JD, Khatib R, Yusuf S. Why are we failing to implement effective therapies in cardiovascular disease? *Eur Heart J.* 2013;34(17):1262-1269.
8. Yusuf S, Islam S, Chow CK, et al. Use of secondary prevention drugs for cardiovascular disease in the community in high-income, middle-income, and low-income countries (the PURE Study): a prospective epidemiological survey. *Lancet.* 2011;378(9798):1231-1243.
9. Nieuwlaat R, Wilczynski N, Navarro T, et al. Interventions for enhancing medication adherence. *Cochrane Database Syst Rev.* 2014(11):CD000011.
10. Craig P, Dieppe P, Macintyre S, Michie S, Nazareth I, Petticrew M. Developing and evaluating complex interventions: the new Medical Research Council guidance. *Int J Nurs Stud.* 2013;50(5):587-592.
11. Roth GA, Forouzanfar MH, Moran AE, et al. Demographic and epidemiologic drivers of global cardiovascular mortality. *N Engl J Med.* 2015;372(14):1333-1341.
12. Joshi P, Islam S, Pais P, et al. Risk factors for early myocardial infarction in South Asians compared with individuals in other countries. *JAMA.* 2007;297(3):286-294.
13. Shah NS, Huffman MD, Ning H, Lloyd-Jones DM. Trends in myocardial infarction secondary prevention: The National Health and Nutrition Examination Surveys (NHANES), 1999-2012. *J Am Heart Assoc.* 2015;4(4).
14. Shah NS, Huffman MD, Ning H, Lloyd-Jones DM. Trends in vascular risk factor treatment and control in US stroke survivors: the National Health and Nutrition Examination Surveys (1999-2010). *Circ Cardiovasc Qual Outcomes.* 2013;6(3):270-277.
15. Schwalm JD, McKee M, Huffman MD, Yusuf S. Resource Effective Strategies to Prevent and Treat Cardiovascular Disease. *Circulation.* 2016;133(8):742-755.
16. Fogarty International Centre. Strategic Plan. Advancing Science for Global Health. National Institutes of Health. https://www.fic.nih.gov/About/Documents/fogarty-international-center-nih-strategic-plan.pdf [20 October 2017].
17. Fuller RH, Perel P, Navarro-Ruan T, Nieuwlaat R, Haynes RB, Huffman MD. Improving medication adherence in patients with cardiovascular disease: a systematic review. *Heart.* 2018;104(15):1238-1243.
18. World Health Organization. Prevention of Recurrent Heart attack and strokes in low and middle income populations. Evidence based recommendations for policy makers and health professionals. Geneva, 2007. (12 October 2017).
19. Smith SC, Jr., Benjamin EJ, Bonow RO, et al. AHA/ACCF secondary prevention and risk reduction therapy for patients with coronary and other atherosclerotic vascular disease: 2011 update: a guideline from the American Heart Association and American College of Cardiology Foundation endorsed by the World Heart Federation and the Preventive Cardiovascular Nurses Association. *J Am Coll Cardiol.* 2011;58(23):2432-2446.
20. World Health Organization 2007. Guidelines for Prevention of Cardiovascular Disease Guidelines for assessment and management of cardiovascular risk. http://www.who.int/cardiovascular_diseases/guidelines/Full%20text.pdf [14 October 2017].
21. Piepoli MF, Hoes AW, Agewall S, et al. 2016 European Guidelines on cardiovascular disease prevention in clinical practice: The Sixth Joint Task Force of the European Society of Cardiology and Other Societies on Cardiovascular Disease Prevention in Clinical Practice (constituted by representatives of 10 societies and by invited experts)Developed with the special contribution of the European Association for Cardiovascular Prevention & Rehabilitation (EACPR). *Eur Heart J.* 2016;37(29):2315-2381.

22. Law MR, Watt HC, Wald NJ. The underlying risk of death after myocardial infarction in the absence of treatment. *Arch Intern Med.* 2002;162(21):2405-2410.
23. Group PC. Randomised trial of a perindopril-based blood-pressure-lowering regimen among 6,105 individuals with previous stroke or transient ischaemic attack. *Lancet.* 2001;358(9287):1033-1041.
24. Heart Outcomes Prevention Evaluation Study I, Yusuf S, Sleight P, et al. Effects of an angiotensin-converting-enzyme inhibitor, ramipril, on cardiovascular events in high-risk patients. *N Engl J Med.* 2000;342(3):145-153.
25. Antithrombotic Trialists C. Collaborative meta-analysis of randomised trials of antiplatelet therapy for prevention of death, myocardial infarction, and stroke in high risk patients. *BMJ.* 2002;324(7329):71-86.
26. Heart Protection Study Collaborative G. MRC/BHF Heart Protection Study of cholesterol lowering with simvastatin in 20,536 high-risk individuals: a randomised placebo-controlled trial. *Lancet.* 2002;360(9326):7-22.
27. Simes J, Furberg CD, Braunwald E, et al. Effects of pravastatin on mortality in patients with and without coronary heart disease across a broad range of cholesterol levels. The Prospective Pravastatin Pooling project. *Eur Heart J.* 2002;23(3):207-215.
28. Fox KM, Investigators EUtOrocewPiscAd. Efficacy of perindopril in reduction of cardiovascular events among patients with stable coronary artery disease: randomised, double-blind, placebo-controlled, multicentre trial (the EUROPA study). *Lancet.* 2003;362(9386):782-788.
29. Joshi R, Cardona M, Iyengar S, et al. Chronic diseases now a leading cause of death in rural India--mortality data from the Andhra Pradesh Rural Health Initiative. *Int J Epidemiol.* 2006;35(6):1522-1529.
30. Joshi R, Jan S, Wu Y, MacMahon S. Global inequalities in access to cardiovascular health care: our greatest challenge. *J Am Coll Cardiol.* 2008;52(23):1817-1825.
31. Bailey JE, Lee MD, Somes GW, Graham RL. Risk factors for antihypertensive medication refill failure by patients under Medicaid managed care. *Clin Ther.* 1996;18(6):1252-1262.
32. Barat I, Andreasen F, Damsgaard EM. Drug therapy in the elderly: what doctors believe and patients actually do. *Br J Clin Pharmacol.* 2001;51(6):615-622.
33. Hobbs FD, Erhardt L. Acceptance of guideline recommendations and perceived implementation of coronary heart disease prevention among primary care physicians in five European countries: the Reassessing European Attitudes about Cardiovascular Treatment (REACT) survey. *Fam Pract.* 2002;19(6):596-604.
34. Davis M. Current targets: where are we going? *Heart.* 2003;89 Suppl 2:ii6-9; discussion ii35-37.
35. Miller NH, Hill M, Kottke T, Ockene IS. The multilevel compliance challenge: recommendations for a call to action. A statement for healthcare professionals. *Circulation.* 1997;95(4):1085-1090.
36. Paes AH, Bakker A, Soe-Agnie CJ. Impact of dosage frequency on patient compliance. *Diabetes Care.* 1997;20(10):1512-1517.
37. Huffman MD, Rao KD, Pichon-Riviere A, et al. A cross-sectional study of the microeconomic impact of cardiovascular disease hospitalization in four low- and middle-income countries. *PLoS One.* 2011;6(6):e20821.
38. Gaziano TA, Reddy KS, Paccaud F, Horton S, Chaturvedi V. Cardiovascular Disease. In: Jamison DT, Breman JG, Measham AR, Alleyne G, Claeson M, Evans DB, et al., editors. Disease Control Priorities in Developing Countries, 2nd ed. New York: Oxford University Press; 2006. p. 645-62.
39. Lim SS, Gaziano TA, Gakidou E, et al. Prevention of cardiovascular disease in high-risk individuals in low-income and middle-income countries: health effects and costs. *Lancet.* 2007;370(9604):2054-2062.
40. California Medi-Cal Type 2 Diabetes Study G. Closing the gap: effect of diabetes case management on glycemic control among low-income ethnic minority populations: the California Medi-Cal type 2 diabetes study. *Diabetes Care.* 2004;27(1):95-103.
41. Aubert RE, Herman WH, Waters J, et al. Nurse case management to improve glycemic control in diabetic patients in a health maintenance organization. A randomized, controlled trial. *Ann Intern Med.* 1998;129(8):605-612.
42. Choe HM, Mitrovich S, Dubay D, Hayward RA, Krein SL, Vijan S. Proactive case management of high-risk patients with type 2 diabetes mellitus by a clinical pharmacist: a randomized controlled trial. *Am J Manag Care.* 2005;11(4):253-260.
43. Couch C, Sheffield P, Gerthoffer T, Ries A, Hollander P. Clinical outcomes in patients with type 2 diabetes managed by a diabetes resource nurse in a primary care practice. *Proc (Bayl Univ Med Cent).* 2003;16(3):336-340.

44. Davidson MB, Ansari A, Karlan VJ. Effect of a nurse-directed diabetes disease management program on urgent care/emergency room visits and hospitalizations in a minority population. *Diabetes Care.* 2007;30(2):224-227.
45. Fanning EL, Selwyn BJ, Larme AC, DeFronzo RA. Improving efficacy of diabetes management using treatment algorithms in a mainly Hispanic population. *Diabetes Care.* 2004;27(7):1638-1646.
46. Fera T, Bluml BM, Ellis WM, Schaller CW, Garrett DG. The Diabetes Ten City Challenge: interim clinical and humanistic outcomes of a multisite community pharmacy diabetes care program. *J Am Pharm Assoc (2003).* 2008;48(2):181-190.
47. Gabbay RA, Lendel I, Saleem TM, et al. Nurse case management improves blood pressure, emotional distress and diabetes complication screening. *Diabetes Res Clin Pract.* 2006;71(1):28-35.
48. Gary TL, Bone LR, Hill MN, et al. Randomized controlled trial of the effects of nurse case manager and community health worker interventions on risk factors for diabetes-related complications in urban African Americans. *Prev Med.* 2003;37(1):23-32.
49. Krein SL, Klamerus ML, Vijan S, et al. Case management for patients with poorly controlled diabetes: a randomized trial. *Am J Med.* 2004;116(11):732-739.
50. Litaker D, Mion L, Planavsky L, Kippes C, Mehta N, Frolkis J. Physician - nurse practitioner teams in chronic disease management: the impact on costs, clinical effectiveness, and patients' perception of care. *J Interprof Care.* 2003;17(3):223-237.
51. Martin DC, Berger ML, Anstatt DT, et al. A randomized controlled open trial of population-based disease and case management in a Medicare Plus Choice health maintenance organization. *Prev Chronic Dis.* 2004;1(4):A05.
52. Sikka R, Waters J, Moore W, Sutton DR, Herman WH, Aubert RE. Renal assessment practices and the effect of nurse case management of health maintenance organization patients with diabetes. *Diabetes Care.* 1999;22(1):1-6.
53. Xavier D, Gupta R, Kamath D, et al. Community health worker-based intervention for adherence to drugs and lifestyle change after acute coronary syndrome: a multicentre, open, randomised controlled trial. *Lancet Diabetes Endocrinol.* 2016;4(3):244-253.
54. Kamath DY, Xavier D, Gupta R, et al. Rationale and design of a randomized controlled trial evaluating community health worker-based interventions for the secondary prevention of acute coronary syndromes in India (SPREAD). *Am Heart J.* 2014;168(5):690-697.
55. Phillips LS, Ziemer DC, Doyle JP, et al. An endocrinologist-supported intervention aimed at providers improves diabetes management in a primary care site: improving primary care of African Americans with diabetes (IPCAAD) 7. *Diabetes Care.* 2005;28(10):2352-2360.
56. Meigs JB, Cagliero E, Dubey A, et al. A controlled trial of web-based diabetes disease management: the MGH diabetes primary care improvement project. *Diabetes Care.* 2003;26(3):750-757.
57. Ali MK, Singh K, Kondal D, et al. Effectiveness of a Multicomponent Quality Improvement Strategy to Improve Achievement of Diabetes Care Goals: A Randomized, Controlled Trial. *Ann Intern Med.* 2016;165(6):399-408.
58. Group CTW, Shah S, Singh K, et al. Improving diabetes care: multi-component cardiovascular disease risk reduction strategies for people with diabetes in South Asia--the CARRS multi-center translation trial. *Diabetes Res Clin Pract.* 2012;98(2):285-294.
59. Mohanan PP, Mathew R, Harikrishnan S, et al. Presentation, management, and outcomes of 25 748 acute coronary syndrome admissions in Kerala, India: results from the Kerala ACS Registry. *Eur Heart J.* 2013;34(2):121-129.
60. Huffman MD, Mohanan PP, Devarajan R, et al. Effect of a Quality Improvement Intervention on Clinical Outcomes in Patients in India With Acute Myocardial Infarction: The ACS QUIK Randomized Clinical Trial. *JAMA.* 2018;319(6):567-578.
61. Huffman MD, Mohanan PP, Devarajan R, et al. Acute coronary syndrome quality improvement in Kerala (ACS QUIK): Rationale and design for a cluster-randomized stepped-wedge trial. *Am Heart J.* 2017;185:154-160.
62. Singh K, Devarajan R, Mohanan PP, et al. Implementation and acceptability of a heart attack quality improvement intervention in India: a mixed methods analysis of the ACS QUIK trial. *Implement Sci.* 2019;14(1):12.

63. Kalra A, Glusenkamp N, Anderson K, et al. American College of Cardiology (ACC)'s PINNACLE India Quality Improvement Program (PIQIP)-Inception, progress and future direction: A report from the PIQIP Investigators. *Indian Heart J.* 2016;68 Suppl 3:S1-S4.
64. Kalra A, Glusenkamp N, Oetgen WJ, Virani SS. Contemporary data on cardiovascular diseases in Indians: American College of Cardiology's PINNACLE India Quality Improvement Program. *Indian Heart J.* 2016;68(3):434-435.
65. Kalra A, Pokharel Y, Glusenkamp N, et al. Gender disparities in cardiovascular care access and delivery in India: Insights from the American College of Cardiology's PINNACLE India Quality Improvement Program (PIQIP). *Int J Cardiol.* 2016;215:248-251.
66. Pokharel Y, Wei J, Hira RS, et al. Guideline-Directed Medication Use in Patients With Heart Failure With Reduced Ejection Fraction in India: American College of Cardiology's PINNACLE India Quality Improvement Program. *Clin Cardiol.* 2016;39(3):145-149.
67. Kalra A, Pokharel Y, Hira RS, et al. Cardiovascular Disease Performance Measures in the Outpatient Setting in India: Insights From the American College of Cardiology's PINNACLE India Quality Improvement Program (PIQIP). *J Am Heart Assoc.* 2015;4(5).
68. Wood F, Salam A, Singh K, et al. Process evaluation of the impact and acceptability of a polypill for prevention of cardiovascular disease. *BMJ Open.* 2015;5(9):e008018.
69. Salam A, Stewart F, Singh K, et al. INterpreting the Processes of the UMPIRE Trial (INPUT): protocol for a qualitative process evaluation study of a fixed-dose combination (FDC) strategy to improve adherence to cardiovascular medications. *BMJ Open.* 2013;3(5).
70. Thom S, Poulter N, Field J, et al. Effects of a fixed-dose combination strategy on adherence and risk factors in patients with or at high risk of CVD: the UMPIRE randomized clinical trial. *JAMA.* 2013;310(9):918-929.
71. Kirk MA, Kelley C, Yankey N, Birken SA, Abadie B, Damschroder L. A systematic review of the use of the Consolidated Framework for Implementation Research. *Implement Sci.* 2016;11:72.
72. Damschroder LJ, Aron DC, Keith RE, Kirsh SR, Alexander JA, Lowery JC. Fostering implementation of health services research findings into practice: a consolidated framework for advancing implementation science. *Implement Sci.* 2009;4:50.
73. Glasgow RE, Vogt TM, Boles SM. Evaluating the public health impact of health promotion interventions: the RE-AIM framework. *Am J Public Health.* 1999;89(9):1322-1327.
74. Glasgow RE, Harden SM, Gaglio B, et al. RE-AIM Planning and Evaluation Framework: Adapting to New Science and Practice With a 20-Year Review. *Front Public Health.* 2019;7:64.
75. King DK, Shoup JA, Raebel MA, et al. Planning for Implementation Success Using RE-AIM and CFIR Frameworks: A Qualitative Study. *Front Public Health.* 2020;8:59.
76. Yoo SGK, Prabhakaran D, Huffman MD. Evaluating and Improving Cardiovascular Health System Management in Low- and Middle-Income Countries. *Circ Cardiovasc Qual Outcomes.* 2017;10(11).
77. Russell Bernard. 2006. Reserach Methods in Anthropology: Qalitative and Quantitative Approaches.
78. Mendenhall E. Beyond Comorbidity: A Critical Perspective of Syndemic Depression and Diabetes in Cross-cultural Contexts. *Med Anthropol Q.* 2016;30(4):462-478.
79. Mendenhall E. Syndemics: a new path for global health research. *Lancet.* 2017;389(10072):889-891.
80. Pinnock H, Barwick M, Carpenter CR, et al. Standards for Reporting Implementation Studies (StaRI) Statement. *BMJ.* 2017;356:i6795.
81. Oakley A, Strange V, Bonell C, Allen E, Stephenson J, Team RS. Process evaluation in randomised controlled trials of complex interventions. *BMJ.* 2006;332(7538):413-416.
82. WHO Guide to cost-effectiveness analysis. World Health Organization Geneva, 2003. http://www.who.int/choice/publications/p_2003_generalised_cea.pdf. Accessed on 10 August, 2017.
83. Shi L, Hodges M, Drummond M, et al. Good research practices for measuring drug costs in cost-effectiveness analyses: an international perspective: the ISPOR Drug Cost Task Force report--Part VI. *Value Health.* 2010;13(1):28-33.
84. Husereau D, Drummond M, Petrou S, et al. Consolidated Health Economic Evaluation Reporting Standards (CHEERS)--explanation and elaboration: a report of the ISPOR Health Economic Evaluation Publication Guidelines Good Reporting Practices Task Force. *Value Health.* 2013;16(2):231-250.
85. Hay JW, Smeeding J, Carroll NV, et al. Good research practices for measuring drug costs in cost effectiveness analyses: issues and recommendations: the ISPOR Drug Cost Task Force report--Part I. *Value Health.* 2010;13(1):3-7.

86. Drummond M, Barbieri M, Cook J, et al. Transferability of economic evaluations across jurisdictions: ISPOR Good Research Practices Task Force report. *Value Health.* 2009;12(4):409-418.
