# Supplementary material for: Assessing a multicomponent quality improvement strategy for chronic care of cardiovascular diseases in India: The C-QIP randomized feasibility trial
Source: PLoS Med. 2026 Sep 29;23(9):e1004932. doi: 10.1371/journal.pmed.1004932 (PMC13641716; doi:10.1371/journal.pmed.1004932)
Supplement: Supplementary file 5 [file pmed.1004932.s003.docx]

**Online Supplementary file 3**

**Supplement 3: Cardiovascular Quality Improvement (C-QIP) Strategy Components**

**Table 1.** Description of C-QIP strategy components

| C-QIP Component | Target | Who delivered | Frequency | Materials/tools | Intended mechanism | Fidelity indicator |
| --- | --- | --- | --- | --- | --- | --- |
| 1.CCC-facilitated care | Patients | CCC | Monthly/quarterly as per patients’ follow-up plan | CCC training manual | - Regular follow-ups, Reminders  - Coordinated care | - Intervention delivery % |
| 2.EHR-DSS | Physicians | Physician and CCC | Monthly/quarterly as per patients’ follow-up plan | EHR records | - To maintain all records centrally in one place | - prompt acceptance and rejection % |
| 3. SMS reminder | Patients | Automatic text messages | Daily | Text messages in regional languages | - Information  - Reminders | - patient acknowledge receipt of SMS |
| 4.Patient diary | Patients | CCC | Patient Diary - Monthly/quarterly as per patients’ follow-up plan  VITA tool - Daily | Book and calendar templates | - Information  - Medicine adherence | VITA tool completion |
| 5.Audit-feedback | Physician and CCC | Research Team | Quarterly | Reports | - Performance Review  - PDSA plan | PDSA plan completion |

**Refer to the enclosed TiDieR checklist for detailed description of intervention components.*

**Detailed description of C-QIP Strategy Components and mode of delivery**

1. **Training of Non-Physician Health Workers (NPHW)**

NPHWs were trained on-site by the research coordinating center (RCC) team at the start of the trial over 3 days using a standardized training manual. The training module consisted of five modules focusing on the following topics: C-QIP study overview; CVD conditions and risk factors; management of CVDs; behaviour changes in CVD care; and study measurements. The training manual was provided to all NPHWs and were asked to get familiarized with it beforehand. For each module, a pre-module test was conducted, comprising of 10 multiple-choice or short-answer questions for pre-training knowledge assessment. The same test (post-module) was repeated after the completion of modules and the RCC team discussed these answers and provided explanations to incorrect answers as required. Additionally, pre- and post-training assessments were conducted. The RCC team used the pre-training assessment and pre-module tests to identify important topics and emphasised them during the training. During the trial recruitment phase, the RCC team convened virtual meetings quarterly to discuss the study status and common concerns raised by NPHWs over time. The RCC team also circulated monthly newsletters which highlighted recruitment updates and key reminders for NPHWs and physicians based on the study status and monitoring of site activities. In case of staff turnover, the new NPHWs were trained by the RCC team on-site. The newly appointed NPHW was asked to join the clinical team at least 2-3 weeks before the relieving date of existing NPHW and shadow the existing NPHW to learn about the role and responsibilities of NPHW related to patient screening, eligibility assessment, recruitment, investigations, follow-ups visits and to take over from the existing NPHW.

**2. Clinical decision support system (CDSS) algorithm for cardiovascular diseases**

Screen all patients for any of the following (one or more) cardiovascular diseases (aged ≥18 - 75 years)

HFrEF* ≤40%

Ischemic stroke

Coronary Heart Disease

**GDMT**

Statin

Aspirin (low dose)

Lifestyle advice^≠^

**GDMT**

Statin

ACE-I /ARB

Beta-blocker

Aspirin (low dose)

Lifestyle advice^≠^

**GDMT**

ACE-I / ARNI

Beta-blocker

MRA (spironolactone)

Lifestyle advice^≠^

IF BP ≥140/90

ADD thiazide like diuretic OR + ACEi Indapamide

**Loop diuretics** to achieve euvolemia

REVIEW BP (at 3 month): IF BP ≥130 or ≥80; symptoms, adherence, investigations,

ADD starting dose CCB

Up-titrate

ACEi, BB, MRA

ADD full dose of ACEi, BB

REVIEW BP (6 month): IF BP ≥130 or ≥80

**Loop diuretics** to achieve euvolemia

Increase to full dose of CCB

ADD starting dose CCB

REVIEW BP (12 month): IF BP ≥130 or ≥80

**Loop diuretics** to achieve euvolemia

Increase full dose CCB

ADD starting dose ACEi

REVIEW BP: IF BP ≥130 or ≥80

ADD thiazide like diuretic

Increase to full dose of ACEi

Check that patient has been taking drugs regularly and correctly; ADVISE lifestyle modification

GDMT=Guideline directed medical therapy; *Heart failure with reduced ejection fraction

**Coronary Heart Disease Management Plan (Example)**

| **Baseline** | **3-month follow-up** | **6-month follow-up** |
| --- | --- | --- |
| Diagnostics: BP, Lipids panel, HbA1c, FBG, K, Cr, ECG, TTE, Ischemia evaluations  Meds (guideline-directed medical therapy):   a. Aspirin b. Beta-blocker c. ACE-I / ARB d. Statin   Recommend initiation of low doses of 4 medication classes, such as:   a. Aspirin 75 mg daily b. Metoprolol succinate 25 mg daily c. Ramipril 5 mg daily d. Statin 40 mg/Atorva or rosuvastatin 20 mg. | Diagnostics: BP, Lipids panel, HbA1c, FBG, Cr  Meds:  a. If patient is not on all 4 classes of guideline-directed medical therapy (aspirin, two BP lowering drugs and statin), recommend adding the missing drug class.  OR  b. If patient is on all 4 classes of GDMT, and BP>130/90 mmHg or LDL-c >70 mg/dl recommend titrating BP and lipid lowering medications to higher doses (pending blood pressure, lipids, renal function)  For example:   a. Aspirin 75 mg b. Metoprolol succinate 50 mg daily c. Enalapril 5 mg or 10 mg daily d. Statin 20 mg | Diagnostics: BP, Lipids panel, HbA1c, FBG, Cr  Meds:  a. If patient is not on all 4 classes of guideline-directed medical therapy (aspirin, two BP lowering drugs and statin), recommend adding the missing drug class.  OR  b. If patient is on all 4 classes of GDMT, and BP>130/90 mmHg or LDL-c >70 mg/dl recommend titrating BP and lipid lowering medications to higher doses (pending blood pressure, lipids, renal function)  For example:   a. Aspirin 75 mg b. Metoprolol succinate 50 mg daily c. Enalapril 10 mg or 15 mg daily d. Amlodipine 5 mg or 10 mg daily e. Statin 20 mg |

**Heart failure with reduced ejection fraction (HFrEF), EF≤40% Management Plan (Example)**

| **Baseline** | **1-month follow-up** | **3-month follow-up** |
| --- | --- | --- |
| Diagnostics: Basic metabolic panel (K, Cr), BNP, ECG, TTE, Ischemia evaluations  Meds (guideline-directed medical therapy):  a. ACE-I or ARB or ARNI  b. Beta-blocker  c. Mineralocorticoid receptor antagonist  Recommend initiation of low doses of all 3 medication classes, such as:  a. Ramipril or Enalapril 2.5 mg or 5 mg daily  b. Carvedilol 3.125 mg BID or Metoprolol succinate 12.5 mg daily or Bisoprolol 1.25 mg daily.  c. Spironolactone 12.5 mg daily | Diagnostics: Basic metabolic panel (K, Cr), BP, Lipids, Blood sugars  Meds:  a. If patient is not on all 3 classes of guideline-directed medical therapy, recommend adding the missing drug class.  OR  b. If patient is on all 3 classes of guideline-directed medical therapy, recommend titrating one or all of the medications to higher doses (pending blood pressure, renal function, potassium level)  For example:  a. Lisinopril 5 mg or 10 mg daily  b. Carvedilol 6.25 mg BID or Metoprolol succinate 25 mg daily or Bisoprolol 2.5 mg daily.  c. Spironolactone 25 mg daily | Diagnostics: Basic metabolic panel (K, Cr), BP, Lipids, Blood sugars  Meds:  a. If patient is not on all 3 classes of guideline-directed medical therapy, recommend adding the missing drug class.  OR  b. If patient is on all 3 classes of guideline-directed medical therapy, recommend titrating one or all of the medications to higher doses (pending blood pressure, renal function, potassium level)  For example:  a. Lisinopril 20 mg daily  b. Carvedilol 12.5 mg BID or Metoprolol succinate 50 mg daily or Bisoprolol 5 mg daily.  c. Spironolactone 25 mg daily |

**Considerations for patients with HFrEF:**

- When initiating therapy with spironolactone, verify eGFR >30 and serum potassium <5 mEQ/L. If patient develops serum potassium>5.5 mEQ/L, reduce dose, change to every other day dosing, or discontinue therapy. If renal function worsens, consider dose reduction or discontinuation.
- ACE-I or ARB or ARNI should not be given to women who are pregnant or may become pregnant while on therapy.
- Patients with HFrEF and signs of volume overload will likely need diuretics to help decongestion.

**Ischemic Stroke Management Plan (Example)**

| **Baseline** | **3-month follow-up** | **6-month follow-up** |
| --- | --- | --- |
| Diagnostics: BP, Lipids panel, HbA1c, FBG, K, Cr, BNP, ECG, TTE, Ischemia evaluations  Meds (guideline-directed medical therapy):   a. Aspirin b. Beta-blocker c. ACE-I / ARB d. Statin   Recommend initiation of low doses of all 3 medication classes, such as:   a. Aspirin 75 mg daily b. Thiazide like diuretic 1.25 mg or 2.5 mg daily or Fixed dose combination of ACEi/Indapamide (Perindopril + Indapamide, 4/1.25 mg) c. Statin 40 mg or Atorva/Rosuvastatin 20 mg daily | Diagnostics: BP, Lipids panel, HbA1c, FBG, Cr  Meds:  a. If patient is not on all 4 classes of guideline-directed medical therapy (aspirin, two BP lowering drugs and statin), recommend adding the missing drug class.  OR  b. If patient is on all 4 classes of GDMT, and BP>130/90 mmHg or LDL-c >70 mg/dl recommend titrating BP and lipid lowering medications to higher doses (pending blood pressure, lipids, renal function)  For example:   a. Aspirin 75 mg daily b. Thiazide like diuretic 1.25 mg or 2.5 mg daily or Fixed dose combination of ACEi/Indapamide (Perindopril + Indapamide, 4/1.25 mg) c. Statin 40 mg or Atorva/Rosuvastatin 20 mg daily | Diagnostics: BP, Lipids panel, HbA1c, FBG, Cr  Meds:  a. If patient is not on all 4 classes of guideline-directed medical therapy (aspirin, two BP lowering drugs and statin), recommend adding the missing drug class.  OR  b. If patient is on all 4 classes of GDMT, and BP>130/90 mmHg or LDL-c >70 mg/dl recommend titrating BP and lipid lowering medications to higher doses (pending blood pressure, lipids, renal function)  For example:   a. Aspirin 75 mg daily b. Thiazide like diuretic 1.25 mg or 2.5 mg daily or Fixed dose combination of ACEi/Indapamide (Perindopril + Indapamide, 4/1.25 mg) c. Losartan 25 mg or 50 mg or Telmisartan 40 mg or 80 mg d. Statin 40 mg or Atorva/Rosuvastatin 20 mg daily |

^≠^**Lifestyle advise for patients with established CVD at each clinic visit**

1. Stop all tobacco use, avoid second-hand tobacco smoke
2. Drink no more than 2 units of alcohol per day, and avoid alcoholic drinks on at least two days of the week
3. Increase physical activity to equivalent of brisk walk 150 minutes per week
4. If overweight, lose weight
5. Eat heart-healthy diet:
   a. Eat low-salt diet
   b. Eat ≥5 servings of vegetables/fruits per day
   c. Use healthy oils (e.g., olive, sunflower)
   d. Eat nuts, legumes, whole grains, and foods rich in potassium
   e. Limit red meat to once or twice a week at most
   f. Eat fish, or other food rich in omega 3 fatty acids (e.g., flax seeds) at least twice a week.
   g. Avoid added sugar from cakes, cookies, sweets, fizzy drinks, and juice.

**Provision for specific patients and general considerations for DSS configuration**

- ^±^Low-dose aspirin recommended in patients with Ischemic stroke/TIA/Myocardial infarction at least one month ago.
- BP goal: <130/80 for people with CAD, or Stroke
- People with history of heart failure or atrial fibrillation, and cerebrovascular disease
  - Add beta-blocker with initial treatment
- If aspirin intolerant, use clopidogrel
- ACE-I causes chronic cough, in approx. 10% patients if not tolerated, give an ARB, Neither ACE-I nor ARB to given to the pregnant women
- When BP<140/90, ACE inhibitors and beta-blockers should be added unless symptoms preclude
- Consider increasing to intensification dose of thiazide-like-diuretic before further titrations. Hypokalemia more common using intensification dose diuretic – consider increasing lab monitoring.
- **Targets in HF patients**: Heart rate goal: <70 bpm, HbA1c <7%, Hb >12 gm/dl, BMI <25, 6-min walking distance >400 meters
- **Lipids goal: LDLc <70 mg/dl** in those with CVD. Prescribe high-intensity statin, 40 mg Atorvastatin/20 mg rosuvastatin in all with established CHD and cerebrovascular disease, and patients with heart failure with history of CHD/Stroke. Increase dose if LDLc ≥100 mg/dl and the patient can tolerate higher dose
- **Glycemic goal: HbA1c <7%.** Manage diabetes as per the mPOWER HEART/CARRS-T treatment algorithm.
- Reduce BB dose at HR<50. In HF patients, further reduce BB.
- If BP is above target, then adding both ACE+BB are fine. If below target, then replacing CCB with these recommendations is reasonable.
- It is generally recommended to add ACE or ARB post-MI as long as the treating provider does not think that this will result in hypotension.
- Symptomatic patients with BP>180/100 need referral for emergency evaluation and management.
- Statin dose depends on the underlying cause of HF. If due to ischemic cause, then statin therapy per usual secondary prevention. If non-ischemic cardiomyopathy is the cause of HF, then statin is not recommended.
- Dual anti platelet therapy recommendation: Post-MI: yes, Ischemic stroke: debatable HFrEF: no
- If K>5, do not prescribe MRA.
- Stable CHD - If HR<50 reduce BB dose. If patient is on Full dose BB, reduce it to the starting dose. If patient is on starting dose BB, then stop prescribing BB.
- Increase dose of diuretic to full (Diuretic is the first line of treatment to manage BP) if not already on full dose
- Heart Failure - If patient is taking CCB. Advise CCB is not recommended as per the GDMT.
- If a patient has post MI and ischemic stroke – Preference to CDSS rules is given to Post MI - DSS recommendations
- If a patient has ischemic stroke and heart failure - Preference to CDSS rules is given to HFrEF - DSS recommendations
- If a patient has post MI, ischemic stroke and heart failure - Antiplatelet and Statins - will be as per Post MI; Antihypertensives - will be as per HFrEF
- If drug dose is between No to <Half dose consider it as NO, and if the drug dose is between Half dose to <Full dose, consider it as Half dose. And if the drug dose is FULL or >Full dose consider it as Full dose

**CVD Drugs and doses considered in the CDSS**

| **Class** | **Medication** | **Starting dose** | **Intensification dose** | **Frequency** |
| --- | --- | --- | --- | --- |
| Antiplatelet inhibitors | Aspirin (acetyl salicylic acid) | 75 mg |  | Daily |
|  | Clopidogrel | 75 mg |  | Daily |
| Beta-blockers | Metoprolol succinate sustained release | 12.5 mg, 25 mg | 50 mg | Daily |
|  | Carvedilol | 3.125 mg | 6.25 mg, 12.5 mg, 25 | Twice daily |
|  | Carvedilol SR |  |  |  |
|  | Bisoprolol | 1.25 mg | 10 mg | Daily |
|  | Nebivolol | 1.25 mg | 10 mg | Daily |
|  | Atenolol | 25 mg | 50 mg | Daily |
| ACE-inhibitors | Lisinopril | 2.5 or 5 mg | 10 or 20 mg | Daily |
|  | Ramipril | 5 mg | 10 mg | Daily |
|  | Enalapril | 2.5 mg | 20 mg | Twice daily |
|  | Perindopril | 4-5 mg | 8-10 mg |  |
| ARB | Losartan | 50 mg | 100 mg |  |
|  | Telmisartan | 40 mg | 80 mg |  |
|  | Candesartan | 4 - 8 mg | 10- 20mg | Twice daily |
| Diuretic, thiazide like | Chlorthalidone | 12.5 mg | 25 mg | Daily |
|  | Indapamide SR | 1.5 mg | 1.5 mg | Daily |
|  | Chlorothalidone | 12.5 | 25 mg | Daily |
|  | HCTZ | 12.5 mg | 25 mg | Daily |
|  | Indapamide | 2.5 mg | 5 mg | Daily |
|  | Indapamide SR | 1.5 mg |  | Daily |
|  | Metolazone | 2.5 mg | 20 mg | Daily |
| Loop-diuretics | Furosemide | 20 - 40 mg | 100 mg (max. dose) | Daily |
|  | Torasemide | 5 - 20 mg | 40 mg, 100 mg | Daily |
| CCB | Amlodipine | 5 mg | 10 mg | Daily |
|  | Cilnidipine | 5 mg | 10 mg | Daily |
|  | Diltiazem | 90 mg | 360 mg | Daily |
|  | Nifedipine | 10 mg | 40 mg | Daily |
| MRA | Spironolactone | 25 mg | 50 mg | Daily |
|  | Eplerenone | 25 mg | 50 mg | Daily |
| ARNI | Sacubitril/Valsartan | 24/26 mg | 49/51 mg, 97/103 mg | Twice daily |
| Lipid lowering drugs | Atorvastatin | 10 mg | 20 mg | Daily |
|  | Rosuvastatin | 5 mg | 10 mg | Daily |
|  | Simvastatin | 20 mg | 40 mg | Daily |
|  |  |  |  |  |
|  |  |  |  |  |
|  |  |  |  |  |
|  |  |  |  |  |

**Clinical Decision Support System (CDSS) management plan for CVD**

3. **Text message-based reminders for healthy lifestyle**

The text messages are personalized, i.e., it includes patient name, site name, date of follow-up visit, etc. as per the patient’s profile. 3-4 messages based on different themes (diet, exercise, tobacco/alcohol quitting, medicine reminders, clinic visit reminders, weight control, BP, blood cholesterol, blood glucose monitoring etc.) are sent every day. A few text messages are sent more frequently and frequency is reduced over time (for e.g., medicine adherence messages are sent daily for first month, twice a week for second month and only once a week after 2 months). The message includes a URL link and participants are asked to click the link if they have received or read the message.

Template messages along with frequency or schedule of text-messages used in the C-QIP Trial.

| **Welcome and clinic visit reminder messages (All patients)** | **Text message Schedule** |
| --- | --- |
| Hello, Ms/Mrs/Mr. {name}. Your next clinic appointment for heart check-up at {site_name} is on {follow-up_date}. Ensure that your lab tests are done if requested. Skipping appointments or not being as diligent with your heart care in the short-term can lead to long-term health issues including heart attack and stroke. Please click this LINK if you have read this message …………….. **C-QIP Study team** | Twice: 1 week and 2 days prior to next clinic visit date. |
| Hello, Ms/Mrs/Mr. {name}. If you missed your clinic appointment, please contact your Cardiovascular Care Coordinator. Refer to your patient diary for contact details. Please click this LINK if you have read this message …………….. **C-QIP Study team** | 2 days after the overdue clinic visit date |
| **Medicine adherence messages (All patients)** |  |
| Hello, Ms/Mrs/Mr. {name}. Please take your prescribed medicines regularly! Taking regular medicines reduces the chance of hospitalizations and health care costs. If you missed to take your medicines, please contact your Cardiovascular Care Coordinator. Refer to your patient diary for contact details. Please click this LINK if you have read this message …………….. **C-QIP Study team** | **First 4 weeks**:  daily once at 11 am  **Next 4 weeks**:  two days in a week  (Monday, 11 am  Thursday, 11 am)  **After 2 months**:  Once a week  (Monday, 11 am) |
| Hello, Ms/Mrs/Mr. {name}. Get into the habit of taking medicines at the same time each day- like after your morning prayers or after morning/evening meals, or when you brush your teeth at night. Please complete the VITA tool daily after taking your medicines. For any questions, please contact your Cardiovascular Care Coordinator. Refer to your patient diary for contact details. Please click this LINK if you have read this message …………….. **C-QIP Study team** | **First 4 weeks**:  daily once at 6 pm  **Next 4 weeks**:  two days in a week  (Monday, 6 pm,  Thursday, 6 pm)  **After 2 months**:  Once a week  (Thursday, 6 pm) |
| **Diet messages (All patients)** |  |
| Hello, Ms/Mrs/Mr. (Name). Making small changes can make a big difference for your heart health. Add fresh vegetables and seasonal fruits to your diet! A colourful plate with plenty of red, orange, yellow, and green vegetables help ensure you are getting the vitamins and minerals you need. Please click this LINK if you have read this message …………….. **C-QIP Study team** | Once a week (Sunday, 9am) |
| Hello, Ms/Mrs/Mr. (Name). Making small changes can make a big difference for your heart health. Add whole grains, pulses and nuts to your diet! Please click this LINK if you have read this message …………….. **C-QIP Study team** | Once a week (Tuesday, 12 noon) |
| Hello, Ms/Mrs/Mr. (Name). As we grow older, we become more sensitive to salt, which can cause swelling in the legs and feet. Excess salt can increase your blood pressure and your risk for future heart attack and stroke. Reduce salt intake in your diet (around 1 teaspoon per day). Please click this LINK if you have read this message …………….. **C-QIP Study team** | Once a week (Thursday, 12 noon) |
| Hello, Ms/Mrs/Mr. (Name). **FOLLOW HEART-HEALTHY DIET**. Eating fried foods, high fat and packaged foods, and sweets can lead to weight gain and worsening of your heart disease and diabetes. Avoid fried foods, packaged foods, and sweets. (Less than 5 teaspoons of fats/oils in a day). Please click this LINK if you have read this message …………….. **C-QIP Study team** | Once a week (Saturday, 12 noon) |
| **Exercise messages (All patients)** |  |
| Hello, Ms/Mrs/Mr. (Name). **Be physically more active.** Start by activities you enjoy – walking, playing, gardening or household chores. Avoid sitting for long hours. Please click this LINK if you have read this message …………….. **C-QIP Study team** | Once a week (Sunday, 6am) |
| Hello, Ms/Mrs/Mr. (Name). **"Exercise is medicine for a Healthy Heart"**. Aim for 30 minutes of daily exercise such as walking, cycling, gardening, cleaning, or playing a sport. Please click this LINK if you have read this message …………….. **C-QIP Study team** | Once a week (Tuesday, 6am) |
| Hello, Ms/Mrs/Mr. (Name). Simple activities like taking the stairs instead of elevators, walking to nearest grocery store or doing domestic chores such as cleaning can help you to be physically active! Please click this LINK if you have read this message …………….. **C-QIP Study team** | Once a week (Thursday, 6am) |
| Hello, Ms/Mrs/Mr. (Name). Not feeling like exercising? Even a 5-minute walk has real health benefits and it is absolutely free. Start with 5 minutes of exercise and increase it over time. Please click this LINK if you have read this message …………….. **C-QIP Study team** | Once a week (Saturday, 6am) |
| **Tobacco messages (Only to those who are current smokers)** |  |
| Hello, Ms/Mrs/Mr. (Name). **Tobacco or** Smoking damages your heart walls. It is never too late to get benefits from quitting smoking/tobacco. Quitting, even in later life, can lower your risk of future heart attack, stroke, and cancer over time. Please click this LINK if you have read this message …………….. **C-QIP Study team** | once a week (Monday, 8am) |
| Hello, Ms/Mrs/Mr. (Name). The benefits of quitting tobacco or smoking are almost immediate. After 12 hours of quitting smoking, the carbon monoxide level in your blood drops to normal. Within 2-12 weeks, your circulation improves and lung function increases and within 1-9 months, coughing and shortness of breath decrease. Choose your health and quit tobacco or smoking! Please click this LINK if you have read this message …………….. **C-QIP Study team** | Once a week (Wednesday, 8am) |
| Hello, Ms/Mrs/Mr. (Name). Tobacco users or smokers have up to 2-times increased risk of stroke and a 4-times increased risk of heart disease. Quit tobacco or smoking to avoid stroke and heart diseases. Please click this LINK if you have read this message …………….. **C-QIP Study team** | Once a week (Friday, 8am) |
| Hello, Ms/Mrs/Mr. (Name). Tobacco users or smokers are at much higher risk of falling ill and dying early of cancer, heart attack, and lung diseases. Quit tobacco or smoking to avoid cancer, heart attack and lung diseases! Please click this LINK if you have read this message …………….. **C-QIP Study team** | Once in 2-weeks (1^st^ and 3^rd^ Saturdays, 8am) |
| Hello Ms/Mrs/Mr. (Name). Reduce your craving for cigarettes/ tobacco, with substitutes such as nicotine transdermal patches or nicotine tablets. Please click this LINK if you have read this message …………….. **C-QIP Study team** | Once in 2-weeks (2^nd^ and 4^th^ Sundays, 8am) |
| Hello Ms/Mrs/Mr. (Name). Replace your cigarettes/ tobacco with healthy habits. Reduce your craving for cigarettes/ tobacco, with substitutes such as chewing gums, peppermints, cloves, saunf, or cardamom. Please click this LINK if you have read this message …………….. **C-QIP Study team** | Once in 2-weeks (1^st^ and 3^rd^ Mondays, 8am) |
| Hello Ms/Mrs/Mr. (Name). One week of less tobacco consumption/smoking! You may have noticed some changes in your body. This will pass quickly. Great work! Keep continuing your journey with us. In our next clinic appointment, we hope to see you much better. Please click this LINK if you have read this message …………….. **C-QIP Study team** | 1 week after the clinic visit (if patient is achieving the goal) - manually sent by CCC once a week. |
| Hello Ms/Mrs/Mr. (Name). One week without tobacco consumption or smoking. Quitting smoking is the best thing you have done for yourself and your family. We are proud of you! For any questions, please contact your Cardiovascular Care Coordinator. Refer to your patient diary for contact details. Please click this LINK if you have read this message …………….. **C-QIP Study team** | If patient reports quitting tobacco /smoking for one week  manually sent by CCC once a week. |
| Hello Ms/Mrs/Mr. (Name). One month without tobacco consumption or smoking. Quitting tobacco or smoking is a transformational process that benefits your health, your life and your family. We are proud of you! For any questions, please contact your Cardiovascular Care Coordinator. Refer to your patient diary for contact details. Please click this LINK if you have read this message …………….. **C-QIP Study team** | If patient reports quitting tobacco / smoking for one month  manually sent by CCC once a month. |
| **Alcohol messages (only to current alcohol users)** |  |
| Hello, Ms/Mrs/Mr. (Name). **Drinking alcohol is bad for your heart heath!** Doctors recommend not to drink or to limit alcohol drinking to 2 drinks or less in a day for men and only one drink or less in a day for women. Please click this LINK if you have read this message …………….. **C-QIP Study team** | Once a week (Saturday morning, 9am) |
| Hello, Ms/Mrs/Mr. (Name). Alcohol use can lead to high blood pressure, heart failure and stroke. Improve your heart health, stop alcohol drinking! Please click this LINK if you have read this message …………….. **C-QIP Study team** | Once a week (Sunday morning, 9am) |
| Hello, Ms/Mrs/Mr. (Name). Drinking too much alcohol can lead to more than 200 diseases, e.g., heart problems, liver diseases, cancer, and injuries. Choose your heart health, stop alcohol drinking! Please click this LINK if you have read this message …………….. **C-QIP Study team** | Once a week (Monday eve, 5pm) |
| Hello, Ms/Mrs/Mr. (Name). People who don't drink alcohol are healthier, can control their weight easily, more alert and sleep better compared to people who drink alcohol. Avoid alcohol drinking! Please click this LINK if you have read this message …………….. **C-QIP Study team** | Once a week (Tues eve, 5pm) |
| Hello, Ms/Mrs/Mr. (Name). Avoiding alcohol drinking has a lot of benefits such as low blood sugar levels and low cholesterol levels! Avoid alcohol drinking! Please click this LINK if you have read this message …………….. **C-QIP Study team** | Once a week (Wed, eve, 5pm) |
| Hello Ms/Mrs/Mr. (Name). Your craving for alcohol is TEMPORARY but the damage to your heart is PERMANENT. **Replace** your alcoholic drink with a glass of water/fresh fruit juice/non-alcoholic drink and engage yourself in an activity that you enjoy. Please click this LINK if you have read this message …………….. **C-QIP Study team** | Once a week. (Thursday, evening, 5pm) |
| Hello Ms/Mrs/Mr (Name). One week of less alcohol drinking! You may have noticed some changes in your body. This will pass quickly. Great work! Keep continuing your journey with us. In your next clinic visit, we hope to see you much better. Please click this LINK if you have read this message …………….. **C-QIP Study team** | 1 week after (before) the (next) clinic visit (if patient is achieving the goal) - manually sent by CCC once a week. |
| Hello Ms/Mrs/Mr. (Name). One week without alcohol drinking. Stopping alcohol drinking is the best thing you have done for yourself and your family. We are proud of you! For any questions, please contact your Cardiovascular Care Coordinator. Refer to your patient diary for contact details. Please click this LINK if you have read this message …………….. **C-QIP Study team** | If patient reports stopping alcohol drinking for one week  manually sent by CCC once a week. |
| Hello Ms/Mrs/Mr. (Name). One month without alcohol drinking. Stopping alcohol drinking is a transformational process that benefits your health, your life and your family. For any questions, please contact your Cardiovascular Care Coordinator. Refer to your patient diary for contact details. Please click this LINK if you have read this message …………….. **C-QIP Study team** | If patient reports stopping alcohol drinking for one month  manually sent by CCC once a month. |
| **Stress messages (all patients)** |  |
| Hello, Ms/Mrs/Mr. (Name). Talk to your friends, family, Cardiovascular Care Coordinator or Doctor to improve your emotional and physical health. “A healthy heart will give you the courage to face and overcome any challenge in life.” Please click this LINK if you have read this message …………….. **C-QIP Study team** | Once in 2-weeks (1^st^ and 3^rd^ Mondays, evening 6 pm) |
| **Clinical signs and Symptoms messages (all patients)** |  |
| Hello, Ms/Mrs/Mr. (Name). Contact your Cardiovascular Care Coordinator if you feel chest pain, discomfort, shortness of breath, confusion, dizziness, cold sweat, swelling in ankles, feet, or legs. Please click this LINK if you have read this message …………….. **C-QIP Study team** | Once a week for the first month, then bi-weekly for next 2months. (Wednesday, evening 5pm) |
| **Weight control messages (all patients)** |  |
| Hello, Ms/Mrs/Mr. (Name). **Watch your weight.** Keep a healthy weight. Some ways to maintain a healthy weight include limiting portion size and being physically active. Please click this LINK if you have read this message …………….. **C-QIP Study team** | Once in 2-weeks (2^nd^ and 4^th^ Saturdays, morning 7 am) |
| **BP, Cholesterol, and Blood sugar monitoring (all patients, Blood sugar – only for Diabetes patients)** |  |
| Hello, Ms/Mrs/Mr. (Name). **Watch your numbers.** Keep your blood pressure under control (**BP<140/80** mmHg). Please regularly check BP at home or at the nearest chemist shop or clinic as per your doctor's advice. High blood pressure could lead to problems with your heart, eye, brain, and kidneys. Please click this LINK if you have read this message …………….. **C-QIP Study team** | Monthly (1^st^ Friday morning, 7 am) |
| Hello, Ms/Mrs/Mr. (Name). **Watch your numbers.** Keep your blood cholesterol under control (**LDLc<100** mg/dl). High blood cholesterol can block/damage your heart arteries and increase risk of death. Please click this LINK if you have read this message …………….. **C-QIP Study team** | Monthly (2^nd^ Saturday morning, 7 am)  “Manually sent by CCC or 3-monthly if controlled” |
| Hello, Ms/Mrs/Mr. (Name). **Watch your numbers.** Keep your blood sugar levels under control (**FBG<100** mg/dl). High blood sugar could lead to problems with your heart, eye, brain, and kidneys. Please click this LINK if you have read this message …………….. **C-QIP Study team** | Monthly (3^rd^ Saturday morning, 7 am)  “Manually sent by CCC or 3-monthly if controlled” |

4. **Patient Diary – Screenshot of Goals setting for Heathy Lifestyle**

**Screenshot of VITA Tool for Medication Adherence**

**5. C-QIP Audit and Feedback Report (template) and PDSA worksheet**
