# Supplementary material for: Assessing a multicomponent quality improvement strategy for chronic care of cardiovascular diseases in India: The C-QIP randomized feasibility trial
Source: PLoS Med. 2026 Sep 29;23(9):e1004932. doi: 10.1371/journal.pmed.1004932 (PMC13641716; doi:10.1371/journal.pmed.1004932)
Supplement: Supplementary file 6 [file pmed.1004932.s005.docx]

**Supplementary Figures**

## **Fig A** Adoption of electronic decision support system (eDSS) prompts review and acceptance by physicians: overall and by site.

### a) Antiplatelet Prompts

|  | Baseline (N=198) | 12 months (N=92) | EOS (N=187) |
| --- | --- | --- | --- |
| Overall use of Antiplatelet prompts | 67.7% | 59.8% | 70.6% |
| Site 01 | 73% | 74% | 62% |
| Site 02 | 58.5% | 44.8% | 49% |
| Site 03 | 59.6% | 47.6% | 87.2% |
| Site 04 | 78.7% | 78.3% | 81.8% |

### b) Blood Pressure Prompts

|  | Baseline (N=198) | 12 months (N=91) | EOS (N=186) |
| --- | --- | --- | --- |
| Overall use of Blood Pressure prompts | 40.4% | 49.5% | 51.6% |
| Site 01 | 27% | 42% | 29% |
| Site 02 | 54.7% | 60.7% | 54.9% |
| Site 03 | 25.5% | 14.3% | 58.7% |
| Site 04 | 47.5% | 73.9% | 56.4% |

### c) Lipids Prompts

|  | Baseline (N=198) | 12 months (N=92) | EOS (N=187) |
| --- | --- | --- | --- |
| Overall use of Lipids prompts | 68.2% | 68.1% | 63.9% |
| Site 01 | 72% | 68% | 53% |
| Site 02 | 51% | 46.4% | 56% |
| Site 03 | 67.4% | 71.4% | 89.1% |
| Site 04 | 67.2% | 91.3% | 74.5% |

### d) Glycemia eDSS Prompts

|  | Baseline (N=198) | 12 months (N=92) | EOS (N=187) |
| --- | --- | --- | --- |
| Overall use of Glucose prompts | 80.6% | 81.6% | 79.7% |
| Site 01 | 72% | 68% | 53% |
| Site 02 | 51% | 46.4% | 56% |
| Site 03 | 67.4% | 71.4% | 89.1% |
| Site 04 | 67.2% | 91.3% | 74.5% |

## **Fig B** Self-reported medication adherence at EOS by prespecified sub-groups

*CI=confidence interval; BMI=body mass index (Kg/m2); LDLc=low-density lipoprotein cholesterol, mg/dl=milligrams per deciliter; mmHg=millimoles per mercury; INR=Indian rupees*

**Fig C** Mean systolic blood pressure reduction at end-of-study by prespecified subgroups

*CI=confidence interval; BMI=body mass index (Kg/m2); LDLc=low-density lipoprotein cholesterol, mg/dl=milligrams per deciliter; mmHg=millimoles per mercury*
