# Supplementary material for: Assessing a multicomponent quality improvement strategy for chronic care of cardiovascular diseases in India: The C-QIP randomized feasibility trial
Source: PLoS Med. 2026 Sep 29;23(9):e1004932. doi: 10.1371/journal.pmed.1004932 (PMC13641716; doi:10.1371/journal.pmed.1004932)
Supplement: Supplementary file 7 [file pmed.1004932.s006.docx]

**Supplementary Tables**

## **Table A** Fidelity of C-QIP Strategy: Self-reported cardiovascular care coordinator delivered counselling of lifestyle modifications for intervention arm participants

|  | **Baseline**  **(N=198)** | **12 months follow-up (N=114)** | **EOS**  **(N=197)** |
| --- | --- | --- | --- |
| **Participant counselled for lifestyle modifications** | **197 (99.5%)** | **91 (79.8%)** | **187 (94.4%)** |
| Healthy diet | 197 (100%) | 91 (100%) | 186 (99.5%) |
| Exercise | 197 (100%) | 91 (100%) | 184 (98.4%) |
| Smoking cessation  *(If said yes/quit to smoking)* | 24 (68.6%)  (n=35) | 6 (100%) (n=6) | 9 (60%)  (n=15) |
| Avoidance of stress | 159 (80.7%) | 82 (90.1%) | 170 (90.9%) |
| Abstinence/ limited use of alcohol  *(If said yes to alcohol)* | 10 (83.3%)  (n=12) | 10 (83.3%)  (n=12) | 2 (33.3%)  (n=6) |
| Home monitoring of blood pressure | 159 (80.7%) | 83 (91.2%) | 169 (90.4%) |

*N=number of participants; EOS=End of study visit*

## **Table B** Feasibility of electronic decision-support system recommended intermediate follow-up visits completion for intervention arm participants

| **Intermediate Visit (IV)** | **Expected** | **Completed** |
| --- | --- | --- |
| **Between baseline and annual visits** | | |
| IV 2.1 | 195 | 193 |
| IV 2.2 | 193 | 188 |
| IV 2.3 | 188 | 180 |
| IV 2.4 | 151 | 117 |
| IV 2.5 | 97 | 62 |
| IV 2.6 | 53 | 31 |
| IV 2.7 | 26 | 13 |
| IV 2.8 | 13 | 3 |
| IV 2.9 | 3 | 1 |
| **Post annual visits** | | |
| IV 3.1 | 66 | 55 |
| IV 3.2 | 41 | 33 |
| IV 3.3 | 17 | 7 |
| IV 3.4 | 4 | 1 |
| IV 3.5 | 1 | 1 |

*IV=Intermediate visits*

*The table above shows the expected and actual number of completed intermediate visits between the baseline and annual visits, and visits completed between the first annual visit and end of the study visit.*

*Average follow-up visit duration between intermediate visits was approximately 3 months or as recommended per the electronic decision support system prompts for chronic care of patients with ischemic heart disease, ischemic stroke or heart failure regardless of ejection fraction.*

## **Table C** Self-reported timeliness of care and type of reminders received

|  | **Baseline** | | **End of Study (EOS)** | | **p-value*** |
| --- | --- | --- | --- | --- | --- |
|  | **C-QIP strategy (N=198)** | **Usual care (N=203)** | **C-QIP strategy (N=187)** | **Usual care (N=192)** |  |
| **Total time spend for lab tests, include waiting (in minutes; Median, IQR)** | 30 (15, 45) | 20 (15, 35) | 20 (15, 40) | 30 (15, 60) | **<0.001** |
| **Total time spend waiting for consultation  (in minutes; Median, IQR)** | 20 (10, 40) | 20 (10, 60) | 15 (10, 25) | 20 (10, 40) | 0.13 |
| **Total time spend in-person with doctor  (in minutes; Median, IQR)** | 9 (5, 10) | 8 (5, 10) | 10 (5, 10) | 7 (5, 10) | **<0.001** |
| **Type of reminders received** |  |  |  |  |  |
| Verbal reminder at current visit | 147 (74.2%) | 143 (70.4%) | 130 (65.7%) | 134 (66%) | 0.95 |
| Phone call for upcoming appointment | 56 (28.3%) | 71 (35%) | 93 (47%) | 57 (28.1%) | **<0.001** |
| Phone call if missed appointment | 43 (21.7%) | 34 (16.8%) | 140 (70.7%) | 9 (4.4%) | **<0.001** |
| Phone call to go for lab investigations | 13 (6.6%) | 9 (4.4%) | 82 (41.4%) | 1 (0.5%) | **<0.001** |
| **p-value is for the difference between the two groups at end of study* | | | | | |

## **Table D** Self-reported family support at baseline and end of study.

| **Family has helped the participant to** | | **Baseline** | | **End of Study (EOS)** | | **p-value*** |
| --- | --- | --- | --- | --- | --- | --- |
|  | | **C-QIP strategy (N=198)** | **Usual care (N=203)** | **C-QIP strategy (N=198)** | **Usual care (N=203)** |  |
| Eat healthy | | 182 (91.9%) | 146 (71.9%) | 187 (94.4%) | 138 (68%) |  |
| Exercise | | 150 (75.8%) | 126 (62.1%) | 185 (93.4%) | 132 (65%) | 0.06 |
|  | | **N=35** | **N=26** | **N=45** | **N=38** |  |
| Quit smoking  *(If said yes/quit to smoking)* | | 14 (40%) | 7 (15.6%) | 9 (34.6%) | 1 (2.6%) | **<0.001** |
| **Family reminds participant to** | |  |  |  |  |  |
| Eat healthy | | 176 (88.9%) | 141 (69.5%) | 187 (94.4%) | 138 (68%) |  |
| Exercise | | 157 (79.3%) | 129 (63.6%) | 181 (91.4%) | 127 (62.6%) | 0.06 |
|  | | **N=35** | **N=26** | **N=45** | **N=38** |  |
| Quit smoking  *(If said yes/quit to smoking)* | | 14 (40%) | 8 (17.8%) | 8 (30.8%) | 1 (2.6%) | **<0.001** |
| **Family joins with the participant to** | |  |  |  |  |  |
| Eat healthy | | 170 (85.9%) | 136 (67%) | 186 (93.9%) | 137 (67.5%) | 0.39 |
| Exercise | | 103 (52%) | 75 (37%) | 127 (64.1%) | 96 (47.3%) | 0.68 |
|  | | **N=35** | **N=26** | **N=45** | **N=38** |  |
| Quit smoking  *(If said yes/quit to smoking)* | | 7 (20%) | 2 (4.4%) | 2 (7.7%) | - | **0.05** |
|  | **p-value is for the difference between the two groups at EOS* | | | | | |

## **Table E** Serious adverse events

| **Event description** | **Total events reported in the trial** | | |
| --- | --- | --- | --- |
|  | **Total** | **C-QIP strategy** | **Usual care** |
| **Deaths (all cause)** | **19** | **9** | **10** |
| Cardiovascular deaths | 11 | 6 | 5 |
| Other deaths* | 8 | 3 | 5 |
| Death followed by  severe illness |  |  | 3 |
| Road traffic accident |  |  | 1 |
| Cancer complications |  | 1 |  |
| Deteriorating health |  | 1 |  |
| Post-operation  complications |  | 1 |  |
| Difficulty in breathing |  |  | 1 |
| **Hospitalizations** | **5** | **5** | **0** |
| Angina on exertion | 2 | 2 |  |
| Chest pain | 1 | 1 |  |
| Anteroseptal  Myocardial Infarction | 1 | 1 |  |
| Planned angiography | 1 | 1 |  |
|  | | | |

## **Table F** Comparison of baseline characteristics of participants who completed the EOS visit versus participants who did not complete EOS visit

|  | **Completed EOS visit** | **Lost to follow up** | **p-value** |
| --- | --- | --- | --- |
|  | N=379 | N=22 |  |
| Age (years; mean, SD) | 57.1 (11.7) | 62.7 (11.0) | 0.03 |
| Male, n (%) | 277 (73.1%) | 16 (72.7%) | 0.97 |
| Education, (years; mean, SD) | 10.7 (5.6) | 11.6 (4.3) | 0.44 |
| Household Income (monthly) Indian rupees, median (IQR) | 20000 (10000, 30000) | 27500 (20000, 30000) | 0.05 |
| Systolic BP (mm Hg; mean, SD) | 127.4 (18.1) | 134.5 (19.9) | 0.07 |
| Diastolic BP (mm Hg; mean, SD) | 76.2 (11.4) | 78.8 (14.3) | 0.30 |
| Total cholesterol (mg/dL; mean, SD) | 144.3 (44.2)  (n=370) | 149.0 (37.7) (n=22) | 0.63 |
| LDLc (mg/dL; mean, SD) | 80.2 (37.4) (n=367) | 84.7 (35.2) (n=21) | 0.59 |
| FBG (mg/dL; mean, SD) | 123.0 (55.2)  (n=182) | 87.6 (23.6)  (n=4) | 0.20 |
| HbA1c (%; mean, SD) | 6.7 (1.6)  (n=225) | 8.0 (1.7)  (n=7) | 0.03 |
| **Medical history** |  |  |  |
| Coronary heart disease, n (%) | 286 (75.5%) | 10 (45.5%) | 0.002 |
| Heart failure, n (%) | 76 (20.1%) | 6 (27.3%) | 0.41 |
| Ischemic stroke, n (%) | 65 (17.2%) | 9 (40.9%) | 0.005 |
| Hypertension, n (%) | 178 (47.0%) | 15 (68.2%) | 0.05 |
| Diabetes, n (%) | 155 (40.9%) | 13 (59.1%) | 0.09 |
| **Medications use** |  |  |  |
| Antiplatelet | 336 (88.7%) | 15 (68.2%) | 0.005 |
| Lipid lowering (Statin) | 330 (87.1%) | 14 (63.6%) | 0.002 |
| ACEi/ARB | 227 (59.9%) | 12 (54.5%) | 0.62 |
| ARNI (n=82; heart failure patients) | 24 (32%) | 1 (17%) | 0.44 |
| Beta-blockers | 269 (71.0%) | 15 (68.2%) | 0.78 |
| Calcium Channel Blockers | 64 (16.9%) | 7 (31.8%) | 0.07 |
| Diuretics | 29 (7.7%) | 1 (4.5%) | 0.59 |
| Did participant forget to take medications (in last 1 month)? | 56 (14.8%) | 3 (13.6%) | 0.96 |
| On feeling worse does the participant stops medication on his/her own? | 19 (5.0%) | 0 (0.0%) | 0.28 |

**EOS=End of study visit; SD=standard deviation, mg/dl=milligrams per deciliter, BP=blood pressure*

## **Table G** Prescription of medications and adherence to self-care measures at end of study visit – Using Inverse Probability Weighting

| **Medications use** | **Relative Risk (95% CI)** | **p value** | **Relative Difference, %, (95% CI)** |
| --- | --- | --- | --- |
| **Antiplatelet** | 1.09 (1.02, 1.16) | **0.006** | 7.8 (2.3, 13.2) |
| **Lipid lowering** | 1.18 (1.10, 1.26) | **<0.001** | 14.0 (8.2, 19.8) |
| **ACEi/ARB** | 1.17 (1.01, 1.36) | **0.041** | 9.2 (0.6, 17.9) |
| **Beta-blockers** | 1.17 (1.05, 1.31) | **0.005** | 11.4 (3.7, 19.2) |
| **Calcium channel blockers** | 1.31 (0.91, 1.90) | 0.151 | 5.2 (-1.9, 12.3) |
| **Diuretics** | 1.84 (0.98, 3.46) | 0.058 | 4.8 (-0.0, 9.8) |
| **ARNI (only HF)** | 0.87 (0.50, 1.51) | 0.612 | -4.4 (-21.5, 12.7) |
| **Patients with coronary heart disease (N=296)** | | | |
| **Medications use** |  |  |  |
| Antiplatelet | 1.08 (1.03, 1.14) | **0.003** | 7.5 (2.6, 12.4) |
| Lipid lowering | 1.15 (1.08, 1.23) | **<0.001** | 12.7 (7.0, 18.4) |
| ACEi/ARB | 1.09 (0.92, 1.29) | 0.319 | 5.1 (-4.9, 15.1) |
| Beta-blockers | 1.18 (1.06, 1.32) | **0.003** | 13.2 (4.7, 21.7) |
| Antiplatelet + Lipid lowering drug (statin) + ACEi/ARB + Beta Blocker | 1.53 (1.22, 1.91) | **<0.001** | 19.2 (9.9, 28.6) |
| **Patients with ischemic stroke (N=74)** | | | |
| **Medications use** |  |  |  |
| Antiplatelet | 1.12 (0.94, 1.34) | 0.207 | 9.5 (-4.9, 23.8) |
| Lipid lowering | 1.30 (1.05, 1.60) | **0.016** | 21.0 (5.3, 36.7) |
| ACEi /ARB/Diuretics | 1.52 (1.09, 2.10) | **0.013** | 27.0 (6.4, 47.6) |
| Antiplatelet + Lipid lowering drug (statin) + ACEi/ARB/Diuretic | 2.24 (1.41, 3.55) | **0.001** | 40.4 (20.7, 60.0) |
| **Patients with Heart failure (N=82)** | | | |
| **Medications use** |  |  |  |
| ACEi/ARB/ARNI | 1.08 (0.83, 1.41) | 0.560 | 5.6 (-13.2, 24.5) |
| Beta-blockers | 1.04 (0.83, 1.30) | 0.757 | 2,9 (-15.4, 21.2) |
| MRA | 1.19 (0.91, 1.54) | 0.198 | 11.0 (-5.5, 27.5) |
| SGLT2i | 1.13 (0.73, 1.77) | 0.583 | 5.7 (-14.4, 25.8) |
| ACEi /ARB/ ARNI + Beta blocker + MRA | 1.10 (0.69, 1.75) | 0.696 | 4.0 (-15.7, 23.6) |
| ACEi/ARB/ARNI + Beta blocker + MRA + SGLT2i | 1.11 (0.57, 2.17) | 0.755 | 2.9 (-15.3, 21.1) |
| **Patient-level measures (self-reported)** | | | |
| Adherence to prescribed treatment | 1.07 (1.03, 1.12) | **<0.001** | 6.5 (3.1, 10.0) |
| Adherence to diet plan | 1.07 (1.02, 1.13) | **0.008** | 6.1 (1.6, 10.5) |
| Adherence to physical activity | 1.22 (1.15, 1.29) | **<0.001** | 16.8 (11.9, 21.6) |

## **Table H** Processes of care, health status, and treatment satisfaction at EOS - Using Inverse Probability Weighting

|  | **Mean difference (95% CI)** | **p value** |
| --- | --- | --- |
| **Clinic visits** | 2.30 (1.62, 2.98) | **<0.001** |
| **BP checked** | 2.93 (2.17, 3.70) | **<0.001** |
| **Cholesterol testing** | 0.77 (0.45, 1.10) | **<0.001** |
| **Blood sugar testing** | 1.61 (0.77, 2.44) | **<0.001** |
| **Electrocardiogram** | 0.17 (-0.03, 0.38) | 0.098 |
| **Global Physical Health Score** | 0.80 (-0.19, 1.79) | 0.113 |
| **Global Mental Health Score** | -0.10 (-1.17, 0.97) | 0.860 |
| **Combined Global Health Score** | 0.71 (-1.16, 2.58) | 0.457 |
| **Treatment satisfaction score** | 0.22(0.11, 0.33) | **<0.001** |
| **Satisfaction with current treatment** | 0.50 (0.37, 0.62) | **<0.001** |
| **Continue using current treatment** | 0.21 (0.07, 0.34) | **0.003** |
| **Recommend current treatment to someone else** | 0.36 (0.19, 0.54) | **<0.001** |

## **Table I** Clinical measures by treatment group at EOS - Using Inverse Probability Weighting

| **Clinical measures** | **Mean difference (95% CI)** | **p value** |
| --- | --- | --- |
| Systolic BP (mmHg) | -1.66 (-4.48, 1.14) | 0.246 |
| Diastolic BP (mmHg) | -1.30 (-3.08, 0.49) | 0.155 |
| Heart rate (beats/minute) | -0.23 (-1.82, 2.28) | 0.827 |
| Total cholesterol (mg/dL) | -2.48 (-10.50, 5.53) | 0.544 |
| HDL cholesterol (mg/dL) | -0.66 (-2.50, 1.18) | 0.483 |
| Triglycerides (mg/dL) | -3.63 (-15.26, 7.99) | 0.540 |
| LDL cholesterol (mg/dL) | 1.26 (-4.88, 7.39) | 0.688 |
| LDL cholesterol (mg/dL) (without HF) | 2.41 (-4.19, 9.00) | 0.474 |
| Fasting blood glucose (mg/dL) | 10.52 (-1.73, 22.76) | 0.092 |
| HbA1c (%) | -0.01 (-0.30, 0.28) | 0.968 |
| HbA1c (%) (with diabetes) | 0.01 (-0.47, 0.49) | 0.979 |
| Serum creatinine | 0.02 (-0.06, 0.10) | 0.661 |
| Weight (Kg) | -1.27 (-3.48, 0.94) | 0.261 |
| BMI (Kg/m2) | -0.69 (-1.48, 0.10) | 0.087 |

## **Table J** Site-stratified GDMT patterns between treatment groups

| **Medications use** | **Usual care (Control arm)** | | | | **C-QIP strategy (Intervention arm)** | | | |
| --- | --- | --- | --- | --- | --- | --- | --- | --- |
|  | **N** | **Baseline** | **N** | **EOS** | **N** | **Baseline** | **N** | **EOS** |
| **Site 01** | | | | |  |  |  |  |
| **CHD (n=51)** | | | | |  |  |  |  |
| Antiplatelet + Lipid lowering drug (statin) + ACEi/ARB + Beta Blocker | 23 | 15 (65.2%) | 23 | 14 (60.9%) | 28 | 21 (75.0%) | 28 | 19 (67.9%) |
| **Stroke (n=1)** | | | | |  |  |  |  |
| Antiplatelet + Lipid lowering drug (statin) + ACEi/ARB/Diuretic | 1 | 1 (100%) | 1 | 1 (100%) | 0 | - | 0 | - |
| **Heart failure (n=31)** | | | | |  |  |  |  |
| ACEi /ARB/ ARNI + Beta blocker + MRA | 17 | 9 (52.9%) | 17 | 8 (47.1%) | 14 | 11 (78.6%) | 14 | 6 (42.9%) |
| ACEi/ARB/ARNI + Beta blocker + MRA + SGLT2i | 17 | 6 (35.3%) | 17 | 4 (23.5%) | 14 | 9 (64.3%) | 14 | 3 (21.4%) |
| **Site 02** | | | | |  |  |  |  |
| **CHD (n=107)** | | | | |  |  |  |  |
| Antiplatelet + Lipid lowering drug (statin) + ACEi/ARB + Beta Blocker | 54 | 30 (55.6%) | 54 | 23 (42.6%) | 53 | 39 (73.6%) | 53 | 40 (75.5%) |
| **Stroke (n=0)** | | | | |  |  |  |  |
| **Heart failure (n=9)** | | | | |  |  |  |  |
| ACEi /ARB/ ARNI + Beta blocker + MRA | 5 | 4 (80%) | 5 | 4 (80%) | 4 | 3 (75.0%) | 4 | 2 (50.0%) |
| ACEi/ARB/ARNI + Beta blocker + MRA + SGLT2i | 5 | 4 (80%) | 5 | 4 (80%) | 4 | 3 (75.0%) | 4 | 2 (50.0%) |
| **Site 03** | | | | |  |  |  |  |
| **CHD (n=95)** | | | | |  |  |  |  |
| Antiplatelet + Lipid lowering drug (statin) + ACEi/ARB + Beta Blocker | 49 | 14 (28.6%) | 49 | 8 (16.3%) | 46 | 11 (23.9%) | 46 | 9 (19.6%) |
| **Stroke (n=0)** | | | | |  |  |  |  |
| **Heart failure (n=20)** | | | | |  |  |  |  |
| ACEi /ARB/ ARNI + Beta blocker + MRA | 7 | 2 (28.6%) | 7 | 3 (42.9%) | 13 | 3 (23.1%) | 13 | 3 (23.1%) |
| ACEi/ARB/ARNI + Beta blocker + MRA + SGLT2i | 7 | 1 (14.3%) | 7 | 2 (28.6%) | 13 | 1 (7.7%) | 13 | 1 (7.7%) |
| **Site 04** | | | | |  |  |  |  |
| **CHD (n=43)** | | | | |  |  |  |  |
| Antiplatelet + Lipid lowering drug (statin) + ACEi/ARB + Beta Blocker | 19 | 2 (10.5%) | 19 | 2 (10.5%) | 24 | 6 (25.0%) | 24 | 20 (83.3%) |
| **Stroke (n=73)** | | | | |  |  |  |  |
| Antiplatelet + Lipid lowering drug (statin) + ACEi/ARB/Diuretic | 43 | 14 (32.6%) | 43 | 13 (30.2%) | 30 | 18 (60.0%) | 30 | 23 (76.7%) |
| **Heart failure (n=22)** | | | | |  |  |  |  |
| ACEi /ARB/ ARNI + Beta blocker + MRA | 8 | 1 (12.5%) | 8 | 1 (12.5%) | 14 | 3 (21.4%) | 14 | 7 (50.0%) |
| ACEi/ARB/ARNI + Beta blocker + MRA + SGLT2i | 8 | 2 (25.0%) | 8 | 2 (25.0%) | 14 | 2 (14.3%) | 14 | 4 (28.6%) |

*GDMT=guideline directed medical therapy

## **Table K** Summary of changes from the statistical analysis plan

| **No.** | **SAP Specification** | **Actual Analysis** | **Reasons for clarification/deviation** |
| --- | --- | --- | --- |
| 1 | The baseline difference between the groups for the continuous variables will be assessed using t-test or Wilcoxon-rank sum test and for the categorical variables chi-square test will be used. (Baseline analysis; Page no. 9) | Baseline characteristics were presented descriptively by randomized group without reporting p values. | Although SAP-specified baseline significance testing was performed as an internal check and did not identify meaningful between-group differences, we did not report baseline p values in line with reviewer guidance and CONSORT principles for randomized trials. Randomization balances measured and unmeasured characteristics in expectation; therefore, baseline characteristics are presented descriptively rather than interpreted using statistical tests. |
| 2 | The adjusted model will include the treatment group, time as a categorical variable, a treatment-by-time interaction and the baseline values, age, sex and other covariates which will be identified as significant at baseline comparisons. (Adjusted Analyses- For Secondary and Exploratory outcomes; Page no. 12) | Final adjusted models included treatment group, time, age, sex, and site as a fixed effect. | Covariates were not selected based on baseline statistical significance. Although internal checks did not identify meaningful baseline differences between groups, the adjusted models used clinically and methodologically relevant covariates specified in the protocol/SAP, consistent with Reviewer 1’s recommendation to avoid data-driven covariate selection. We checked for the treatment-by-time interaction, since it wasn’t significant, we did not include it the model. |
| 3 | For both secondary and exploratory outcomes, the model will include the following terms: treatment group, time as categorical variable, a treatment by time interaction and the baseline values. (Exploratory analyses: Mean change in systolic blood pressure and LDL-cholesterol; Page no. 11) | Final adjusted models included age, sex, and site (as a fixed effect) | We checked for the treatment-by-time interaction, since it wasn’t significant, we did not include it the model. |
| 4 | To account for within-subject correlations, we will assume that errors are correlated according to compound-symmetry covariance structure and will use Generalized Estimating Equations (GEE) (Zeger and Liang, 1988). (Exploratory analyses: Mean change in systolic blood pressure and LDL-cholesterol; Page no. 11) | Generalized Estimating Equations (GEE) were used with an interchangeable (exchangeable) correlation structure. | With two time points, compound symmetry and interchangeable correlation structures are equivalent; therefore, no substantive deviation was introduced. |
| 5 | The analysis will be according to the intention to treat principle. The baseline characteristics of the participants who completed and did not complete the final visit will be compared. We do not plan to impute the missing data for the secondary outcomes (Missing data handling; Page no. 13) | We have included the table for baseline characteristics of participants who completed and did not complete the baseline visits. (**see Table S6**)  As per the reviewers’ comment, we have now included the sensitivity analysis by using Inverse Probability Treatment Weighting technique to account for missing data at the final visit. (**see Tables S7- S9**) | No sensitivity analysis was planned for secondary outcomes initially. |
| 6 | We will also test pairwise differences between treatment groups for each visit separately. (Exploratory analyses: Mean change in systolic blood pressure and LDL-cholesterol; Page no. 11) | Analyses were restricted to baseline and end-of-study visits; therefore, pairwise comparisons at intermediate visits were not performed. | As the final analysis was restricted to baseline and end-of-study visits, pairwise comparisons at intermediate time points were not applicable. |
| 7 | Time points for analysis | All primary, secondary and exploratory outcomes were analysed at end-of-study visit. | The final analysis compared all implementation-related and clinical outcomes at the end-of-study visits. |
| 8 | Trial-based cost analysis | Not reported | Economic evaluation is being analysed separately and will be reported in a future manuscript. |
| 9 | Qualitative CFIR-based implementation evaluation | Not reported as a trial outcome in this manuscript | CFIR-informed qualitative work was used to support intervention co-design/contextual interpretation and has been reported separately as formative work; additional qualitative/mixed-methods findings, using RE-AIM framework, will be reported separately. |
