# Supplementary material for: Assessing a multicomponent quality improvement strategy for chronic care of cardiovascular diseases in India: The C-QIP randomized feasibility trial
Source: PLoS Med. 2026 Sep 29;23(9):e1004932. doi: 10.1371/journal.pmed.1004932 (PMC13641716; doi:10.1371/journal.pmed.1004932)
Supplement: Supplementary file 8 [file pmed.1004932.s009.docx]

**PLOS MEDICINE — PEER REVIEW**

**Confidential Comments to the Editor**

PMEDICINE-D-26-00187R1

**Recommendation: MAJOR REVISION**

# Overall Assessment

The C-QIP trial is a well-motivated and methodologically coherent feasibility RCT of a multicomponent cardiovascular quality improvement strategy across four Indian outpatient sites. The study was prospectively registered, the SAP was finalised prior to data lock, and the protocol is published (American Heart Journal, 2024). High retention (>91% both arms), prospective measurement of implementation outcomes, and clinically meaningful GDMT improvements in the coronary heart disease and ischemic stroke subgroups represent genuine strengths.

However, the manuscript has substantive deficiencies in data integrity, SAP adherence, and selective outcome reporting that prevent recommendation for publication in its current form. Major revision is warranted.

# Principal Concerns for the Editor's Attention

## 1. Data Integrity

There are multiple numerical inconsistencies between the abstract, main text, Table 1, Table 2, and Figure 1 — including the reported follow-up duration (18 months in abstract vs. ~15 months observed), CVD condition prevalences, and trial flow counts. These raise concerns about the rigour of cross-checking during manuscript preparation and should be verified against the locked dataset before publication.

## 2. Non-disclosure of SAP Deviations

Comparison of the pre-specified SAP with the manuscript reveals at least four deviations: (i) the treatment-by-time interaction term specified in the GEE model appears to have been omitted; (ii) the EOS data reallocation algorithm is undefined in both the SAP and the manuscript; (iii) three pre-specified subgroup variables are absent from the subgroup analyses; and (iv) the handling of 19 deceased participants in the ITT GEE analyses is not described. None of these deviations are declared anywhere in the manuscript. PLOS Medicine's transparency standards require explicit disclosure of all departures from the pre-specified analysis plan.

## 3. Validity of Effect Estimates for Future Trial Planning

Individual-level randomisation within shared outpatient clinics creates a plausible contamination risk (providers reviewing DSS prompts for C-QIP participants may alter care for co-located controls). No quantitative assessment of contamination is presented. The largest reported effect — RR=2.41 for ischemic stroke GDMT bundle (n=74, just 30 C-QIP and 44 control patients) — is likely to be used to power a future confirmatory hybrid trial. If contamination has inflated this estimate, the confirmatory trial may be substantially underpowered. The authors must address this with a sensitivity analysis or structured quantitative discussion.

## 4. Selective Reporting of Pre-specified Outcomes

The SAP and protocol both pre-specify a cost analysis and a qualitative component (CFIR-based appropriateness interviews) as study outcomes. Neither is reported in this manuscript, nor is their omission acknowledged. Under PLOS Medicine's outcome reporting standards, authors must either present these data or provide a clear statement of their status (e.g., reported separately, data not yet analysed).

# Summary

The underlying science is sound and the clinical question is important. The concerns identified are addressable. I recommend inviting a major revision with a detailed author response to the issues raised, and I am willing to re-review the revised manuscript.

*End of Confidential Comments to the Editor*
