# Supplementary material for: Assessing a multicomponent quality improvement strategy for chronic care of cardiovascular diseases in India: The C-QIP randomized feasibility trial
Source: PLoS Med. 2026 Sep 29;23(9):e1004932. doi: 10.1371/journal.pmed.1004932 (PMC13641716; doi:10.1371/journal.pmed.1004932)
Supplement: Supplementary file 9 [file pmed.1004932.s011.docx]

**Review Comments**

**Comments from the academic editor:**

The departure from the initial statistical analysis plan is a serious concern, and how the authors address this will be fundamental.

**Response:** We thank the Academic Editor for highlighting this important issue. We agree that any departures from the prespecified statistical analysis plan (SAP) should be transparently reported and justified. We have therefore revised the Statistical Analysis section and referred to the supplement that provides “Clarifications and deviations from the prespecified SAP”. In this section, we now explicitly describe: (i) the prespecified SAP approach (SAP specification); (ii) analyses implemented (Actual Analysis); (iii) Reasons for deviation in response to reviewer concerns. We have added a supplementary table S11 comparing SAP-specified analyses with the final manuscript analyses, including the rationale for each deviation. (**Page 10**).

**Supplementary Table S11. Summary of changes from the statistical analysis plan and justification**

| **No.** | **SAP Specification** | **Actual Analysis** | **Reasons for deviation** |
| --- | --- | --- | --- |
| 1 | The baseline difference between the groups for the continuous variables will be assessed using t-test or Wilcoxon-rank sum test and for the categorical variables chi-square test will be used. (Baseline analysis; Page no. 9) | Baseline characteristics were presented descriptively by randomized group without reporting p values. | Although SAP-specified baseline significance testing was performed as an internal check and did not identify meaningful between-group differences, we did not report baseline p values in line with reviewer guidance and CONSORT principles for randomized trials. Randomization balances measured and unmeasured characteristics in expectation; therefore, baseline characteristics are presented descriptively rather than interpreted using statistical tests. |
| 2 | The adjusted model will include the treatment group, time as a categorical variable, a treatment-by-time interaction and the baseline values, age, sex and other covariates which will be identified as significant at baseline comparisons. (Adjusted Analyses- For Secondary and Exploratory outcomes; Page no. 12) | Final adjusted models included treatment group, time, age, sex, and site as a fixed effect. | Covariates were not selected based on baseline statistical significance. Although internal checks did not identify meaningful baseline differences between groups, the adjusted models used clinically and methodologically relevant covariates specified in the protocol/SAP, consistent with Reviewer 1’s recommendation to avoid data-driven covariate selection. We checked for the treatment-by-time interaction, since it wasn’t significant, we did not include it the model. |
| 3 | For both secondary and exploratory outcomes, the model will include the following terms: treatment group, time as categorical variable, a treatment by time interaction and the baseline values. (Exploratory analyses: Mean change in systolic blood pressure and LDL-cholesterol; Page no. 11) | Final adjusted models included age, sex, and site (as a fixed effect) | We checked for the treatment-by-time interaction, since it wasn’t significant, we did not include it the model. |
| 4 | To account for within-subject correlations, we will assume that errors are correlated according to compound-symmetry covariance structure and will use Generalized Estimating Equations (GEE) (Zeger and Liang, 1988). (Exploratory analyses: Mean change in systolic blood pressure and LDL-cholesterol; Page no. 11) | Generalized Estimating Equations (GEE) were used with an interchangeable (exchangeable) correlation structure. | With two time points, compound symmetry and interchangeable correlation structures are equivalent; therefore, no substantive deviation was introduced. |
| 5 | The analysis will be according to the intention to treat principle. The baseline characteristics of the participants who completed and did not complete the final visit will be compared. We do not plan to impute the missing data for the secondary outcomes (Missing data handling; Page no. 13) | We have included the table for baseline characteristics of participants who completed and did not complete the baseline visits. (**see Table S6**)  As per the reviewers’ comment, we have now included the sensitivity analysis by using Inverse Probability Treatment Weighting technique to account for missing data at the final visit. (**see Tables S7- S9**) | No sensitivity analysis was planned for secondary outcomes initially. |
| 6 | We will also test pairwise differences between treatment groups for each visit separately. (Exploratory analyses: Mean change in systolic blood pressure and LDL-cholesterol; Page no. 11) | Analyses were restricted to baseline and end-of-study visits; therefore, pairwise comparisons at intermediate visits were not performed. | As the final analysis was restricted to baseline and end-of-study visits, pairwise comparisons at intermediate time points were not applicable. |
| 7 | Time points for analysis | All primary, secondary and exploratory outcomes were analysed at end-of-study visit. | The final analysis compared all implementation-related and clinical outcomes at the end-of-study visits. |
| 8 | Trial-based cost analysis | Not reported | Economic evaluation is being analysed separately and will be reported in a future manuscript. |
| 9 | Qualitative CFIR-based implementation evaluation | Not reported | CFIR-informed qualitative work was used to support intervention co-design/contextual interpretation and has been reported separately as formative work; additional qualitative/mixed-methods findings, using RE-AIM framework, will be reported separately. |

-----------------------------------------------------------

**Comments from the reviewers:**

**Reviewer #1: Major Comments**

1) The statistical analysis is described as confirmatory in several sections (e.g., use of hypothesis testing with a fixed α=0.05), despite the trial being explicitly positioned as a feasibility and hybrid type 1 implementation study. For feasibility trials, emphasis should be placed on estimation (e.g., point estimates with confidence intervals) rather than formal hypothesis testing. The current analytical framing risks overstating inferential claims that the study is not powered to support.

**Response:** Thank you for your comment. We have revised the manuscript throughout to avoid confirmatory language and to emphasize estimation. The trial was designed as a feasibility randomized trial with implementation outcomes as the primary outcomes. We now present estimates with 95% CIs as the primary analytic output and interpret p values descriptively rather than as definitive measures of significance.

2) The SAP indicates that baseline differences between randomised groups will be tested using significance tests (t-tests, Wilcoxon tests, chi-square tests). This practice is discouraged in randomised trials, as any observed imbalance is due to chance rather than bias. Baseline characteristics should be presented descriptively without hypothesis testing, and any covariate adjustment should be prespecified based on clinical relevance rather than statistical significance at baseline.

**Response:** Thank you for your comment. Although the SAP included baseline significance testing, we did not report baseline p values in the manuscript **Table 1** and only describe baseline characteristics descriptively without any significance test. Randomization should balance measured and unmeasured baseline characteristics of study participants.

We have added a short amendment to the SAP as clarification to the reviewer’s comments. (**Table S11**).

3) For secondary outcomes, covariates included in adjusted models are described as those "identified as significant at baseline comparisons." This data-driven approach introduces a risk of overfitting and post-randomisation bias. Covariates for adjustment should be prespecified a priori based on clinical or methodological rationale, not on baseline p-values.

**Response:** Thank you for your comment. To clarify, although it was mentioned in the SAP, the primary adjusted models included only prespecified clinically and methodologically relevant covariates. Final adjusted models included treatment group, time, age, sex, and site as a fixed effect. The protocol and SAP both included age, sex and site as core adjustment variables. We confirm that no covariates were selected on the basis of baseline statistical significance. (**Table S11**).

4) The choice of log-binomial GEE models for binary outcomes is appropriate in principle; however, the SAP lacks sufficient detail regarding the assumed working correlation structure, robustness of standard errors, and how clustering by site (four clinics) will be handled. Given the multi-site design, failure to explicitly address site-level clustering may result in underestimated uncertainty.

**Response:** We agree and have clarified the modelling approach. For repeated measures within participants, we used GEE with an exchangeable correlation structure with robust standard errors. Site was included as a fixed effect because the trial included only four sites, making random-effects estimation of between-site variance unstable. (**Table S11**).

5) The plan to reallocate "end of study" measurements to scheduled visits (12 or 24 months) is insufficiently justified and may introduce measurement error or misclassification of follow-up time. A clearer definition of the estimand and handling of variable follow-up duration is required, particularly given the stated average follow-up of ~18 months.

**Response:** We agree that the prior wording was confusing or the end-of-study (EOS) visits calculation was overstated in the SAP description. We have clarified that the primary analysis time point for this manuscript is the end-of-study visit, defined as the final study assessment completed between July and October 2024 among active participants. We no longer describe the EOS data as reallocated to 12- or 24-month visits in the main analysis. **(page 7)**

The observed median follow-up was similar between the treatment groups, i.e., 15.3 months (or 459 days) in C-QIP and 15.1 months (452 days) in usual care, whereas the SAP referred to an expected average follow-up of approximately 18 months under the planned variable follow-up design. (**page 10**).

6) Missing data are stated to be handled via complete-case analysis, with no imputation planned. This assumption implicitly requires data to be missing completely at random (MCAR), which is unlikely in longitudinal behavioural and clinical outcomes. At minimum, the authors should justify the plausibility of MCAR, report the extent and patterns of missingness, and consider sensitivity analyses (e.g., multiple imputation or inverse probability weighting).

**Response:** Thank you for your comment. In the manuscript, we have mentioned that ITT approach was used for the primary analysis not complete case analysis. We had relatively small numbers (n=22, 5.4%) of missing data for primary, and secondary outcomes. GEE analysis performed as the main analysis for secondary outcomes assumes that data is MCAR (missing completely at random). Additionally, we have provided a missing-data table showing the extent and pattern of missingness by baseline characteristics. We also added sensitivity analyses using inverse probability weighting (IPTW) for secondary and exploratory outcomes in the **supplementary Tables S6-S9**.

7) Multiple secondary and exploratory outcomes are analysed without any discussion of multiplicity or control of false-positive findings. Even in feasibility studies, the lack of a clear hierarchy or interpretive framework for multiple comparisons limits the credibility of reported p-values.

**Response:** Thank you for your comment. We now clarify that the trial was not designed to test a confirmatory effectiveness hypothesis and that secondary and exploratory outcomes are interpreted as estimation-generating. No formal multiplicity adjustment was applied; instead, we emphasize effect sizes, CIs, consistency and plausibility across related outcomes.

We did not apply formal adjustment for multiplicity because C-QIP was a feasibility trial designed to estimate implementation performance and preliminary effect sizes rather than to test confirmatory hypotheses. Findings across secondary and exploratory outcomes are interpreted cautiously, with emphasis on magnitude, precision, and consistency rather than isolated p values.

8) Subgroup analyses are extensive and include numerous patient characteristics. Although the SAP notes that the study is not powered for these analyses, there is no clear guidance on how these results will be interpreted or reported. Given the high risk of spurious findings, subgroup analyses should be substantially reduced, clearly labelled as exploratory, and interpreted strictly qualitatively.

**Response:** Thank you for your comment. Both the subgroup analyses are clearly labelled as exploratory and hypothesis-generating, and presented in the supplement. We only report prespecified subgroup analysis results for medication adherence and systolic BP, as stated in the SAP. The SAP prespecified subgroup analyses for adherence and systolic BP and noted that results should be treated with caution because the study was not powered for subgroup analyses.

9) The integration of qualitative and quantitative data for implementation outcomes ("data triangulation") is conceptually appropriate, but the statistical analysis plan does not specify how qualitative findings will be systematically incorporated alongside quantitative estimates. A clearer mixed-methods analytical framework is needed to ensure transparency and reproducibility.

**Response:** Thank you for your comment. We clarify that the present manuscript reports quantitative findings from the randomized feasibility trial, including implementation outcomes, GDMT prescribing, adherence, care processes, and exploratory clinical measures. We do not report a formal mixed-methods integration or triangulation analysis in this manuscript. The qualitative CFIR-guided and RE-AIM-informed process evaluation addresses separate implementation questions and will be reported separately. We have revised the Methods: study outcomes section to make this distinction explicit. **(page 8).**

**Minor Comments**

10) The SAP alternates between describing outcomes at 12 months, 24 months, and "end of study," which creates ambiguity regarding the primary time point for analysis. Consistent terminology and a clearly defined primary analysis time point would improve clarity.

**Response:** The primary analysis time point is now clearly defined as end of study visit (EOS). **(page 7)**

11) The use of compound symmetry as the assumed covariance structure in GEE models is stated without justification. The authors should explain why this structure is appropriate or indicate whether alternative structures were explored.

**Response:** We assumed compound symmetry as it provides unbiased estimates. Since we only have two timepoints (baseline and end-of-study visit timepoints), exchangeable correlation structure is the method of choice. **(Table S11)**

12) For continuous outcomes (e.g., systolic blood pressure, LDL cholesterol), it is unclear whether model assumptions (e.g., normality, linearity) will be assessed and how violations will be handled.

**Response:** We checked for the linear model assumptions, and added details to the statistical analysis section. **(page 9).**

13) The SAP states that pairwise comparisons between treatment groups at each visit will be performed, but it does not clarify whether these are prespecified contrasts or post hoc analyses, nor how they relate to the primary treatment-by-time interaction.

**Response:** To clarify, for final analysis, we only considered baseline and EOS visits, therefore, no pair-wise comparisons were needed. We have now added a note in the SAP as a supplement, and detailed Table presenting deviations from the SAP and corresponding justifications. **(Table S11).**

14) Reporting of effect sizes is inconsistent across outcomes. A standardised approach (e.g., risk ratios with 95% confidence intervals for binary outcomes; mean differences with 95% confidence intervals for continuous outcomes) should be explicitly stated.

**Response:** Thank you for flagging this. We now consistently report relative risk (RRs) or risk differences with 95% CIs for binary outcomes and mean differences with 95% CIs for continuous outcomes. **(page 9 and updated Figure 4).**

15) The description of safety analyses is minimal and largely descriptive. Clarification is needed on whether any comparative safety analyses between groups are planned and how exposure time will be accounted for.

**Response:** Serious adverse events are reported descriptively by arm. Because C-QIP was a feasibility trial with low event counts, no formal comparative safety testing was planned as the safety endpoints were not the primary outcomes and trial was not powered for clinical endpoints based comparative analysis.

16) Although STATA version 18 is specified, no details are provided on reproducibility practices (e.g., prespecified analysis scripts, version control, or audit trails), which are increasingly expected in contemporary clinical trials.

**Response:** To clarify, all analyses were performed using prespecified STATA scripts, retained with version logs and independently checked by the study statistician. A permanent archived copy of analysis code will be made available subject to data-sharing and ethics approvals. **(Page 10).**

-----------------------------------------------------------------------------------------

**Reviewer #2: Summary**

This manuscript reports the C-QIP feasibility RCT, evaluating a five-component quality improvement strategy (cardiovascular care coordinator, EHR-DSS, SMS reminders, patient diary/VITA, and audit-feedback) for chronic CVD care across four Indian outpatient sites (n=410; mean follow-up ~15 months). The study addresses an important implementation gap in LMIC cardiovascular care and is appropriately scoped as a feasibility trial. High retention, strong fidelity, and meaningful GDMT improvements in the coronary heart disease and ischemic stroke subgroups are commendable. However, several methodological and reporting concerns require revision before the manuscript is suitable for publication.

Major Concerns

1. Internal Numerical Inconsistencies

1.      The abstract states 'mean follow-up 18 months', but the results report a median of 15.3/15.1 months (C-QIP/usual care) and Figure 1 implies ~459/452 days. The SAP's 18-month figure is an expected mean under a variable follow-up design; the observed value differed. The abstract must be corrected and the discrepancy acknowledged.

**Response:** Thank you for your comment. We agree and have corrected the abstract. The observed median follow-up was approximately 15 months, and expected mean follow-up was 18 months, as reported in the SAP. **(page no. 2).**

2.      The abstract reports ischemic stroke 18.5% and heart failure 18.0%, but Table 1 shows HF 22.7%/18.2% and stroke 15.2%/21.7% by arm. These figures must be reconciled with the subgroup Ns in Table 2 (stroke N=74, HF N=82) throughout.

**Response:** Thank you for your comment. Overall % for ischemic stroke and heart failure was 18.4% and 20.4%, respectively. **Table 1** reports data by treatment group: heart failure in 45/198 C-QIP and 37/203 usual care participants, and ischemic stroke in 30/198 and 44/203 participants. The total denominators used for subgroups in **Table 2** are now clearly reported as ischemic stroke n=74/401 and heart failure n=82/401.

3.      Figure 1 lists 11 vs 1 dropouts and 9 vs 10 deaths, but the text implies different totals. A CONSORT-compatible flow diagram with transparent accounting of all post-randomisation losses is required.

**Response:** Thank you for your comment. We have re-checked **Figure 1** CONSORT flow diagram regarding total number of screened, ineligible, randomized, baseline assessed, EOS assessed, deaths, and withdrawals/lost to follow-up included in each analysis. We have added reasons for ineligibility in **Figure 1**. The number of dropout and deaths reported in the Figure 1 and main text are consistent and matching. **(Page 10).**

2. Contamination Risk

Individual-level randomisation within shared outpatient clinics creates a plausible and unquantified contamination risk: providers reviewing DSS prompts for C-QIP patients may modify care for controls attending the same clinics. The manuscript acknowledges this but provides no quantitative assessment (e.g., site-stratified comparison of control-arm GDMT rates). Given that future confirmatory trials will be powered on these effect estimates — notably the RR=2.41 for ischemic stroke GDMT (n=74) — overestimation due to contamination is a critical issue. A sensitivity analysis or, at minimum, a structured discussion of the likely direction and magnitude of bias is required.

**Response:** We agree that individual randomization within shared clinics could have introduced contamination. The most plausible direction of such contamination would be attenuation (underestimation or dilution) of the observed between-group effect, because provider learning or spill-over from C-QIP intervention strategy could improve prescribing for usual-care participants. To explore this possibility, we added site-stratified GDMT rates comparing baseline and end-of-study prescribing patterns in usual care and C-QIP groups. Usual-care GDMT did not show consistent improvement across sites, which does not suggest substantial contamination; however, these descriptive analyses are limited by small site-specific denominators. We therefore interpret effect estimates cautiously and recommend cluster randomization in a future confirmatory trial.

**Table S.10.** Site-stratified GDMT patterns between treatment groups at baseline and EOS.

| **Medications use** | **Usual care** | | | | **C-QIP strategy** | | | |
| --- | --- | --- | --- | --- | --- | --- | --- | --- |
|  | **N** | **Baseline** | **N** | **EOS** | **N** | **Baseline** | **N** | **EOS** |
| **Site 01** | | | | |  |  |  |  |
| **CHD (n=51)** | | | | |  |  |  |  |
| Antiplatelet + Lipid lowering drug (statin) + ACEi/ARB + Beta Blocker | 23 | 15 (65.2%) | 23 | 14 (60.9%) | 28 | 21 (75.0%) | 28 | 19 (67.9%) |
| **Stroke (n=1)** | | | | |  |  |  |  |
| Antiplatelet + Lipid lowering drug (statin) + ACEi/ARB/Diuretic | 1 | 1 (100%) | 1 | 1 (100%) | 0 | - | 0 | - |
| **Heart failure (n=31)** | | | | |  |  |  |  |
| ACEi /ARB/ ARNI + Beta blocker + MRA | 17 | 9 (52.9%) | 17 | 8 (47.1%) | 14 | 11 (78.6%) | 14 | 6 (42.9%) |
| ACEi/ARB/ARNI + Beta blocker + MRA + SGLT2i | 17 | 6 (35.3%) | 17 | 4 (23.5%) | 14 | 9 (64.3%) | 14 | 3 (21.4%) |
| **Site 02** | | | | |  |  |  |  |
| **CHD (n=107)** | | | | |  |  |  |  |
| Antiplatelet + Lipid lowering drug (statin) + ACEi/ARB + Beta Blocker | 54 | 30 (55.6%) | 54 | 23 (42.6%) | 53 | 39 (73.6%) | 53 | 40 (75.5%) |
| **Stroke (n=0)** | | | | |  |  |  |  |
| **Heart failure (n=9)** | | | | |  |  |  |  |
| ACEi /ARB/ ARNI + Beta blocker + MRA | 5 | 4 (80%) | 5 | 4 (80%) | 4 | 3 (75.0%) | 4 | 2 (50.0%) |
| ACEi/ARB/ARNI + Beta blocker + MRA + SGLT2i | 5 | 4 (80%) | 5 | 4 (80%) | 4 | 3 (75.0%) | 4 | 2 (50.0%) |
| **Site 03** | | | | |  |  |  |  |
| **CHD (n=95)** | | | | |  |  |  |  |
| Antiplatelet + Lipid lowering drug (statin) + ACEi/ARB + Beta Blocker | 49 | 14 (28.6%) | 49 | 8 (16.3%) | 46 | 11 (23.9%) | 46 | 9 (19.6%) |
| **Stroke (n=0)** | | | | |  |  |  |  |
| **Heart failure (n=20)** | | | | |  |  |  |  |
| ACEi /ARB/ ARNI + Beta blocker + MRA | 7 | 2 (28.6%) | 7 | 3 (42.9%) | 13 | 3 (23.1%) | 13 | 3 (23.1%) |
| ACEi/ARB/ARNI + Beta blocker + MRA + SGLT2i | 7 | 1 (14.3%) | 7 | 2 (28.6%) | 13 | 1 (7.7%) | 13 | 1 (7.7%) |
| **Site 04** | | | | |  |  |  |  |
| **CHD (n=43)** | | | | |  |  |  |  |
| Antiplatelet + Lipid lowering drug (statin) + ACEi/ARB + Beta Blocker | 19 | 2 (10.5%) | 19 | 2 (10.5%) | 24 | 6 (25.0%) | 24 | 20 (83.3%) |
| **Stroke (n=73)** | | | | |  |  |  |  |
| Antiplatelet + Lipid lowering drug (statin) + ACEi/ARB/Diuretic | 43 | 14 (32.6%) | 43 | 13 (30.2%) | 30 | 18 (60.0%) | 30 | 23 (76.7%) |
| **Heart failure (n=22)** | | | | |  |  |  |  |
| ACEi /ARB/ ARNI + Beta blocker + MRA | 8 | 1 (12.5%) | 8 | 1 (12.5%) | 14 | 3 (21.4%) | 14 | 7 (50.0%) |
| ACEi/ARB/ARNI + Beta blocker + MRA + SGLT2i | 8 | 2 (25.0%) | 8 | 2 (25.0%) | 14 | 2 (14.3%) | 14 | 4 (28.6%) |

3. Deviations from the Pre-specified SAP

The SAP specifies a treatment-by-time interaction term in all GEE models; this appears absent from the analyses as reported. Any departure from the pre-specified model must be disclosed.

**Response:** Thank you for your comment. We checked for interaction term between treatment group and time and did not find it significant between treatment groups-by-time. Therefore, the results are reported without including the treatment-by-time interaction term. **(Table S11)**

The SAP describes reallocating EOS data to a scheduled visit timepoint but does not define the algorithm; the manuscript does not clarify this either. Please explain how EOS data were handled.

**Response:** Thank you for your comment. We have removed ambiguous language around reallocation. The primary analysis uses EOS as the final observed study assessment. We have now revised the methods section to provide a clear explanation for the EOS visit definition. **(Page 7).**

Three pre-specified subgroup variables (education, income, presenting condition) are absent from Figures S.2-S.3 without explanation.

**Response:** We checked the **Figures S.2-S.3** and noted that these three pre-specified subgroup variables are already reported.

No imputation was planned for continuous outcomes, but the handling of the 19 deceased participants in GEE ITT analyses is not described. A sensitivity analysis is warranted.

**Response:** Thank you for your comment. We now clarify that deceased participants contributed data until death and were included in safety outcomes. We have added a sensitivity analysis using IPWT in the supplement to account for missing data (**Tables S7-S9**).

4. Heart Failure GDMT Inconsistency

The Methods define HF GDMT as a four-drug bundle (ACEi/ARB/ARNI + beta-blocker + MRA + SGLT2i), yet the abstract and primary results focus on the three-drug bundle, omitting SGLT2i. The null finding for SGLT2i (42.2% vs 43.2%, RR=1.28, p=0.237) — a now-essential HFrEF therapy — should be explicitly reported and discussed. Potential explanations (cost, availability, formulary access in India) should be addressed.

**Response:** Thank you for your comment. To clarify, previously in the abstract and results, we did not describe HF GDMT by three or four drug classes as the GDMT rates were no different between the treatment groups. We have now revised the manuscript to report four-drug bundle for HF GDMT including SGLT2i. We have also added a brief discussion noting that limited improvement in SGLT2 inhibitor use may reflect the small heart failure subgroup, evolving guideline implementation during the trial period, and potential cost, availability, and formulary-access barriers in routine outpatient care in India. (**page nos. 11 and 15**)

Minor Concerns

*       Abstract exceeds the 300-word PLOS Medicine limit (currently 418 words); condensation is required.

**Response:** Abstract is now shortened to <300 words. (**page 2**)

*       Alcohol counselling fidelity fell from 83% to 33% at EOS (Table S.1, n=6): this striking decline contradicts the high-fidelity narrative and requires explanation.

**Response:** Thank you for your comment. Since, alcohol moderation counselling was assessed only among participants reporting alcohol use; the EOS denominator was small (n=6), making this estimate unstable and should not be generalized. (**Table S1**)

*       The direction of effect for the physical health T-score appears contradictory: mean 46.9 (C-QIP) vs 48.6 (usual care), yet beta=+0.95 in favour of C-QIP. Please verify and clarify.

**Response:** Thank you for your comment. Please note, the mean physical health T-score for C-QIP id 48.6 and for usual care it is 46.9, the direction of coefficient is in line with this (as reported in **Figure 3**).

*       Significant subgroup interactions (private facility, ischemic stroke) are mentioned but not quantified in the text; numerical estimates and a brief discussion of their implications for scale-up are needed.

**Response:** Thank you for your comment. We have now added numerical estimates and a brief discussion of their implications for scale-up, but we are cautious in terms of making strong claims based on this feasibility trial findings. **(page 12)**

*       The SAP pre-specifies cost analysis and a qualitative (CFIR-based) component as outcomes. Neither is reported, nor is their omission acknowledged. Please clarify the reporting status of both.

**Response:** Thank you for this important observation. We agree that the manuscript should explicitly clarify the reporting status of the cost analysis and qualitative CFIR-based component that were described in the statistical analysis plan. We have revised the Methods **(page 8)** to make clear that the present manuscript focuses on the randomized feasibility trial outcomes: feasibility, fidelity, adoption, acceptability, GDMT prescribing, adherence, care processes, and exploratory clinical measures. The cost analysis and qualitative CFIR-based implementation evaluation were not included in this manuscript because they address distinct research questions and require separate analytic methods and reporting standards. The qualitative CFIR-informed work was used to inform the co-design of the C-QIP strategy and has been reported separately as part of the formative phase ([Singh et. al *Global Heart 2022*](https://pubmed.ncbi.nlm.nih.gov/36382156/)). The trial-based cost analysis is being analysed separately and will be reported in a future economic evaluation. The manuscript already notes that the present analysis primarily estimates implementation outcomes and preliminary effects for process and clinical outcomes. We have now added a clarification to the Methods (**Page 8**).

*       The screening-to-randomisation data in Figure 1 lack detail on reasons for ineligibility; this is standard for feasibility trial reporting (cf. Eldridge et al., CONSORT 2016 extension for pilot/feasibility trials).

**Response:** Thank you for your comment. We have now added main reasons for ineligibility to **Figure 1.**

*       'Poison regression' in the SAP (page 10) should read 'Poisson'; please confirm this typographical error is not carried into the manuscript.

**Response:** We confirm that Poisson regression is correctly spelled in this manuscript. **(Page 9)**

**Reviewer #3:**

This is a well presented RCT with 410 participants of a QI strategy for cardiovascular health in India. I have the following suggestions/concerns/recommendations.

**Response:** Thank you for your constructive feedback.

*       Abstract has no aim. Not ideal in abstracts to say "implementation outcomes were "strong" - I recommend just putting the data with a direction rather than persuasive language. The measures of retention and fidelity seem to be a % but no outcome measure so cannot understand what the numbers mean. Also, how many patients received lifestyle advice appears to be int eh results. I find the abstract somewhat biased in its reporting. Although conclusion seems to reflect the findings.

**Response:** Thank you for your comment. We have revised the abstract to include a clear aim and removed persuasive language such as “strong.” We now present implementation results neutrally with numerators/denominators and include limitations. **(page 2)**

*       Suggest an overview of the intervention be provided in a table as the current text is dense. The intervention is very complex which is 'ok' but does make it a little complex to replicate given the current text. Perhaps also consider using the Tidier checklist or similar?

**Response:** Thank you for your excellent suggestions. We have added to the supplement, a TIDieR-style table and checklist summarizing the five intervention components: 1.) CCC-facilitated care, 2) EHR-DSS, 3) SMS reminders, 4) patient diary/VITA, and 5) audit-feedback.

**Supplement file 1.** Description of C-QIP strategy components

| C-QIP Component | Target | Who delivered | Frequency | Materials/tools | Intended mechanism | Fidelity indicator |
| --- | --- | --- | --- | --- | --- | --- |
| 1.CCC-facilitated care | Patients | CCC | Monthly/quarterly as per patients’ follow-up plan | CCC training manual | - Regular follow-ups, Reminders  - Coordinated care | - Intervention delivery % |
| 2.EHR-DSS | Physicians | Physician and CCC | Monthly/quarterly as per patients’ follow-up plan | EHR records | - To maintain all records centrally in one place | - prompt acceptance and rejection % |
| 3. SMS reminder | Patients | Automatic text messages | Daily | Text messages in regional languages | - Information  - Reminders | - patient acknowledge receipt of SMS |
| 4.Patient diary | Patients | CCC | Patient Diary - Monthly/quarterly as per patients’ follow-up plan  VITA tool - Daily | Book and calendar templates | - Information  - Medicine adherence | VITA tool completion |
| 5.Audit-feedback | Physician and CCC | Research Team | Quarterly | Reports | - Performance Review  - PDSA plan | PDSA plan completion |

*CCC=cardiovascular care coordinator, EHR-DSS=electronic health records and decision support system, SMS=short message service

*       I have some concerns about individual patient level randomisation when intervention was a hospital/practice level - how was cross-over managed?

**Response:** The individual-level randomized design was selected for this feasibility trial to maximize recruitment efficiency within four outpatient clinics, balance patient-level characteristics, and evaluate feasibility of delivering patient-facing and provider-facing C-QIP intervention components within routine clinics. This approach was also informed by prior experience with individual-level randomization in multicomponent quality-improvement trials, including the CARRS Translation Trial, PLOS Medicine 2024 (<https://doi.org/10.1371/journal.pmed.1004335>). However, we agree that because C-QIP included provider-facing components, individual randomization within shared clinics creates a risk of provider-level spill-over. We have acknowledged this in limitations and recommend cluster randomization for a future hybrid effectiveness-implementation trial. **(page 17)**

*       The outcomes are more clearly detailed in the manuscript but I admit I have a concern re the multiple primary outcomes. I appreciate it is an implementation trial but I find the multiple (4x and from both patient and provider perspectives) primary outcomes a problem because impossible to know if the intervention as actually effective.

**Response:** We agree this needed clarification. We now describe the four implementation domains as co-primary feasibility outcomes, not multiple effectiveness endpoints, which were also aligned with Proctor’s recommended implementation outcomes. The purpose was not to determine whether the intervention was “effective” on a single primary clinical endpoint, but to assess whether the intervention could be delivered as intended and outcomes measured. **(Page 7).**

*        In outcomes section would also be good to have the timepoints of measurement.

**Response:** We agree and have now specified the timepoints of measurements considered for analysis: baseline and EOS. **(Page 8).**

*       Overall, I found the follow-up times unclear and confusing eg "post-randomization follow-up period ranged from 12 - 24 months, depending on participant enrolment in the trial." That is, when were patients follow-up? At 12 and 24-months or something different.

**Response:** Thank you for highlighting this point. We agree that the original wording could be interpreted as suggesting fixed 12- and 24-month outcome assessments. We have revised the Methods section to clarify that the main analytic timepoints for primary and secondary outcomes were baseline and the end-of-study (EOS) visit. Because recruitment and randomization occurred over a staggered period, participants had variable follow-up duration. To maximize follow-up within the trial close-out period, EOS visits were conducted between July and October 2024 and replaced the next scheduled/expected annual visit, whether that would otherwise have occurred at approximately 12 or 24 months after randomization. **(page 7).**

*       For the "Comparison with other trials" on p10. I recommend consideration of the QUEL cluster RCT that also has a parallel process evaluation (Process evaluation of a data-driven quality improvement program within a cluster randomised controlled trial to improve coronary heart disease management in Australian primary care | PLOS One). The preprint is here and this paper is in press with Circ Population and Outcomes Effectiveness of data-driven quality improvement on hospitalizations and health outcomes for people with coronary heart disease in primary care (QUEL): a cluster randomised controlled trial with 24-month follow-up | medRxiv and here is the protocol (QUality improvement in primary care to prevent hospitalisations and improve Effectiveness and efficiency of care for people Living with coronary heart disease (QUEL): protocol for a 24-month cluster randomised controlled trial in primary care | BMC Primary Care | Springer Nature Link). There is also the QUICR trial

that is ongoing with results still not published but here is the protocol (Data-driven collaborative QUality improvement in Cardiac Rehabilitation (QUICR) to increase program completion: protocol for a cluster randomized controlled trial | BMC Cardiovascular Disorders | Springer Nature Link). Preprint of results is available here (<https://apc01.safelinks.protection.outlook.com/?url=https%3A%2F%2Fwww.medrxiv.org%2Fcontent%2F10.1101%2F2025.06.12.25329537v1%3F__cf_chl_tk%3DDulwDDSRzteLPFcSGFsgEeo5G1dvJJTb50XCMpf6dRE-1775978684-1.0.1.1-AwHUvrgAneQ6vXILPTel8wdpUZixEN9o3EGLW4oT5hs&data=05%7C02%7Ckavita%40ccdcindia.org%7C15b95460c4ad417f2ef508dea20cf127%7Cbc95d6f5d5b847a7a8f6886cd31a6f5b%7C0%7C0%7C639126373956936683%7CUnknown%7CTWFpbGZsb3d8eyJFbXB0eU1hcGkiOnRydWUsIlYiOiIwLjAuMDAwMCIsIlAiOiJXaW4zMiIsIkFOIjoiTWFpbCIsIldUIjoyfQ%3D%3D%7C0%7C%7C%7C&sdata=1pdb4LADivedfQGqMzZU%2F2OnP3a%2B5KFJcb8UD71Fp0o%3D&reserved=0>) with main paper to be published in Circ: Cardiovascular Population Health and Outcomes in coming days (DOICIRCCQO-2025-012904-T). I feel these studies are highly relevant to the current paper.

**Response:** Thank you for your suggestions. We have added QUEL and QUICR to the discussion as relevant data-driven QI comparator trials, while noting differences in setting, cluster design and health-system context. **(page 15).**

*       There are a lot of abbreviations and I would recommend trying to significantly reduce eg EOS, C-QIP, CCB, ARNI, GDMT, DSS. I think this would improve readability.

**Response:** Thank you for your comment. We have expanded these acronyms on first use and reduced their use throughout the manuscript to improve readability.

Any attachments provided with reviews can be seen via the following link: <https://apc01.safelinks.protection.outlook.com/?url=https%3A%2F%2Fwww.editorialmanager.com%2Fpmedicine%2Fl.asp%3Fi%3D1114337%26l%3DOHKC5BGH&data=05%7C02%7Ckavita%40ccdcindia.org%7C15b95460c4ad417f2ef508dea20cf127%7Cbc95d6f5d5b847a7a8f6886cd31a6f5b%7C0%7C0%7C639126373956951337%7CUnknown%7CTWFpbGZsb3d8eyJFbXB0eU1hcGkiOnRydWUsIlYiOiIwLjAuMDAwMCIsIlAiOiJXaW4zMiIsIkFOIjoiTWFpbCIsIldUIjoyfQ%3D%3D%7C0%7C%7C%7C&sdata=DYlt21CmwANwTMecN%2Fk5F1cP4uHRsWVpP7Z%2FN%2BOyeAw%3D&reserved=0>

--------------------------------------------------------- ---

**General editorial requests:**

*       Please upload any figures associated with your paper as individual TIF or EPS files with 300dpi resolution at resubmission; please read our figure guidelines for more information on our requirements: <https://apc01.safelinks.protection.outlook.com/?url=http%3A%2F%2Fjournals.plos.org%2Fplosmedicine%2Fs%2Ffigures&data=05%7C02%7Ckavita%40ccdcindia.org%7C15b95460c4ad417f2ef508dea20cf127%7Cbc95d6f5d5b847a7a8f6886cd31a6f5b%7C0%7C0%7C639126373956965896%7CUnknown%7CTWFpbGZsb3d8eyJFbXB0eU1hcGkiOnRydWUsIlYiOiIwLjAuMDAwMCIsIlAiOiJXaW4zMiIsIkFOIjoiTWFpbCIsIldUIjoyfQ%3D%3D%7C0%7C%7C%7C&sdata=zFxGTfO0TabKJzTbUVLvOaU7PAPgOCsaMlowbj4l2mA%3D&reserved=0>. While revising your submission, we strongly recommend that you use PLOS's NAAS tool (<https://apc01.safelinks.protection.outlook.com/?url=https%3A%2F%2Fngplosjournals.pagemajik.ai%2Fartanalysis&data=05%7C02%7Ckavita%40ccdcindia.org%7C15b95460c4ad417f2ef508dea20cf127%7Cbc95d6f5d5b847a7a8f6886cd31a6f5b%7C0%7C0%7C639126373956980865%7CUnknown%7CTWFpbGZsb3d8eyJFbXB0eU1hcGkiOnRydWUsIlYiOiIwLjAuMDAwMCIsIlAiOiJXaW4zMiIsIkFOIjoiTWFpbCIsIldUIjoyfQ%3D%3D%7C0%7C%7C%7C&sdata=8%2Bjh3W8EFGEtOVSCVk%2BeOpTS%2BxAZvX%2BC7MFXAfQeS3I%3D&reserved=0>) to test your figure files. NAAS can convert your figure files to the TIFF file type and meet basic requirements (such as print size, resolution), or provide you with a report on issues that do not meet our requirements and that NAAS cannot fix.

**Response:** We have provided figures as .tiff file as per journal’s requirement

After uploading your figures to PLOS's NAAS tool - <https://apc01.safelinks.protection.outlook.com/?url=https%3A%2F%2Fngplosjournals.pagemajik.ai%2Fartanalysis&data=05%7C02%7Ckavita%40ccdcindia.org%7C15b95460c4ad417f2ef508dea20cf127%7Cbc95d6f5d5b847a7a8f6886cd31a6f5b%7C0%7C0%7C639126373956995589%7CUnknown%7CTWFpbGZsb3d8eyJFbXB0eU1hcGkiOnRydWUsIlYiOiIwLjAuMDAwMCIsIlAiOiJXaW4zMiIsIkFOIjoiTWFpbCIsIldUIjoyfQ%3D%3D%7C0%7C%7C%7C&sdata=8y9Q18XSbV2BSQltpVmxgWnvgIb03B4VzUWM1Jl9POI%3D&reserved=0>, NAAS will process the files provided and display the results in the "Uploaded Files" section of the page as the processing is complete.

If the uploaded figures meet our requirements (or NAAS is able to fix the files to meet our requirements), the figure will be marked as "fixed" above. If NAAS is unable to fix the files, a red "failed" label will appear above.

When NAAS has confirmed that the figure files meet our requirements, please download the file via the download option, and include these NAAS processed figure files when submitting your revised manuscript.

**Response:** We have uploaded NAAS processed figure files.

*       Please ensure that the paper adheres to the PLOS Data Availability Policy (see <https://apc01.safelinks.protection.outlook.com/?url=http%3A%2F%2Fjournals.plos.org%2Fplosmedicine%2Fs%2Fdata-availability&data=05%7C02%7Ckavita%40ccdcindia.org%7C15b95460c4ad417f2ef508dea20cf127%7Cbc95d6f5d5b847a7a8f6886cd31a6f5b%7C0%7C0%7C639126373957013393%7CUnknown%7CTWFpbGZsb3d8eyJFbXB0eU1hcGkiOnRydWUsIlYiOiIwLjAuMDAwMCIsIlAiOiJXaW4zMiIsIkFOIjoiTWFpbCIsIldUIjoyfQ%3D%3D%7C0%7C%7C%7C&sdata=4OL%2FZG1Uc512nvQN4meJQK19FQVLKhDNJXjcBuJzzbk%3D&reserved=0>), which requires that all data underlying the study's findings be provided in a repository or as Supporting Information. For data residing with a third party, authors are required to provide instructions with contact information (web or email address) for obtaining the data. Please note that a study author cannot be the contact person for the data. PLOS journals do not allow statements supported by "data not shown" or "unpublished results." For such statements, authors must provide supporting data or cite public sources that include it.

**Response:** We have included data availability statement in compliance with PLOS data availability policy.

* Data availability statement: Please note that your current data availability statement is not sufficient, and we do not allow an author of the study to be the point of contact. Please modify this statement, according to the guidelines above.

**Response:** We have added data availability statement and provided detail of an independent point of contact.

*       We expect all researchers with submissions to PLOS in which author-generated code underpins the findings in the manuscript to make all author-generated code available without restrictions upon publication of the work. In cases where code is central to the manuscript, we may require the code to be made available as a condition of publication. Authors are responsible for ensuring that the code is reusable and well documented. Please make any custom code available, either as part of your data deposition or as a supplementary file. Please add a sentence to your data availability statement regarding any code used in the study, e.g. "The code used in the analysis is available from Github [URL] and archived in Zenodo [DOI link]" Please review our guidelines at <https://apc01.safelinks.protection.outlook.com/?url=https%3A%2F%2Fjournals.plos.org%2Fplosmedicine%2Fs%2Fmaterials-software-and-code-sharing&data=05%7C02%7Ckavita%40ccdcindia.org%7C15b95460c4ad417f2ef508dea20cf127%7Cbc95d6f5d5b847a7a8f6886cd31a6f5b%7C0%7C0%7C639126373957034607%7CUnknown%7CTWFpbGZsb3d8eyJFbXB0eU1hcGkiOnRydWUsIlYiOiIwLjAuMDAwMCIsIlAiOiJXaW4zMiIsIkFOIjoiTWFpbCIsIldUIjoyfQ%3D%3D%7C0%7C%7C%7C&sdata=qlw2FCdUgI%2BAfK3%2FUzAxKIlf0rFHXHbsGWKotKBKSZo%3D&reserved=0>and ensure that your code is shared in a way that follows best practice and facilitates reproducibility and reuse. Because Github depositions can

be readily changed or deleted, we encourage you to make a permanent DOI'd copy (e.g. in Zenodo) and provide the URL.

**Response:** Thank you for your comment. De-identified participant-level data and statistical code may be made available to qualified researchers upon reasonable request, subject to ethics approvals and data-sharing agreements. Analysis code will be archived in a permanent repository.

* Financial disclosure: Please include the initials of the person who obtained the grant, as well as a URL to the funders' website.

**Response:** This study was supported by the Fogarty International Center, National Institutes of Health, United States, grant 1K43TW011164 awarded to KS.

* Ethics statement: Thank you for stating that signed consent forms were obtained. Please specify whether this was informed consent.

**Response:** Thank you for your comment**.** We confirm that “written informed consent was obtained.”

**FORMATTING - GENERAL**

*  Abstract: Please include the main limitations of your study at the end of the Methods and Findings section.

**Response:** We have added the main limitations at the end of the Methods and Findings section.

*       At this stage, we ask that you include a short, non-technical Author Summary of your research to make findings accessible to a wide audience that includes both scientists and non-scientists. The Author Summary should immediately follow the Abstract in your revised manuscript. This text is subject to editorial change and should be distinct from the scientific abstract. Ideally each sub-heading should contain 2-3 single sentence, concise bullet points containing the most salient points from your study. In the final bullet point of 'What Do These Findings Mean?', please include the main limitations of the study in non-technical language. Please see our author guidelines for more information: <https://apc01.safelinks.protection.outlook.com/?url=https%3A%2F%2Fjournals.plos.org%2Fplosmedicine%2Fs%2Frevising-your-manuscript%23loc-author-summary&data=05%7C02%7Ckavita%40ccdcindia.org%7C15b95460c4ad417f2ef508dea20cf127%7Cbc95d6f5d5b847a7a8f6886cd31a6f5b%7C0%7C0%7C639126373957059269%7CUnknown%7CTWFpbGZsb3d8eyJFbXB0eU1hcGkiOnRydWUsIlYiOiIwLjAuMDAwMCIsIlAiOiJXaW4zMiIsIkFOIjoiTWFpbCIsIldUIjoyfQ%3D%3D%7C0%7C%7C%7C&sdata=G0f7eA0UZIeDi7guQ7VP9L3e4AR44jhMt3MDz8Ok4vQ%3D&reserved=0>.

**Response:** We have added a short, non-technical author summary in 2-3 single sentence.

*       Please express the main results with 95% CIs as well as p values. When reporting p values please report as p<0.001 and where higher as the exact p value p=0.002, for example. Throughout, suggest reporting statistical information as follows to improve clarity for the reader "22% (95% CI [13%,28%]; p</=)". Please be sure to define all numerical values at first use.

**Response:** We report main results with 95% CIs as well as p values.

*       Please include page numbers and line numbers in the manuscript file. Use continuous line numbers (do not restart the numbering on each page).

**Response:** We have added page numbers and line numbers using continuous line numbers.

*       Please cite the reference numbers in square brackets. Citations should precede punctuation.

**Response:** Reference numbers are in square brackets.

FIGURES AND TABLES

*       Please provide titles and legends for all figures and tables (including those in Supporting Information files).

**Response:** All figures have titles and legends.

*       Please define all abbreviations used in each figure/table (including those in Supporting Information files).

**Response:** Done.

**SUPPLEMENTARY MATERIAL**

*       Please note that supplementary material will be posted as supplied by the authors. Therefore, please amend it according to the relevant comments outlined here.

*       Please cite your Supporting Information as outlined here: <https://apc01.safelinks.protection.outlook.com/?url=https%3A%2F%2Fjournals.plos.org%2Fplosmedicine%2Fs%2Fsupporting-information&data=05%7C02%7Ckavita%40ccdcindia.org%7C15b95460c4ad417f2ef508dea20cf127%7Cbc95d6f5d5b847a7a8f6886cd31a6f5b%7C0%7C0%7C639126373957080714%7CUnknown%7CTWFpbGZsb3d8eyJFbXB0eU1hcGkiOnRydWUsIlYiOiIwLjAuMDAwMCIsIlAiOiJXaW4zMiIsIkFOIjoiTWFpbCIsIldUIjoyfQ%3D%3D%7C0%7C%7C%7C&sdata=D9FKx7bj5kWblGKVrUeC6nFLci1vNmvmkO1alBpfQTM%3D&reserved=0>

**Response:** Thank you for your comment.

REFERENCES

*       PLOS uses the numbered citation (citation-sequence) method and first six authors, et al.

**Response:** Done

*       Please ensure that journal name abbreviations match those found in the National Center for Biotechnology Information (NCBI) databases (<https://apc01.safelinks.protection.outlook.com/?url=http%3A%2F%2Fwww.ncbi.nlm.nih.gov%2Fnlmcatalog%2Fjournals&data=05%7C02%7Ckavita%40ccdcindia.org%7C15b95460c4ad417f2ef508dea20cf127%7Cbc95d6f5d5b847a7a8f6886cd31a6f5b%7C0%7C0%7C639126373957100491%7CUnknown%7CTWFpbGZsb3d8eyJFbXB0eU1hcGkiOnRydWUsIlYiOiIwLjAuMDAwMCIsIlAiOiJXaW4zMiIsIkFOIjoiTWFpbCIsIldUIjoyfQ%3D%3D%7C0%7C%7C%7C&sdata=nlDVOXb5BO%2Fpys%2FVvnHsqT1tYr50fyIwIuLHMNiv5oM%3D&reserved=0>), and are appropriately formatted and capitalised.

**Response:** Done.

*       Where website addresses are cited, please include the complete URL and specify the date of access (e.g. [accessed: 12/06/2023]).

**Response:** Done.

*       Please also see <https://apc01.safelinks.protection.outlook.com/?url=https%3A%2F%2Fjournals.plos.org%2Fplosmedicine%2Fs%2Fsubmission-guidelines%23loc-references&data=05%7C02%7Ckavita%40ccdcindia.org%7C15b95460c4ad417f2ef508dea20cf127%7Cbc95d6f5d5b847a7a8f6886cd31a6f5b%7C0%7C0%7C639126373957119263%7CUnknown%7CTWFpbGZsb3d8eyJFbXB0eU1hcGkiOnRydWUsIlYiOiIwLjAuMDAwMCIsIlAiOiJXaW4zMiIsIkFOIjoiTWFpbCIsIldUIjoyfQ%3D%3D%7C0%7C%7C%7C&sdata=qKuuFza2tXNX8em5QbTWoxaeH8GkiYdazV8%2Bnvtljr8%3D&reserved=0>for further details on reference formatting.

**Response:** Done.

RCTs

*       Please structure the Methods section using the following sub-headings: Study design and participants, Randomization and masking, Procedures, Outcomes, Statistical analysis.

**Response:** Done.

*       Please ensure that all prespecified outcomes (primary, secondary, and exploratory) are listed in the Methods/Outcomes section and indicate whether there are outcomes that are not presented in the current report.

**Response:** Done.

*       Please specify the dates (Month Day, Year) during which study enrollment and follow up occurred.

**Response:** Done.

*       Please include absolute numbers wherever you report percentages; eg, n/N (%)

**Response:** Done.

*       Please present the safety data for the study including numbers of specific events and whether or not adverse events are thought to be related to treatment. AEs should be reported in the abstract, per CONSORT and CONSORT-Harms.

**Response:** Done.

*       Thank you for including the CONSORT checklist. We ask you to use use section and paragraph numbers, rather than page numbers. Please modify

**Response:** Done.

*       Please report your abstract according to CONSORT for abstracts, following the PLOS Medicine abstract structure (Background, Methods and Findings, Conclusions) <https://apc01.safelinks.protection.outlook.com/?url=https%3A%2F%2Fwww.equator-network.org%2Freporting-guidelines%2Fconsort-abstracts%2F&data=05%7C02%7Ckavita%40ccdcindia.org%7C15b95460c4ad417f2ef508dea20cf127%7Cbc95d6f5d5b847a7a8f6886cd31a6f5b%7C0%7C0%7C639126373957134816%7CUnknown%7CTWFpbGZsb3d8eyJFbXB0eU1hcGkiOnRydWUsIlYiOiIwLjAuMDAwMCIsIlAiOiJXaW4zMiIsIkFOIjoiTWFpbCIsIldUIjoyfQ%3D%3D%7C0%7C%7C%7C&sdata=%2BEqdxar2kMKkOp%2FzIxsyJC4ilzDxZQa9Q3C1nVwPFWA%3D&reserved=0>

**Response:** Done.

**EDITORIAL Formatting Comments**

Ref: A Randomized Feasibility Trial of a Multicomponent Quality Improvement Strategy for Chronic Care of Cardiovascular Diseases: Findings from the C-QIP Trial in India

PMEDICINE-D-26-00187R1

Dear Dr Singh,

Thank you for your patience while we performed technical checks on your submission. Please see below for a list of requirements for you to address while conducting your revisions.

Journal Requirements:

1) Please provide amend your detailed Financial Disclosure statement. This is published with the article. It must therefore be completed in full sentences and contain the exact wording you wish to be published.

**Response:** Thank you for your comment. We have added detailed financial disclosure statement.

i) State the initials, alongside each funding source, of each author to receive each grant.

**Response:** Done.

ii)State what role the funders took in the study. If the funders had no role in your study, please state: “The funders had no role in study design, data collection and analysis, decision to publish, or preparation of the manuscript.”

**Response:** Done.

iii) If any authors received a salary from any of your funders, please state which authors and which funders.

**Response:** Done

2) Please provide separate figure files (Figures 1 - 4) in .tif or .eps format. For more information about figure files please see our guidelines: <https://apc01.safelinks.protection.outlook.com/?url=https%3A%2F%2Fjournals.plos.org%2Fplosmedicine%2Fs%2Ffigures&data=05%7C02%7Ckavita%40ccdcindia.org%7C2771bf406c2b4b11eb8608dea4297be0%7Cbc95d6f5d5b847a7a8f6886cd31a6f5b%7C0%7C0%7C639128695282888590%7CUnknown%7CTWFpbGZsb3d8eyJFbXB0eU1hcGkiOnRydWUsIlYiOiIwLjAuMDAwMCIsIlAiOiJXaW4zMiIsIkFOIjoiTWFpbCIsIldUIjoyfQ%3D%3D%7C0%7C%7C%7C&sdata=T6oS130WWtghFB5ZiCpR%2FnAQRvdSpMNcWjCSjMgXhJ0%3D&reserved=0>, <https://apc01.safelinks.protection.outlook.com/?url=https%3A%2F%2Fjournals.plos.org%2Fplosmedicine%2Fs%2Ffigures%23loc-file-requirements&data=05%7C02%7Ckavita%40ccdcindia.org%7C2771bf406c2b4b11eb8608dea4297be0%7Cbc95d6f5d5b847a7a8f6886cd31a6f5b%7C0%7C0%7C639128695282947772%7CUnknown%7CTWFpbGZsb3d8eyJFbXB0eU1hcGkiOnRydWUsIlYiOiIwLjAuMDAwMCIsIlAiOiJXaW4zMiIsIkFOIjoiTWFpbCIsIldUIjoyfQ%3D%3D%7C0%7C%7C%7C&sdata=CVn8vYdYSBFoUie5JP1V1u0HHGumkzmKLgv3FGvUmms%3D&reserved=0>

**Response:** Done.

3)We do not publish any copyright or trademark symbols that usually accompany proprietary names, eg (R), (C), or TM  (e.g. next to drug or reagent names). Please remove all instances of trademark/copyright symbols throughout the text, including [PROMIS®] in file "C-QIP Trial Results on" on page [7].

**Response:** Done.

4) Tables should not be uploaded as individual files. Please remove these files and include the Tables in your manuscript file as editable, cell-based objects. For more information about how to format tables, see our guidelines: <https://apc01.safelinks.protection.outlook.com/?url=https%3A%2F%2Fjournals.plos.org%2Fplosmedicine%2Fs%2Ftables&data=05%7C02%7Ckavita%40ccdcindia.org%7C2771bf406c2b4b11eb8608dea4297be0%7Cbc95d6f5d5b847a7a8f6886cd31a6f5b%7C0%7C0%7C639128695283030230%7CUnknown%7CTWFpbGZsb3d8eyJFbXB0eU1hcGkiOnRydWUsIlYiOiIwLjAuMDAwMCIsIlAiOiJXaW4zMiIsIkFOIjoiTWFpbCIsIldUIjoyfQ%3D%3D%7C0%7C%7C%7C&sdata=yizgBjpP1ll9s5qyCivrejkI9MzqVpfvz2aPfTEoLeM%3D&reserved=0>

**Response:** Done.

5)We have noticed that you have uploaded Supporting Information files (Online supplementary file_18.01.2026.pdf. C-QIP Protocol 07 Feb 2022.pdf,        C-QIP_SAP_Final version_30.10.2024.pdf, CONSORT 2025_C-QIP.pdf), but you have not included a list of legends. Please add a full list of legends for your Supporting Information files after the references list.

**Response:** Done.

6)We notice that your supplementary figures (S1 -S3 Figures and S1 - S6 Tables) are uploaded with the file type 'Figure'. Please amend the file type to 'Supporting Information'. Please ensure that each Supporting Information file has a legend listed in the manuscript after the references list.

**Response:** Done.

7) Please note that the contact for accessing your data cannot be an author on the manuscript. Please therefore replace the current contact information with a third party contact, such as an institutional data access committee.

**Response:** Done.
