# Supplementary material for: Assessing a multicomponent quality improvement strategy for chronic care of cardiovascular diseases in India: The C-QIP randomized feasibility trial
Source: PLoS Med. 2026 Sep 29;23(9):e1004932. doi: 10.1371/journal.pmed.1004932 (PMC13641716; doi:10.1371/journal.pmed.1004932)
Supplement: Supplementary file 10 [file pmed.1004932.s012.docx]

**Review Comments**

**Comments from the academic editor:**

To be able to respond to the reviewers the authors have had to conduct many new analyses (including sensitivity analyses etc.). These added to the initial analyses can amount to 'multiple comparisons' and risk of chance findings. While the authors do not need to adjust for multiple comparisons; it could make sense to ask them to comment on the possibility of chance findings from multiple comparison, and whether this shouldn't perhaps be included in the limitations.

***Response****: We thank the Academic Editor for this important observation. We agree that the number of outcomes and additional exploratory, subgroup, and sensitivity analyses increases the possibility that some statistically significant findings may have occurred by chance.*

*We have not applied a formal adjustment for multiple comparisons because this was a feasibility trial designed primarily to assess implementation outcomes and estimate preliminary effect sizes rather than to conduct confirmatory hypothesis testing. In addition, the sensitivity analyses were conducted to assess the robustness of the primary findings rather than to identify additional independent treatment effects. Nevertheless, we agree that the potential for chance findings should be made explicit.*

*We have therefore revised the Strengths and limitations section to acknowledge that, given the number of secondary, exploratory, and subgroup comparisons, our study findings should be interpreted cautiously and regarded as hypothesis-generating pending confirmation in a larger, adequately powered trial. (refer to page no. 17)*

-----------------------------------------------------------

Comments from the reviewers:

**Reviewer #1:** The authors have addressed the major methodological and statistical concerns raised in the previous review. The manuscript has been substantially improved, particularly with respect to the feasibility-trial framing, clarification of the statistical analysis plan deviations, prespecified covariate adjustment, handling of missing data, subgroup analyses, and interpretation of secondary outcomes. I have only two remaining statistical comments:

***Response****: We thank the reviewer for their careful review and for the positive assessment of the revised manuscript. We have addressed the two remaining statistical comments as follows:*

a) The addition of IPTW sensitivity analyses is appreciated. Please briefly summarize in the manuscript whether the conclusions from the IPTW analyses differed materially from the primary analyses and comment on the implications for the robustness of the findings to missing-data assumptions.

***Response****: Thank you for this suggestion. We have now added a brief statement in the Results section indicating that the IPTW analyses, which accounted for the probability of end-of-study visit completion, produced estimates that were directionally consistent with the primary complete-case analyses. The magnitude of the estimated effects for the key process-of-care outcomes, including GDMT prescription and self-reported adherence outcomes, did not differ substantially from the primary analyses. These findings support the robustness of the main conclusions. (refer to page no. 13)*

b) Model diagnostics: The manuscript indicates that model assumptions for continuous outcomes were assessed. Please provide a brief description of the diagnostic procedures performed (e.g., assessment of residual distributions and model fit) and indicate whether any important violations were identified.

***Response****: We thank the reviewer for this helpful suggestion. We have revised the Statistical analysis section to provide additional detail on the diagnostic procedures used for continuous outcomes. Specifically, we assessed residual distributions using residual-versus-fitted plots, histograms, and normal quantile plots, and examined whether influential observations or marked deviations from model assumptions were present. (refer to page no. 9)*

--------------------------------------------------------- ---

General editorial requests:

**GENERAL**


* Please confirm that your title complies with PLOS Medicine's style. Your title must be nondeclarative and not a question. It should begin with main concept if possible. "Effect of" should be used only if causality can be inferred, i.e., for an RCT. Please place the study design ("A randomized controlled trial," "A retrospective study," "A modelling study," etc.) in the subtitle (ie, after a colon). Please modify your title accordingly.

**Response:** Yes, we confirm the manuscript title complies with PLOS Medicine’s journal guidelines.


* Please ensure that all abbreviations are defined at first use throughout the text.
**Response:** Yes, we confirm all abbreviations are defined on first use throughout the manuscript.


* Please confirm that all numbers presented in the abstract are present and identical to numbers presented in the main manuscript text.

**Response:** Yes, we have cross-checked all numbers presented in the abstract and these are consistent with reported numbers in the main manuscript text and Tables/Figures.

*       Please ensure that the paper adheres to the PLOS Data Availability Policy (see <https://apc01.safelinks.protection.outlook.com/?url=http%3A%2F%2Fjournals.plos.org%2Fplosmedicine%2Fs%2Fdata-availability&data=05%7C02%7Ckavita%40ccdcindia.org%7C15b95460c4ad417f2ef508dea20cf127%7Cbc95d6f5d5b847a7a8f6886cd31a6f5b%7C0%7C0%7C639126373957013393%7CUnknown%7CTWFpbGZsb3d8eyJFbXB0eU1hcGkiOnRydWUsIlYiOiIwLjAuMDAwMCIsIlAiOiJXaW4zMiIsIkFOIjoiTWFpbCIsIldUIjoyfQ%3D%3D%7C0%7C%7C%7C&sdata=4OL%2FZG1Uc512nvQN4meJQK19FQVLKhDNJXjcBuJzzbk%3D&reserved=0>), which requires that all data underlying the study's findings be provided in a repository or as Supporting Information. For data residing with a third party, authors are required to provide instructions with contact information (web or email address) for obtaining the data. Please note that a study author cannot be the contact person for the data. PLOS journals do not allow statements supported by "data not shown" or "unpublished results." For such statements, authors must provide supporting data or cite public sources that include it.

**Response:** We have included data availability statement in compliance with PLOS data availability policy.

* Please provide the data, or provide reasoning why this is legally not allowed.

**Response:** We have added data availability statement and provided detail of an independent point of contact to facilitate data sharing. As per Indian Council of Medical Research (ICMR) guidelines, we cannot share study data directly, without ensuring data sharing agreements to comply with our Indian data protection rights.

*       We expect all researchers with submissions to PLOS in which author-generated code underpins the findings in the manuscript to make all author-generated code available without restrictions upon publication of the work. In cases where code is central to the manuscript, we may require the code to be made available as a condition of publication. Authors are responsible for ensuring that the code is reusable and well documented. Please make any custom code available, either as part of your data deposition or as a supplementary file. Please add a sentence to your data availability statement regarding any code used in the study, e.g. "The code used in the analysis is available from Github [URL] and archived in Zenodo [DOI link]" Please review our guidelines at <https://apc01.safelinks.protection.outlook.com/?url=https%3A%2F%2Fjournals.plos.org%2Fplosmedicine%2Fs%2Fmaterials-software-and-code-sharing&data=05%7C02%7Ckavita%40ccdcindia.org%7C15b95460c4ad417f2ef508dea20cf127%7Cbc95d6f5d5b847a7a8f6886cd31a6f5b%7C0%7C0%7C639126373957034607%7CUnknown%7CTWFpbGZsb3d8eyJFbXB0eU1hcGkiOnRydWUsIlYiOiIwLjAuMDAwMCIsIlAiOiJXaW4zMiIsIkFOIjoiTWFpbCIsIldUIjoyfQ%3D%3D%7C0%7C%7C%7C&sdata=qlw2FCdUgI%2BAfK3%2FUzAxKIlf0rFHXHbsGWKotKBKSZo%3D&reserved=0>and ensure that your code is shared in a way that follows best practice and facilitates reproducibility and reuse. Because Github depositions can

be readily changed or deleted, we encourage you to make a permanent DOI'd copy (e.g. in Zenodo) and provide the URL.

**Response:** Thank you for your comment. De-identified participant-level data and statistical code may be made available to qualified researchers upon reasonable request, subject to ethics approvals and data-sharing agreements. Analysis code will be archived in a permanent repository.

* Please provide a statement on where the code can be found in your data availability statement.
**Response:** see page no. 18


* Please disclose any use of AI tools and technologies to the study or article content at the end of the Methods. Please specify the name(s) and version(s) of any tool(s) used and a description of their use, including how the AI-assisted or generated content was evaluated by authors, and a clear statement of which aspects of the study, article contents, data, or supporting files were affected/generated by AI tool usage. Please also include a statement specifying who retains responsibility for the final manuscript. See our guidelines for more information: [https://apc01.safelinks.protection.outlook.com/?url=https%3A%2F%2Fjournals.plos.org%2Fplosmedicine%2Fs%2Fethical-publishing-practice%23loc-artificial-intelligence-tools-and-technologies&data=05%7C02%7Cmeetushi%40ccdcindia.org%7C9b902b2949cb4403130608dedb4ccc5e%7Cbc95d6f5d5b847a7a8f6886cd31a6f5b%7C0%7C0%7C639189320055829554%7CUnknown%7CTWFpbGZsb3d8eyJFbXB0eU1hcGkiOnRydWUsIlYiOiIwLjAuMDAwMCIsIlAiOiJXaW4zMiIsIkFOIjoiTWFpbCIsIldUIjoyfQ%3D%3D%7C0%7C%7C%7C&sdata=%2Bt6zgxyVg9P7Br2SS4uUFuL42GGGOHax9YhQCOco9C4%3D&reserved=0](https://apc01.safelinks.protection.outlook.com/?url=https%3A%2F%2Fjournals.plos.org%2Fplosmedicine%2Fs%2Fethical-publishing-practice%23loc-artificial-intelligence-tools-and-technologies&data=05%7C02%7Ckavita%40ccdcindia.org%7C04a6ea66b93e42bd044108dedf1bba69%7Cbc95d6f5d5b847a7a8f6886cd31a6f5b%7C0%7C0%7C639193507672929952%7CUnknown%7CTWFpbGZsb3d8eyJFbXB0eU1hcGkiOnRydWUsIlYiOiIwLjAuMDAwMCIsIlAiOiJXaW4zMiIsIkFOIjoiTWFpbCIsIldUIjoyfQ%3D%3D%7C0%7C%7C%7C&sdata=GKTLCYGhmtrrqiSsFCc3SkpnWmHlw3hnbtNSrSTBNBY%3D&reserved=0)<[https://apc01.safelinks.protection.outlook.com/?url=https%3A%2F%2Fjournals.plos.org%2Fplosmedicine%2Fs%2Fethical-publishing-practice%23loc-artificial-intelligence-tools-and-technologies&data=05%7C02%7Cmeetushi%40ccdcindia.org%7C9b902b2949cb4403130608dedb4ccc5e%7Cbc95d6f5d5b847a7a8f6886cd31a6f5b%7C0%7C0%7C639189320055853739%7CUnknown%7CTWFpbGZsb3d8eyJFbXB0eU1hcGkiOnRydWUsIlYiOiIwLjAuMDAwMCIsIlAiOiJXaW4zMiIsIkFOIjoiTWFpbCIsIldUIjoyfQ%3D%3D%7C0%7C%7C%7C&sdata=FKOJHCo8pqeeBxq%2Bipk1GYnpdIzolSIgHHBoj93vy%2Fs%3D&reserved=0](https://apc01.safelinks.protection.outlook.com/?url=https%3A%2F%2Fjournals.plos.org%2Fplosmedicine%2Fs%2Fethical-publishing-practice%23loc-artificial-intelligence-tools-and-technologies&data=05%7C02%7Ckavita%40ccdcindia.org%7C04a6ea66b93e42bd044108dedf1bba69%7Cbc95d6f5d5b847a7a8f6886cd31a6f5b%7C0%7C0%7C639193507672943330%7CUnknown%7CTWFpbGZsb3d8eyJFbXB0eU1hcGkiOnRydWUsIlYiOiIwLjAuMDAwMCIsIlAiOiJXaW4zMiIsIkFOIjoiTWFpbCIsIldUIjoyfQ%3D%3D%7C0%7C%7C%7C&sdata=Z9O1Nrp47r%2FSqQaObXcpj3RKLcOiBEYz84AR018ipwQ%3D&reserved=0)> <[https://apc01.safelinks.protection.outlook.com/?url=https%3A%2F%2Fjournals.plos.org%2Fplosmedicine%2Fs%2Fethical-publishing-practice%23loc-artificial-intelligence-tools-and-technologies&data=05%7C02%7Cmeetushi%40ccdcindia.org%7C9b902b2949cb4403130608dedb4ccc5e%7Cbc95d6f5d5b847a7a8f6886cd31a6f5b%7C0%7C0%7C639189320055897747%7CUnknown%7CTWFpbGZsb3d8eyJFbXB0eU1hcGkiOnRydWUsIlYiOiIwLjAuMDAwMCIsIlAiOiJXaW4zMiIsIkFOIjoiTWFpbCIsIldUIjoyfQ%3D%3D%7C0%7C%7C%7C&sdata=WH%2BlZ7VQt%2F%2F9FmXD%2Fn3RuC%2B5bM9unDwqFg1OYcBXRr4%3D&reserved=0](https://apc01.safelinks.protection.outlook.com/?url=https%3A%2F%2Fjournals.plos.org%2Fplosmedicine%2Fs%2Fethical-publishing-practice%23loc-artificial-intelligence-tools-and-technologies&data=05%7C02%7Ckavita%40ccdcindia.org%7C04a6ea66b93e42bd044108dedf1bba69%7Cbc95d6f5d5b847a7a8f6886cd31a6f5b%7C0%7C0%7C639193507672957061%7CUnknown%7CTWFpbGZsb3d8eyJFbXB0eU1hcGkiOnRydWUsIlYiOiIwLjAuMDAwMCIsIlAiOiJXaW4zMiIsIkFOIjoiTWFpbCIsIldUIjoyfQ%3D%3D%7C0%7C%7C%7C&sdata=xRcOLrYQdLU0SQWOjdCjmrSrpNqbLx6wZEI7ALNuqyY%3D&reserved=0)> <[https://apc01.safelinks.protection.outlook.com/?url=https%3A%2F%2Fjournals.plos.org%2Fplosmedicine%2Fs%2Fethical-publishing-practice%23loc-artificial-intelligence-tools-and-technologies&data=05%7C02%7Cmeetushi%40ccdcindia.org%7C9b902b2949cb4403130608dedb4ccc5e%7Cbc95d6f5d5b847a7a8f6886cd31a6f5b%7C0%7C0%7C639189320055918179%7CUnknown%7CTWFpbGZsb3d8eyJFbXB0eU1hcGkiOnRydWUsIlYiOiIwLjAuMDAwMCIsIlAiOiJXaW4zMiIsIkFOIjoiTWFpbCIsIldUIjoyfQ%3D%3D%7C0%7C%7C%7C&sdata=lI4ILPLZFzruB3VF5w9mLaH1KVnBOsPR6LqBWAk%2FFUE%3D&reserved=0;](https://apc01.safelinks.protection.outlook.com/?url=https%3A%2F%2Fjournals.plos.org%2Fplosmedicine%2Fs%2Fethical-publishing-practice%23loc-artificial-intelligence-tools-and-technologies&data=05%7C02%7Ckavita%40ccdcindia.org%7C04a6ea66b93e42bd044108dedf1bba69%7Cbc95d6f5d5b847a7a8f6886cd31a6f5b%7C0%7C0%7C639193507672970481%7CUnknown%7CTWFpbGZsb3d8eyJFbXB0eU1hcGkiOnRydWUsIlYiOiIwLjAuMDAwMCIsIlAiOiJXaW4zMiIsIkFOIjoiTWFpbCIsIldUIjoyfQ%3D%3D%7C0%7C%7C%7C&sdata=FkideQm%2FyHqCrifpmeWzADtzSM7%2BYkML7Dk6ejr1LaM%3D&reserved=0)>.
**Response:** We did not use any AI tools for this study reporting.

* In the author summary, in the final bullet point of 'What Do These Findings Mean?', please include the main limitations of the study in non-technical language.
**Response:** Done.

* Statistical reporting: Please revise throughout the manuscript, including tables and figures.
- Please report statistical information as follows to improve clarity for the reader ""22% (95% CI [13,28]; p</=)"".
- Please separate upper and lower bounds with commas instead of hyphens as the latter can be confused with reporting of negative values.
- Please repeat statistical definitions (HR, CI etc.) for each set of parentheses.

**Response:** Done.


* Please remove the 'conclusions' subheading from the discussion. Please also remove any other subheadings from the discussion.
**Response:** Done.


FUNDING STATEMENT
* The funding statement should include: specific grant numbers, initials of authors who received each award, URLs to sponsors’ websites. Also, please state whether any sponsors or funders (other than the named authors) played any role in study design, data collection and analysis, the decision to publish, or preparation of the manuscript. If they had no role in the research, include this sentence: “The funders had no role in study design, data collection and analysis, decision to publish, or preparation of the manuscript.”

**Response:** Done.


* Please clarify the funder of grant “K24HL175228 to MDH” and provide a URL to the funders website.

**Response:** Done.

DATA AVAILABILITY

* PLOS Medicine requires that the de-identified data underlying the specific results in a published article be made available, without restrictions on access, in a public repository or as Supporting Information at the time of article publication, provided it is legal and ethical to do so. Please see the policy at <https://apc01.safelinks.protection.outlook.com/?url=http%3A%2F%2Fjournals.plos.org%2Fplosmedicine%2Fs%2Fdata-availability&data=05%7C02%7Ckavita%40ccdcindia.org%7Ca31dad9e572e41c64c2a08ded91d0d6b%7Cbc95d6f5d5b847a7a8f6886cd31a6f5b%7C0%7C0%7C639186915975885338%7CUnknown%7CTWFpbGZsb3d8eyJFbXB0eU1hcGkiOnRydWUsIlYiOiIwLjAuMDAwMCIsIlAiOiJXaW4zMiIsIkFOIjoiTWFpbCIsIldUIjoyfQ%3D%3D%7C0%7C%7C%7C&sdata=6Njkue9fPpBCurb9aqqj76I5v9%2BflvnxYUFjDnt5a7s%3D&reserved=0>and FAQs at

<https://apc01.safelinks.protection.outlook.com/?url=http%3A%2F%2Fjournals.plos.org%2Fplosmedicine%2Fs%2Fdata-availability%23loc-faqs-for-data-policy&data=05%7C02%7Ckavita%40ccdcindia.org%7Ca31dad9e572e41c64c2a08ded91d0d6b%7Cbc95d6f5d5b847a7a8f6886cd31a6f5b%7C0%7C0%7C639186915975897383%7CUnknown%7CTWFpbGZsb3d8eyJFbXB0eU1hcGkiOnRydWUsIlYiOiIwLjAuMDAwMCIsIlAiOiJXaW4zMiIsIkFOIjoiTWFpbCIsIldUIjoyfQ%3D%3D%7C0%7C%7C%7C&sdata=ZBxPWYu1YUx7fUfhPHAeVQA8atikqsJlJ0UP%2F%2Fp2%2F48%3D&reserved=0>

**Response:** Thank you for your comment. De-identified participant-level data and statistical code may be made available to qualified researchers upon reasonable request, subject to ethics approvals and data-sharing agreements. Analysis code will be archived in a permanent repository.

* Please include the statement on code availability in the data availability statement. Please see guidance here:<https://apc01.safelinks.protection.outlook.com/?url=https%3A%2F%2Fjournals.plos.org%2Fplosmedicine%2Fs%2Fcode-sharing-guidance%23loc-data-availability-statement-examples&data=05%7C02%7Ckavita%40ccdcindia.org%7Ca31dad9e572e41c64c2a08ded91d0d6b%7Cbc95d6f5d5b847a7a8f6886cd31a6f5b%7C0%7C0%7C639186915975908931%7CUnknown%7CTWFpbGZsb3d8eyJFbXB0eU1hcGkiOnRydWUsIlYiOiIwLjAuMDAwMCIsIlAiOiJXaW4zMiIsIkFOIjoiTWFpbCIsIldUIjoyfQ%3D%3D%7C0%7C%7C%7C&sdata=Oi4r9ZV1k4oH2JhUceserRtuMIwpx0NOf4exlH%2B9NAc%3D&reserved=0>.

**Response:** see page no. 18.

TABLES AND FIGURES
* When a p value is given, please specify the statistical test used to determine it in the legend.

**Response:** Done.


CLINICAL TRIALS
* Please complete the CONSORT 2025 checklist and ensure that all components of CONSORT 2025 are present in the manuscript, including how randomization was performed, allocation concealment, blinding of intervention, definition of lost to follow-up, power statement. When completing the checklist, please use section and paragraph numbers, rather than page numbers. The checklist should be included as supporting information, and should be cited in the article.
**Response:** Done.


* Thank you for including the CONSORT checklist. We ask you to use use section and paragraph numbers, rather than page numbers. Please modify. I appreciate we asked this previously, but the CONSORT checklist that is currently uploaded still uses page numbers.
**Response:** Done.


* Please add the following statement, or similar, to the Methods: "This study is reported as per the Consolidated Standards of Reporting Trials (CONSORT) guidelines (S1 Checklist)."

**Response:** Done.

**Technical requests from the Journal**

**Journal Requirements:**

1) Please ensure a full list of SI Legends are included in the manuscript file.

**Response:** Done.

2) Some material included in your submission may be copyrighted. According to PLOS’s copyright policy, authors who use figures or other material (e.g., graphics, clipart, maps) from another author or copyright holder must demonstrate or obtain permission to publish this material under the Creative Commons Attribution 4.0 International (CC BY 4.0) License used by PLOS journals. Please closely review the details of PLOS’s copyright requirements here: PLOS Licenses and Copyright. If you need to request permissions from a copyright holder, you may use PLOS's Copyright Content Permission form.

Please respond directly to this email and provide any known details concerning your material's license terms and permissions required for reuse, even if you have not yet obtained copyright permissions or are unsure of your material's copyright compatibility. Once you have responded and addressed all other outstanding technical requirements, you may resubmit your manuscript within Editorial Manager.

Potential Copyright Issues:


i) [Supplementary file 2]: Please confirm (a) that you are the photographer; or (b) provide written permission from the photographer to publish the photo(s) under our CC BY 4.0 license.

**Response:** Thank you for your comment. We confirm that the photographs of intervention materials included in Supplementary File 2 was taken by one of the co-authors of this manuscript. The co-author has provided permission for the photographs to be published in accordance with the journal's requirements.

ii) [Supplementary file 2]: Please confirm whether you drew the images / clip-art within the figure panels by hand. If you did not draw the images, please provide (a) a link to the source of the images or icons and their license / terms of use; or (b) written permission from the copyright holder to publish the images or icons under our CC BY 4.0 license. Alternatively, you may replace the images with open source alternatives. See these open source resources you may use to replace images / clip-art:

**Response:** Thank you for your comment. We have removed the first page of Audit and Feedback report containing clip arts and logos of the study and trial.

iii] [Supplementary file 2] includes an image of an identifiable person. Please provide written confirmation or release forms, signed by the subject(s) (or their parent/legally authorized guardian), giving permission to be photographed and to have their images published under our CC BY 4.0 license. Otherwise, we kindly request that you remove the photograph.

**Response:** Thank you for your comment. We have removed the screenshot of login page of Electronic Health Records developed for the C-QIP Trial containing an identifiable individual photograph.

iv) [ C-QIP Protocol,Supplementary file 2 ] contains branding/a logo. We are not permitted to publish this under our CC BY 4.0 license, even with permission. We ask that you please remove or replace it.

**Response:** Thank you for your comment. We have removed the first page of Audit and Feedback report containing logos of the study trial and study collaborating sites.

v) [Supplementary file 2] contains screenshots. We are not permitted to publish these under our CC BY 4.0 license; websites are usually intellectual property and are copyrighted. This includes peripheral graphics of the web browser such as the [X] buttons. We ask that you please remove or replace it

**Response:** Thank you for your comment. We have removed all screenshots of Electronic Health Records developed for the C-QIP Trial.
