# Supplementary material for: Assessing a multicomponent quality improvement strategy for chronic care of cardiovascular diseases in India: The C-QIP randomized feasibility trial
Source: PLoS Med. 2026 Sep 29;23(9):e1004932. doi: 10.1371/journal.pmed.1004932 (PMC13641716; doi:10.1371/journal.pmed.1004932)
Supplement: Supplementary file 11 [file pmed.1004932.s013.docx]

Requests from Editors:

TITLE:

* Please change your title to: "Assessing a Multicomponent Quality Improvement Strategy for Chronic Care of Cardiovascular Diseases in India: the c-QIP randomised feasibility trial".

**Response:** Thank you for this suggestion. We have updated the manuscript title to reflect this change. Refer to page 1.

DATA AVAILABILITY STATEMENT

*Thank you for providing contact details that are not the study author. However, we need you to include more detail into this statement. This include the reason why the data cannot be made available, and the requirements for researchers to gain access to the data (see point C below). We also need you to include a statement on where the code can be found in the Data Availability Statement, in the metadata of the manuscript.

* The Data Availability Statement (DAS) requires revision. For each data source used in your study:

a) If the data are freely or publicly available, note this and state the location of the data: within the paper, in Supporting Information files, or in a public repository (include the DOI or accession number).

b) If the data are owned by a third party but freely available upon request, please note this and state the institutional owner of the data set and contact information for data requests (institutional web or email address). Note that a study author cannot be the contact for the data.

c) If the data are not freely available, please describe briefly the ethical, legal, or contractual restriction that prevents you from sharing it. Please also include an appropriate institutional contact (institutional web or email address) for inquiries (again, this cannot be a study author).

-Code: Thank you for stating that the code will be archived in a permanent repository. Please provide the DOI in the Data Availability statement in the metadata.

**Response:** Thank you for the above suggestions. We have updated the data availability statement and provided clear rationale/justification to support why full study dataset cannot be directly shared publicly. De-identified data and/codes may be made available upon reasonable request by contacting an independent non-study person, data manager at the institution who has access to the full study dataset and statistical analysis codes. Refer to page 18.

STATISTICAL REPORTING:

Thank you for amending your statistical reporting. However, there are still instances in the text and the tables where this is not correct. Please revise throughout the manuscript, including tables and figures.

- Please report statistical information as follows to improve clarity for the reader ""22% (95% CI [13,28]; p</=)"".

- Please separate upper and lower bounds with commas instead of hyphens as the latter can be confused with reporting of negative values.

- Please repeat statistical definitions (HR, CI etc.) for each set of parentheses.

**Response:** Thank you for highlighting this. We have now carefully reviewed the manuscript and incorporated above suggestions throughout the manuscript, abstract and results section.

*In the tables and figures, we appreciate you used a comma to separate the 95% CIs. However, please report these in square brackets rather than round ones, according to our guidelines.

*In the text, please follow our guidelines for statistical reporting. This includes separating the 95% CI values with a comma (not a hyphen), and presenting these values within square brackets.

**Response:** thank you, we have incorporated these suggestions in the Tables/Figures, and manuscript text.

TABLES AND FIGURES

* When a p value is given, please specify the statistical test used to determine it in the legend. Please declare in the legend of Table 2 which statistical test was used to calculate the presented p-values.

**Response.** Thank you, we have specified the statistical test used to determine where ever p-value is given in the Tables legend.
