# Supplementary material for: Assessing a multicomponent quality improvement strategy for chronic care of cardiovascular diseases in India: The C-QIP randomized feasibility trial
Source: PLoS Med. 2026 Sep 29;23(9):e1004932. doi: 10.1371/journal.pmed.1004932 (PMC13641716; doi:10.1371/journal.pmed.1004932)
Supplement: Peer review history

# Editor report: Decision Letter 0

**DOI:** 10.1371/journal.pmed.1004932.r001

**Authors:** Suzanne De Bruijn

23 Jan 2026

Dear Dr Singh,

Thank you for submitting your manuscript entitled "A Randomized Feasibility Trial of a Multicomponent Quality Improvement Strategy for Chronic Care of Cardiovascular Diseases: Findings from the C-QIP Trial in India" for consideration by PLOS Medicine.

Your manuscript has now been evaluated by the PLOS Medicine editorial staff, and I am writing to let you know that we would like to send your submission out for external peer review.

However, we noted some discrepancies in inclusion/exclusion criteria between the protocol and the manuscript. Could you please provide an explanation for these discrepancies, and where necessary add them to the methods, clearly stating when and why they were changed?

Furthermore, before we can send your manuscript to reviewers, we need you to complete your submission by providing the metadata that is required for full assessment. To this end, please login to Editorial Manager where you will find the paper in the 'Submissions Needing Revisions' folder on your homepage. Please click 'Revise Submission' from the Action Links and complete all additional questions in the submission questionnaire.

For clinical studies, please upload a copy of your trial study protocol as a supporting information file. The study protocol should be the version submitted for approval to the institutional review board or ethics committee, should include any amendments to the study protocol, as well as the date of their approval by the institutional review or ethics committee. Please also detail any deviations from the study protocol in the Methods section of your manuscript. The editors will consider the protocol and study conduct prior to a final decision for external review.

Please re-submit your manuscript within two working days, i.e. by Jan 27 2026 11:59PM.

Login to Editorial Manager here: https://www.editorialmanager.com/pmedicine

Once your full submission is complete, your paper will undergo a series of checks in preparation for peer review. Once your manuscript has passed all checks it will be sent out for review.

Feel free to email us at plosmedicine@plos.org if you have any queries relating to your submission.

Kind regards,

Suzanne De Bruijn, PhD

Associate Editor

PLOS Medicine

# Peer review: Decision Letter 1

**DOI:** 10.1371/journal.pmed.1004932.r002

**Authors:** Suzanne De Bruijn

24 Apr 2026

Dear Dr Singh,

Many thanks for submitting your manuscript "A Randomized Feasibility Trial of a Multicomponent Quality Improvement Strategy for Chronic Care of Cardiovascular Diseases: Findings from the C-QIP Trial in India" (PMEDICINE-D-26-00187R1) to PLOS Medicine. The paper has been reviewed by subject experts and a statistician; their comments are included below and can also be accessed here: [LINK]

As you will see, the reviewers think your study is interesting, but there are several major concerns. After discussing the paper with the editorial team and an academic editor with relevant expertise, I'm pleased to invite you to revise the paper in response to the reviewers' comments. We would like you to address all comments. Furthermore, we are expecting robust and transparent responses to the concerns regarding the statistical analysis, and regarding the deviations from the statistical analysis plan, which will be crucial for us to consider your paper further. We plan to send the revised paper to some or all of the original reviewers, and we cannot provide any guarantees at this stage regarding publication.

We further ask you to address our editorial requests, which you can find at the bottom of this email. Please also include these in your point-by-point response. In addition to these revisions, you may need to complete some formatting changes, which you will receive in a follow up email. A member of our team will be in touch with a set of requests shortly. If you do not receive a separate email within a few days, please assume that checks have been completed, and no additional changes are required.

When you upload your revision, please include a point-by-point response that addresses all of the reviewer and editorial points (that can be found at the bottom of this email), indicating the changes made in the manuscript and either an excerpt of the revised text or the location (eg: page and line number) where each change can be found. Please also be sure to check the general editorial comments at the end of this letter and include these in your point-by-point response. When you resubmit your paper, please include a clean version of the paper as the main article file and a version with changes tracked as a marked-up manuscript. It may also be helpful to check the guidelines for revised papers at http://journals.plos.org/plosmedicine/s/revising-your-manuscript for any that apply to your paper.

We ask that you submit your revision by May 15 2026 11:59PM. However, if this deadline is not feasible, please contact me by email, and we can discuss a suitable alternative.

Don't hesitate to contact me directly with any questions (sbruijn@plos.org).

Best regards,

Suzanne

Suzanne De Bruijn, PhD

Associate Editor

PLOS Medicine

sbruijn@plos.org

-----------------------------------------------------------

Comments from the academic editor:

The departure from the initial statistical analysis plan is a serious concern, and how the authors address this will be fundamental.

-----------------------------------------------------------

Comments from the reviewers:

Reviewer #1: Major Comments

1) The statistical analysis is described as confirmatory in several sections (e.g., use of hypothesis testing with a fixed α=0.05), despite the trial being explicitly positioned as a feasibility and hybrid type 1 implementation study. For feasibility trials, emphasis should be placed on estimation (e.g., point estimates with confidence intervals) rather than formal hypothesis testing. The current analytical framing risks overstating inferential claims that the study is not powered to support.

2) The SAP indicates that baseline differences between randomised groups will be tested using significance tests (t-tests, Wilcoxon tests, chi-square tests). This practice is discouraged in randomised trials, as any observed imbalance is due to chance rather than bias. Baseline characteristics should be presented descriptively without hypothesis testing, and any covariate adjustment should be prespecified based on clinical relevance rather than statistical significance at baseline.

3) For secondary outcomes, covariates included in adjusted models are described as those "identified as significant at baseline comparisons." This data-driven approach introduces a risk of overfitting and post-randomisation bias. Covariates for adjustment should be prespecified a priori based on clinical or methodological rationale, not on baseline p-values.

4) The choice of log-binomial GEE models for binary outcomes is appropriate in principle; however, the SAP lacks sufficient detail regarding the assumed working correlation structure, robustness of standard errors, and how clustering by site (four clinics) will be handled. Given the multi-site design, failure to explicitly address site-level clustering may result in underestimated uncertainty.

5) The plan to reallocate "end of study" measurements to scheduled visits (12 or 24 months) is insufficiently justified and may introduce measurement error or misclassification of follow-up time. A clearer definition of the estimand and handling of variable follow-up duration is required, particularly given the stated average follow-up of ~18 months.

6) Missing data are stated to be handled via complete-case analysis, with no imputation planned. This assumption implicitly requires data to be missing completely at random (MCAR), which is unlikely in longitudinal behavioural and clinical outcomes. At minimum, the authors should justify the plausibility of MCAR, report the extent and patterns of missingness, and consider sensitivity analyses (e.g., multiple imputation or inverse probability weighting).

7) Multiple secondary and exploratory outcomes are analysed without any discussion of multiplicity or control of false-positive findings. Even in feasibility studies, the lack of a clear hierarchy or interpretive framework for multiple comparisons limits the credibility of reported p-values.

8) Subgroup analyses are extensive and include numerous patient characteristics. Although the SAP notes that the study is not powered for these analyses, there is no clear guidance on how these results will be interpreted or reported. Given the high risk of spurious findings, subgroup analyses should be substantially reduced, clearly labelled as exploratory, and interpreted strictly qualitatively.

9) The integration of qualitative and quantitative data for implementation outcomes ("data triangulation") is conceptually appropriate, but the statistical analysis plan does not specify how qualitative findings will be systematically incorporated alongside quantitative estimates. A clearer mixed-methods analytical framework is needed to ensure transparency and reproducibility.

Minor Comments

10) The SAP alternates between describing outcomes at 12 months, 24 months, and "end of study," which creates ambiguity regarding the primary time point for analysis. Consistent terminology and a clearly defined primary analysis time point would improve clarity.

11) The use of compound symmetry as the assumed covariance structure in GEE models is stated without justification. The authors should explain why this structure is appropriate or indicate whether alternative structures were explored.

12) For continuous outcomes (e.g., systolic blood pressure, LDL cholesterol), it is unclear whether model assumptions (e.g., normality, linearity) will be assessed and how violations will be handled.

13) The SAP states that pairwise comparisons between treatment groups at each visit will be performed, but it does not clarify whether these are prespecified contrasts or post hoc analyses, nor how they relate to the primary treatment-by-time interaction.

14) Reporting of effect sizes is inconsistent across outcomes. A standardised approach (e.g., risk ratios with 95% confidence intervals for binary outcomes; mean differences with 95% confidence intervals for continuous outcomes) should be explicitly stated.

15) The description of safety analyses is minimal and largely descriptive. Clarification is needed on whether any comparative safety analyses between groups are planned and how exposure time will be accounted for.

16) Although STATA version 18 is specified, no details are provided on reproducibility practices (e.g., prespecified analysis scripts, version control, or audit trails), which are increasingly expected in contemporary clinical trials.

Reviewer #2: Summary

This manuscript reports the C-QIP feasibility RCT, evaluating a five-component quality improvement strategy (cardiovascular care coordinator, EHR-DSS, SMS reminders, patient diary/VITA, and audit-feedback) for chronic CVD care across four Indian outpatient sites (n=410; mean follow-up ~15 months). The study addresses an important implementation gap in LMIC cardiovascular care and is appropriately scoped as a feasibility trial. High retention, strong fidelity, and meaningful GDMT improvements in the coronary heart disease and ischemic stroke subgroups are commendable. However, several methodological and reporting concerns require revision before the manuscript is suitable for publication.

Major Concerns

1. Internal Numerical Inconsistencies

1.The abstract states 'mean follow-up 18 months', but the results report a median of 15.3/15.1 months (C-QIP/usual care) and Figure 1 implies ~459/452 days. The SAP's 18-month figure is an expected mean under a variable follow-up design; the observed value differed. The abstract must be corrected and the discrepancy acknowledged.

2.The abstract reports ischemic stroke 18.5% and heart failure 18.0%, but Table 1 shows HF 22.7%/18.2% and stroke 15.2%/21.7% by arm. These figures must be reconciled with the subgroup Ns in Table 2 (stroke N=74, HF N=82) throughout.

3.Figure 1 lists 11 vs 1 dropouts and 9 vs 10 deaths, but the text implies different totals. A CONSORT-compatible flow diagram with transparent accounting of all post-randomisation losses is required.

2. Contamination Risk

Individual-level randomisation within shared outpatient clinics creates a plausible and unquantified contamination risk: providers reviewing DSS prompts for C-QIP patients may modify care for controls attending the same clinics. The manuscript acknowledges this but provides no quantitative assessment (e.g., site-stratified comparison of control-arm GDMT rates). Given that future confirmatory trials will be powered on these effect estimates — notably the RR=2.41 for ischemic stroke GDMT (n=74) — overestimation due to contamination is a critical issue. A sensitivity analysis or, at minimum, a structured discussion of the likely direction and magnitude of bias is required.

3. Deviations from the Pre-specified SAP

4.The SAP specifies a treatment-by-time interaction term in all GEE models; this appears absent from the analyses as reported. Any departure from the pre-specified model must be disclosed.

5.The SAP describes reallocating EOS data to a scheduled visit timepoint but does not define the algorithm; the manuscript does not clarify this either. Please explain how EOS data were handled.

6.Three pre-specified subgroup variables (education, income, presenting condition) are absent from Figures S.2-S.3 without explanation.

7.No imputation was planned for continuous outcomes, but the handling of the 19 deceased participants in GEE ITT analyses is not described. A sensitivity analysis is warranted.

4. Heart Failure GDMT Inconsistency

The Methods define HF GDMT as a four-drug bundle (ACEi/ARB/ARNI + beta-blocker + MRA + SGLT2i), yet the abstract and primary results focus on the three-drug bundle, omitting SGLT2i. The null finding for SGLT2i (42.2% vs 43.2%, RR=1.28, p=0.237) — a now-essential HFrEF therapy — should be explicitly reported and discussed. Potential explanations (cost, availability, formulary access in India) should be addressed.

Minor Concerns

*Abstract exceeds the 300-word PLOS Medicine limit (currently 418 words); condensation is required.

*Alcohol counselling fidelity fell from 83% to 33% at EOS (Table S.1, n=6): this striking decline contradicts the high-fidelity narrative and requires explanation.

*The direction of effect for the physical health T-score appears contradictory: mean 46.9 (C-QIP) vs 48.6 (usual care), yet beta=+0.95 in favour of C-QIP. Please verify and clarify.

*Significant subgroup interactions (private facility, ischemic stroke) are mentioned but not quantified in the text; numerical estimates and a brief discussion of their implications for scale-up are needed.

*The SAP pre-specifies cost analysis and a qualitative (CFIR-based) component as outcomes. Neither is reported, nor is their omission acknowledged. Please clarify the reporting status of both.

*The screening-to-randomisation data in Figure 1 lack detail on reasons for ineligibility; this is standard for feasibility trial reporting (cf. Eldridge et al., CONSORT 2016 extension for pilot/feasibility trials).

*'Poison regression' in the SAP (page 10) should read 'Poisson'; please confirm this typographical error is not carried into the manuscript.

Reviewer #3: This is a well presented RCT with 410 participants of a QI strategy for cardiovascular health in India. I have the following suggestions/concerns/recommendations.

*Abstract has no aim. Not ideal in abstracts to say "implementation outcomes were "strong" - I recommend just putting the data with a direction rather than persuasive language. The measures of retention and fidelity seem to be a % but no outcome measure so cannot understand what the numbers mean. Also, how many patients received lifestyle advice appears to be int eh results. I find the abstract somewhat biased in its reporting. Although conclusion seems to reflect the findings.

*Suggest an overview of the intervention be provided in a table as the current text is dense. The intervention is very complex which is 'ok' but does make it a little complex to replicate given the current text. Perhaps also consider using the Tidier checklist or similar?

*I have some concerns about individual patient level randomisation when intervention was a hospital/practice level - how was cross-over managed?

*The outcomes are more clearly detailed in the manuscript but I admit I have a concern re the multiple primary outcomes. I appreciate it is an implementation trial but I find the multiple (4x and from both patient and provider perspectives) primary outcomes a problem because impossible to know if the intervention as actually effective.

* In outcomes section would also be good to have the timepoints of measurement.

*Overall I found the follow-up times unclear and confusing eg "post-randomization follow-up period ranged from 12 - 24 months, depending on participant enrolment in the trial." That is, when were patients follow-up? At 12 and 24-months or something different.

*For the "Comparison with other trials" on p10. I recommend consideration of the QUEL cluster RCT that also has a parallel process evaluation (Process evaluation of a data-driven quality improvement program within a cluster randomised controlled trial to improve coronary heart disease management in Australian primary care | PLOS One). The preprint is here and this paper is in press with Circ Population and Outcomes Effectiveness of data-driven quality improvement on hospitalizations and health outcomes for people with coronary heart disease in primary care (QUEL): a cluster randomised controlled trial with 24-month follow-up | medRxiv and here is the protocol (QUality improvement in primary care to prevent hospitalisations and improve Effectiveness and efficiency of care for people Living with coronary heart disease (QUEL): protocol for a 24-month cluster randomised controlled trial in primary care | BMC Primary Care | Springer Nature Link). There is also the QUICR trial that is ongoing with results still not published but here is the protocol (Data-driven collaborative QUality improvement in Cardiac Rehabilitation (QUICR) to increase program completion: protocol for a cluster randomized controlled trial | BMC Cardiovascular Disorders | Springer Nature Link). Preprint of results is available here (https://www.medrxiv.org/content/10.1101/2025.06.12.25329537v1?__cf_chl_tk=DulwDDSRzteLPFcSGFsgEeo5G1dvJJTb50XCMpf6dRE-1775978684-1.0.1.1-AwHUvrgAneQ6vXILPTel8wdpUZixEN9o3EGLW4oT5hs ) with main paper to be published in Circ: Cardiovascular Population Health and Outcomes in coming days (DOICIRCCQO-2025-012904-T). I feel these studies are highly relevant to the current paper.

*There are a lot of abbreviations and I would recommend trying to significantly reduce eg EOS, C-QIP, CCB, ARNI, GDMT, DSS. I think this would improve readability.

Any attachments provided with reviews can be seen via the following link: [LINK]

--------------------------------------------------------- ---

General editorial requests:

*Please upload any figures associated with your paper as individual TIF or EPS files with 300dpi resolution at resubmission; please read our figure guidelines for more information on our requirements: http://journals.plos.org/plosmedicine/s/figures. While revising your submission, we strongly recommend that you use PLOS's NAAS tool (https://ngplosjournals.pagemajik.ai/artanalysis) to test your figure files. NAAS can convert your figure files to the TIFF file type and meet basic requirements (such as print size, resolution), or provide you with a report on issues that do not meet our requirements and that NAAS cannot fix.

After uploading your figures to PLOS's NAAS tool - https://ngplosjournals.pagemajik.ai/artanalysis, NAAS will process the files provided and display the results in the "Uploaded Files" section of the page as the processing is complete.

If the uploaded figures meet our requirements (or NAAS is able to fix the files to meet our requirements), the figure will be marked as "fixed" above. If NAAS is unable to fix the files, a red "failed" label will appear above.

When NAAS has confirmed that the figure files meet our requirements, please download the file via the download option, and include these NAAS processed figure files when submitting your revised manuscript.

*Please ensure that the paper adheres to the PLOS Data Availability Policy (see http://journals.plos.org/plosmedicine/s/data-availability), which requires that all data underlying the study's findings be provided in a repository or as Supporting Information. For data residing with a third party, authors are required to provide instructions with contact information (web or email address) for obtaining the data. Please note that a study author cannot be the contact person for the data. PLOS journals do not allow statements supported by "data not shown" or "unpublished results." For such statements, authors must provide supporting data or cite public sources that include it.

* Data availability statement: Please note that your current data availability statement is not sufficient, and we do not allow an author of the study to be the point of contact. Please modify this statement, according to the guidelines above.

*We expect all researchers with submissions to PLOS in which author-generated code underpins the findings in the manuscript to make all author-generated code available without restrictions upon publication of the work. In cases where code is central to the manuscript, we may require the code to be made available as a condition of publication. Authors are responsible for ensuring that the code is reusable and well documented. Please make any custom code available, either as part of your data deposition or as a supplementary file. Please add a sentence to your data availability statement regarding any code used in the study, e.g. "The code used in the analysis is available from Github [URL] and archived in Zenodo [DOI link]" Please review our guidelines at https://journals.plos.org/plosmedicine/s/materials-software-and-code-sharing and ensure that your code is shared in a way that follows best practice and facilitates reproducibility and reuse. Because Github depositions can be readily changed or deleted, we encourage you to make a permanent DOI'd copy (e.g. in Zenodo) and provide the URL.

* Financial disclosure: Please include the initials of the person who obtained the grant, as well as a URL to the funders' website.

* Ethics statement: Thank you for stating that signed consent forms were obtained. Please specify whether this was informed consent.

FORMATTING - GENERAL

* Abstract: Please include the main limitations of your study at the end of the Methods and Findings section.

*At this stage, we ask that you include a short, non-technical Author Summary of your research to make findings accessible to a wide audience that includes both scientists and non-scientists. The Author Summary should immediately follow the Abstract in your revised manuscript. This text is subject to editorial change and should be distinct from the scientific abstract. Ideally each sub-heading should contain 2-3 single sentence, concise bullet points containing the most salient points from your study. In the final bullet point of 'What Do These Findings Mean?', please include the main limitations of the study in non-technical language. Please see our author guidelines for more information: https://journals.plos.org/plosmedicine/s/revising-your-manuscript#loc-author-summary.

*Please express the main results with 95% CIs as well as p values. When reporting p values please report as p<0.001 and where higher as the exact p value p=0.002, for example. Throughout, suggest reporting statistical information as follows to improve clarity for the reader "22% (95% CI [13%,28%]; p</=)". Please be sure to define all numerical values at first use.

*Please include page numbers and line numbers in the manuscript file. Use continuous line numbers (do not restart the numbering on each page).

*Please cite the reference numbers in square brackets. Citations should precede punctuation.

FIGURES AND TABLES

*Please provide titles and legends for all figures and tables (including those in Supporting Information files).

*Please define all abbreviations used in each figure/table (including those in Supporting Information files).

SUPPLEMENTARY MATERIAL

*Please note that supplementary material will be posted as supplied by the authors. Therefore, please amend it according to the relevant comments outlined here.

*Please cite your Supporting Information as outlined here: https://journals.plos.org/plosmedicine/s/supporting-information

REFERENCES

*PLOS uses the numbered citation (citation-sequence) method and first six authors, et al.

*Please ensure that journal name abbreviations match those found in the National Center for Biotechnology Information (NCBI) databases (http://www.ncbi.nlm.nih.gov/nlmcatalog/journals), and are appropriately formatted and capitalised.

*Where website addresses are cited, please include the complete URL and specify the date of access (e.g. [accessed: 12/06/2023]).

*Please also see https://journals.plos.org/plosmedicine/s/submission-guidelines#loc-references for further details on reference formatting.

RCTs

*Please structure the Methods section using the following sub-headings: Study design and participants, Randomization and masking, Procedures, Outcomes, Statistical analysis.

*Please ensure that all prespecified outcomes (primary, secondary, and exploratory) are listed in the Methods/Outcomes section and indicate whether there are outcomes that are not presented in the current report.

*Please specify the dates (Month Day, Year) during which study enrollment and follow up occurred.

*Please include absolute numbers wherever you report percentages; eg, n/N (%)

*Please present the safety data for the study including numbers of specific events and whether or not adverse events are thought to be related to treatment. AEs should be reported in the abstract, per CONSORT and CONSORT-Harms.

*Thank you for including the CONSORT checklist. We ask you to use use section and paragraph numbers, rather than page numbers. Please modify

*Please report your abstract according to CONSORT for abstracts, following the PLOS Medicine abstract structure (Background, Methods and Findings, Conclusions) https://www.equator-network.org/reporting-guidelines/consort-abstracts/

Attachment Submitted filename: CQIP_confidential_editor.docx

# Author comment: Author response to Decision Letter 2

**DOI:** 10.1371/journal.pmed.1004932.r003

1 Jun 2026

Attachment Submitted filename: Response_29.05.2026.docx

# Peer review: Decision Letter 2

**DOI:** 10.1371/journal.pmed.1004932.r004

**Authors:** Suzanne De Bruijn

3 Jul 2026

Dear Dr Singh,

Many thanks for submitting your manuscript "A Randomized Feasibility Trial of a Multicomponent Quality Improvement Strategy for Chronic Care of Cardiovascular Diseases: Findings from the C-QIP Trial in India" (PMEDICINE-D-26-00187R2) to PLOS Medicine. The paper has been reviewed by subject experts and a statistician; their comments are included below and can also be accessed here: [LINK]

As you will see, the reviewers think the manuscript has substantially improved, but reviewer #1 has some remaining concerns. After discussing the paper with the editorial team and an academic editor with relevant expertise, I'm pleased to invite you to revise the paper in response to the reviewers' comments. We plan to send the revised paper to some or all of the original reviewers, and we cannot provide any guarantees at this stage regarding publication.

Furthermore, we ask you to address all editorial requests, which you can find at the bottom of this email. In addition to these revisions, you may need to complete some formatting changes, which you will receive in a follow up email. A member of our team will be in touch with a set of requests shortly. If you do not receive a separate email within a few days, please assume that checks have been completed, and no additional changes are required.

When you upload your revision, please include a point-by-point response that addresses all of the reviewer and editorial points, indicating the changes made in the manuscript and either an excerpt of the revised text or the location (eg: page and line number) where each change can be found. Please also be sure to check the general editorial comments at the end of this letter and include these in your point-by-point response. When you resubmit your paper, please include a clean version of the paper as the main article file and a version with changes tracked as a marked-up manuscript. It may also be helpful to check the guidelines for revised papers at http://journals.plos.org/plosmedicine/s/revising-your-manuscript for any that apply to your paper.

We ask that you submit your revision by Jul 24 2026 11:59PM. However, if this deadline is not feasible, please contact me by email, and we can discuss a suitable alternative.

Don't hesitate to contact me directly with any questions (sbruijn@plos.org).

Best regards,

Suzanne

Suzanne De Bruijn, PhD

Associate Editor

PLOS Medicine

sbruijn@plos.org

-----------------------------------------------------------

Comments from the academic editor:

To be able to respond to the reviewers the authors have had to conduct many new analyses (including sensitivity analyses etc.). These added to the initial analyses can amount to 'multiple comparisons' and risk of chance findings. While the authors do not need to adjust for multiple comparisons; it could make sense to ask them to comment on the possibility of chance findings from multiple comparison, and whether this shouldn't perhaps be included in the limitations.

-----------------------------------------------------------

Comments from the reviewers:

Reviewer #1: The authors have addressed the major methodological and statistical concerns raised in the previous review. The manuscript has been substantially improved, particularly with respect to the feasibility-trial framing, clarification of the statistical analysis plan deviations, prespecified covariate adjustment, handling of missing data, subgroup analyses, and interpretation of secondary outcomes. I have only two remaining statistical comments:

a) The addition of IPTW sensitivity analyses is appreciated. Please briefly summarize in the manuscript whether the conclusions from the IPTW analyses differed materially from the primary analyses and comment on the implications for the robustness of the findings to missing-data assumptions.

b) Model diagnostics: The manuscript indicates that model assumptions for continuous outcomes were assessed. Please provide a brief description of the diagnostic procedures performed (e.g., assessment of residual distributions and model fit) and indicate whether any important violations were identified.

Reviewer #2: The author have adequately addressed the suggestions.

Any attachments provided with reviews can be seen via the following link: [LINK]

--------------------------------------------------------- ---

General editorial requests:

GENERAL

* Please confirm that your title complies with PLOS Medicine's style. Your title must be nondeclarative and not a question. It should begin with main concept if possible. "Effect of" should be used only if causality can be inferred, i.e., for an RCT. Please place the study design ("A randomized controlled trial," "A retrospective study," "A modelling study," etc.) in the subtitle (ie, after a colon). Please modify your title accordingly.

* Please ensure that all abbreviations are defined at first use throughout the text.

* Please confirm that all numbers presented in the abstract are present and identical to numbers presented in the main manuscript text.

*Please ensure that the paper adheres to the PLOS Data Availability Policy (see http://journals.plos.org/plosmedicine/s/data-availability), which requires that all data underlying the study's findings be provided in a repository or as Supporting Information. For data residing with a third party, authors are required to provide instructions with contact information (web or email address) for obtaining the data. Please note that a study author cannot be the contact person for the data. PLOS journals do not allow statements supported by "data not shown" or "unpublished results." For such statements, authors must provide supporting data or cite public sources that include it.

* Please provide the data, or provide reasoning why this is legally not allowed.

*We expect all researchers with submissions to PLOS in which author-generated code underpins the findings in the manuscript to make all author-generated code available without restrictions upon publication of the work. In cases where code is central to the manuscript, we require the code to be made available as a condition of publication. Authors are responsible for ensuring that the code is reusable and well documented. Please make any custom code available, either as part of your data deposition or as a supplementary file. Please add a sentence to your data availability statement regarding any code used in the study, e.g. "The code used in the analysis is available from Github [URL] and archived in Zenodo [DOI link]" Please review our guidelines at https://journals.plos.org/plosmedicine/s/materials-software-and-code-sharing and ensure that your code is shared in a way that follows best practice and facilitates reproducibility and reuse. Because Github depositions can be readily changed or deleted, we encourage you to make a permanent DOI'd copy (e.g. in Zenodo) and provide the URL.

* Please provide a statement on where the code can be found in your data availability statement.

* Please disclose any use of AI tools and technologies to the study or article content at the end of the Methods. Please specify the name(s) and version(s) of any tool(s) used and a description of their use, including how the AI-assisted or generated content was evaluated by authors, and a clear statement of which aspects of the study, article contents, data, or supporting files were affected/generated by AI tool usage. Please also include a statement specifying who retains responsibility for the final manuscript. See our guidelines for more information: https://journals.plos.org/plosmedicine/s/ethical-publishing-practice#loc-artificial-intelligence-tools-and-technologies.

* In the author summary, in the final bullet point of 'What Do These Findings Mean?', please include the main limitations of the study in non-technical language.

* Statistical reporting: Please revise throughout the manuscript, including tables and figures.

- Please report statistical information as follows to improve clarity for the reader ""22% (95% CI [13,28]; p</=)"".

- Please separate upper and lower bounds with commas instead of hyphens as the latter can be confused with reporting of negative values.

- Please repeat statistical definitions (HR, CI etc.) for each set of parentheses.

* Please remove the 'conclusions' subheading from the discussion. Please also remove any other subheadings from the discussion.

FUNDING STATEMENT

* The funding statement should include: specific grant numbers, initials of authors who received each award, URLs to sponsors’ websites. Also, please state whether any sponsors or funders (other than the named authors) played any role in study design, data collection and analysis, the decision to publish, or preparation of the manuscript. If they had no role in the research, include this sentence: “The funders had no role in study design, data collection and analysis, decision to publish, or preparation of the manuscript.”

* Please clarify the funder of grant “K24HL175228 to MDH” and provide a URL to the funders website.

DATA AVAILABILITY

* PLOS Medicine requires that the de-identified data underlying the specific results in a published article be made available, without restrictions on access, in a public repository or as Supporting Information at the time of article publication, provided it is legal and ethical to do so. Please see the policy at http://journals.plos.org/plosmedicine/s/data-availability and FAQs at

http://journals.plos.org/plosmedicine/s/data-availability#loc-faqs-for-data-policy

* Please include the statement on code availability in the data availability statement. Please see guidance here: https://journals.plos.org/plosmedicine/s/code-sharing-guidance#loc-data-availability-statement-examples.

TABLES AND FIGURES

* When a p value is given, please specify the statistical test used to determine it in the legend.

CLINICAL TRIALS

* Please complete the CONSORT 2025 checklist and ensure that all components of CONSORT 2025 are present in the manuscript, including how randomization was performed, allocation concealment, blinding of intervention, definition of lost to follow-up, power statement. When completing the checklist, please use section and paragraph numbers, rather than page numbers. The checklist should be included as supporting information, and should be cited in the article.

* Thank you for including the CONSORT checklist. We ask you to use use section and paragraph numbers, rather than page numbers. Please modify. I appreciate we asked this previously, but the CONSORT checklist that is currently uploaded still uses page numbers.

* Please add the following statement, or similar, to the Methods: "This study is reported as per the Consolidated Standards of Reporting Trials (CONSORT) guidelines (S1 Checklist)."

# Author comment: Author response to Decision Letter 3

**DOI:** 10.1371/journal.pmed.1004932.r005

21 Jul 2026

Attachment Submitted filename: Response_06.07.2026.docx

# Editor report: Decision Letter 3

**DOI:** 10.1371/journal.pmed.1004932.r006

**Authors:** Suzanne De Bruijn

27 Jul 2026

Dear Dr. Singh,

Thank you very much for re-submitting your manuscript "A Randomized Feasibility Trial of a Multicomponent Quality Improvement Strategy for Chronic Care of Cardiovascular Diseases: Findings from the C-QIP Trial in India" (PMEDICINE-D-26-00187R3) for review by PLOS Medicine.

I have discussed the paper with my colleagues, and I am pleased to say that provided the remaining editorial and production issues are dealt with we are planning to accept the paper for publication in the journal.

The remaining issues that need to be addressed are listed at the end of this email. Please provide a point-by-point response.

***Please note while forming your response, if your article is accepted, you may have the opportunity to make the peer review history publicly available. The record will include editor decision letters (with reviews) and your responses to reviewer comments. If eligible, we will contact you to opt in or out.***

In revising the manuscript for further consideration here, please ensure you address the specific points made by each reviewer and the editors. In your rebuttal letter you should indicate your response to the reviewers' and editors' comments and the changes you have made in the manuscript. Please submit a clean version of the paper as the main article file. A version with changes marked must also be uploaded as a marked up manuscript file.

Please also check the guidelines for revised papers at http://journals.plos.org/plosmedicine/s/revising-your-manuscript for any that apply to your paper. If you haven't already, we ask that you provide a short, non-technical Author Summary of your research to make findings accessible to a wide audience that includes both scientists and non-scientists. The Author Summary should immediately follow the Abstract in your revised manuscript. This text is subject to editorial change and should be distinct from the scientific abstract.

In addition to these revisions, you may need to complete some formatting changes, which you will receive in a follow up email. A member of our team will be in touch with a set of requests shortly. If you do not receive a separate email within a few days, please assume that checks have been completed, and no additional changes are required.

We expect to receive your revised manuscript within 1 week. Please email us (plosmedicine@plos.org) if you have any questions or concerns.

Please review your reference list to ensure that it is complete and correct. If you have cited papers that have been retracted, please include the rationale for doing so in the manuscript text, or remove these references and replace them with relevant current references. Any changes to the reference list should be mentioned in the rebuttal letter that accompanies your revised manuscript.

Please note, when your manuscript is accepted, an uncorrected proof of your manuscript will be published online ahead of the final version, unless you've already opted out via the online submission form. If, for any reason, you do not want an earlier version of your manuscript published online or are unsure if you have already indicated as such, please let the journal staff know immediately at plosmedicine@plos.org.

If you have any questions in the meantime, please contact me or the journal staff on plosmedicine@plos.org.

We look forward to receiving the revised manuscript by Aug 03 2026 11:59PM.

Sincerely,

Suzanne De Bruijn, PhD

Associate Editor

PLOS Medicine

plosmedicine.org

------------------------------------------------------------

Requests from Editors:

TITLE:

* Please change your title to: "Assessing a Multicomponent Quality Improvement Strategy for Chronic Care of Cardiovascular Diseases in India: the c-QIP randomised feasibility trial".

DATA AVAILABILITY STATEMENT

*Thank you for providing contact details that are not the study author. However, we need you to include more detail into this statement. This include the reason why the data cannot be made available, and the requirements for researchers to gain access to the data (see point C below). We also need you to include a statement on where the code can be found in the Data Availability Statement, in the metadata of the manuscript.

* The Data Availability Statement (DAS) requires revision. For each data source used in your study:

a) If the data are freely or publicly available, note this and state the location of the data: within the paper, in Supporting Information files, or in a public repository (include the DOI or accession number).

b) If the data are owned by a third party but freely available upon request, please note this and state the institutional owner of the data set and contact information for data requests (institutional web or email address). Note that a study author cannot be the contact for the data.

c) If the data are not freely available, please describe briefly the ethical, legal, or contractual restriction that prevents you from sharing it. Please also include an appropriate institutional contact (institutional web or email address) for inquiries (again, this cannot be a study author).

-Code: Thank you for stating that the code will be archived in a permanent repository. Please provide the DOI in the Data Availability statement in the metadata.

STATISTICAL REPORTING:

Thank you for amending your statistical reporting. However, there are still instances in the text and the tables where this is not correct. Please revise throughout the manuscript, including tables and figures.

- Please report statistical information as follows to improve clarity for the reader ""22% (95% CI [13,28]; p</=)"".

- Please separate upper and lower bounds with commas instead of hyphens as the latter can be confused with reporting of negative values.

- Please repeat statistical definitions (HR, CI etc.) for each set of parentheses.

*In the tables and figures, we appreciate you used a comma to separate the 95% CIs. However, please report these in square brackets rather than round ones, according to our guidelines.

*In the text, please follow our guidelines for statistical reporting. This includes separating the 95% CI values with a comma (not a hyphen), and presenting these values within square brackets.

TABLES AND FIGURES

* When a p value is given, please specify the statistical test used to determine it in the legend. Please declare in the legend of Table 2 which statistical test was used to calculate the presented p-values.

# Author comment: Author response to Decision Letter 4

**DOI:** 10.1371/journal.pmed.1004932.r007

3 Aug 2026

Attachment Submitted filename: Response_28.07.2026.docx

# Editor report: Decision Letter 4

**DOI:** 10.1371/journal.pmed.1004932.r008

**Authors:** Suzanne De Bruijn

18 Aug 2026

Dear Dr Singh,

On behalf of my colleagues and the Academic Editor, Andre P Kengne, I am pleased to inform you that we have agreed to publish your manuscript "Assessing a Multicomponent Quality Improvement Strategy for Chronic Care of Cardiovascular Diseases in India: The C-QIP Randomized Feasibility Trial" (PMEDICINE-D-26-00187R4) in PLOS Medicine.

Before your manuscript can be formally accepted you will need to complete some formatting changes, which you will receive in a follow up email. Please be aware that it may take several days for you to receive this email; during this time no action is required by you. Once you have received these formatting requests, please note that your manuscript will not be scheduled for publication until you have made the required changes.

In the meantime, please log into Editorial Manager at http://www.editorialmanager.com/pmedicine/, click the "Update My Information" link at the top of the page, and update your user information to ensure an efficient production process.

PRESS

We frequently collaborate with press offices. If your institution or institutions have a press office, please notify them about your upcoming paper at this point, to enable them to help maximise its impact. If the press office is planning to promote your findings, we would be grateful if they could coordinate with medicinepress@plos.org. If you have not yet opted out of the early version process, we ask that you notify us immediately of any press plans so that we may do so on your behalf.

We also ask that you take this opportunity to read our Embargo Policy regarding the discussion, promotion and media coverage of work that is yet to be published by PLOS. As your manuscript is not yet published, it is bound by the conditions of our Embargo Policy. Please be aware that this policy is in place both to ensure that any press coverage of your article is fully substantiated and to provide a direct link between such coverage and the published work. For full details of our Embargo Policy, please visit http://www.plos.org/about/media-inquiries/embargo-policy/.

To enhance the reproducibility of your results, we recommend that you deposit your laboratory protocols in protocols.io, where a protocol can be assigned its own identifier (DOI) such that it can be cited independently in the future. Additionally, PLOS ONE offers an option to publish peer-reviewed clinical study protocols. Read more information on sharing protocols at https://plos.org/protocols?utm_medium=editorial-email&utm_source=authorletters&utm_campaign=protocols

Thank you again for submitting to PLOS Medicine. We look forward to publishing your paper.

Sincerely,

Suzanne De Bruijn, PhD

Associate Editor

PLOS Medicine
